# Diretrizes da Sociedade Brasileira de Cardiologia sobre Angina Instável e Infarto Agudo do Miocárdio sem Supradesnível do Segmento ST – 2021

**DOI:** 10.36660/abc.20210180

**Published:** 2021-07-15

**Authors:** José Carlos Nicolau, Gilson Soares Feitosa, João Luiz Petriz, Remo Holanda de Mendonça Furtado, Dalton Bertolim Précoma, Walmor Lemke, Renato Delascio Lopes, Ari Timerman, José A. Marin, Luiz Bezerra, Bruno Ferraz de Oliveira Gomes, Eduardo Cavalcanti Lapa Santos, Leopoldo Soares Piegas, Alexandre de Matos Soeiro, Alexandre Jorge de Andrade Negri, Andre Franci, Brivaldo Markman, Bruno Mendonça Baccaro, Carlos Eduardo Lucena Montenegro, Carlos Eduardo Rochitte, Carlos José Dornas Gonçalves Barbosa, Cláudio Marcelo Bittencourt das Virgens, Edson Stefanini, Euler Roberto Fernandes Manenti, Felipe Gallego Lima, Francisco das Chagas Monteiro, Harry Correa, Henrique Patrus Mundim Pena, Ibraim Masciarelli Francisco Pinto, João Luiz de Alencar Araripe Falcão, Joberto Pinheiro Sena, José Maria Peixoto, Juliana Ascenção de Souza, Leonardo Sara da Silva, Lilia Nigro Maia, Louis Nakayama Ohe, Luciano Moreira Baracioli, Luís Alberto de Oliveira Dallan, Luis Augusto Palma Dallan, Luiz Alberto Piva e Mattos, Luiz Carlos Bodanese, Luiz Eduardo Fonteles Ritt, Manoel Fernandes Canesin, Marcelo Bueno da Silva Rivas, Marcelo Franken, Marcos José Gomes Magalhães, Múcio Tavares de Oliveira, Nivaldo Menezes Filgueiras, Oscar Pereira Dutra, Otávio Rizzi Coelho, Paulo Ernesto Leães, Paulo Roberto Ferreira Rossi, Paulo Rogério Soares, Pedro Alves Lemos, Pedro Silvio Farsky, Rafael Rebêlo C. Cavalcanti, Renato Jorge Alves, Renato Abdala Karam Kalil, Roberto Esporcatte, Roberto Luiz Marino, Roberto Rocha Corrêa Veiga Giraldez, Romeu Sérgio Meneghelo, Ronaldo de Souza Leão Lima, Rui Fernando Ramos, Sandra Nivea dos Reis Saraiva Falcão, Talia Falcão Dalçóquio, Viviana de Mello Guzzo Lemke, William Azem Chalela, Wilson Mathias

**Affiliations:** 1 Hospital das Clínicas da Faculdade de Medicina da Universidade de São Paulo Instituto do Coração São PauloSP Brasil Instituto do Coração (InCor), Hospital das Clínicas da Faculdade de Medicina da Universidade de São Paulo (HCFMUSP), São Paulo, SP – Brasil; 2 Escola Bahiana de Medicina e Saúde Pública SalvadorBA Brasil Escola Bahiana de Medicina e Saúde Pública, Salvador, BA – Brasil; 3 Centro Universitário de Tecnologia e Ciência SalvadorBA Brasil Centro Universitário de Tecnologia e Ciência (UniFTC), Salvador, BA – Brasil; 4 Hospital Barra D'Or Rede D'Or São Luiz Rio de JaneiroRJ Brasil Hospital Barra D'Or, Rede D'Or São Luiz, Rio de Janeiro, RJ – Brasil; 5 Hospital Israelita Albert Einstein São PauloSP Brasil Hospital Israelita Albert Einstein, São Paulo, SP – Brasil; 6 Sociedade Hospitalar Angelina Caron Campina Grande do SulPR Brasil Sociedade Hospitalar Angelina Caron, Campina Grande do Sul, PR – Brasil; 7 Clínica Cardiocare CuritibaPR Brasil Clínica Cardiocare, Curitiba, PR – Brasil; 8 Hospital das Nações CuritibaPR Brasil Hospital das Nações, Curitiba, PR – Brasil; 9 Duke University Durham EUA Duke University, Durham – EUA; 10 Instituto Dante Pazzanese de Cardiologia São PauloSP Brasil Instituto Dante Pazzanese de Cardiologia, São Paulo, SP – Brasil; 11 Hospital das Clínicas da Faculdade de Medicina da Universidade de São Paulo Ribeirão PretoSP Brasil Hospital das Clínicas da Faculdade de Medicina da Universidade de São Paulo, Ribeirão Preto, SP – Brasil; 12 Centro Universitário UNINOVAFAPI TeresinaPI Brasil Centro Universitário UNINOVAFAPI, Teresina, PI – Brasil; 13 Universidade Federal do Rio de Janeiro Rio de JaneiroRJ Brasil Universidade Federal do Rio de Janeiro (UFRJ), Rio de Janeiro, RJ – Brasil; 14 Universidade Federal de Pernambuco Hospital das Clínicas RecifePE Brasil Hospital das Clínicas da Universidade Federal de Pernambuco (UFPE), Recife, PE – Brasil; 15 Hospital do Coração São PauloSP Brasil Hospital do Coração (HCor), São Paulo, SP – Brasil; 16 Faculdade de Medicina da Universidade de São Paulo São PauloSP Brasil Faculdade de Medicina da Universidade de São Paulo (HCFMUSP), São Paulo, SP – Brasil; 17 Hospital Universitário Lauro Wanderley João PessoaPB Brasil Hospital Universitário Lauro Wanderley, João Pessoa, PB – Brasil; 18 Hospital Sírio-Libanês São PauloSP Brasil Hospital Sírio-Libanês, São Paulo, SP – Brasil; 19 Pronto Socorro Cardiológico de Pernambuco RecifePE Brasil Pronto Socorro Cardiológico de Pernambuco (PROCAPE), Recife, PE – Brasil; 20 Hospital do Coração do Brasil Rede D'Or São Luiz BrasíliaDF Brasil Hospital do Coração do Brasil, Rede D'Or São Luiz, Brasília, DF – Brasil; 21 Universidade Federal da Bahia SalvadorBA Brasil Universidade Federal da Bahia (UFBA), Salvador, BA – Brasil; 22 Universidade Federal de São Paulo Escola Paulista de Medicina São PauloSP Brasil Escola Paulista de Medicina da Universidade Federal de São Paulo (UNIFESP), São Paulo, SP – Brasil; 23 Instituto de Medicina Vascular Mãe de Deus Porto AlegreRS Brasil Instituto de Medicina Vascular Mãe de Deus, Porto Alegre, RS – Brasil; 24 Universidade Federal do Maranhão São LuísMA Brasil Universidade Federal do Maranhão (UFMA), São Luís, MA – Brasil; 25 Clínica Unicardio FlorianópolisSC Brasil Clínica Unicardio, Florianópolis, SC – Brasil; 26 Faculdade de Ciências Médicas de Minas Gerais Belo HorizonteMG Brasil Faculdade de Ciências Médicas de Minas Gerais (FELUMA), Belo Horizonte, MG – Brasil; 27 Grupo Fleury São PauloSP Brasil Grupo Fleury, São Paulo, SP – Brasil; 28 Universidade Federal do Ceará FortalezaCE Brasil Universidade Federal do Ceará (UFC), Fortaleza, CE – Brasil; 29 Hospital Santa Izabel da Santa Casa da Bahia SalvadorBA Brasil Hospital Santa Izabel da Santa Casa da Bahia, Salvador, BA – Brasil; 30 Universidade José do Rosário Vellano Belo HorizonteMG Brasil Universidade José do Rosário Vellano (UNIFENAS), Belo Horizonte, MG – Brasil; 31 Centro de Diagnóstico por Imagem GoiâniaGO Brasil Centro de Diagnóstico por Imagem (CDI), Goiânia, GO – Brasil; 32 Faculdade de Medicina de São José do Rio Preto São José do Rio PretoSP Brasil Faculdade de Medicina de São José do Rio Preto (FAMERP), São José do Rio Preto, SP – Brasil; 33 Rede D'Or São Luiz RecifePE Brasil Rede D'Or São Luiz, Recife, PE – Brasil; 34 Pontifícia Universidade Católica do Rio Grande do Sul Porto AlegreRS Brasil Pontifícia Universidade Católica do Rio Grande do Sul (PUC-RS), Porto Alegre, RS – Brasil; 35 Hospital Cárdio Pulmonar SalvadorBA Brasil Hospital Cárdio Pulmonar, Salvador, BA – Brasil; 36 Universidade Estadual de Londrina LondrinaPR Brasil Universidade Estadual de Londrina, Londrina, PR – Brasil; 37 Rede D'Or São Luiz Rio de JaneiroRJ Brasil Rede D'Or São Luiz, Rio de Janeiro, RJ – Brasil; 38 Universidade do Estado do Rio de Janeiro Rio de JaneiroRJ Brasil Universidade do Estado do Rio de Janeiro (UERJ), Rio de Janeiro, RJ – Brasil; 39 Real Hospital Português São PauloSP Brasil Real Hospital Português, São Paulo, SP – Brasil; 40 Universidade do Estado da Bahia SalvadorBA Brasil Universidade do Estado da Bahia (UNEB), Salvador, BA – Brasil; 41 Universidade Salvador SalvadorBA Brasil Universidade Salvador (UNIFACS), Salvador, BA – Brasil; 42 Hospital EMEC SalvadorBA Brasil Hospital EMEC, Salvador, BA – Brasil; 43 Fundação Universitária de Cardiologia do Rio Grande do Sul Instituto de Cardiologia Porto AlegreRS Brasil Instituto de Cardiologia – Fundação Universitária de Cardiologia do Rio Grande do Sul, Porto Alegre, RS – Brasil; 44 Universidade Estadual de Campinas Faculdade de Ciências Médicas CampinasSP Brasil Faculdade de Ciências Médicas da Universidade Estadual de Campinas (UNICAMP), Campinas, SP – Brasil; 45 Santa Casa de Misericórdia de Porto Alegre Porto AlegreRS Brasil Santa Casa de Misericórdia de Porto Alegre, Porto Alegre, RS – Brasil; 46 Faculdade de Medicina Evangélica Mackenzie CuritibaPR Brasil Faculdade de Medicina Evangélica Mackenzie, Curitiba, PR – Brasil; 47 Hospital do Coração de Alagoas MaceióAL Brasil Hospital do Coração de Alagoas, Maceió, AL – Brasil; 48 Santa Casa de Misericórdia da São Paulo São PauloSP Brasil Santa Casa de Misericórdia da São Paulo, São Paulo, SP – Brasil; 49 Universidade Federal de Ciências da Saúde de Porto Alegre Porto AlegreRS Brasil Universidade Federal de Ciências da Saúde de Porto Alegre, Porto Alegre, RS – Brasil; 50 Hospital Madre Teresa Belo HorizonteMG Brasil Hospital Madre Teresa, Belo Horizonte, MG – Brasil; 51 Universidade Federal do Rio de Janeiro Rio de JaneiroRJ Brasil Universidade Federal do Rio de Janeiro (UFRJ), Rio de Janeiro, RJ – Brasil; 52 Universidade de Fortaleza FortalezaCE Brasil Universidade de Fortaleza (UNIFOR), Fortaleza, CE – Brasil; 53 Cardiocare Clínica Cardiológica CuritibaPR Brasil Cardiocare Clínica Cardiológica, Curitiba, PR – Brasil

**Table:** Diretrizes da Sociedade Brasileira de Cardiologia sobre Angina Instável e Infarto Agudo do Miocárdio sem Supradesnível do Segmento ST – 2021

O relatório abaixo lista as declarações de interesse conforme relatadas à SBC pelos especialistas durante o período de desenvolvimento desta diretriz, 2020.	
Especialista	Tipo de relacionamento com a indústria
Alexandre de Matos Soeiro	Nada a ser declarado
Alexandre Jorge de Andrade Negri	Declaração financeira A - Pagamento de qualquer espécie e desde que economicamente apreciáveis, feitos a (i) você, (ii) ao seu cônjuge / companheiro ou a qualquer outro membro que resida com você, (iii) a qualquer pessoa jurídica em que qualquer destes seja controlador, sócio, acionista ou participante, de forma direta ou indireta, recebimento por palestras, aulas, atuação como proctor de treinamentos, remunerações, honorários pagos por participações em conselhos consultivos, de investigadores, ou outros comitês, etc. Provenientes da indústria farmacêutica, de órteses, próteses, equipamentos e implantes, brasileiras ou estrangeiras: - AMIB: Instrutor de cursos de pós-graduação.
Andre Franci	Nada a ser declarado
Ari Timerman	Declaração financeira A - Pagamento de qualquer espécie e desde que economicamente apreciáveis, feitos a (i) você, (ii) ao seu cônjuge/companheiro ou a qualquer outro membro que resida com você, (iii) a qualquer pessoa jurídica em que qualquer destes seja controlador, sócio, acionista ou participante, de forma direta ou indireta, recebimento por palestras, aulas, atuação como proctor de treinamentos, remunerações, honorários pagos por participações em conselhos consultivos, de investigadores, ou outros comitês, etc. Provenientes da indústria farmacêutica, de órteses, próteses, equipamentos e implantes, brasileiras ou estrangeiras: - Sanofi e Daiichi Sankyo: Aulas sobre Síndromes Coronárias Agudas. Outros relacionamentos Financiamento de atividades de educação médica continuada, incluindo viagens, hospedagens e inscrições para congressos e cursos, provenientes da indústria farmacêutica, de órteses, próteses, equipamentos e implantes, brasileiras ou estrangeiras: - Sanofi: Viagem para Congresso.
Brivaldo Markman Filho	Nada a ser declarado
Bruno Ferraz de Oliveira Gomes	Nada a ser declarado
Bruno Mendonça Baccaro	Nada a ser declarado
Carlos Eduardo Lucena Montenegro	Declaração financeira A - Pagamento de qualquer espécie e desde que economicamente apreciáveis, feitos a (i) você, (ii) ao seu cônjuge/companheiro ou a qualquer outro membro que resida com você, (iii) a qualquer pessoa jurídica em que qualquer destes seja controlador, sócio, acionista ou participante, de forma direta ou indireta, recebimento por palestras, aulas, atuação como proctor de treinamentos, remunerações, honorários pagos por participações em conselhos consultivos, de investigadores, ou outros comitês, etc. Provenientes da indústria farmacêutica, de órteses, próteses, equipamentos e implantes, brasileiras ou estrangeiras: - Servier: Vastarel/Procoralan; Novartis: Entresto; Merk: Concor; Astrazeneca. Outros relacionamentos Financiamento de atividades de educação médica continuada, incluindo viagens, hospedagens e inscrições para congressos e cursos, provenientes da indústria farmacêutica, de órteses, próteses, equipamentos e implantes, brasileiras ou estrangeiras: - Pfizer: Amiloidose; Servier: DAC/IC; Novartis: IC.
Carlos Eduardo Rochitte	Nada a ser declarado
Carlos José Dornas Gonçalves Barbosa	Declaração financeira A - Pagamento de qualquer espécie e desde que economicamente apreciáveis, feitos a (i) você, (ii) ao seu cônjuge/companheiro ou a qualquer outro membro que resida com você, (iii) a qualquer pessoa jurídica em que qualquer destes seja controlador, sócio, acionista ou participante, de forma direta ou indireta, recebimento por palestras, aulas, atuação como proctor de treinamentos, remunerações, honorários pagos por participações em conselhos consultivos, de investigadores, ou outros comitês, etc. Provenientes da indústria farmacêutica, de órteses, próteses, equipamentos e implantes, brasileiras ou estrangeiras: - Daiichi Sankyo: Effient; Servier: Brilinta. Outros relacionamentos Financiamento de atividades de educação médica continuada, incluindo viagens, hospedagens e inscrições para congressos e cursos, provenientes da indústria farmacêutica, de órteses, próteses, equipamentos e implantes, brasileiras ou estrangeiras: - Bayer: Xarelto; Daiichi Sankyo: Lixiana.
Cláudio Marcelo Bittencourt das Virgens	Declaração financeira A - Pagamento de qualquer espécie e desde que economicamente apreciáveis, feitos a (i) você, (ii) ao seu cônjuge/companheiro ou a qualquer outro membro que resida com você, (iii) a qualquer pessoa jurídica em que qualquer destes seja controlador, sócio, acionista ou participante, de forma direta ou indireta, recebimento por palestras, aulas, atuação como proctor de treinamentos, remunerações, honorários pagos por participações em conselhos consultivos, de investigadores, ou outros comitês, etc. Provenientes da indústria farmacêutica, de órteses, próteses, equipamentos e implantes, brasileiras ou estrangeiras: - Novo Nordisk: Semaglutida; Daiichi Sankyo: Edoxabana.
Dalton Bertolim Précoma	Declaração financeira A - Pagamento de qualquer espécie e desde que economicamente apreciáveis, feitos a (i) você, (ii) ao seu cônjuge / companheiro ou a qualquer outro membro que resida com você, (iii) a qualquer pessoa jurídica em que qualquer destes seja controlador, sócio, acionista ou participante, de forma direta ou indireta, recebimento por palestras, aulas, atuação como proctor de treinamentos, remunerações, honorários pagos por participações em conselhos consultivos, de investigadores, ou outros comitês, etc. Provenientes da indústria farmacêutica, de órteses, próteses, equipamentos e implantes, brasileiras ou estrangeiras: - Servier: Doença Coronariana; Bayer: Anticoagulates; Daiichi Sankyo: Anticoagulantes; Novo Nordisk. B - financiamento de pesquisas sob sua responsabilidade direta/pessoal (direcionado ao departamento ou instituição) provenientes da indústria farmacêutica, de órteses, próteses, equipamentos e implantes, brasileiras ou estrangeiras: - Novartis: Insuficiência Cardíaca; Apellis: Antiinflamatório para Covid; Astrazeneca: Antidiabético/Insuficiência Cardíaca; Boehringer. Outros relacionamentos Financiamento de atividades de educação médica continuada, incluindo viagens, hospedagens e inscrições para congressos e cursos, provenientes da indústria farmacêutica, de órteses, próteses, equipamentos e implantes, brasileiras ou estrangeiras: - Bayer: Anticoagulates; Novo Nordisk: Antidiabético; Servier: DAC; Daiichi Sankyo.
Edson Stefanini	Nada a ser declarado
Eduardo Cavalcanti Lapa Santos	Nada a ser declarado
Euler Roberto Fernandes Manent	Declaração financeira A - Pagamento de qualquer espécie e desde que economicamente apreciáveis, feitos a (i) você, (ii) ao seu cônjuge / companheiro ou a qualquer outro membro que resida com você, (iii) a qualquer pessoa jurídica em que qualquer destes seja controlador, sócio, acionista ou participante, de forma direta ou indireta, recebimento por palestras, aulas, atuação como proctor de treinamentos, remunerações, honorários pagos por participações em conselhos consultivos, de investigadores, ou outros comitês, etc. Provenientes da indústria farmacêutica, de órteses, próteses, equipamentos e implantes, brasileiras ou estrangeiras: - Bayer e Pfizer: Anticoagulantes; Daiichi Sankyo: Antiplaquetários; Boeheringer. B - financiamento de pesquisas sob sua responsabilidade direta/pessoal (direcionado ao departamento ou instituição) provenientes da indústria farmacêutica, de órteses, próteses, equipamentos e implantes, brasileiras ou estrangeiras: - Astrazeneca: Antiplaquetários, ISGLT2; Daiichi Sankyo: Antiplaquetários; Astra Zeneca: Insuficiência Cardíaca, Antiplaquetários. C - Financiamento de pesquisa (pessoal), cujas receitas tenham sido provenientes da indústria farmacêutica, de órteses, próteses, equipamentos e implantes, brasileiras ou estrangeiras: - Boerinhger: Insuficiência Cardíaca; AstraZeneca: Dapaglifozina. Outros relacionamentos Financiamento de atividades de educação médica continuada, incluindo viagens, hospedagens e inscrições para congressos e cursos, provenientes da indústria farmacêutica, de órteses, próteses, equipamentos e implantes, brasileiras ou estrangeiras: - Bayer: Anticoagulates.
Felipe Gallego Lima	Declaração financeira A - Pagamento de qualquer espécie e desde que economicamente apreciáveis, feitos a (i) você, (ii) ao seu cônjuge/companheiro ou a qualquer outro membro que resida com você, (iii) a qualquer pessoa jurídica em que qualquer destes seja controlador, sócio, acionista ou participante, de forma direta ou indireta, recebimento por palestras, aulas, atuação como proctor de treinamentos, remunerações, honorários pagos por participações em conselhos consultivos, de investigadores, ou outros comitês, etc. Provenientes da indústria farmacêutica, de órteses, próteses, equipamentos e implantes, brasileiras ou estrangeiras: - Sanofi: Clexane; Servier: Ticagrelor. Outros relacionamentos Financiamento de atividades de educação médica continuada, incluindo viagens, hospedagens e inscrições para congressos e cursos, provenientes da indústria farmacêutica, de órteses, próteses, equipamentos e implantes, brasileiras ou estrangeiras: - Sanofi: Enoxaparina; Servier: Ticagrelor.
Francisco das Chagas Monteiro Júnior	Nada a ser declarado
Gilson Soares Feitosa Filho	Declaração financeira A - Pagamento de qualquer espécie e desde que economicamente apreciáveis, feitos a (i) você, (ii) ao seu cônjuge/companheiro ou a qualquer outro membro que resida com você, (iii) a qualquer pessoa jurídica em que qualquer destes seja controlador, sócio, acionista ou participante, de forma direta ou indireta, recebimento por palestras, aulas, atuação como proctor de treinamentos, remunerações, honorários pagos por participações em conselhos consultivos, de investigadores, ou outros comitês, etc. Provenientes da indústria farmacêutica, de órteses, próteses, equipamentos e implantes, brasileiras ou estrangeiras: - Bayer: Estudo COMPASS.
Harry Correa Filho	Declaração financeira A - Pagamento de qualquer espécie e desde que economicamente apreciáveis, feitos a (i) você, (ii) ao seu cônjuge/companheiro ou a qualquer outro membro que resida com você, (iii) a qualquer pessoa jurídica em que qualquer destes seja controlador, sócio, acionista ou participante, de forma direta ou indireta, recebimento por palestras, aulas, atuação como proctor de treinamentos, remunerações, honorários pagos por participações em conselhos consultivos, de investigadores, ou outros comitês, etc. Provenientes da indústria farmacêutica, de órteses, próteses, equipamentos e implantes, brasileiras ou estrangeiras: - Bayer: Anticoagulação.
Henrique Patrus Mundim Pena	Declaração financeira A - Pagamento de qualquer espécie e desde que economicamente apreciáveis, feitos a (i) você, (ii) ao seu cônjuge/companheiro ou a qualquer outro membro que resida com você, (iii) a qualquer pessoa jurídica em que qualquer destes seja controlador, sócio, acionista ou participante, de forma direta ou indireta, recebimento por palestras, aulas, atuação como proctor de treinamentos, remunerações, honorários pagos por participações em conselhos consultivos, de investigadores, ou outros comitês, etc. Provenientes da indústria farmacêutica, de órteses, próteses, equipamentos e implantes, brasileiras ou estrangeiras: - Lilly: Diabetes e Risco Cardiovascular; Daiichi Sankyo: Antiagregante Plaquetário; Pfizer e Bayer: Anticoagulante Oral. Outros relacionamentos Financiamento de atividades de educação médica continuada, incluindo viagens, hospedagens e inscrições para congressos e cursos, provenientes da indústria farmacêutica, de órteses, próteses, equipamentos e implantes, brasileiras ou estrangeiras: - Pfizer e Bayer: Anticoagulante Oral; Daiichi Sankyo: Antiagregante Plaquetário.
Ibraim Masciarelli Francisco Pinto	Declaração financeira A - Pagamento de qualquer espécie e desde que economicamente apreciáveis, feitos a (i) você, (ii) ao seu cônjuge/companheiro ou a qualquer outro membro que resida com você, (iii) a qualquer pessoa jurídica em que qualquer destes seja controlador, sócio, acionista ou participante, de forma direta ou indireta, recebimento por palestras, aulas, atuação como proctor de treinamentos, remunerações, honorários pagos por participações em conselhos consultivos, de investigadores, ou outros comitês, etc. Provenientes da indústria farmacêutica, de órteses, próteses, equipamentos e implantes, brasileiras ou estrangeiras: - Novo Nordisk: Diabetes.
João Luiz de Alencar Araripe Falcão	Nada a ser declarado
João Luiz Petriz	Declaração financeira A - Pagamento de qualquer espécie e desde que economicamente apreciáveis, feitos a (i) você, (ii) ao seu cônjuge/companheiro ou a qualquer outro membro que resida com você, (iii) a qualquer pessoa jurídica em que qualquer destes seja controlador, sócio, acionista ou participante, de forma direta ou indireta, recebimento por palestras, aulas, atuação como proctor de treinamentos, remunerações, honorários pagos por participações em conselhos consultivos, de investigadores, ou outros comitês, etc. Provenientes da indústria farmacêutica, de órteses, próteses, equipamentos e implantes, brasileiras ou estrangeiras: - Daiichi Sankyo e Servier: Cardiologia.
Joberto Pinheiro Sena	Declaração financeira A - Pagamento de qualquer espécie e desde que economicamente apreciáveis, feitos a (i) você, (ii) ao seu cônjuge/companheiro ou a qualquer outro membro que resida com você, (iii) a qualquer pessoa jurídica em que qualquer destes seja controlador, sócio, acionista ou participante, de forma direta ou indireta, recebimento por palestras, aulas, atuação como proctor de treinamentos, remunerações, honorários pagos por participações em conselhos consultivos, de investigadores, ou outros comitês, etc. Provenientes da indústria farmacêutica, de órteses, próteses, equipamentos e implantes, brasileiras ou estrangeiras: - Servier: Palestras sobre SCA; Bayer: Aulas em manejo de anticoagulantes e antitrombóticos; Daiichi Sankyo: Manejo de antiplaquetários em SCA.
José A. Marin-Neto	Nada a ser declarado
José Carlos Nicolau	Declaração financeira A - Pagamento de qualquer espécie e desde que economicamente apreciáveis, feitos a (i) você, (ii) ao seu cônjuge / companheiro ou a qualquer outro membro que resida com você, (iii) a qualquer pessoa jurídica em que qualquer destes seja controlador, sócio, acionista ou participante, de forma direta ou indireta, recebimento por palestras, aulas, atuação como proctor de treinamentos, remunerações, honorários pagos por participações em conselhos consultivos, de investigadores, ou outros comitês, etc. Provenientes da indústria farmacêutica, de órteses, próteses, equipamentos e implantes, brasileiras ou estrangeiras: - Amgen: Hipolipemiante; Bayer: Anticoagulante; Daiichi Sankyo: Antiplaquetário, anticoagulante; Novartis: Inibidor SRA, hipolipemiante; Sanofi: Anticoagulante, hipolipemiante, antiplaquetário; Servier: antiplaquetário. B - financiamento de pesquisas sob sua responsabilidade direta/pessoal (direcionado ao departamento ou instituição) provenientes da indústria farmacêutica, de órteses, próteses, equipamentos e implantes, brasileiras ou estrangeiras: - Astrazeneca: Antiplaquetário, hipoglicemiante; Bayer: Anticoagulante; Esperion: Hipolipemante; CSL Behring: hipolipemiante; Dalcor: Aumento HDL; Jansen: Anticoagulante; Novartis: Inibidor SRA; Novo Nordisk: Hipoglicemiante; Sanofi: Anticoagulate, hipolipemiante; Vifor: Deficiência de Ferro. C - Financiamento de pesquisa (pessoal), cujas receitas tenham sido provenientes da indústria farmacêutica, de órteses, próteses, equipamentos e implantes, brasileiras ou estrangeiras: - Roche: Kits de laboratório para avaliação de agregabilidade plaquetária. Outros relacionamentos Financiamento de atividades de educação médica continuada, incluindo viagens, hospedagens e inscrições para congressos e cursos, provenientes da indústria farmacêutica, de órteses, próteses, equipamentos e implantes, brasileiras ou estrangeiras: - Sanofi: Anticoagulante, antiplaquetário, hipolipemiante; Bayer: Anticoagulante; Servier: antiplaquetário.
José Maria Peixoto	Nada a ser declarado
Juliana Ascenção de Souza	Nada a ser declarado
Leonardo Sara da Silva	Nada a ser declarado
Leopoldo Soares Piegas	Nada a ser declarado
Lilia Nigro Maia	Nada a ser declarado
Louis Nakayama Ohe	Declaração financeira A - Pagamento de qualquer espécie e desde que economicamente apreciáveis, feitos a (i) você, (ii) ao seu cônjuge / companheiro ou a qualquer outro membro que resida com você, (iii) a qualquer pessoa jurídica em que qualquer destes seja controlador, sócio, acionista ou participante, de forma direta ou indireta, recebimento por palestras, aulas, atuação como proctor de treinamentos, remunerações, honorários pagos por participações em conselhos consultivos, de investigadores, ou outros comitês, etc. Provenientes da indústria farmacêutica, de órteses, próteses, equipamentos e implantes, brasileiras ou estrangeiras: - Daiichi Sankyo: Edoxabana. Outros relacionamentos Financiamento de atividades de educação médica continuada, incluindo viagens, hospedagens e inscrições para congressos e cursos, provenientes da indústria farmacêutica, de órteses, próteses, equipamentos e implantes, brasileiras ou estrangeiras: Outros relacionamentos - Abbott e Meryl: Hemodinâmica. Outros relacionamentos Participação em comitês de compras de materiais ou fármacos em instituições de saúde ou funções assemelhadas: - Comitê para compra de aparelhos para unidades de Covid-19.
Luciano Moreira Baracioli	Declaração financeira A - Pagamento de qualquer espécie e desde que economicamente apreciáveis, feitos a (i) você, (ii) ao seu cônjuge / companheiro ou a qualquer outro membro que resida com você, (iii) a qualquer pessoa jurídica em que qualquer destes seja controlador, sócio, acionista ou participante, de forma direta ou indireta, recebimento por palestras, aulas, atuação como proctor de treinamentos, remunerações, honorários pagos por participações em conselhos consultivos, de investigadores, ou outros comitês, etc. Provenientes da indústria farmacêutica, de órteses, próteses, equipamentos e implantes, brasileiras ou estrangeiras: - Sandoz: Clopidogrel; Bayer: Rivaroxabana. Outros relacionamentos Financiamento de atividades de educação médica continuada, incluindo viagens, hospedagens e inscrições para congressos e cursos, provenientes da indústria farmacêutica, de órteses, próteses, equipamentos e implantes, brasileiras ou estrangeiras: - Bayer: Anticoagulação.
Luís Alberto de Oliveira Dallan	Outros relacionamentos Participação em comitês de compras de materiais ou fármacos em instituições de saúde ou funções assemelhadas: - Pregão.
Luis Augusto Palma Dallan	Nada a ser declarado
Luiz Alberto Piva e Mattos	Nada a ser declarado
Luiz Bezerra Neto	Declaração financeira A - Pagamento de qualquer espécie e desde que economicamente apreciáveis, feitos a (i) você, (ii) ao seu cônjuge / companheiro ou a qualquer outro membro que resida com você, (iii) a qualquer pessoa jurídica em que qualquer destes seja controlador, sócio, acionista ou participante, de forma direta ou indireta, recebimento por palestras, aulas, atuação como proctor de treinamentos, remunerações, honorários pagos por participações em conselhos consultivos, de investigadores, ou outros comitês, etc. Provenientes da indústria farmacêutica, de órteses, próteses, equipamentos e implantes, brasileiras ou estrangeiras: - Pfizer e Bayer: Aula anticoagulante; Libbs: Aula anti-hipertensivos. Outros relacionamentos Participação societária de qualquer natureza e qualquer valor economicamente apreciável de empresas na área de saúde, de ensino ou em empresas concorrentes ou fornecedoras da SBC: - Sócio da L&P Cardio, empresa de serviços médicos.
Luiz Carlos Bodanese	Nada a ser declarado
Luiz Eduardo Fonteles Ritt	Declaração financeira A - Pagamento de qualquer espécie e desde que economicamente apreciáveis, feitos a (i) você, (ii) ao seu cônjuge / companheiro ou a qualquer outro membro que resida com você, (iii) a qualquer pessoa jurídica em que qualquer destes seja controlador, sócio, acionista ou participante, de forma direta ou indireta, recebimento por palestras, aulas, atuação como proctor de treinamentos, remunerações, honorários pagos por participações em conselhos consultivos, de investigadores, ou outros comitês, etc. Provenientes da indústria farmacêutica, de órteses, próteses, equipamentos e implantes, brasileiras ou estrangeiras: - Pfizer: Amiloidose.
Manoel Fernandes Canesin	Nada a ser declarado
Marcelo Bueno da Silva Rivas	Nada a ser declarado
Marcelo Franken	Nada a ser declarado
Marcos José Gomes Magalhães	Nada a ser declarado
Múcio Tavares de Oliveira Júnior	Nada a ser declarado
Nivaldo Menezes Filgueiras Filho	Declaração financeira A - Pagamento de qualquer espécie e desde que economicamente apreciáveis, feitos a (i) você, (ii) ao seu cônjuge / companheiro ou a qualquer outro membro que resida com você, (iii) a qualquer pessoa jurídica em que qualquer destes seja controlador, sócio, acionista ou participante, de forma direta ou indireta, recebimento por palestras, aulas, atuação como proctor de treinamentos, remunerações, honorários pagos por participações em conselhos consultivos, de investigadores, ou outros comitês, etc. Provenientes da indústria farmacêutica, de órteses, próteses, equipamentos e implantes, brasileiras ou estrangeiras: - Pfizer: Eliquis, Apixabana; Boehringer: Pradaxa, Praxbind, Jardiance; Astrazenca: Forxiga; Novo Nordisk; Daiichi Sankyo; Novartis; Bayer. B - financiamento de pesquisas sob sua responsabilidade direta/pessoal (direcionado ao departamento ou instituição) provenientes da indústria farmacêutica, de órteses, próteses, equipamentos e implantes, brasileiras ou estrangeiras: - Astrazeneca: Dapaglifozina. Outros relacionamentos Financiamento de atividades de educação médica continuada, incluindo viagens, hospedagens e inscrições para congressos e cursos, provenientes da indústria farmacêutica, de órteses, próteses, equipamentos e implantes, brasileiras ou estrangeiras: - Pfizer: Eliquis; Daiichi Sankyo: Effient; Bayer: Xarelto.
Oscar Pereira Dutra	Declaração financeira A - Pagamento de qualquer espécie e desde que economicamente apreciáveis, feitos a (i) você, (ii) ao seu cônjuge / companheiro ou a qualquer outro membro que resida com você, (iii) a qualquer pessoa jurídica em que qualquer destes seja controlador, sócio, acionista ou participante, de forma direta ou indireta, recebimento por palestras, aulas, atuação como proctor de treinamentos, remunerações, honorários pagos por participações em conselhos consultivos, de investigadores, ou outros comitês, etc. Provenientes da indústria farmacêutica, de órteses, próteses, equipamentos e implantes, brasileiras ou estrangeiras: - Astrazeneca: Antiglicêmicos; Daiichi Sankyo: Anticoagulantes; Sandoz: Anti-hipertensivos. B - financiamento de pesquisas sob sua responsabilidade direta/pessoal (direcionado ao departamento ou instituição) provenientes da indústria farmacêutica, de órteses, próteses, equipamentos e implantes, brasileiras ou estrangeiras: - Boheringer: Antiglicêmicos; Novartis: Insuficiência Cardíaca; Sanofi: Antilipêmicos. C - Financiamento de pesquisa (pessoal), cujas receitas tenham sido provenientes da indústria farmacêutica, de órteses, próteses, equipamentos e implantes, brasileiras ou estrangeiras: - Sanofi e EMS: Anticoagulantes; Amgen: Antilipêmicos. Outros relacionamentos Financiamento de atividades de educação médica continuada, incluindo viagens, hospedagens e inscrições para congressos e cursos, provenientes da indústria farmacêutica, de órteses, próteses, equipamentos e implantes, brasileiras ou estrangeiras: - Bayer: Anticoagulantes.
Otávio Rizzi Coelho	Declaração financeira B - financiamento de pesquisas sob sua responsabilidade direta/pessoal (direcionado ao departamento ou instituição) provenientes da indústria farmacêutica, de órteses, próteses, equipamentos e implantes, brasileiras ou estrangeiras: - Boheringer: Empaglifozina. Outros relacionamentos Financiamento de atividades de educação médica continuada, incluindo viagens, hospedagens e inscrições para congressos e cursos, provenientes da indústria farmacêutica, de órteses, próteses, equipamentos e implantes, brasileiras ou estrangeiras: - Daiichi Sankyo: Prasugrel; Bayer: Rivaroxabana; Astrazeneca: Dapaglifozina.
Paulo Ernesto Leães	Outros relacionamentos Financiamento de atividades de educação médica continuada, incluindo viagens, hospedagens e inscrições para congressos e cursos, provenientes da indústria farmacêutica, de órteses, próteses, equipamentos e implantes, brasileiras ou estrangeiras: - Boehringer: Anticoagulantes Participação em comitês de compras de materiais ou fármacos em instituições de saúde ou funções assemelhadas: Comitê de Padronização da Santa Casa de Porto Alegre
Paulo Roberto Ferreira Rossi	Declaração financeira A - Pagamento de qualquer espécie e desde que economicamente apreciáveis, feitos a (i) você, (ii) ao seu cônjuge / companheiro ou a qualquer outro membro que resida com você, (iii) a qualquer pessoa jurídica em que qualquer destes seja controlador, sócio, acionista ou participante, de forma direta ou indireta, recebimento por palestras, aulas, atuação como proctor de treinamentos, remunerações, honorários pagos por participações em conselhos consultivos, de investigadores, ou outros comitês, etc. Provenientes da indústria farmacêutica, de órteses, próteses, equipamentos e implantes, brasileiras ou estrangeiras: - Novartis: Sacubitril, Valsartan.
Paulo Rogério Soares	Declaração financeira A - Pagamento de qualquer espécie e desde que economicamente apreciáveis, feitos a (i) você, (ii) ao seu cônjuge / companheiro ou a qualquer outro membro que resida com você, (iii) a qualquer pessoa jurídica em que qualquer destes seja controlador, sócio, acionista ou participante, de forma direta ou indireta, recebimento por palestras, aulas, atuação como proctor de treinamentos, remunerações, honorários pagos por participações em conselhos consultivos, de investigadores, ou outros comitês, etc. Provenientes da indústria farmacêutica, de órteses, próteses, equipamentos e implantes, brasileiras ou estrangeiras: - Astrazeneca: Dapagliflozina; Servier: Ticagrelor; Bayer: Rivaroxabana.
Pedro Alves Lemos Neto	Nada a ser declarado
Pedro Silvio Farsky	Declaração financeira A - Pagamento de qualquer espécie e desde que economicamente apreciáveis, feitos a (i) você, (ii) ao seu cônjuge / companheiro ou a qualquer outro membro que resida com você, (iii) a qualquer pessoa jurídica em que qualquer destes seja controlador, sócio, acionista ou participante, de forma direta ou indireta, recebimento por palestras, aulas, atuação como proctor de treinamentos, remunerações, honorários pagos por participações em conselhos consultivos, de investigadores, ou outros comitês, etc. Provenientes da indústria farmacêutica, de órteses, próteses, equipamentos e implantes, brasileiras ou estrangeiras: - Aliança Boehringer Ingelheim/Lilly: I -SGLT2.
Rafael Rebêlo C. Cavalcanti	Nada a ser declarado
Remo Holanda de Mendonça Furtado	Declaração financeira A - Pagamento de qualquer espécie e desde que economicamente apreciáveis, feitos a (i) você, (ii) ao seu cônjuge / companheiro ou a qualquer outro membro que resida com você, (iii) a qualquer pessoa jurídica em que qualquer destes seja controlador, sócio, acionista ou participante, de forma direta ou indireta, recebimento por palestras, aulas, atuação como proctor de treinamentos, remunerações, honorários pagos por participações em conselhos consultivos, de investigadores, ou outros comitês, etc. Provenientes da indústria farmacêutica, de órteses, próteses, equipamentos e implantes, brasileiras ou estrangeiras: - Astra Zeneca: Dapagliflozina (Diabetes, Insuficiência Cardíaca); Servier: Ticagrelor; Bayer: Rivaroxabana. B - financiamento de pesquisas sob sua responsabilidade direta/pessoal (direcionado ao departamento ou instituição) provenientes da indústria farmacêutica, de órteses, próteses, equipamentos e implantes, brasileiras ou estrangeiras: - Bayer: Rivaroxabana (Covid); Astra Zeneca: Dapagliflozina (Covid), Dapagliflozina (IC); Pfizer: Tofacitinib (Covid), Apixabana (FA); EMS, Aché, Servier, Health Canada, GSK, Servier.
Renato Abdala Karam Kalil	Nada a ser declarado
Renato Delascio Lopes	Declaração financeira A - Pagamento de qualquer espécie e desde que economicamente apreciáveis, feitos a (i) você, (ii) ao seu cônjuge / companheiro ou a qualquer outro membro que resida com você, (iii) a qualquer pessoa jurídica em que qualquer destes seja controlador, sócio, acionista ou participante, de forma direta ou indireta, recebimento por palestras, aulas, atuação como proctor de treinamentos, remunerações, honorários pagos por participações em conselhos consultivos, de investigadores, ou outros comitês, etc. Provenientes da indústria farmacêutica, de órteses, próteses, equipamentos e implantes, brasileiras ou estrangeiras: - Bayer: Anticoagulante; Boehringer Ingleheim: Anticoagulação e Diabetes; Pfizer: Anticoagulação; Bristol-Myers Squibb; Daiichi Sankyo; Glaxo Smith Kline; Medtronic; Merck; Portola; Sanofi.B - financiamento de pesquisas sob sua responsabilidade direta/pessoal (direcionado ao departamento ou instituição) provenientes da indústria farmacêutica, de órteses, próteses, equipamentos e implantes, brasileiras ou estrangeiras: - Pfizer: Apixaban; Bayer: Rivaroxaban; Novartis: Sacubitril, Valsartan. Outros relacionamentos Financiamento de atividades de educação médica continuada, incluindo viagens, hospedagens e inscrições para congressos e cursos, provenientes da indústria farmacêutica, de órteses, próteses, equipamentos e implantes, brasileiras ou estrangeiras: - Bayer: Rivaroxabana; Pfizer: Apixabana.
Renato Jorge Alves	Declaração financeira A - Pagamento de qualquer espécie e desde que economicamente apreciáveis, feitos a (i) você, (ii) ao seu cônjuge / companheiro ou a qualquer outro membro que resida com você, (iii) a qualquer pessoa jurídica em que qualquer destes seja controlador, sócio, acionista ou participante, de forma direta ou indireta, recebimento por palestras, aulas, atuação como proctor de treinamentos, remunerações, honorários pagos por participações em conselhos consultivos, de investigadores, ou outros comitês, etc. Provenientes da indústria farmacêutica, de órteses, próteses, equipamentos e implantes, brasileiras ou estrangeiras: - Amgen: Evolocumabe; PTC: Volanesorsen; Pfizer: Apixaban.
Roberto Esporcatte	Declaração financeira A - Pagamento de qualquer espécie e desde que economicamente apreciáveis, feitos a (i) você, (ii) ao seu cônjuge / companheiro ou a qualquer outro membro que resida com você, (iii) a qualquer pessoa jurídica em que qualquer destes seja controlador, sócio, acionista ou participante, de forma direta ou indireta, recebimento por palestras, aulas, atuação como proctor de treinamentos, remunerações, honorários pagos por participações em conselhos consultivos, de investigadores, ou outros comitês, etc. Provenientes da indústria farmacêutica, de órteses, próteses, equipamentos e implantes, brasileiras ou estrangeiras: - Bayer: Anticoagulação.
Roberto Luiz Marino	Declaração financeira B - financiamento de pesquisas sob sua responsabilidade direta/pessoal (direcionado ao departamento ou instituição) provenientes da indústria farmacêutica, de órteses, próteses, equipamentos e implantes, brasileiras ou estrangeiras: - CSL Behring: Infarto do Miocárdio.
Roberto Rocha Corrêa Veiga Giraldez	Nada a ser declarado
Romeu Sérgio Meneghelo	Nada a ser declarado
Ronaldo de Souza Leão Lima	Outros relacionamentos Participação societária de qualquer natureza e qualquer valor economicamente apreciável de empresas na área de saúde, de ensino ou em empresas concorrentes ou fornecedoras da SBC: - Fonte Imagem: sócio minoritário.
Rui Fernando Ramos	Declaração financeira A - Pagamento de qualquer espécie e desde que economicamente apreciáveis, feitos a (i) você, (ii) ao seu cônjuge / companheiro ou a qualquer outro membro que resida com você, (iii) a qualquer pessoa jurídica em que qualquer destes seja controlador, sócio, acionista ou participante, de forma direta ou indireta, recebimento por palestras, aulas, atuação como proctor de treinamentos, remunerações, honorários pagos por participações em conselhos consultivos, de investigadores, ou outros comitês, etc. Provenientes da indústria farmacêutica, de órteses, próteses, equipamentos e implantes, brasileiras ou estrangeiras: - Aspen: Fondaparinux. Financiamento de atividades de educação médica continuada, incluindo viagens, hospedagens e inscrições para congressos e cursos, provenientes da indústria farmacêutica, de órteses, próteses, equipamentos e implantes, brasileiras ou estrangeiras: - Aspen: Fondaparinux.
Sandra Nivea dos Reis Saraiva Falcão	Nada a ser declarado
Talia Falcão Dalçóquio	Declaração financeira B - financiamento de pesquisas sob sua responsabilidade direta/pessoal (direcionado ao departamento ou instituição) provenientes da indústria farmacêutica, de órteses, próteses, equipamentos e implantes, brasileiras ou estrangeiras: - Amgen, DalCor Pharma UK Ltd, CSL Behring LLC: Farmacêutica.
Viviana de Mello Guzzo Lemke	Nada a ser declarado
Walmor Lemke	Nada a ser declarado
William Azem Chalela	Nada a ser declarado
Wilson Mathias Júnior	Nada a ser declarado

**Table:** Diretrizes da SBC sobre Angina Instável e IAM sem supradesnível de ST – Atualização 2021

Coordenador Geral: José C. Nicolau; Assistente da Coordenação: Remo H.M. Furtado; Supervisão da versão em Inglês: Renato D. Lopes; Secretaria: Daniele Gullo		
Parte 1 - Avaliação e Condutas na Emergência	Parte 2 – Condutas Durante a Hospitalização	Parte 3: Recomendações na Alta e Cuidados Pós-alta Hospitalar
**Coordenadores:**	**Coordenadores:**	**Coordenadores:**
Ari Timerman	Leopoldo S. Piegas	Gilson Feitosa Filho
João Luiz Petriz	José A. Marin-Netto	Luiz Bezerra Neto
Bruno Ferraz	Dalton Précoma	Eduardo Lapa
	Walmor Lemke	
**Revisores Parte 1**	**Revisores Parte 2**	**Revisores Parte 3**
Brivaldo Markman Filho	Andre Franci	Alexandre de Matos Soeiro
Bruno Mendonça Baccaro	Edson Stefanini	Alexandre Jorge de Andrade Negri
Carlos Eduardo Rochitte	Euler Roberto Fernandes Manenti	Carlos Eduardo Lucena Montenegro
Leonardo Sara	Felipe Galego Lima	Cláudio Marcelo Bittencourt das Virgens
Carlos Jose Dornas Gonçalves Barbosa	Harry Correa Filho	Francisco das Chagas Monteiro Júnior
Louis Nakayama Ohe	Ibraim Masciarelli Francisco Pinto	Henrique Patrus Mundim Pena
Luiz Carlos Bodanese	Lilia Nigro Maia	João Luiz de Alencar Araripe Falcão
Marcelo Bueno da Silva Rivas	Luciano Moreira Baracioli	Joberto Pinheiro Sena
Paulo Roberto Ferreira Rossi	Luís Alberto de Oliveira Dallan	José Maria Peixoto
Paulo Rogério Soares	Luis Augusto P. Dallan	Juliana Ascenção de Souza
Pedro Alves Lemos Neto	Luiz Alberto Piva e Mattos	Luiz Eduardo Fonteles Ritt
Renato Delascio Lopes	Marcelo Franken	Manoel Fernandes Canesin
Roberto Esporcatte	Oscar Pereira Dutra	Marcos José Gomes Magalhães
Roberto Luiz Marino	Otávio Rizzi Coelho	Múcio Tavares de Oliveira Júnior
Roberto Rocha Corrêa Veiga Giraldez	Paulo Ernesto Leães	Nivaldo Menezes Filgueiras Filho
Rui Fernando Ramos	Renato Kalil	Pedro Silvio Farsky
Talia Falcão Dalçóquio	Romeu Sérgio Meneghelo	Rafael Rebêlo C. Cavalcanti
William Azem Chalela	Ronaldo de Souza Leão Lima	Renato Jorge Alves
	Vivivana Lemke	Sandra Nives dos Reis Saraiva Falcão
	Wilson Mathias Júnior	

## Definições das Recomendações e Evidências

### Recomendações

**Classe I:** Condições para as quais há evidências conclusivas, ou, na sua falta, consenso geral de que o procedimento é seguro e útil/eficaz.
**Classe II:** Condições para as quais há evidências conflitantes e/ou divergência de opinião sobre segurança e utilidade/eficácia do procedimento.
**Classe IIa:** Peso ou evidência/opinião a favor do procedimento. A maioria aprova.
**Classe IIb:** Segurança e utilidade/eficácia menos bem estabelecida, não havendo predomínio de opiniões a favor.
**Classe III:** Condições para as quais há evidências e/ou consenso de que o procedimento não é útil/eficaz e, em alguns casos, pode ser prejudicial.

### Evidências

**Nível A:** Dados obtidos a partir de múltiplos estudos randomizados de bom porte, concordantes e/ou de metanálise robusta de estudos clínicos randomizados.
**Nível B:** Dados obtidos a partir de metanálise menos robusta, a partir de um único estudo randomizado ou de estudos não randomizados (observacionais).
**Nível C:** Dados obtidos de opiniões consensuais de especialistas.

## Mudança de recomendações

**Table:** 

Mudança de recomendações - Marcadores Bioquímicos					
Diretriz 2014			Diretriz 2020		
Marcadores bioquímicos de necrose miocárdica devem ser mensurados em todos os pacientes com suspeita de síndromes coronarianas agudas sem supradesnível do segmento ST (SCASSST). Os marcadores devem ser medidos na admissão e repetidos pelo menos uma vez, 6 a 9h após (preferencialmente 9 a 12h após o início dos sintomas), caso a primeira dosagem seja normal ou discretamente elevada.	I	C	Biomarcadores bioquímicos de necrose miocárdica devem ser mensurados em todos os pacientes com suspeita de SCASSST. Quando troponina ultrassensível estiver disponível, a dosagem sérica deve ser realizada na admissão e idealmente reavaliada em 1h ou até 2h. Caso indisponível, a troponina convencional deve ser coletada na admissão e repetida pelo menos uma vez, 3 a 6h após, caso a primeira dosagem seja normal ou discretamente elevada.	I	B
Creatinoquinase MB (CK-MB) massa e troponinas são os marcadores bioquímicos de escolha.	I	A	Dosagens CK-MB massa podem ser utilizadas se dosagens de troponina não estiverem disponíveis.	IIb	B
Para pacientes que chegam precocemente à emergência (antes de 6h do início dos sintomas), mioglobina e troponina ultrassensível podem ser consideradas em adição a um marcador mais tardio (CK-MB ou troponina).	IIb	B	As troponinas são os biomarcadores de escolha em pacientes com suspeita de IAM.	I	A
			Utilização da mioglobina para detecção de necrose miocárdica em pacientes com suspeita de SCASSST.	III	

**Table:** 

Mudança de recomendações - Teste ergométrico					
Diretriz 2014			Diretriz 2020		
Pacientes de risco baixo (clínica e eletrocardiograma [ECG]) e com marcadores bioquímicos normais devem ser encaminhados para teste ergométrico após 9h, idealmente até 12h, em regime ambulatorial.	I	B	Pacientes de risco baixo (clínica e ECG) e com biomarcadores normais devem ser encaminhados para teste ergométrico após 9 a 12h em observação. Dentro das rotas da unidade de dor torácica (UDT), estes exames podem ser feitos como critério de alta.	I	B

**Table:** 

Mudança de recomendações - Cardiologia nuclear					
Diretriz 2014			Diretriz 2020		
Cintilografia miocárdica de perfusão em estresse e repouso é uma alternativa ao teste ergométrico nos pacientes com impossibilidade para o mesmo.	I	C	Cintilografia miocárdica de estresse (físico ou farmacológico) pode ser utilizada como método de estratificação em pacientes sem dor torácica recorrente, sem evidências eletrocardiográficas de isquemia e/ou elevação de troponina.	I	B
Pacientes em vigência de dor torácica podem ser avaliados pela cintilografia miocárdica de perfusão em repouso para determinar a origem isquêmica ou não da dor	IIa	A	Pacientes em vigência de dor torácica e eletrocardiograma sem alterações isquêmicas podem ser avaliados pela cintilografia miocárdica de perfusão em repouso para determinar a origem isquêmica ou não da dor.	IIa	A

**Table:** 

Mudança de Recomendações - Analgesia e sedação					
Diretriz 2014			Diretriz 2020		
Administrar sulfato de morfina a pacientes de risco intermediário e alto.	I	C	Administrar sulfato de morfina em pacientes que mantêm dor contínua, apesar de terapia anti-isquêmica otimizada	IIb	B
Administrar benzodiazepínicos a pacientes de alto risco.	I	C	Administrar benzodiazepínicos em pacientes com sinais e	IIb	C
Administrar benzodiazepínicos a pacientes de risco intermediário.	IIa	C	sintomas de ansiedade persistente.		

**Table:** 

Mudança de recomendações - Nitratos					
Diretriz 2014			Diretriz 2020		
Uso de nitrato em pacientes com risco intermediário e alto.	I	C	Uso de nitrato sublingual para alívio da angina.	I	C
			Uso de nitrato endovenoso para controle de angina persistente, hipertensão arterial ou sinais de congestão.	I	C

**Table:** 

Mudança de recomendações - Betabloqueadores					
Diretriz 2014			Diretriz 2020		
Administrar betabloqueadores por via oral (VO) a pacientes de risco intermediário e alto.	I	B	Administrar betabloqueadores VO nas primeiras 24h em pacientes sem contraindicações (sinais de insuficiência cardíaca, sinais de baixo débito, risco aumentado de choque cardiogênico ou outras contraindicações ao betabloqueador).	IIa	B

**Table:** 

Mudança de recomendações - Uso de antiplaquetários na sala de emergência					
Diretriz 2014			Diretriz 2020		
Tienopiridínicos em pacientes com contraindicação ao ácido acetilsalicílico (AAS).	I	B	Em pacientes alérgicos a AAS, está indicada monoterapia inicial com inibidor P2Y_12_ (uso preferencial de ticagrelor ou prasugrel).	I	C

**Table:** 

Mudança de recomendações - Antagonistas dos receptores da glicoproteína Ilb/IIIa					
Diretriz 2014			Diretriz 2020		
Adição de inibidores da GP Ilb/IIIa em pacientes que apresentam recorrência de sintomas isquêmicos na vigência de dupla antiagregação plaquetária oral e anticoagulação.	IIa	C	Na estratégia conservadora, adição de tirofiban em pacientes que apresentam recorrência de sintomas isquêmicos na vigência de dupla antiagregação plaquetária oral e anticoagulação.	IIb	C

**Table:** 

Mudança de recomendações - Anticoagulantes					
Diretriz 2014			Diretriz 2020		
Uso de heparina não fracionada (HNF) em todos os pacientes.	I	A	Uso preferencial de HNF em pacientes com disfunção renal grave *(clearance* < 15mL/min) e obesos com peso > 150kg	IIa	B
Uso de heparinas de baixo peso molecular (HBPM) em todos os pacientes.	I	A	Uso de enoxaparina em pacientes sem disfunção renal grave *(clearance* < 15mL/min/1,73m^2^), até a revascularização, por 8 dias ou até a alta hospitalar, 1mg/kg 12/12h (0,75mg/kg, 12/12h, se ≥ 75 anos; 1mg/kg, 24/24h, se clearence de creatinina entre 15 e 30mL/min/1,73m2; máximo de 150mg por dose.	I	A

## Novas recomendações

**Table:** 

História e exame físico		
Pacientes com suspeita de SCASSST e dor persistente, dispneia, palpitações ou síncope devem ser encaminhados para serviços de emergência idealmente monitorados em ambulância.	I	C
Pacientes com suspeita de SCASSST com achados de menor gravidade, (i. e., sem dor persistente, dispneia, palpitações ou síncope) podem procurar por meios próprios o serviço de emergência mais próximo com capacidade de realizar ECG e dosagem de troponina.	IIb	C

**Table:** 

Eletrocardiograma		
O ECG deve ser repetido em caso de recorrência dos sintomas.	I	C
As derivações V3R-V4R, V7-V9 devem ser realizadas em pacientes que permanecem sintomáticos e apresentam ECG de 12 derivações não diagnóstico.	I	C

**Table:** 

Marcadores bioquímicos		
Na disponibilidade de troponina, nenhum outro marcador necessita ser solicitado para fins diagnósticos.	I	B

**Table:** 

Angiografia por tomografia computadorizada das artérias coronárias		
Investigação da dor torácica aguda pela técnica do descarte triplo *(triple rule-out).*	IIb	B

**Table:** 

Rotina diagnóstica e critérios de internação		
A triagem inicial deve ser realizada com base em história clínica, exame físico, ECG de 12 derivações em até 10min e troponina.	I	A
O escore HEART deve ser utilizado para estratificação de risco e auxílio na decisão de alta hospitalar.	I	B
Pacientes com escore HEART ≤ 3 associado à troponina negativa, ECG sem alteração isquêmica e ausência de antecedentes de doença arterial coronariana (DAC) podem ser liberados do serviço de emergência para reavaliação ambulatorial.	I	B
Triagem hospitalar realizada por enfermeiros habilitados visando ao reconhecimento precoce de pacientes sob maior risco.	IIa	B
Uso de escore EDACS e ADAPT para estratificação de risco clínico como opção ao escore HEART.	IIa	B
Utilização do escore HEART visando à liberação precoce em pacientes atendidos por ambulância.	IIb	C

**Table:** 

Estratégia invasiva de urgência		
A estratégia invasiva urgente/imediata está indicada em pacientes com SCASSST com angina refratária e/ou instabilidade hemodinâmica e/ou elétrica (sem comorbidades graves ou contraindicações para esses procedimentos).	I	A

**Table:** 

Controle glicêmico		
É recomendável mensurar, na admissão, os níveis glicêmicos de todos os pacientes com suspeita de infarto agudo do miocárdio (IAM), assim como monitorar evolutivamente a glicemia dos pacientes diabéticos ou que apresentem hiperglicemia durante a internação.	I	C
Controle glicêmico com protocolos de utilização de insulina intermitente deve ser considerado em pacientes com níveis glicêmicos > 180mg / dL, com cautela, para evitar episódios de hipoglicemia.	IIa	C
Em pacientes com maior risco de hipoglicemia, tais como idosos, nefropatas e pacientes com efeito residual de hipoglicemiantes orais e/ou em jejum, o controle glicêmico deve ser ajustado para tolerar níveis glicêmicos um pouco mais altos e, assim, prevenir hipoglicemia.	IIa	C

**Table:** 

Betabloqueadores	
Administrar betabloqueador por via intravenosa (IV) em pacientes com fatores de risco para choque cardiogênico.	III

**Table:** 

Terapia antiplaquetária inicial		
Não realizar pré-tratamento com segundo antiplaquetário inibidor do receptor P2Y_12_ nos pacientes instáveis e/ou com risco elevado, indicados para estratégia invasiva de forma imediata, sendo sua utilização recomendada para sala de cateterismo quando anatomia coronariana conhecida e intervenção coronária percutânea (ICP) programada.	I	B
Não há indicação rotineira de se iniciar iP2Y_12_ como pré-tratamento em pacientes indicados para estratégia invasiva precoce (< 24h).	I	B
Clopidogrel (dose de ataque) independente da estratégia inicial (conservadora ou invasiva) em pacientes de muito alto risco para sangramento ou necessidade de anticoagulação oral a longo prazo.	IIa	C
Estratégia de pré-tratamento com prasugrel não é recomendada.	III	

**Table:** 

Anticoagulantes		
Uso de fondaparinux 2,5mg por via subcutânea (SC) 1 vez/dia por 8 dias ou até a alta hospitalar como alternativa à enoxaparina, especialmente no paciente de elevado risco hemorrágico.	I	B
Monitoramento do fator anti-Xa em pacientes com *clearance* entre 15 e 30mL/min e obesos com peso entre 100 e 150kg em uso de enoxaparina.	IIa	B
Uso preferencial de enoxaparina *versus* HNF em pacientes com *clearance ≥* 15mL/min/1,73m^2^), a não ser que cirurgia de revascularização miocárdica esteja planejada para as próximas 24h.	IIa	B
Uso preferencial de HNF na emergência ou sala de hemodinâmica em pacientes de muito alto risco, com proposta de cateterismo imediato (< 2h).	IIa	C
Uso de enoxaparina em pacientes com *clearance* de creatinina < 15mL/min e peso > 150kg.	III	
Uso de fondaparinux em pacientes com *clearance* de creatinina < 20mL/min.	III	

## Parte 1 – Avaliação e Condutas na Emergência

### 1. Introdução

A dor torácica aguda é uma das causas mais frequentes de atendimento nas unidades de emergência (UE), correspondendo a mais de 5% das visitas em UE e até 10% das visitas não relacionadas a traumatismos. A incidência de dor torácica varia entre 9 e 19 por 1.000 pessoas/ano atendidas em UE e pode representar até 40% das causas de internação hospitalar. A maioria desses pacientes recebe alta com diagnóstico de dor torácica não especificada ou causa não cardíaca; no entanto, cerca de 25% dos pacientes internados apresentam diagnóstico final de síndrome coronariana aguda (SCA).^1^^,^^2^

O subgrupo de pacientes que não apresentam supradesnível do segmento ST no eletrocardiograma (ECG) e contêm sintomas ou achados em exames complementares compatíveis com etiologia coronariana compõem as síndromes coronarianas agudas sem supradesnível do segmento ST (SCASSST), objeto dessa diretriz. A SCASSST pode causar morbidade significativa e mortalidade se não for tratada de forma imediata e adequada. Atraso no tratamento apropriado pode resultar em resultados adversos graves, salientando a necessidade de verificar a presença de SCA pela avaliação de dados clínicos e exames complementares.^3^

### 2. Definições

A dor torácica é o principal sintoma em um paciente com SCA. O ECG deve ser realizado e interpretado nos primeiros 10min do contato médico em pacientes suspeitos para SCA, e seus achados podem diferenciar o paciente em dois grupos:

- SCACSST: paciente com dor torácica aguda e supradesnivelamento persistente do segmento ST ou bloqueio de ramo esquerdo (BRE) novo ou presumivelmente novo, condição geralmente relacionada com oclusão coronariana e necessidade de reperfusão imediata.
- SCASSST: paciente com dor torácica aguda sem supradesnivelamento persistente do segmento ST, associado ou não a outras alterações de ECG que sugerem isquemia miocárdica de alguma natureza com amplo espectro de gravidade: elevação transitória do segmento ST, infradesnivelamento transitório ou persistente do seguimento ST, inversão de onda T, outras alterações inespecíficas da onda T (plana ou pseudonormalização) e até mesmo ECG normal. Neste grupo, estão os pacientes com angina instável (AI), ou seja, sem alterações de marcadores de necrose miocárdica, e aqueles com infarto agudo do miocárdio sem supradesnivelamento do segmento ST (IAMSSST), quando há elevação de marcadores de necrose miocárdica.

O diagnóstico de IAMSSST é confirmado quando da presença de isquemia na lesão miocárdica aguda, confirmada por elevação nos níveis de troponina. Com base em sua fisiopatologia e contexto clínico, o IAM é classificado em vários subtipos (Tabela 1.1 e 1.2). A elevação dos níveis de troponina pode ser secundária à isquemia miocárdica, mas também pode ocorrer em outras situações clínicas (Tabela 1.3).

**Tabela 1.1:** Lesão e infarto do miocárdio^1^

Definição de lesão miocárdica
O termo lesão miocárdica deve ser empregado em pacientes com valores de troponina cardíaca, em pelo menos uma dosagem, acima do percentil 99 do limite da normalidade. A lesão é considerada aguda se houver comportamento dinâmico com ascensão e/ou queda dos valores basais. Valores de troponina persistentemente elevados são considerados lesão miocárdica crônica.
**Definição de infarto agudo do miocárdio (IAM tipos 1, 2 e 3)**
A definição de IAM implica a presença de lesão miocárdica aguda em um contexto clínico de isquemia miocárdica:
• Sintomas sugestivos de isquemia miocárdica aguda
• Nova alteração isquêmica no ECG
• Nova onda Q patológica no ECG
• Exame de imagem evidenciando nova alteração de contratilidade ou perda de miocárdio viável consistente com etiologia isquêmica
• Identificação de trombo intracoronário por angiografia ou necrópsia (apenas para o tipo 1)

**Tabela 1.2:** Classificação do infarto agudo do miocárdio (IAM) de acordo com fatores desencadeantes

Classificação (tipos)	Descrição
1	IAM espontâneo relacionado com isquemia miocárdica secundária a evento coronariano como ruptura ou erosão de placa aterosclerótica coronariana
2	IAM secundário à isquemia por desequilíbrio de oferta / demanda de oxigênio pelo miocárdio, não relacionado diretamente à aterotrombose coronariana
3	Morte súbita na presença de sintomas sugestivos de isquemia acompanhada por novas alterações isquêmicas no ECG ou fibrilação ventricular e que ocorre antes de os biomarcadores serem coletados ou de sua elevação. Ou IAM confirmado por necrópsia
4a	IAM associado à intervenção coronariana percutânea ≤ 48h – definido pelo aumento de troponina maior que 5 vezes do percentil 99 do limite da normalidade ou 20% de níveis basais já aumentados, associado a um dos achados a seguir: Nova alteração isquêmica no ECG Nova onda Q patológica no ECG Exame de imagem evidenciando nova alteração de contratilidade ou perda de miocárdio viável de padrão consistente com isquemia miocárdica Achados angiográficos com complicações que levem à limitação do fluxo coronário (dissecção, oclusão de vaso epicárdico, perda de circulação colateral e embolização distal)
4b	IAM associado à trombose de *stent* documentada por angiografia ou necrópsia
4c	IAM relacionado à reestenose *intrastent* ou pós-angioplastia na ausência de outras lesões ou trombo intracoronário que o justifiquem
5	IAM associado à cirurgia de revascularização miocárdica ≤ 48h – definido pelo aumento maior que 10 vezes do percentil 99 do limite da normalidade ou 20% de níveis basais já aumentados, associado a um dos achados a seguir: Nova onda Q patológica no ECG Exame de imagem evidenciando nova alteração de contratilidade ou perda de miocárdio viável com padrão de etiologia isquêmica Achado angiográfico que evidencie oclusão de novo enxerto ou artéria coronária nativa

Fonte: adaptada de Thygesen K et al.^4^ ECG: eletrocardiograma.

**Tabela 1.3:** Causas de lesão miocárdica

Lesão miocárdica de etiologia isquêmica
• **Ruptura ou erosão de placa arterosclerótica com trombose**

• **Lesão miocárdica relacionada com isquemia miocárdica por desequilíbrio entre oferta/consumo de oxigênio**
Redução na perfusão miocárdica:
• Espasmo arterial coronariano
• Doença coronariana microvascular
• Embolismo coronariano
• Dissecção coronariana
• Bradiarritmia sustentada
• Hipotensão ou choque
• Falência respiratória
• Anemia grave
Aumento no consumo de oxigênio:
• Taquiarritmia sustentada
• Crise hipertensiva
**Lesão miocárdica por outras condições cardíacas não isquêmicas**
• Insuficiência cardíaca
• Miocardite
• Cardiomiopatias de qualquer tipo
• Síndrome de Takotsubo
• Procedimento de revascularização miocárdica
• Ablação miocárdica por cateter
• Desfibrilação ou cardioversão elétrica
• Contusão miocárdica
**Lesão miocárdica por condições sistêmicas**
• Sepse ou processo infeccioso ativo
• Doença renal crônica
• Acidente vascular cerebral (AVC) e hemorragia subaracnóidea
• Embolia pulmonar e hipertensão pulmonar
• Doença miocárdica infiltrativa (p. ex., amiloidose, sarcoidose)
• Agentes quimioterápicos
• Doente crítico
• Atividade física extrema

Situações em que ocorre elevação de marcadores de necrose miocárdica, na ausência de isquemia detectada por quadro clínico, ECG ou exames de imagem, devem ser definidas como lesão miocárdica aguda, e não IAM. Podem ser secundárias a causas cardíacas (como procedimentos cardiovasculares, miocardite, arritmias, insuficiência cardíaca descompensada) ou extracardíacas (como choque, anemia grave, sepse e hipóxia).^4^

A lesão miocárdica é frequentemente relacionada a condições clínicas de pior prognóstico. É preciso realizar a diferenciação entre causas isquêmicas ou não isquêmicas, a fim de evitar intervenções invasivas desnecessárias e direcionar condutas a outras etiologias possíveis (Tabela 1.3).

**Figura 1.1:**
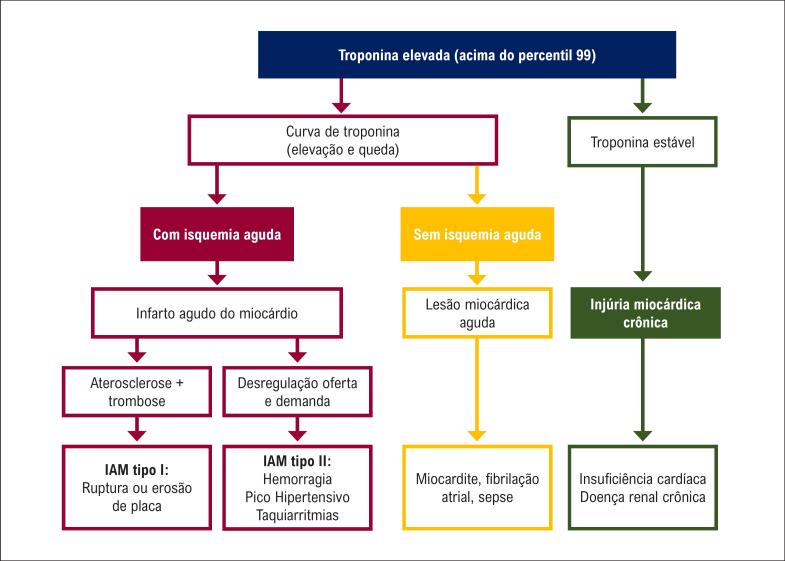
Algoritmo de interpretação da elevação de troponina. Curva de troponina significa elevação superior a 20%

A Figura 1.1 sumariza a interpretação da elevação de troponina frente aos cenários de lesão e isquemia coronariana.

#### 2.1. Conceito de MINOCA

Os casos de IAM sem a presença de doença arterial coronariana (DAC) obstrutiva são classificados como MINOCA (do inglês, *myocardial infarction with non-obstructive coronary arteries*). Aproximadamente dois terços dos pacientes com MINOCA têm apresentação clínica de IAMSSST.^5^

Conforme documento elaborado por grupo de trabalho organizado pela Sociedade Europeia de Cardiologia^6^ e a 4º Definição Universal de Infarto do Miocárdio,^4^ os critérios diagnósticos para MINOCA são: IAM, documentação angiográfica com ausência de DAC obstrutiva (ateromatose com estenose < 50% ou coronária normais) e nenhuma causa clinicamente evidente não coronariana que justifique a apresentação aguda.

Diferentes mecanismos fisiopatológicos podem causar MINOCA:

- Disfunções coronárias epicárdicas (p. ex., ruptura de placa aterosclerótica, ulceração, fissuração, erosão ou dissecção coronária).
- Desequilíbrio entre oferta e consumo de oxigênio (p. ex., espasmo coronariano e embolia coronariana).
- Disfunção endotelial coronariana (p. ex., doença microvascular).

O prognóstico da MINOCA é extremamente variável e depende do mecanismo subjacente e de fatores de risco associados, tais como idade e sexo feminino. Alguns estudos apontam para mortalidade hospitalar inferior ao IAM com coronariopatia obstrutiva e mortalidade em 1 ano semelhante ao IAM com obstrução univascular. No entanto, na análise dos pacientes com IAMSSST do estudo ACUITY, o subgrupo com MINOCA apresentou maior mortalidade em 1 ano (4,7% *vs.* 3,6%), ligada a um aumento de mortes não cardíacas.^7^^-^^9^

**Figura 1.2:**
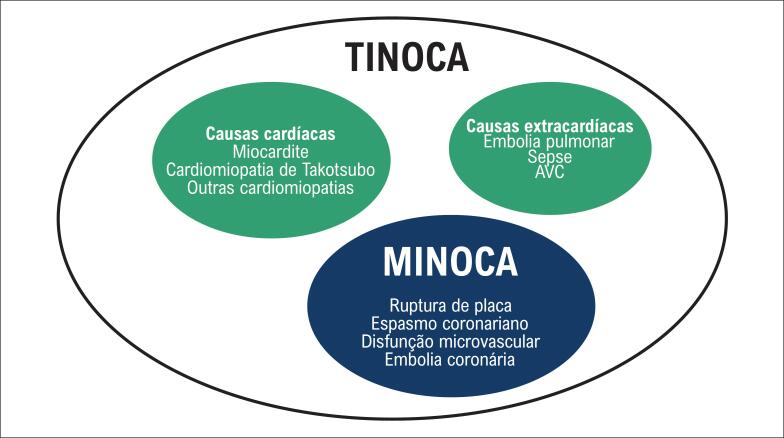
MINOCA e TINOCA: paradigma conceitual. Adaptada de Pasupathy et al., 2017.^10^

O grande grupo de pacientes que se apresentam com elevação de troponina na ausência de obstrução coronariana e manifestações clínicas de infarto é classificado como portador de TINOCA (do inglês, *troponin-positive nonobstructive coronary arteries*), contemplando aqueles com lesão de etiologia isquêmica (MINOCA) e as demais etiologias descritas de lesão miocárdica não isquêmica (p. ex., miocardite ou síndrome de Takotsubo; vide Figura 1.2).

#### 2.2. Angina Instável

A AI é definida como isquemia miocárdica na ausência de necrose miocárdica, ou seja, com biomarcadores negativos. Durante o manejo inicial da SCA, frequentemente, é difícil diferenciar AI do IAMSSST com base apenas em critérios clínicos (*i. e.*, antes que a dosagem de marcadores de necrose miocárdica esteja disponível), devendo ambas as entidades serem conduzidas de forma semelhante nessa fase. O aumento da sensibilidade da troponina diminuiu o percentual de pacientes diagnosticados como AI e aumentou o de pacientes com IAMSSST. Pacientes com AI têm prognósticos diferentes, podendo ter desde um risco relativamente baixo até um risco alto. As classificações de AI baseadas na apresentação clínica e em informações prognósticas facilitam a condução terapêutica, como será discutido no decorrer deste documento.^11^^-^^16^

### 3. Epidemiologia

O IAM é a principal causa de morte no Brasil e no mundo.^17^ Em 2017, segundo o DATASUS, 7,06% (92.657 pacientes) do total de óbitos foram causados por IAM. O IAM representou 10,2% das internações no Sistema Único de Saúde (SUS), sendo mais prevalente em pacientes com idade superior a 50 anos, em que representou 25% das internações).^18^

No registro BRACE, que avaliou as internações por SCA em 72 hospitais no Brasil, as SCASSST representaram 45,7% das internações, das quais cerca de 2/3 ocorreram por IAM e 1/3 por AI. O estudo revelou, no geral, uma taxa reduzida de uso de terapias que impactam o prognóstico de pacientes com SCA, com importantes diferenças regionais. Além disso, desenvolveu um escore de desempenho demonstrando que quanto maior a aderência a tratamentos comprovados, menor a mortalidade.^19^

### 4. Fisiopatologia

A principal característica fisiopatológica da SCA é a instabilização da placa aterosclerótica, envolvendo erosão ou ruptura e subsequente formação de trombo oclusivo ou suboclusivo. Tal limitação de fluxo, no entanto, pode ocorrer por outros mecanismos como vasospasmo, embolia ou dissecção coronariana. Outros fatores podem estar envolvidos na fisiopatologia da SCA por alterar a oferta e/ou o consumo de oxigênio miocárdico, tais como anemia, hipertensão, taquicardia, cardiomiopatia hipertrófica, estenose aórtica, entre outras.^20^

Em animais, observou-se que a isquemia progride do subendocárdio ao subepicárdio. O tempo de progressão pode prolongar-se quando há a presença de irrigação colateral eficiente, redução no consumo de oxigênio pelo miocárdio e intermitência no fluxo (gerando pré-condicionamento isquêmico). A função ventricular sofre alterações decorrentes da progressão da isquemia miocárdica, ocorrendo disfunção diastólica inicialmente, seguida ou não por disfunção sistólica. A redução na duração do insulto isquêmico está relacionada com a menor área de necrose miocárdica. Diversos estudos também sugerem que o restabelecimento da perfusão é responsável por algum grau de lesão miocárdica (lesão de reperfusão), principalmente em situações de oclusão total da coronária.^21^

### 5. Avaliação inicial

#### 5.1. Abordagem inicial

Pacientes com suspeita de SCA e sinais de gravidade (dor persistente, dispneia, palpitações decorrentes de arritmias potencialmente graves e síncope) devem ser encaminhados para serviços de emergência, idealmente monitorados em ambulância. Pacientes sem sinais de gravidade (ver sinais citados anteriormente) podem ser orientados a procurar por meios próprios o serviço de emergência mais próximo com capacidade de realizar ECG e dosagem de biomarcadores cardíacos, de preferência a troponina.

Pacientes com SCA devem ser prontamente avaliados quanto ao seu risco de complicações isquêmicas e hemorrágicas.

Diversas ferramentas (escores) foram idealizadas com esse intuito e, em conjunto com o julgamento clínico, podem auxiliar a definir quais pacientes se beneficiam de internação, exames complementares e tratamento específico.

#### 5.2. Diagnóstico e avaliação prognóstica

##### 5.2.1. História e dados clínicos

###### 5.2.1.1. Caracterização da dor torácica e angina

O quadro clínico de angina pode ser definido por quatro características principais da dor: localização, característica, duração e fatores de intensificação ou alívio.

- Localização: usualmente localizado no tórax, próximo ao esterno. Contudo, pode acometer ou irradiar do epigástrio à mandíbula, região interescapular e braços (mais comumente para o esquerdo, menos comumente para ambos ou para o braço direito).
- Tipo: o desconforto geralmente é descrito como pressão, aperto ou peso. Por vezes, como uma sensação de estrangulamento, compressão ou queimação. Pode ser acompanhado por dispneia, sudorese, náuseas ou síncope.
- Duração: o tempo de duração do desconforto anginoso estável geralmente é curta (< 10min); episódios ≥ 10min sugerem SCA. Contudo, duração contínua prolongada (horas ou dias) ou efêmera (poucos segundos) tem menor probabilidade de SCA.
- Fatores de intensificação ou alívio: uma importante característica da angina é a sua relação com o esforço físico. Os sintomas classicamente aparecem ou se intensificam ao esforço. Em pacientes com antecedente de angina, uma redução no limiar de esforço necessário para desencadear angina sugere SCA. O desconforto por SCA não costuma se modificar com relação à respiração ou posição.

A história clínica do paciente com SCASSST desempenha importante papel na estratificação de risco. Na presença de angina, pacientes com SCASSST podem se apresentar em quatro situações:

- Angina de repouso prolongada (> 20min).
- Angina de início recente (classe II ou III pela classificação da Canadian Cardiovascular Society): em geral, são pacientes que manifestam sintomas típicos de angina em um período inferior a 2 meses e progressão para esforços menores, habituais.
- Agravamento recente de angina estável prévia (angina em crescendo). Esforços menores desencadeando angina, aumento da intensidade e/ou duração da dor, alterações na irradiação, alterações no padrão de resposta ao uso de nitratos.
- Angina pós-infarto.

Algumas características, antecedentes e comorbidades se relacionam com uma maior probabilidade de SCA:

- Idade avançada e sexo masculino.
- Fatores de risco para aterosclerose: tabagismo, diabetes, dislipidemia, hipertensão arterial e insuficiência renal crônica.
- Antecedente familiar de DAC.
- Aterosclerose prévia sintomática, tais como doença arterial obstrutiva periférica, doença carotídea, doença coronariana prévia.
- Doenças inflamatórias crônicas, como lúpus ou atrite reumatoide.

Pacientes com SCA podem se apresentar com sintomas atípicos, tais como dor epigástrica isolada, sensação de plenitude gástrica, dor perfurante, dor pleurítica ou dispneia. Embora a principal forma de apresentação de SCA em mulheres e idosos (>75 anos) seja angina típica, a ocorrência de apresentações atípicas é maior nesses indivíduos, assim como em pacientes com diabetes, insuficiência renal e demência.

Sumariamente, a dor torácica pode ser classificada conforme proposto pelos investigadores do estudo CASS^22^ (Tabela 1.4).

**Tabela 1.4:** Classificação de angina proposta pelos investigadores do estudo CASS

Dor definitivamente anginosa	Dor retroesternal precipitada por esforço com irradiação para ombro, pescoço ou braço esquerdo e atenuada por repouso ou nitrato em menos de 10min
Dor provavelmente anginosa	Apresenta a maioria das características da dor definitivamente anginosa
Dor provavelmente não anginosa	Dor de característica atípica que não preenche critérios para dor anginosa
Dor definitivamente não anginosa	Dor sem correlação com atividade física, sugere ser de origem extracardíaca e não é atenuada por nitratos

Fonte: Adaptada de National Heart Lung and Blood Institute Coronary Artery Study.^22^

###### 5.2.1.2. SCASSST no Idoso

Os idosos com SCA geralmente apresentam perfil de risco diferente dos não idosos: têm maior prevalência de hipertensão arterial, diabetes melito, IAM prévio, angina, doença vascular periférica, acidente vascular cerebral (AVC), doença multiarterial e insuficiência cardíaca. Em geral, o idoso se apresenta para o atendimento médico mais tardiamente após o início dos sintomas. Na SCASSST, em vez de dor, frequentemente apresentam os chamados “equivalentes isquêmicos”, como dispneia, mal-estar, confusão mental, síncope ou edema pulmonar. Os idosos têm maior incidência de complicações na SCASSST, o que implica a necessidade de tratamento mais intensivo. Entretanto, especialmente naqueles acima de 75 anos de idade, frequentemente, a terapêutica mais adequada – com betabloqueador, ácido acetilsalicílico (AAS), anticoagulante e hipolipemiante – não é utilizada. No registro do estudo TIMI III,^23^ com 3.318 pacientes portadores de SCASSST, 828 pacientes tinham mais de 75 anos. Esses indivíduos receberam terapêutica anti-isquêmica e foram submetidos à cinecoronariografia em menor percentual, em relação aos mais jovens. Embora apresentassem DAC mais grave e extensa, foram menos frequentemente submetidos a procedimentos de revascularização miocárdica e tiveram mais eventos adversos em até 6 semanas de evolução. Em estudo de banco de dados nacional, a utilização de terapias comprovadamente eficazes após SCA aumentou nos últimos 15 anos tanto nos muito idosos (idade > 80 anos) quanto nos mais jovens (< 50 anos), sendo tal aumento associado à melhora da sobrevida pós-alta nos dois grupos.^24^

###### 5.2.1.3. História Pregressa

####### 5.2.1.3.1. Pacientes Submetidos a Procedimentos de Revascularização Miocárdica-Intervenção Coronária Percutânea e/ou Cirurgia de Revascularização do Miocárdio

A recorrência de angina após cirurgia de revascularização do miocárdio (CRVM) ou intervenção coronária percutânea (ICP) pode significar o desenvolvimento de complicações agudas, novas lesões, trombose do *stent* ou reestenose. Dor torácica até 48h após intervenção percutânea sugere a ocorrência de obstrução aguda, espasmo coronário transitório, trombo não oclusivo, oclusão de ramo ou embolização distal. A dor torácica recorrente cerca de 6 meses após implante de *stent* convencional ou, mais tardiamente, após implante de *stent* farmacológico está mais provavelmente relacionada à reestenose. Por outro lado, o aparecimento de angina após 1 ano do implante de *stent* geralmente se relaciona à nova lesão coronária ou a reestenose de *stent* por neoaterosclerose. No caso da CRVM, o aparecimento de dor precocemente geralmente se associa à obstrução trombótica do enxerto. Do primeiro mês até o primeiro ano pós-CRVM, o mecanismo geralmente é o de hiperplasia fibrosa da íntima; após esse período, é indicativo de nova lesão aterosclerótica e/ou degeneração não trombótica do enxerto. O registro TIMI III comparou a incidência de óbito ou infarto não fatal entre pacientes que apresentaram SCASSST com ou sem CRVM prévia. Os pacientes com CRVM prévia tiveram taxas mais elevadas de complicações tanto na análise de até 10 dias pós-admissão (4,5% no grupo com CRVM prévia *vs.* 2,8% no grupo sem CRVM) quanto na análise após 42 dias (7,7% *vs.* 5,1%, respectivamente), sugerindo que este seja um grupo de maior risco, sobretudo por apresentarem aterosclerose mais extensa.^25^

####### 5.2.1.3.2. Fatores de Risco para Doença Arterial Coronariana

Alguns estudos sugerem melhor evolução entre os tabagistas, provavelmente pelo fato de tais indivíduos sofrerem SCA em idade mais precoce e com menor carga aterosclerótica do que os não tabagistas.^26^^,^^27^ Por outro lado, Antman et al. demonstraram que a presença de três ou mais fatores de risco para DAC (hipertensão arterial sistêmica, diabetes, dislipidemia, história familiar e tabagismo) constitui marcador independente de pior prognóstico.^28^

##### 5.2.2. Exame Físico

Durante a avaliação de indivíduos com SCASSST, o exame físico auxilia na identificação de indivíduos de maior risco (aqueles com sinais de disfunção ventricular grave ou complicações mecânicas) e no diagnóstico diferencial da dor torácica não relacionada com SCA.

Como regra, o exame físico normal ou com discretas alterações é insuficiente para estratificação de risco do paciente, pois até pacientes com lesões multiarteriais ou de tronco de coronária esquerda podem apresentar exame físico normal.^29^^-^^31^ No entanto, quando presentes, as alterações no exame físico podem ter implicações importantes na categorização do paciente como de alto risco.

Entre os achados de mau prognóstico, destacam-se a presença de sopro sistólico em foco mitral, taquicardia, taquipneia, hipotensão, sudorese, pulsos finos, terceira bulha e estertores pulmonares.

Alterações do exame físico permitem o diagnóstico diferencial de SCA com outras causas de dor torácica:

- **Cardíacas**: pericardite (atrito pericárdico), tamponamento cardíaco (pulso paradoxal), estenose aórtica (sopro sistólico aórtico), miocardiopatia hipertrófica (sopro sistólico ejetivo paraesternal que aumenta com manobra de Valsalva).
- **Não cardíacas**: dissecção de aorta (divergência de pulso e pressão entre os braços e sopro diastólico de insuficiência aórtica), embolia pulmonar/infarto pulmonar (atrito pleural), pneumotórax (murmúrio vesicular diminuído e timpanismo à percussão), musculoesquelética (dor à palpação).

**Table:** 

História e exame físico - Sumário de recomendações e evidências		
Pacientes com suspeita de SCASSST e dor persistente, dispneia, palpitações ou síncope devem ser encaminhados para serviços de emergência, idealmente monitorados em ambulância.	I	C
Pacientes com suspeita de SCASSST com achados de menor gravidade, *(i. e.,* sem dor persistente, dispneia, palpitações ou síncope) podem procurar por meios próprios o serviço de emergência mais próximo com capacidade de realizar ECG e dosagem de troponina.	IIb	C

##### 5.2.3. Eletrocardiograma

O ECG de 12 derivações é a primeira ferramenta diagnóstica no manejo de pacientes com SCA suspeita. Idealmente, deve ser realizado e interpretado no atendimento pré-hospitalar ou em até 10min após a admissão hospitalar.

Cerca de 1% a 6% dos pacientes com SCASSST têm ECG normal, ou não diagnóstico, à admissão. Nessa situação, o ECG deve ser repetido entre 15 e 30min, principalmente em indivíduos que seguem sintomáticos. Um ECG normal ou não diagnóstico pode ocorrer mesmo na vigência da oclusão da artéria circunflexa ou da coronária direita. Dessa forma, recomenda-se a realização adicional das derivações V_3R_, V_4R_, V_7_, V_8_ e V_9_ para aumentar a sensibilidade do método.

Mais de 1/3 dos pacientes apresentam alterações características de SCA como depressão do segmento ST, elevação transitória de segmento ST e inversão de onda T. Alterações dinâmicas no segmento ST (depressão ou elevação do ST) ou inversões da onda T durante episódio doloroso, que se resolvem pelo menos parcialmente quando os sintomas são aliviados, são importantes marcadores de prognóstico adverso, ou seja, subsequente IAM ou morte.^32^ Pacientes com alterações de ST em derivações anteriores frequentemente apresentam estenose significativa da artéria coronária descendente anterior e constituem um grupo de alto risco.

Alterações do segmento ST e da onda T não são específicos da SCASSST e podem ocorrer em uma série de condições, que incluem: hipertrofia ventricular, pericardite, miocardite, repolarização precoce, alteração eletrolítica, choque, disfunção metabólica e efeito digitálico.

A acurácia diagnóstica de um ECG anormal aumenta quando se dispõe de um traçado de ECG prévio para comparação.

A presença de elevação transitória de segmento ST caracteriza a clássica descrição da angina variante de prinzmetal ou angina vasoespástica. A presença concomitante de elevação do ST nas derivações anteriores e inferiores (refletindo isquemia extensa) se associa a maior risco de morte súbita.^33^

###### 5.2.3.1. Achados do Eletrocardiograma e Prognóstico

Pacientes com SCASSST têm elevado risco de apresentarem, durante sua evolução, alterações isquêmicas de ECG (infra ou supra ST), fibrilação atrial e arritmias ventriculares. Essas alterações implicam pior prognóstico.^31^ No estudo GUSTO II, o ECG de apresentação dos pacientes com SCA teve importância prognóstica em relação à mortalidade precoce.^34^

Bloqueio de ramo esquerdo, hipertrofia ventricular esquerda ou ritmo de marca-passo (MP) cursaram com mortalidade de 11,6%; depressão do segmento ST com 8%; elevação do segmento ST com 7,4%; e inversão da onda T ou ECG normal com 1,2%. No estudo complementar com ECG do Registro TIMI III,^35^ em 1.416 pacientes com SCA, as seguintes formas de apresentação do ECG foram observadas: desvio do segmento ST ≥ 1mm em 14,3%; bloqueio de ramo esquerdo (BRE) em 19%; inversão isolada da onda T em 21,9%; e ausência dessas alterações em 54,9%.

**Table:** 

Eletrocardiograma - Sumário de recomendações e evidências		
Todos os pacientes com suspeita de SCASSST devem realizar ECG. Idealmente, o ECG deve ser realizado em até 10min após a chegada do paciente ao hospital.	I	B
O ECG deve ser repetido nos casos não diagnósticos, pelo menos uma vez, em até 6h.	I	C
O ECG deve ser repetido em caso de recorrência dos sintomas.	I	C
As derivações V3R-V4R, V7-V9 devem ser realizadas em pacientes que permanecem sintomáticos e apresentam ECG de 12 derivações não diagnóstico.	I	C

##### 5.2.4. Biomarcadores

Marcadores bioquímicos são úteis para auxiliar tanto no diagnóstico quanto no prognóstico de pacientes com SCA. Quando as células miocárdicas sofrem lesão, suas membranas celulares perdem a integridade, as proteínas intracelulares se difundem no interstício e vão para os linfáticos e capilares. Após a lesão miocárdica, a cinética dos marcadores depende de diversos fatores: o compartimento intracelular das proteínas; o tamanho das moléculas; o fluxo regional linfático e sanguíneo; e a taxa de depuração do marcador. Tais fatores, em conjunto com as características de cada marcador, diferenciam o desempenho diagnóstico de cada um para IAM.^36^

Em pacientes que se apresentam com quadro sugestivo de SCA, nos quais o diagnóstico de IAM não está estabelecido, os biomarcadores cardíacos são úteis para confirmar o diagnóstico de infarto. Além disso, os mesmos fornecem importantes informações prognósticas, na medida em que existe uma direta associação entre a elevação dos marcadores séricos e o risco de eventos cardíacos a curto e médio prazo.^37^ Os resultados dos marcadores de necrose devem estar disponíveis em até 60min a partir da coleta. Caso a estrutura disponível de laboratório de análises clínicas não viabilize esta meta, as tecnologias *point of care* devem ser consideradas.^38^

###### 5.2.4.1. Troponinas

As troponinas são proteínas do complexo de regulação miofibrilar presentes no músculo estriado cardíaco. Existem três subunidades: troponina T, troponina I e troponina C. A troponina C é coexpressa nas fibras musculares esqueléticas de contração lenta e não é considerada como um marcador específico cardíaco. Nas últimas décadas, foram desenvolvidas técnicas de imunoensaios com anticorpos monoclonais específicos para troponinas T cardíaca (TnTc) e troponina I cardíaca (TnIc). Metanálises demonstraram que TnIc tem sensibilidade e especificidade clínica para o diagnóstico de IAM na ordem de 90% e 97%, respectivamente. As troponinas cardíacas permanecem elevadas por tempo mais prolongado, podendo permanecer elevadas por até 7 dias depois do IAM. As troponinas são os biomarcadores de primeira escolha para avaliação diagnóstica de pacientes com suspeita de IAM, pois apresentam acurácia diagnóstica superior à da CK-MB massa e dos demais biomarcadores de lesão miocárdica. Pacientes com troponinas elevadas apresentam risco aumentado de eventos cardíacos nos primeiros dias de internação e identificam um subgrupo de SCASSST com potencial maior de benefício para manuseio invasivo.^39^ Apesar da identificação acurada de lesão miocárdica pelas troponinas, elas não identificam o(s) mecanismo(s) de lesão; estes podem ser múltiplos, incluindo etiologias não coronarianas como taquiarritmias e miocardite, ou ainda condições não cardíacas, como sepse, embolia pulmonar e insuficiência renal.^40^ Assim, principalmente naqueles casos em que a apresentação clínica não é típica de SCA, devem ser consideradas outras causas de lesão cardíaca relacionadas com aumento de troponinas.

As troponinas têm reconhecido valor na avaliação de pacientes com alterações isquêmicas no ECG ou com clínica sugestiva de dor anginosa. A maior limitação das troponinas convencionais é sua baixa sensibilidade quando o paciente tem um tempo de início do quadro inferior a 6h. Com a introdução das troponinas de alta sensibilidade (Trop-US), passou a ser possível a detecção de níveis mais baixos de troponina, em menor tempo após início do quadro de lesão miocárdica de causa isquêmica.^41^^,^^42^ A unidade usada para expressar os valores da troponina convencional é ng/mL, e nas Trop-US, os valores podem ser expressos em ng/L, com poder de detecção 10 a 100 vezes maior que o das troponinas convencionais.

Nos pacientes que chegam ao serviço de emergência com menos de 3h do início do quadro, as Trop-US são significativamente mais sensíveis que a troponina convencional para diagnóstico de SCA, melhorando em 61% o poder diagnóstico de IAM naquele momento e em 100% se a coleta for 6h após o início do quadro.^43^ Com o aumento da sensibilidade e da acurácia diagnóstica para detecção do IAM utilizando a troponina ultrassensível, foram propostos algoritmos de diagnóstico acelerado. Assim, podemos reduzir o tempo até o diagnóstico, traduzindo menor tempo de permanência na emergência e menor custo.^44^^-^^46^ É recomendada a utilização do algoritmo de investigação em 3h (ver fluxograma de rotina diagnóstica e critérios de internação)

###### 5.2.4.2. Creatinoquinase, suas Isoenzimas e Isoformas

Antes da consolidação das troponinas como biomarcadores com maior acurácia para diagnóstico do IAM, a creatinoquinase MB (CK-MB) era o biomarcador mais utilizado nos protocolos de dor torácica. Idealmente, a CK-MB deve ser mensurada por imunoensaio para dosagem da sua concentração no plasma (CK-MB massa) em vez da sua atividade. Essa mudança no padrão de aferição se deve, em parte, a estudos que demonstraram uma maior sensibilidade e especificidade para IAM com o uso de CK-MB massa.^47^ As subformas da CK-MB têm surgido como marcadores precoces (menos de 6h) de lesão miocárdica e inferência precoce da magnitude do IAM, conforme dado de estudo de necrópsia que demonstrou melhor correlação entre CK-MB massa e tamanho de IAM.^48^ No entanto, a CK-MB massa apresenta como principal limitação elevar-se após dano em outros tecidos não cardíacos (falso-positivos), especialmente após lesão em músculo liso e esquelético. Podem acontecer resultados falso-positivos, em que a CK-MB é positiva e a troponina é negativa em cerca de 4% dos pacientes.^49^ Nos casos em que a CK-MB está elevada e a troponina está normal, ambas dentro de sua janela cinética, deve-se basear a decisão clínica no resultado da troponina.

**Table:** 

Marcadores bioquímicos - Sumário de recomendações e evidências		
As troponinas são os biomarcadores de escolha no diagnóstico de pacientes com suspeita de IAM.	I	A
Na disponibilidade de troponina ultrassensível, nenhum outro marcador deve ser solicitado rotineiramente para diagnóstico de IAM.	I	B
Biomarcadores bioquímicos de necrose miocárdica devem ser mensurados em todos os pacientes com suspeita de SCASSST. Quando troponina ultrassensível estiver disponível, a dosagem sérica deve ser realizada na admissão e, idealmente, reavaliada em 1h ou até 2h. Caso indisponível, a troponina convencional deve ser coletada na admissão e repetida pelo menos uma vez, 3 a 6h após, caso a primeira dosagem seja normal ou discretamente elevada.	I	B
Dosagens CK-MB massa podem ser utilizadas se dosagens de troponina não estiverem disponíveis.	IIb	B
Utilização da mioglobina para detecção de necrose miocárdica em pacientes com suspeita de SCASSST.	III	

##### 5.2.5. Exames de Imagem Não Invasivos na Emergência

###### 5.2.5.1. Avaliação Funcional

Os exames não invasivos têm importante papel no diagnóstico (sobretudo em pacientes com ECG e biomarcador normal) e na estratificação de risco do paciente com suspeita de SCA. A escolha de cada um – quer teste ergométrico, cintilografia de perfusão miocárdica, ressonância cardíaca ou angiografia por tomografia computadorizada das artérias coronárias – dependerá do objetivo e da questão clínica a ser respondida.^50^

###### 5.2.5.2. Teste Ergométrico na Sala de Emergência

Pacientes com dor torácica no setor de emergência, identificados como de risco baixo ou intermediário, podem ser submetidos ao teste ergométrico (TE), cujo resultado normal confere risco anual baixo de eventos cardiovasculares, permitindo a alta hospitalar mais precoce e segura.^50^ Diretrizes nacionais e internacionais recomendam o TE como exame de primeira escolha para a estratificação de risco em pacientes que possam realizar exercício, por ser procedimento de baixo custo, ter larga disponibilidade e com baixa frequência de intercorrências.^51^ No entanto, devem ser afastadas as situações de SCA de moderado a alto risco e alto risco, doenças agudas da aorta, tromboembolismo pulmonar, miocardite e pericardite, pois são contraindicações absolutas para realizar esse exame. Os protocolos devem ser definidos de acordo com as condições clínicas do paciente, sendo os mais recomendados em rampa, Naughton ou Bruce modificado.

Resumo das indicações do TE na SCA (caracterizar baixo risco após estratificação clínica inicial):

- ECG basal e biomarcadores (necrose) sem alterações.
- Ausência de sintomas (dor precordial ou dispneia).
- Estabilidade hemodinâmica e condições adequadas para o esforço físico.

Se o resultado do TE for normal e o paciente tiver evidenciado boa capacidade funcional, outros procedimentos podem não ser necessários em virtude do alto valor preditivo negativo da prova.^51^

**Table:** 

Teste ergométrico - Sumário de recomendações e evidências		
Pacientes de risco baixo (clínica e ECG) e com biomarcadores normais devem ser encaminhados para teste ergométrico após 9 a 12h em observação. Dentro das rotas da unidade de dor torácica (UDT), estes exames podem ser feitos como critério de alta.	I	B
Na impossibilidade de realização do TE ou ECG não interpretável (bloqueio de ramo esquerdo, marca-passo artificial, fibrilação atrial, sobrecarga ventricular esquerda etc.), o paciente pode ser submetido a exames provocativos de isquemia associados à imagem não invasiva	I	B

###### 5.2.5.3. Ecocardiografia

O ecocardiograma é um método complementar de grande utilidade na avaliação da dor torácica na emergência.^52^^-^^54^ É um exame não invasivo, e a informação diagnóstica é disponibilizada em curto espaço de tempo.^55^^-^^59^ Quando realizado durante um episódio de dor precordial, a ausência de anormalidade de contração segmentar ventricular é uma evidência contrária à isquemia como causa do sintoma. Embora o ecocardiograma não seja capaz de garantir se a alteração segmentar é recente ou preexistente, a presença de anormalidades de contração segmentar reforça a probabilidade de DAC, sendo indicativa de infarto, isquemia ou ambos, embora possa também ser evidenciada em casos de miocardites.^60^^-^^62^

Além disso, outras etiologias não menos importantes de dor torácica – tais como dissecção aórtica, estenose aórtica, miocardiopatia hipertrófica e doença pericárdica – podem ser avaliadas pelo método. Doença coronária importante é habitualmente encontrada em pacientes com AI. Esses pacientes são geralmente identificados por história clínica, e alterações eletrocardiográficas reversíveis podem ser detectadas, concomitantes aos episódios de dor. Quando a história e o ECG não são típicos, a documentação de anormalidade da contração segmentar ao ecocardiograma, durante ou imediatamente após um episódio doloroso, sugere fortemente o diagnóstico.^63^ O ecocardiograma avalia ainda a presença e a extensão da disfunção ventricular, assim como a presença e a gravidade de anormalidades valvares.

Além da fração de ejeção do ventrículo esquerdo (FEVE) e da motilidade segmentar, outros parâmetros também são importantes para se avaliar prognóstico. Ersbǿll et al. estudaram pacientes com infarto e FEVE > 40% dentro de 48h da admissão de forma prospectiva. Todos os pacientes foram submetidos à ecocardiografia com avaliação semiautomatizada de *strain global longitudinal* (SGL). De um total de 849 pacientes, 57 (6,7%) tiveram eventos cardíacos graves, e SGL > –14% se associou a um aumento de 3 vezes no risco daqueles eventos.^63^

###### 5.2.5.4. Ecocardiografia de Estresse

A ecocardiografia de estresse vem adquirindo aceitação crescente na avaliação de pacientes no departamento de emergência, e precocemente após hospitalização.^64^ Um estudo analisou 108 pacientes observados por 4h com enzimas seriadas e ECG e submetidos a teste ergométrico ou à ecocardiografia de estresse com dobutamina. Dez pacientes evidenciaram positividade ao teste ergométrico. O mesmo aconteceu com 8 pacientes à ecocardiografia de estresse. Os exames foram concordantes em 4 pacientes. Todos os pacientes com ecocardiografia de estresse sem evidência de isquemia estiveram livres de eventos cardíacos ao final de 12 meses de seguimento, bem como 97% dos pacientes com testes ergométricos negativos.^65^

Estudo nacional avaliou 95 pacientes com AI de baixo e moderado risco com o ecocardiograma sob estresse com dobutamina, a maioria estudada nas primeiras 72h de internação hospitalar. Em relação aos desfechos clínicos analisados (morte, IAM, nova internação por AI e procedimentos de revascularização miocárdica), o exame demonstrou ser seguro e com um excelente valor preditivo negativo (96%), permitindo alta hospitalar precoce sem a necessidade de outros exames.^66^ A utilização de técnicas de perfusão para realização do ecocardiograma de estresse pode aumentar sensibilidade diagnóstica, e suas aplicações são discutidas.na Parte 2 desta diretriz.

Por ser um exame acessível, rápido, não invasivo e de baixo custo, a ecocardiografia tem a capacidade de oferecer informação prognóstica adicional aos parâmetros anteriormente citados, por meio da avaliação da função ventricular global, regional e a identificação de valvopatia associada, podendo ser utilizado rotineiramente na investigação desses pacientes. Suas desvantagens são janela limitada em alguns pacientes e sensibilidade reduzida do estresse com dobutamina em pacientes utilizando betabloqueador.

**Table:** 

Ecocardiografia - Sumário de recomendações e evidências		
O ecocardiograma transtorácico deve ser realizado no diagnóstico diferencial com outras doenças, quando houver suspeita clínica de doenças de aorta, doenças do pericárdio, embolia pulmonar e valvopatias.	I	C
Ecocardiograma de urgência nos casos de complicações decorrentes de SCASSST, como comunicação interventricular e insuficiência mitral.	I	C
Ecocardiografia de estresse pode ser utilizada como método de estratificação funcional em pacientes sem dor torácica recorrente e sem evidências eletrocardiográficas de isquemia e/ou elevação de troponina.	I	B
Pacientes em vigência de dor torácica podem ser avaliados por ecocardiograma em repouso, para verificar a presença de alterações compatíveis com etiologia isquêmica.	IIa	C

###### 5.2.5.5. Cardiologia Nuclear

Vários estudos sugerem que indivíduos com uma cintilografia miocárdica em repouso de baixo risco, realizada na unidade de emergência, apresentam risco de eventos cardíacos subsequentes bastante reduzido. Por outro lado, em pacientes com uma CMP de alto risco, há probabilidade muito aumentada de eles desenvolverem IAM, serem revascularizados (cirurgia ou angioplastia) ou de apresentarem lesões coronárias obstrutivas à coronariografia.^67^^-^^69^ No estudo ERASE (Emergency Room Assessment of Sestamibi for Evalution of Chest Pain), em que foram avaliadas estratégias de atendimento de pacientes com SCA com ECG normal ou não diagnóstico, ainda na sala de emergência, observou-se taxa de admissão de 54% para os pacientes que realizaram CMP e de 63% para os outros, sugerindo que a estratégia inicial com estudo cintigráfico ao repouso é um bom estratificador de risco.^70^

Diretrizes internacionais recomendam o emprego da imagem de perfusão miocárdica em repouso na dor torácica aguda para estratificação de risco em pacientes com suspeita de SCA e ECG não diagnóstico.^71^^-^^73^

*Momento de injeção do radiofármaco:*

As principais aplicações da CPM dentro das primeiras horas da chegada do paciente ao hospital são:

- Injeção do radiofármaco (sestamibi/MIBI ou tetrofosmin marcados com tecnécio-99m, ou sestamibi-99mTc e tetrofosmin-99mTc) em repouso, durante o episódio de dor torácica, com ECG normal ou inespecífico, objetivando rápida definição diagnóstica.
- Injeção do radiofármaco em repouso, na ausência de dor torácica, com ECG normal ou inespecífico, com cessação do sintoma há menos que 6h, mas preferencialmente dentro das 2h precedentes. Wackers et al. demonstraram, nos casos de injeção realizada até 6h da dor na SCA, que a incidência de anormalidades de perfusão foi de 84%, diminuindo para 19% quando a administração intravenosa do radiofármaco ocorreu entre 12 e 18h do último episódio doloroso.^74^

De modo global, as imagens de perfusão em repouso com radiofármacos na avaliação da dor torácica aguda não apresentam contraindicações formais e são bem toleradas pela maioria dos pacientes. A CMP com estresse físico ou estímulo farmacológico nos pacientes com SCA de risco baixo ou intermediário é recomendada após estabilização do quadro agudo, sendo realizada habitualmente durante hospitalização. Condições clínicas e hemodinâmicas estáveis são primordiais em ambas as situações. Limitações do método são disponibilidade no nosso meio e custo.

**Table:** 

Cardiologia nuclear - Sumário de recomendações e evidências		
CPM em repouso na dor torácica aguda para estratificação de risco em pacientes com suspeita clínica de SCA e ECG não diagnóstico.	I	A
Cintilografia miocárdica de estresse (físico ou farmacológico) pode ser utilizada como método de estratificação funcional em pacientes sem dor torácica recorrente, sem evidências eletrocardiográficas de isquemia e/ou elevação de troponina	I	B
Pacientes em vigência de dor torácica e eletrocardiograma sem alterações isquêmicas podem ser avaliados pela cintilografia miocárdica de perfusão em repouso para determinar a origem isquêmica ou não da dor	IIa	A

###### 5.2.5.6. Avaliação Anatômica: Angiotomografia das Artérias Coronárias

Nos últimos anos, a angiotomografia (angioTC) das artérias coronárias se tornou uma ferramenta cada vez mais utilizada na avaliação de pacientes com suspeita de doença coronariana obstrutiva. A acurácia do método para o diagnóstico de estenose luminal, quando comparada à angiografia coronariana invasiva, já está bem demonstrada, com destaque para o seu alto valor preditivo negativo.^75^^-^^77^ Além disso, vários trabalhos comprovam o valor prognóstico do método relacionado à presença e extensão de DAC obstrutiva e não obstrutiva, testado em diversas situações clínicas, auxiliando na tomada de decisões.^78^^,^^79^

Três grandes estudos multicêntricos, controlados e randomizados avaliaram o uso da angioTC na dor torácica atendida em unidades de emergência, incluindo, no total, mais de 3 mil pacientes. O estudo multicêntrico CT-STAT randomizou 699 pacientes com dor torácica de baixo risco para estratégias de estratificação utilizando a angioTC de coronárias ou a cintilografia miocárdica de repouso e estresse.^80^ A estratégia com a angioTC reduziu em 54% o tempo para o diagnóstico e em 38% os custos da internação, sem que houvesse diferença na taxa de eventos adversos com relação à estratégia com a cintilografia. O estudo ACRIN-PA teve como objetivo primário avaliar a segurança da utilização da angioTC na avaliação de pacientes com dor torácica de risco baixo e intermediário em comparação com a abordagem tradicional.^81^ Nenhum dos pacientes com angioTC negativa apresentou o desfecho primário analisado, composto de morte cardíaca ou infarto nos primeiros 30 dias após a admissão. Além disso, os pacientes do grupo angioTC tiveram maior taxa de alta das unidades de emergência (49,6% *vs.* 22,7%) e menos dias de internação (18h *vs.* 24,8h, p < 0,001), sem diferenças significativas nas incidências de coronariografias ou revascularizações em 30 dias. Finalmente, o estudo ROMICAT II avaliou, em grupos semelhante de pacientes, o tempo de permanência na unidade de emergência e custos hospitalares.^82^ O estudo incluiu 1.000 pacientes com idade média de 54 anos (46% do sexo feminino). O tempo de permanência no hospital foi significativamente menor nos pacientes estratificados para angioTC quando comparados ao grupo submetido à avaliação tradicional (23,2 ± 37h *vs.* 30,8 ± 28h; p = 0,0002). O tempo até a exclusão do diagnóstico de SCA também foi menor no grupo submetido à angioTC (17,2 ± 24,6h *vs.* 27,2 ± 19,5h; p < 0,0001). Em relação às metas de segurança, não houve diferença significativa entre os grupos. No grupo estratificado pela angioTC, houve aumento significativo dos pacientes que receberam alta hospitalar diretamente da emergência (46,7% *vs.* 12,4%; p = 0,001), mas o uso de testes diagnósticos foi significativamente maior neste grupo (97% *vs.* 82%, p < 0,001). Apesar do custo mais elevado associado à realização da angioTC e de uma tendência a maior número de cateterismos e revascularizações, os custos globais foram similares entre os dois grupos (p = 0,65).

Metanálise posteriormente publicada confirmou que a estratégia de avaliação de pacientes com dor torácica aguda utilizando a angioTC das artérias coronárias, em comparação com abordagens tradicionais, está relacionada à redução de custo e tempo de internação, com aparente aumento do número de angiografias invasivas e revascularização miocárdica.^83^

####### 5.2.5.6.1. Descarte Triplo

A angioTC pode ser utilizada na sala de emergência tanto para a visualização das artérias coronárias quanto para obter informações relativas à aorta e às artérias pulmonares, permitindo a avaliação de síndromes aórticas agudas, tromboembolismo pulmonar ou outras alterações torácicas que possam ser diagnósticos diferenciais das SCA (como pneumonias e traumatismos).^84^^,^^85^ Por meio de protocolos de aquisição específicos, todas essas informações podem ser obtidas em um único exame. Essa abordagem recebe o nome de descarte triplo (*triple rule-out*), porém só deve ser utilizada em situações específicas, nas quais a avaliação clínica é incapaz de direcionar o diagnóstico.^86^

Em resumo, a utilização da angioTC das artérias coronárias nas unidades de emergência é uma estratégia segura e eficiente para a avaliação de pacientes com dor torácica aguda de risco baixo e intermediário, reduzindo o tempo para o diagnóstico correto e o tempo de internação.^87^ Desvantagens do método são a utilização de radiação ionizante, custo elevado, necessidade de contraste iodado, limitação em pacientes com frequência cardíaca acima de 80 batimentos por minuto ou que não possam utilizar betabloqueador e disponibilidade reduzida em nosso meio.

**Table:** 

Angiografia por tomografia computadorizada das artérias coronárias - Sumário de recomendações e evidências		
Em pacientes com dor torácica aguda de probabilidade baixa a intermediária de DAC, com ECG não diagnóstico e marcadores de necrose miocárdica negativos.	I	A
Investigação da dor torácica aguda pela técnica do descarte triplo *(triple rule-out).*	IIb	B

#### 5.3. Estratificação de risco

##### 5.3.1. Estratificação de risco de eventos isquêmicos cardiovasculares

**Figura 1.3:**
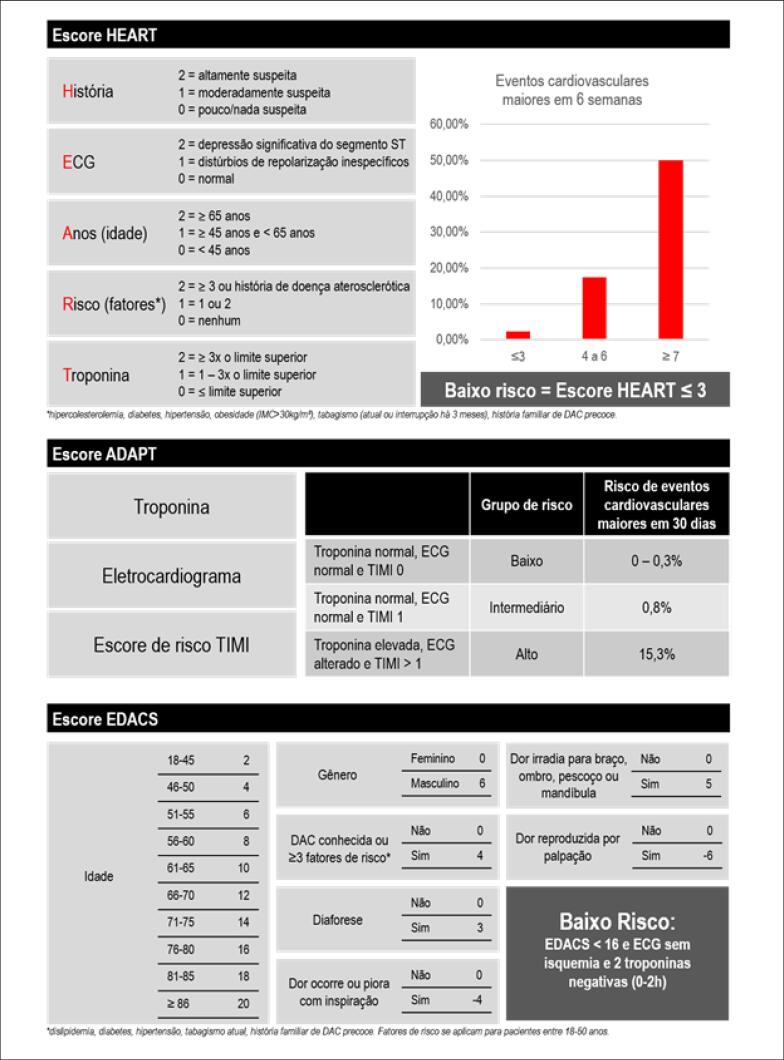
Escores de estratificação de risco clínico para dor torácica

Antman et al., a partir de uma análise do banco de dados do estudo TIMI (Thrombolysis In Myocardial Infarction) 11B, encontraram os seguintes marcadores independentes de pior prognóstico em pacientes com SCASSST (“escore de risco do grupo TIMI”): idade ≥ 65 anos; elevação de marcadores bioquímicos; depressão do segmento ST ≥ 0,5mm; uso de AAS nos 7 dias prévios aos sintomas; presença de três ou mais fatores de risco tradicionais para DAC (hipertensão, hipercolesterolemia, diabetes melito, tabagismo, história familiar); DAC conhecida; angina grave recente (< 24h).^28^ Conferindo um ponto para cada um desses itens, o paciente é classificado em baixo risco (escore de 0 a 2), risco intermediário (escore de 3 a 4) ou alto risco (escore de 5 a 7). Tal escore de risco foi validado em outros estudos de SCASSST, observando-se em todos eles uma associação entre elevação na incidência de eventos (óbito, reinfarto e isquemia recorrente necessitando de revascularização) e maior escore de risco (Figura 1.3).

**Figura 1.4:**
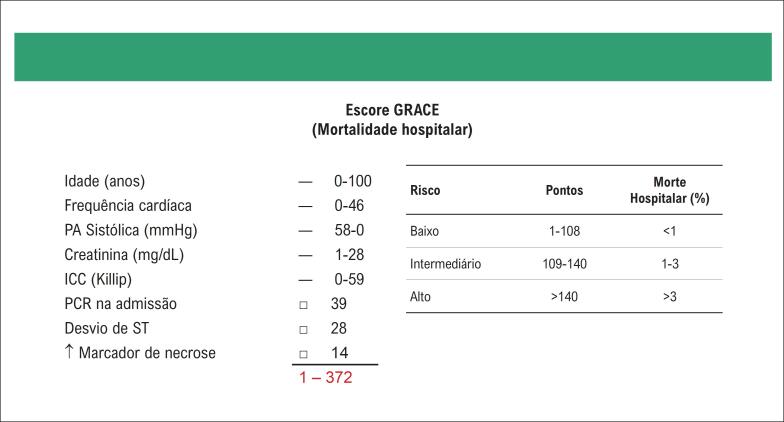
Escore de risco GRACE.

O escore de risco GRACE (Global Registry of Acute Coronary Events) permite uma estratificação mais acurada tanto na admissão quanto na alta hospitalar, graças ao seu bom poder discriminatório (Figura 1.4). Entretanto, apresenta maior complexidade, com a necessidade da utilização de computador ou aparelho digital de uso pessoal para o cálculo do risco.^88^ O escore GRACE original fornece uma estimativa de óbito intra-hospitalar ou óbito e IAM em 6 meses após a alta e, posteriormente, o escore também foi validado para estimativa de risco de 1 e 3 anos. Neste escore, 8 variáveis prognósticas de mortalidade hospitalar foram identificadas, sendo o escore total de um determinado paciente obtido pela soma dos pontos de cada uma delas:

1. Idade em anos – variando de 0 ponto (< 30) a 100 pontos (> 90).
2. Frequência cardíaca (bpm) – variando de 0 ponto (< 50) a 46 pontos (> 200).
3. Pressão arterial sistólica (mmHg) – variando de 0 ponto (> 200) a 58 pontos (< 80).
4. Níveis de creatinina (mg/dL) – variando de 1 ponto (< 0,40) a 28 pontos (> 4,0).
5. Insuficiência cardíaca (Classe Killip) – variando de 0 ponto (classe I) a 59 pontos (classe IV).
6. Parada cardíaca na admissão – variando de 0 ponto (não) a 39 pontos (sim).
7. Desvio do segmento ST – variando de 0 ponto (não) a 28 pontos (sim).
8. Elevação dos níveis de biomarcadores de lesão cardíaca – variando de 0 ponto (não) a 14 pontos (sim).

Fonte: adaptada de Granger et al.^89^

Quando a soma dos pontos for ≤ 108, o paciente é considerado de baixo risco para óbito hospitalar, cuja incidência é ≤ 1%. Quando se situa entre 109 e 140 (risco intermediário) a mortalidade está entre 1% e 3%; quando a soma for maior que 140 (alto risco), a mortalidade é superior a 3%.^89^ Uma nova versão do ESCORE GRACE (GRACE 2.0), com as mesmas variáveis preditoras de desfecho, foi desenvolvida. ampliando a estimativa de risco para os desfechos de morte intra-hospitalar aos 6 meses, 1 ano e 3 anos e de risco de morte ou IAM em 1 ano.^90^ A Tabela 1.5 mostra a estratificação de risco baseada em variáveis clínicas, eletrocardiográficas e laboratoriais.

**Tabela 1.5:** Estratificação de risco de morte ou infarto em pacientes com síndrome isquêmica aguda sem supradesnível do segmento ST^91^

	Alto	Moderado	Baixo
Varável Prognóstica	Pelo menos uma das características a seguir deve estar presente	Nenhuma característica de alto risco, mas com alguma das que seguem adiante	Nenhuma característica de risco intermediário ou alto, mas com alguma das que seguem adiante
História	Agravamento dos sintomas nas últimas 48h. Idade > 75 anos	Idade = 70 a 75 anosInfarto prévio, doença cerebrovascular ou periférica, diabetes melito, cirurgia de revascularização, uso prévio de AAS	
Dor precordial	Dor prolongada (> 20min), em repouso.	Angina de repouso > 20min, resolvida, com probabilidade de DAC moderada a alta Angina em repouso ≤ 20min, com alívio espontâneo ou com nitrato	Novo episódio de angina classe III ou IV da CCS nas últimas 2 semanas sem dor prolongada em repouso, mas com moderada ou alta probabilidade de DAC
Exame físico	Edema pulmonar, piora ou surgimento de sopro de regurgitação mitral, B_3_, novos estertores, hipotensão, bradicardia ou taquicardia		
Eletrocardiograma	Infradesnível do segmento ST ≥ 0,5mm (associado ou não com angina), alteração dinâmica do ST, bloqueio completo de ramo, novo ou presumidamente novo. Taquicardia ventricular sustentada	Inversão onda T > 2mm; ondas Q patológicas.	Normal ou inalterado durante o episódio de dor
Marcadores séricos de isquemia*	Acentuadamente elevados	Discretamente elevados	Normais

* Troponina I cardíaca (Tnlc);

Troponina T cardíaca (TnTc) ou CK-MB (preferencialmente massa) elevados = acima do percentil 99; elevação discreta = acima do nível de detecção e inferior ao percentil 99. CCS: Canadian Cardiovascular Society; DAC: doença arterial coronariana.

O escore HEART avalia o risco de um evento cardíaco maior (infarto, necessidade ou revascularização ou morte) em 6 semanas após sua apresentação inicial em pacientes atendidos com dor torácica, e será discutido no item 5.4.

##### 5.3.2. Estratificação de Risco de Sangramento

O sangramento é associado com prognóstico adverso nas SCASSST, e todos os esforços devem ser implementados para reduzi-lo, sempre que possível. Algumas variáveis podem auxiliar a classificar os pacientes em diferentes níveis de risco para sangramento maior durante a hospitalização. Escores de risco de sangramento foram desenvolvidos baseados em coortes de registros e de estudos clínicos no cenário de SCA. O escore CRUSADE (Can rapid risk stratification of unstable angina patients supress adverse) (www.crusadebleedingscocre.org/) foi desenvolvido a partir de uma coorte de 71.277 pacientes do registro de mesmo nome, posteriormente validado em coorte de 17.857 pacientes do mesmo registro^92^ (Tabela 1.6 e, em população brasileira foi preditor não apenas de sangramento mas também de mortalidade intra-hospitalar (área sob a curva ROC = 0,753, p < 0.001).^93^ A taxa de sangramento maior aumentou gradualmente com a elevação do escore de risco de sangramento. Esse escore tem uma acurácia relativamente alta para estimar o risco de sangramento por incorporar variáveis de admissão e de tratamento. Nesse escore, a idade não está listada entre os fatores prognósticos, mas está contida no cálculo do *clearance* de creatinina.

**Tabela 1.6:** Escore de risco de sangramento CRUSADE (referência b) | Algoritmo usado para determinar o escore de risco CRUSADE para sangramento maior intra-hospitalar

Fator prognóstico	Escores
**Hematócrito basal (%)**	
< 31	9
31-33,9	7
34-36,9	3
37-39,9	2
> 40	0
**Clearance de creatinina (mL/min)**	
< 15	39
16-30	35
31-60	28
61-90	17
91-120	7
> 120	0
**Frequência cardíaca (bpm)**	
< 70	0
71-80	1
81-90	3
91-100	6
101-110	8
111-120	10
> 120	11
**Sexo**	
Masculino	0
Feminino	8
**Sinais de IC na apresentação**	
Não	0
Sim	7
**Doença vascular prévia**	
Não	0
Sim	6
**Diabetes melito**	
Não	0
Sim	6
**Pressão arterial sistólica (mmHg)**	
< 90	10
91-100	8
101-120	5
121-180	1
181-200	3
> 200	5

IC: insuficiência cardíaca.

Outro escore foi derivado dos estudos ACUITY e HORIZONS. Seis variáveis independentes (sexo feminino, idade avançada, creatinina séria elevada, contagem de leucócitos elevada, anemia, SCA com ou sem elevação de ST) e uma variável relacionada ao tratamento (uso de heparina e inibidor de GP IIbIIIa no lugar de bivalirudina isolada), foram identificadas (Tabela 1.7). Esse escore identificou pacientes em risco aumentado de sangramento não relacionado à CRVM e mortalidade após 1 ano.^94^

**Tabela 1.7:** Escore de risco de sangramento proposto por Mehran et al.^94^ Algoritmo usado para determinar o escore de risco para sangramento maior intra-hospitalar

Soma		
Sexo	Homens Mulheres 0 +8	
Idade (anos)	< 50 50-69 60-69 70-79 ≥ 80 0 +3 +6 +9 +12	
Creatinina sérica (mg/dL)	< 1,0 1,0- 1,2- 1,4- 1,6- 1,8- ≥ 2,0 0 +2 +3 +5 +6 +8 +10	
Leucócitos totais (giga/mL)	< 10 10- 12- 14- 16- 18- ≥ 20 0 +2 +3 +5 +6 +8 +10	
Anemia	Não Sim 0 +6	
Apresentação da SCA	IAM com supra – IAM sem supra – angina instável +6 +2 0	
Medicações antitrombóticas	Heparina+IGP IIb/IIIa – bivalirudina 0 –5	
	**Valor total**	

Ambos os escores foram derivados de coortes em que o acesso femoral foi predominantemente ou exclusivamente utilizado. Seu valor prognóstico pode ser menor no cenário de acesso radial.^95^

Finalmente, os autores salientam que nenhum escore (seja de eventos isquêmicos, seja de eventos hemorrágicos) deve substituir a avaliação clínica. São apenas ferramentas que auxiliam o médico na sua decisão.

**Table:** 

Estratificação de risco - Sumário de recomendações e evidências		
Todos pacientes devem ser estratificados e classificados em risco alto, intermediário ou baixo de desenvolverem eventos cardíacos maiores. É recomendável a classificação por mais de um método, e o pior cenário deve ser considerado nas decisões quanto às condutas a serem adotadas.	I	B
Todos os pacientes devem ser estratificados e classificados em risco alto, intermediário ou baixo de sangramento.	I	B

#### 5.4. Fluxograma de Rotina Diagnóstica na Emergência e Critérios para Hospitalização

É fundamental um fluxograma de rotina diagnóstica do paciente com dor torácica, definindo os critérios para alta precoce e internação hospitalar. Isso permite identificar pacientes de baixo risco, que podem ser investigados em ambiente ambulatorial, assim como a detecção de condições cardiológicas de maior gravidade que demandam investigação e internação hospitalar.

A triagem da dor torácica no serviço de emergência é baseada em uma breve história clínica, exame físico, ECG de 12 derivações em até 10min após chegada e mensuração de biomarcadores. Esta rotina de investigação visa principalmente identificar precocemente o paciente de maior risco que necessita de internação hospitalar ou urgente transferência para serviço de hemodinâmica.

Com o objetivo de evitar uma alta inadvertida, esses pacientes devem permanecer monitorados onde um protocolo de diagnóstico acelerado possa ser utilizado. Este protocolo envolve ECG seriado e mensuração de troponina ultrassensível na chegada à emergência e 3h após. Além desse protocolo, a incorporação de escores de risco aplicáveis na sala de emergência, que integram dados demográficos, sintomas, achados eletrocardiográficos e biomarcadores, representa uma importante ferramenta de auxílio para o emergencista na avaliação dos pacientes com dor torácica. Os escores mais utilizados são: HEART, ADAPT e EDACS.^96^^-^^98^ Tais escores estão sumarizados na Figura 1.3.

Greenslade et al. realizaram estudo comparando a eficácia desses escores na liberação precoce no serviço de emergência, verificando que todos os escores foram eficazes com sensibilidade muito elevada.^99^

O escore HEART foi construído utilizando troponina convencional como biomarcador. No entanto, estudos retrospectivos que utilizaram troponina ultrassensível apresentaram resultados similares aos observados nos estudos de validação.^100^^,^^101^ Não existem estudos validando o uso desta ferramenta no atendimento extra-hospitalar. Contudo, há um estudo (ARTICA) em curso que visa validar o uso deste escore no atendimento pré-hospitalar.^102^

O escore HEART de 0 a 3 permite identificar 35% a 46% de pacientes com baixo risco, exibindo alta sensibilidade e valor preditivo negativo, enquanto pacientes com escore de 7 a 10 apresentam alto risco com uma taxa de eventos superior a 50% em 6 semanas.^103^^-^^106^

Quando comparado ao escores GRACE e TIMI, o escore HEART demonstrou maior habilidade em distinguir pacientes de baixo risco para eventos cardíacos maiores, com menor taxa de perda e maior acurácia para a estratificação de risco inicial na sala de emergência.^107^^,^^108^

A crescente utilização conjunta dos dados clínicos, eletrocardiográficos e mensuração de troponina ultrassensível e a sistematização da utilização dos escores amplificaram a eficácia dos protocolos de avaliação diagnóstica e a estratificação de risco da dor torácica na sala de emergência, promovendo alta precoce com segurança e redução de exames adicionais em 30 dias.^109^

Estudo que comparou de forma randomizada a aplicação do escore HEART no fluxo de atendimento emergencial de pacientes com dor torácica verificou um elevado valor preditivo negativo para ocorrência de eventos cardiovasculares maiores no primeiro ano, sem diferenças observadas quanto à internação hospitalar ou readmissão em serviço de emergência.^110^

Neste sentido, o atual impacto da avaliação adicional rotineira por exames de imagem em paciente classificados como baixo risco tem sido reconsiderado, pois, apesar do reconhecido valor quanto à redução do tempo para o diagnóstico, evidências atuais identificaram que essa estratégia pode proporcionar um aumento de exames adicionais sem benefício verificado quanto à ocorrência de IAM ou eventos clínicos relevantes.^111^

#### 5.5. Atuação da Enfermagem no Protocolo de Dor Torácica

A triagem hospitalar, quando realizada por enfermeiros habilitados, melhora a identificação de pacientes de maior risco, assim como reduz o tempo de realização do ECG.^112^ Já foi demonstrado que, em hospitais de zona rural, enfermeiros emergencistas apresentaram alto nível de acurácia, reduzindo tempo de espera e tempo de permanência sem comprometer a segurança dos pacientes.^113^ Dessa forma, a participação ativa da enfermagem na triagem de pacientes com dor torácica deve ser estimulada, assim como o treinamento constante da equipe multidisciplinar na abordagem do paciente com dor torácica.

**Figura 1.5:**
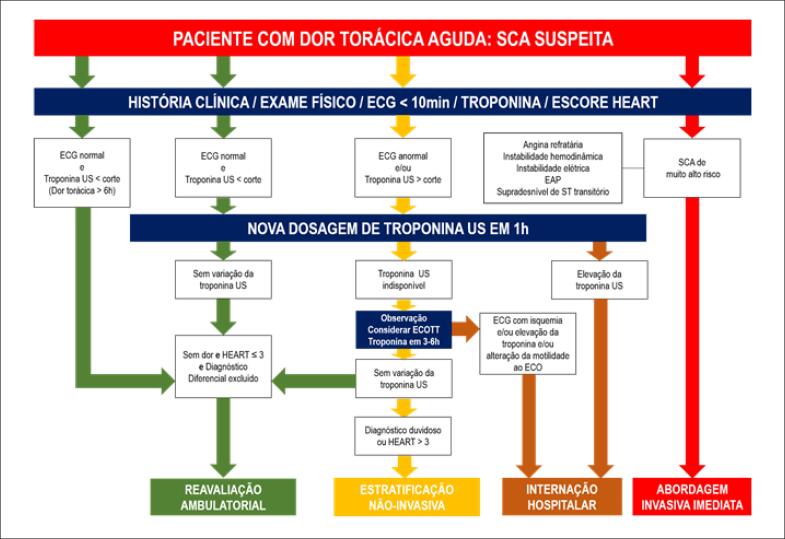
Fluxograma de rotina diagnóstica do paciente com dor torácica aguda na emergência. US: ultrassensível; ECOTT: ecocardiograma transtorácico.

O fluxograma proposto para abordagem do paciente com dor torácica na emergência está exposto na Figura 1.5.

**Table:** 

Rotina diagnóstica e critérios de internação - Sumário de recomendações e evidências		
A triagem inicial deve ser realizada com base em história clínica, exame físico, ECG de 12 derivações em até 10min e troponina.	I	A
O escore HEART deve ser utilizado para estratificação de risco e auxílio na decisão de alta hospitalar precoce.	I	B
Pacientes com escore HEART ≤ 3 associado à troponina em tempo hábil negativa, ECG sem alteração isquêmica e ausência de antecedentes de DAC podem ser liberados do serviço de emergência com segurança para reavaliação ambulatorial.	I	B
Triagem hospitalar realizada por enfermeiros habilitados, visando ao reconhecimento precoce de pacientes sob maior risco.	IIa	B
Uso de escore EDACS e ADAPT para estratificação de risco clínico como opção ao escore HEART.	IIa	B
Utilização do escore HEART visando à liberação precoce em pacientes atendidos por ambulância.	IIb	C

### 6. Condutas na Emergência após Estratificação de Risco

#### 6.1. Indicações de Estratégia Invasiva Emergencial

A estratégia invasiva emergencial está indicada em situações de muito alto risco de morte (Tabela 1.8). Esse perfil de pacientes geralmente não está amplamente representado na maioria dos estudos randomizados. Nesses pacientes, está indicada a intervenção coronariana em até 2h. Em centros onde não há serviço de cardiologia intervencionista disponível, esses pacientes devem idealmente ser transferidos.

**Table:** 

Estratégia invasiva de urgência - Sumário de recomendações e evidências		
A estratégia invasiva urgente/imediata está indicada em pacientes com SCASSST com angina refratária ou instabilidade hemodinâmica ou elétrica (sem comorbidades graves ou contraindicações para estes procedimentos).	I	A

**Tabela 1.8:** Indicação de estratégia invasiva de urgência

Instabilidade hemodinâmica ou choque cardiogênico
Dor torácica refratária ao tratamento medicamentoso
Arritmias malignas ou parada cardiorrespiratória
Complicações mecânicas do infarto
Insuficiência cardíaca aguda
Alterações recorrentes do segmento ST-T com elevação intermitente do segmento ST

#### 6.2. Tratamento inicial (sala de emergência/ambulância)

##### 6.2.1. Oxigênio Suplementar

Hipoxemia associada à isquemia miocárdica pode ocorrer devido a alterações da relação ventilação-perfusão, secundárias a *shunt* arteriovenoso pulmonar (consequente ao aumento da pressão diastólica final do ventrículo esquerdo) e, além disso, formação de edema intersticial e/ou alveolar pulmonar. A hipoxemia, por sua vez, agrava a isquemia miocárdica, aumentando a lesão miocárdica. A mensuração da saturação de O2 (SaO2) através de oximetria digital deve ser realizada desde o atendimento pré-hospitalar e/ou na ambulância, visando ao diagnóstico precoce da hipoxemia.

A administração de oxigenioterapia suplementar em pacientes com IAM está indicada quando o paciente apresentar hipóxia com SaO2 < 90% ou sinais clínicos de desconforto respiratório.^114^ No entanto, a administração de oxigenioterapia a pacientes com suspeita de SCA e SaO2 ≥ 90% não se associou à redução de mortalidade ou a outros desfechos cardiovasculares em 1 ano de seguimento, em um estudo randomizado que incluiu mais de 6 mil pacientes.^115^

A oxigenoterapia deve ser cuidadosa para não eliminar o estímulo respiratório hipóxico na presença de doença pulmonar obstrutiva crônica ou de outras causas de hipercapnia. Pacientes com congestão pulmonar, cianose, hipoxemia arterial comprovada ou insuficiência respiratória associadas devem receber suplementação de oxigênio e ser cuidadosamente acompanhados com gasometrias seriadas. Em casos selecionados que evoluem com sinais de insuficiência respiratória, com persistência de congestão pulmonar e hipoxemia, o suporte ventilatório não invasivo (VNI) deve ser instituído, além de progressão para ventilação invasiva na ocorrência de choque circulatório, falência da VNI e nos pacientes instáveis em transição para revascularização miocárdica urgente (ICP ou CRVM). A administração desnecessária de oxigênio por tempo prolongado pode causar vasoconstrição sistêmica e até mesmo ser prejudicial.

**Table:** 

Oxigenioterapia - Sumário de recomendações e evidências		
Oxigenioterapia (2 a 4L/min) em pacientes com risco intermediário e alto, na presença de SaO2 < 90% e/ou sinais clínicos de desconforto respiratório.	I	C

##### 6.2.2. Analgesia e Sedação

A dor precordial e a ansiedade costumeiramente associada, presentes nas SCA, geralmente levam à hiperatividade do sistema nervoso simpático. Esse estado hiperadrenérgico, além de aumentar o consumo miocárdico de oxigênio, predispõe ao aparecimento de taquiarritmias atriais e ventriculares. A terapêutica antianginosa inicial deve ser realizada com betabloqueadores e nitratos, desde que não haja contraindicações, como choque cardiogênico e/ou hipotensão. O sulfato de morfina poderá ser utilizado em casos refratários ou com contraindicação aos nitratos ou betabloqueadores. Deve ser administrado por via intravenosa, na dose de 2 a 4mg diluídos a cada 5min até, no máximo, 25mg. A administração em pequenos incrementos tem por objetivo evitar efeitos adversos como hipotensão e depressão respiratória. Deve-se evitar derivados da morfina, a não ser em casos de hipersensibilidade a esta, que, nessa situação (de hipersensibilidade), pode ser substituída pelo sulfato de meperidina em doses fracionadas de 20 a 50mg IV.

Apesar de seu potente e eficaz efeito no controle da angina, há evidência de que o uso da morfina reduza o efeito antiplaquetário dos inibidores do receptor P2Y_12_, tanto o clopidogrel^116^ quanto antiplaquetários mais potentes como prasugrel e ticagrelor.^117^^,^^118^

Do ponto de vista de eventos clínicos, uma subanálise do registro CRUSADE^119^ e, mais recentemente, uma metanálise que incluiu quase 70 mil pacientes demonstraram que o uso rotineiro precoce de morfina pode estar associado a aumento de mortalidade intra-hospitalar e eventos cardiovasculares maiores.^120^ Em uma análise *post-hoc* do estudo EARLY-ACS, verificou-se que a morfina se associou a maior risco de eventos isquêmicos precoces quando utilizada concomitantemente ao pré-tratamento com clopidogrel. Porém, no grupo de pacientes sem uso de clopidogrel antes do cateterismo, não se verificou piora de desfecho clínico com uso de morfina.^121^

O emprego rotineiro de ansiolíticos tem sido prática comum em nosso meio. Parece muitas vezes ser dispensável, devendo ser reservado para situações especiais. Estudo clínico randomizado, duplo-cego, envolvendo 131 pacientes do gênero masculino com IAM observou que o grau de ansiedade, pressão arterial, frequência cardíaca e desconforto precordial não diferiu, quer os pacientes tenham sido tratados com diazepam ou com placebo^122^. Os derivados diazepínicos têm sido os mais utilizados nessa indicação.

Anti-inflamatórios não esteroides (AINE) não devem ser utilizados (com exceção do AAS) para controle da dor em pacientes com IAM, pois aumentam o risco de eventos cardiovasculares maiores. Se o paciente estiver em uso prévio de AINE, estes devem ser suspensos durante a internação devido aos efeitos deletérios associados a elevação da pressão arterial, risco de lesão renal aguda e hiperviscosidade sanguínea com piora do prognóstico cardiovascular.^123^

**Table:** 

Analgesia e sedação - Sumário de recomendações e evidências		
Administrar sulfato de morfina em pacientes que mantêm dor contínua, apesar de terapia anti-isquêmica otimizada.	IIb	B
Administrar benzodiazepínicos em pacientes com sinais e sintomas de ansiedade.	IIb	C
Anti-inflamatórios não esteroides (AINE) não devem ser administrados (com exceção do AAS) em pacientes com suspeita de IAM.	III	

##### 6.2.3. Controle Glicêmico

Mais de 30% dos pacientes admitidos com IAM apresentam diabetes melito ou desconhecem o diagnóstico de diabetes. Tais indivíduos apresentam maiores taxas de sangramento e pior prognóstico em 30 dias, quando comparados com pacientes com níveis glicêmicos normais.^124^^-^^127^ Nesse sentido, protocolos de controle glicêmico devem ser instituídos para pacientes com IAM que apresentem hiperglicemia significativa (> 180mg/dL). O alvo dessa terapia é reduzir os níveis glicêmicos e evitar episódios de hipoglicemia (< 70mg/dL), que podem causar diversos efeitos deletérios incluindo a expansão da área de IAM. Em pacientes com maior risco de hipoglicemia, tais como idosos, nefropatas e pacientes com efeito residual de hipoglicemiantes orais e/ou em jejum, o controle glicêmico deve ser ajustado para tolerar níveis glicêmicos um pouco mais altos e, assim, prevenir hipoglicemia.

**Table:** 

Controle glicêmico - Sumário de recomendações e evidências		
É recomendável mensurar, na admissão, os níveis glicêmicos de todos os pacientes com suspeita de SCA, assim como monitorar evolutivamente a glicemia dos pacientes diabéticos ou que apresentem hiperglicemia durante a internação.	I	C
Controle glicêmico com protocolos de utilização de insulina intermitente, devem ser considerados em pacientes com níveis glicêmicos > 180mg/dL, com cautela, para evitar episódios de hipoglicemia.	IIa	C
Em pacientes com maior risco de hipoglicemia, tais como idosos, nefropatas e pacientes com efeito residual de hipoglicemiantes orais e/ou em jejum, o controle glicêmico deve ser ajustado para tolerar níveis glicêmicos um pouco mais altos e, assim, prevenir hipoglicemia.	IIa	C

##### 6.2.4. Terapia anti-isquêmica

O objetivo da terapêutica anti-isquêmica é reduzir o consumo de oxigênio (diminuindo a frequência cardíaca, a pressão arterial e a contratilidade miocárdica) ou aumentar a oferta de oxigênio (administrando oxigênio ou promovendo vasodilatação).

###### 6.2.4.1. Nitratos

Os benefícios terapêuticos dos nitratos estão relacionados aos seus efeitos na circulação periférica e coronária. O seu efeito venodilatador, diminuindo o retorno venoso ao coração e o volume diastólico final do ventrículo esquerdo, reduz o consumo de oxigênio pelo miocárdio. Adicionalmente, observam-se efeitos de vasodilatação de artérias coronárias, normais ou ateroscleróticas, redirecionamento de fluxo intercoronário, com aumento da circulação colateral e inibição da agregação plaquetária. Além do efeito sintomático, os nitratos agem reduzindo a congestão pulmonar, principalmente pela redução do retorno venoso sistêmico.

Não existem estudos clínicos controlados que tenham testado os efeitos dos nitratos em desfechos clínicos e mortalidade na SCASSST, embora seu uso seja universalmente aceito. Os estudos em AI que os avaliaram foram pequenos e do tipo observacional.^128^^-^^132^ A melhora dos sintomas tanto anginosos como congestivos está relacionada ao uso dos nitratos, porém sem impacto quanto a mortalidade, infarto ou necessidade de revascularização.

O tratamento é iniciado na sala de emergência, administrando-se o nitrato por via sublingual (nitroglicerina, mononitrato ou dinitrato de isossorbida). Caso não haja alívio rápido da dor, esses pacientes podem se beneficiar com a administração intravenosa (nitroglicerina e mononitrato de isossorbida são os disponíveis em nosso meio).

Os nitratos estão contraindicados na presença de hipotensão arterial importante (pressão arterial sistólica [PAS] < 100mmHg) ou uso prévio de sildenafil nas últimas 24h, ou uso de tadalafila nas últimas 48h. Um efeito colateral comum é a cefaleia. O uso sublingual de nitroglicerina (0,4mg/comp.), dinitrato de isossorbida (5mg/comp.) ou mononitrato de isossorbida (5mg/comp.) não deve ultrapassar 3 comprimidos, separadas as administrações por intervalos de 5 min. A nitroglicerina IV é empregada na dose de 10*µ*g/min com incrementos de 10*µ*g a cada 5min até obter-se melhora sintomática ou redução da pressão arterial (queda da PAS não deve ser superior a 30% ou PAS não atingindo < 110mmHg), ou então aumento da frequência cardíaca (> 10% da basal). É de se esperar o aparecimento de tolerância aos efeitos hemodinâmicos do medicamento após 24h de uso. O tratamento intravenoso deverá ser mantido por 24 a 48h da última dor anginosa e sua suspensão deverá ser feita de forma gradual.

**Table:** 

Nitratos - Sumário de recomendações e evidências		
Uso de nitrato sublingual para alívio da angina.	I	C
Uso de nitrato endovenoso para controle de angina persistente, hipertensão arterial ou sinais de congestão.	I	C

###### 6.2.4.2. Betabloqueadores

Assim como com os nitratos, a experiência clínica controlada do emprego de betabloqueadores na AI é limitada. A evidência de efeitos benéficos baseia-se em seu mecanismo de ação, em estudos clínicos controlados de pequeno porte, e na extrapolação de resultados de estudos em angina estável e IAM com supra de ST. Os betabloqueadores inibem competitivamente os efeitos das catecolaminas circulantes. Na AI, seus benefícios estão relacionados com sua ação nos receptores beta-1. Diminuem a frequência cardíaca, a pressão arterial e a contratilidade miocárdica, provocando redução do consumo de oxigênio pelo miocárdio. Apesar de a inexistência de estudos randomizados em larga escala avaliando a ação sobre desfechos clínicos graves como mortalidade em pacientes com SCASSST, esses fármacos, juntamente com os nitratos, são considerados agentes de primeira escolha no tratamento das SCA na sala de emergência para pacientes sem contraindicação. Na AI, foram poucos e de pequeno porte os estudos que compararam betabloqueadores com placebo.^133^^-^^135^ Apesar da ausência de efeito redutor de mortalidade, metanálise de cinco pequenos estudos^136^ avaliando a utilização da terapêutica betabloqueadora em 4.700 pacientes com AI mostrou redução de 13% no risco relativo de progressão para IAM. Estudo nacional em pacientes com SCASSST encontrou associação significativa entre o uso de betabloqueador oral com início nas primeiras 24h de hospitalização e menor mortalidade intra-hospitalar, independentemente da presença ou não de disfunção ventricular esquerda.^137^

Apesar de realizado em pacientes com IAM com supradesnível do segmento ST, o estudo COMMIT^138^ sugere que a utilização rotineira de dose elevada de betabloqueador IV seguido de administração oral pode aumentar a incidência de choque cardiogênico, principalmente quando utilizado nas primeiras 24 a 48h de evolução, e em pacientes com quadro clínico de disfunção ventricular esquerda ou com fatores de risco para choque cardiogênico (idade superior a 70 anos, frequência cardíaca > 110bpm/min ou pressão sistólica < 120mmHg). Apesar de não haver estudo clínico randomizado específico para população com SCASSST, estudo de banco de dados com mais de 20 mil pacientes mostra comportamento semelhante ao observado no estudo COMMIT.^139^

Assim, recomenda-se o uso rotineiro de betabloqueador oral nos pacientes sem contraindicação (mais detalhes na Parte 2 desta Diretriz). Caso o paciente apresente dor isquêmica persistente e/ou taquicardia (não compensatória de um quadro de insuficiência cardíaca), pode-se utilizar a formulação venosa. São usados vários regimes terapêuticos na dependência do betabloqueador selecionado. Não existem evidências de superioridade de um betabloqueador sobre outro. Em pacientes que evoluem com disfunção ventricular (FEVE < 40%) o emprego de betabloqueadores é muito recomendado (carvedilol, bisoprolol e succinato de metoprolol) em pacientes sem congestão pulmonar.

A seguir, há uma relação das doses de metoprolol e atenolol, os mais usados em nosso país com essa indicação.

Metoprolol: IV – 5mg (1 a 2min) a cada 5min até completar a dose máxima de 15mg.

VO – 50 a 100mg a cada 12h, iniciado 15min após a última administração IV.

Atenolol: IV – 5mg (1 a 2min) a cada 5min até completar a dose máxima de 10mg.

VO – 25 a 50mg a cada 12h, iniciado 15min após a última administração IV.

Durante a administração intravenosa, deverão ser monitorados, cuidadosamente, a frequência cardíaca, a pressão arterial, o ECG e a ausculta pulmonar.

Betabloqueadores não devem ser utilizados em pacientes com sintomas relacionados a vasospasmo por uso de cocaína durante intoxicação aguda. Em pacientes com asma ou doença pulmonar obstrutiva crônica, são contraindicados apenas na vigência de broncospasmo – nessa situação, betabloqueadores beta-1 seletivo são preconizados.

**Table:** 

Betabloqueadores - Sumário de recomendações e evidências		
Administrar betabloqueadores VO nas primeiras 24h em pacientes sem contraindicações (sinais de insuficiência cardíaca, sinais de baixo débito, risco aumentado de choque cardiogênico ou outras contraindicações ao betabloqueador).	IIa	B
Administrar betabloqueador IV em pacientes de risco intermediário e alto com isquemia persistente, taquicardia e hipertensão, desde que não apresente sinais clínicos / radiológicos de insuficiência cardíaca.	IIb	B
Administrar betabloqueador IV em pacientes com fatores de risco para choque cardiogênico.	III	A

##### 6.2.5. Terapia Antitrombótica Inicial (Sala de Emergência/Ambulância)

###### 6.2.5.1. Antiplaquetários Orais

O fenômeno de instabilidade da placa aterosclerótica coronariana consequente à ruptura ou erosão, com subsequente ativação plaquetária e trombose, é um reconhecido mecanismo de deflagração das SCA.^140^

Esse conceito direcionou os estudos e avanços na terapia antiplaquetária e o conceito atual da dupla terapia com AAS e um fármaco inibidor de receptor plaquetário P2Y_12_ em pacientes com SCA, contemplando os alocados para estratégia invasiva e/ou proposta inicial de tratamento clínico.^141^

As características e evidências científicas para escolhas individualizadas dos fármacos antiplaquetários encontram-se detalhadamente descritas na Parte 2. No entanto, uma das importantes decisões na sala de emergência consiste no momento ideal para o início de um inibidor de P2Y_12_, particularmente em pacientes submetidos à estratégia invasiva precoce com cinecoronariografia e perspectiva de ICP. O termo “pré-tratamento” geralmente se refere ao início de um inibidor de P2Y_12_ antes da definição da anatomia coronariana, seja na ambulância ou na sala de emergência.

####### 6.2.5.1.1. Fundamentos do Pré-tratamento com Bloqueadores de P2Y_12_

O objetivo do pré-tratamento com segundo antiplaquetário potente na SCASSST seria a possibilidade de alcançar inibição plaquetária eficaz e prevenção de maior agregação, sobretudo preparando e protegendo o paciente para estratégia invasiva, potencialmente reduzindo a extensão da trombose, risco de IAM recorrente e minimizando o risco de complicações relacionadas à ICP como trombose de *stent* e infarto periprocedimento.^142^

Em contrapartida, a proposta de pré-tratamento tem reconhecido impacto em aumentar o risco de sangramento associado ou não à cirurgia de revascularização miocárdica, fator que está associado a um relevante aumento da morbimortalidade na SCA.^143^^-^^145^

Uma questão importante se refere ao tempo mínimo ideal para ação clinicamente eficaz do fármaco, sabendo que até mesmo os mais potentes agentes antiplaquetários orais eficazes requerem pelo menos 30 a 60min para inibição plaquetária efetiva e, assim, obter “pré-tratamento apropriado” antes de cateterismo e perspectiva de ICP.^146^^-^^149^

A utilização do segundo agente plaquetário seguindo a proposta de pré-tratamento com bloqueadores de P2Y_12_ foi adotada nos estudos CURE (clopidogrel)^150^^,^^151^ e PLATO (ticagrelor);^152^^,^^153^ no estudo TRITON, o prasugrel poderia ser administrado somente após a identificação da anatomia coronariana.^154^

####### 6.2.5.1.2. Estudos Comparativos de Pré-tratamento versus Utilização do Segundo Antiplaquetário na Sala de Cateterismo

O estudo PRELOAD ARMIDA-5 avaliou o pré-tratamento com clopidogrel em pacientes com SCASSST (n = 409) ou angina estável e não verificou associação com redução de eventos cardiovasculares adversos.^155^

O estudo CREDO foi planejado para avaliar o benefício de início do pré-tratamento com clopidogrel, além de AAS, em pacientes submetidos à ICP. Ensaio este controlado, no qual 2.116 pacientes, sendo 39% com SCA e 61% com DAC estável, receberam 300mg de clopidogrel (n = 1.053) ou placebo (n = 1.063), 3 a 24h antes da ICP. O tempo médio entre a dose de ataque do medicamento de estudo e a ICP foi de 9,8h, dos quais 51% recebem a dose de ataque entre 3 e 6h e os 49% restantes, entre 6 e 24h antes da ICP. O estudo não encontrou redução estatisticamente significativa no desfecho combinado de morte, infarto ou revascularização urgente do vaso-alvo em 28 dias. No entanto, quando a população foi analisada com base no tempo de intervalos de tratamento de 3 a 6h, 6 a 12h e 12 a 24h antes da ICP, observou-se interação significativa em que não houve benefício de pré-tratamento se a dose de clopidogrel tiver sido administrada < 6h antes da ICP, mas houve redução relativa de 35,5% e 40,1% no desfecho final combinado, respectivamente, em grupos que receberam clopidogrel entre 6 e 12h e 12 e 24h antes da ICP, respectivamente. Esses achados corroboram a hipótese da necessidade de maior tempo de ação quando utilizado clopidogrel em proposta de pré-tratamento.^156^

O ensaio clínico ACCOAST (n = 4.033) foi um estudo controlado multicêntrico e randomizado que comparou o tratamento com 60mg de prasugrel administrado na sala de cateterismo no momento da intervenção coronária percutânea com a estratégia de utilização de pré-tratamento com 30mg de prasugrel e uma segunda dose de 30mg durante a angiografia coronariana dentro de 2 a 48h após a randomização.^157^

O desfecho final composto primário foi morte por causas cardiovasculares, IAM, AVC, revascularização urgente ou necessidade de terapia de resgate com inibidores de glicoproteína IIb/IIIa até o sétimo dia. ICP foi realizada em 69% dos pacientes em um tempo médio de 4,3h após a dose inicial de ataque, e apenas 9% dos pacientes incluídos no ensaio ACCOAST tiveram um escore GRACE > 140. A incidência do desfecho final primário até o sétimo dia e aos 30 dias não diferiu significativamente entre o grupo que recebe pré-tratamento com prasugrel *versus* o grupo placebo (10,0% *vs.* 9,8%, HR 1,02, IC 95% 0,84-1,25, p = 0,81). Foi documentada maior taxa de sangramento maior relacionado ou não à CRVM com a estratégia de pré-tratamento (HR, 1,90; IC 95%, 1,19-3,02; p = 0,006). As taxas de sangramento maior (TIMI) e sangramento ameaçador de vida não relacionado à CRVM foram 3 e 6 vezes maiores, respectivamente. Metade de todos os episódios foi relacionada com cirurgia de revascularização urgente, e metade dos eventos de sangramento maior não relacionados à CRVM foi causada por sangramento vascular no local de acesso.

O ensaio clínico randomizado DUBIUS (Downstream versus Upstream administration of P2Y_12_ receptor Blockers In non-ST elevated acUte coronary Syndromes) avaliou 1.449 pacientes com SCASSST e coronariografia programada em até 72h, comparando a estratégia de pré-tratamento com ticagrelor *versus* a utilização do segundo antiplaquetário prasugrel ou ticagrelor (randomização 1:1) administrados na sala de hemodinâmica nos pacientes com indicação de intervenção coronária percutânea.^158^ A população estudada exibia perfil de risco isquêmico moderado (escore GRACE médio = 122) e risco hemorrágico não elevado (escore CRUSADE médio = 22). Análise provisória pré-especificada para futilidade orientou a interrupção precoce do estudo. A taxa do desfecho primário em 30 dias, composto de morte devido a causas vasculares, infarto não fatal ou AVC não fatal e fenômenos hemorrágicos relevantes (BARC tipo 3, 4 e 5), não diferiu significativamente entre a estratégias (2,9% e 3,3%, respectivamente, para não pré-tratar *vs.* pré-tratamento). Esse achado foi consistente no subgrupo que realizou ICP (72% da população), e o tempo médio de realização de ICP foi de 23,3h, notadamente superior ao estudo ACCOAST (4h); os achados, no entanto, foram semelhantes naqueles que realizaram coronariografia após 24h. Esse estudo teve a originalidade de metade da população alocada para não pré-tratamento ter utilizado ticagrelor, porém sem poder para comparação de fármacos. Apesar das limitações de análise por interrupção prematura, representa uma observação de não benefício da estratégia de pré-tratamento na SCASSST com manuseio invasivo, porém salienta a possibilidade de escolha individualizada de fármacos na sala de hemodinâmica.

####### 6.2.5.1.3. Dados de Pré-tratamento em Metanálises

Metanálise que contemplou 16 estudos, incluindo 61.517 pacientes com SCA com ou sem supradesnível do segmento ST, verificou incidência significativamente menor de MACE no subgrupo com SCASSST alocado para pré-tratamento com clopidogrel (OR = 0,83; IC 95% 0,71-0,96, p = 0,01), sem aumento de sangramento maior em pacientes submetidos à ICP em até 48h.^159^

Outra metanálise que incluiu 23 estudos e 60.907 pacientes teve o objetivo de comparar a eficácia e a segurança dos inibidores plaquetários P2Y_12_: clopidogrel, ticagrelor e prasugrel administrados em dois cortes de tempo diferentes em relação à ICP: precoce (< 2h pré-ICP) *versus* tardio (>2h pré-ICP ou pós-ICP). A análise de estudos que incluíram pacientes com SCASSST não demostrou benefício no pré-tratamento com ticagrelor ou prasugrel, mas prasugrel aumentou o risco de sangramento nessa população com SCASSST.^160^

Conforme exposto, não existe um claro benefício estabelecido para uso rotineiro do segundo agente antiplaquetário via oral na abordagem inicial da SCASSST, sobretudo no cenário de estratégia invasiva em caráter emergencial, quando o uso e a escolha do fármaco podem ser definidos na sala de cateterismo. Porém, o pré-tratamento pode ser considerado nos pacientes sem risco hemorrágico crítico, com risco isquêmico moderado ou elevado, com programação para realizar cinecoronariografia ou manuseio inicial conservador em terapia anti-isquêmica plena.

**Figura 1.6:**
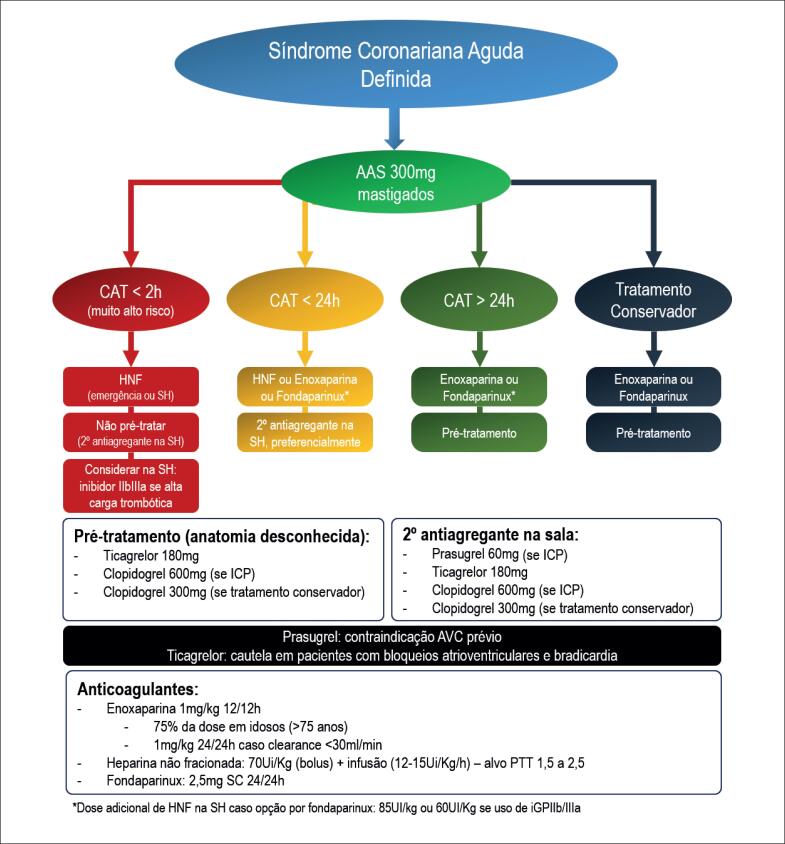
Terapia antitrombótica inicial nas síndromes coronarianas agudas sem supradesnível do segmento ST. CAT: cateterismo; HNF: heparina não fracionada; SH: sala de hemodinâmica

O desafio de equilibrar risco isquêmico com risco de sangramento nos conduz à necessidade de abordagem personalizada para decisão do momento de utilização e escolha do segundo antiplaquetário. Devemos considerar características clínicas, disponibilidade para realizar cinecoronariografia, dados de eficácia, segurança e propriedades farmacodinâmicas dos inibidores plaquetários P2Y_12_ disponíveis para uso no Brasil; contudo, importante salientar que, na decisão de pré-tratamento sem anatomia conhecida, está contraindicado o uso de prasugrel. Na decisão de administração na sala de cateterismo, todos os inibidores P2Y_12_ podem ser utilizados conforme escolha individualizada. Neste contexto, se aplica, na ausência de contraindicações, o uso preferencial de prasugrel ou ticagrelor, fármacos com rápido início de ação e maior eficácia antitrombótica, e a opção do clopidogrel na presença de risco hemorrágico muito elevado (Figura 1.6).

**Table:** 

Terapia antiplaquetária inicial - Sumário de recomendaçõese evidências		
O uso de AAS é recomendado na sala de emergência o mais precoce possível em todos pacientes sem contraindicação, em dose inicial de 150 a 300mg (pacientes sem uso prévio de AAS) e dose de manutenção de 75 a 100mg.	I	A
E recomendado o uso de um inibidor receptor P2Y_12_ em adição a AAS, exceto contraindicações ou elevado risco de sangramento.	I	A
Não realizar pré-tratamento com segundo antiplaquetário inibidor do receptor P2Y_12_ nos pacientes instáveis e/ou com risco elevado, indicados para estratégia invasiva de forma imediata, sendo sua utilização recomendada para sala de cateterismo, quando anatomia coronariana conhecida e ICP programada.	I	B
Não há indicação rotineira de se iniciar iP2Y_12_ como pré- tratamento em pacientes indicados para estratégia invasiva precoce (< 24h).	I	B
Prasugrel (dose de ataque) na sala de hemodinâmica somente após a realização da angiografia se a intervenção coronariana for programada em pacientes sem história de AVC ou AIT prévios.	I	B
Ticagrelor (dose de ataque), independentemente da estratégia inicial conservadora ou invasiva.	I	B
Clopidogrel (dose de ataque), independentemente da estratégia inicial (conservadora ou invasiva) na indisponibilidade ou contraindicação ao ticagrelor ou prasugrel.	I	B
Em pacientes alérgicos a AAS, está indicada monoterapia inicial com inibidor P2Y_12_ (uso preferencial de ticagrelor ou prasugrel).	I	C
Clopidogrel (dose de ataque), independentemente da estratégia inicial (conservadora ou invasiva) em pacientes de muito alto risco para sangramento ou necessidade de anticoagulação oral a longo prazo.	IIa	C
Estratégia de pré-tratamento com prasugrel	III	

###### 6.2.5.2. Antagonistas dos Receptores Glicoproteicos IIb/IIIa

A ativação dos receptores existentes na superfície das plaquetas, denominados glicoproteína (GP) IIb/IIIa, constitui-se no mecanismo final de agregação plaquetária, em consequência de alteração morfológica sofrida pelo receptor, que aumenta a sua afinidade para ligar-se à molécula de fibrinogênio, elemento que funciona como ponte de ligação entre duas plaquetas. Esses fármacos têm sido utilizados em situações clínicas com grande potencial de ativação plaquetária, como, por exemplo, ICPs complexas e complicações trombóticas como *no-reflow* após angioplastia.

O abciximab é um anticorpo monoclonal que atua como bloqueador não competitivo e irreversível dos receptores de GP IIb/IIIa. Tem meia-vida plasmática curta de 5 a 10min, e sua meia-vida biológica é de 6 a 12h após a injeção de um *bolus* isolado. Com essas doses terapêuticas, consegue-se o bloqueio de 80% a 90% dos receptores de superfície. Cinquenta por cento desses receptores ainda permanecem bloqueados 1 semana após sua utilização. A dose recomendada é de 0,25mg/kg em *bolus*, seguida de uma administração de 0,125*µ*g/kg durante 12h.

O tirofiban é um derivado sintético, não peptídio, de molécula pequena, que possui em sua estrutura molecular a sequência RGD (arginina-glicina-aspartato), sítio de reconhecimento das integrinas e presente nas proteínas adesivas do tipo fibrinogênio, fator von Willebrand e vetronectina, entre outras. O tirofiban também age competitivamente no receptor celular IIb/IIIa, impedindo sua ligação ao fibrinogênio. A dose recomendada é a de 0,4*µ*g/kg/min por 30min, seguida da dose de manutenção de 0,1*µ*g/kg/min por 12 a 24 horas após o procedimento (máximo de 96 horas). No caso de se iniciar a utilização do medicamento na sala de cateterismo, deve-se iniciar com a dose de 10*µ*g/kg administrada em *bolus* em 3min, seguida de 0,15*µ*g/kg/min.

Com relação à eficácia, o abciximab e o tirofiban apresentam resultados comparáveis quando o tirofiban é empregado com *bolus* elevado, de 25*µ*g/kg, porém o abciximab mostrou-se superior ao tirofiban quando este foi empregado com a dose de *bolus* habitual de 10*µ*g/kg.^161^^,^^162^

Genericamente, os bloqueadores GP IIb/IIIa tendem a aumentar o risco de hemorragia, e trombocitopenia é complicação rara, mas não negligível.

No contexto de pacientes com SCASSST submetidos à estratégia essencialmente “conservadora” (não realização rotineira de procedimentos intervencionistas precoces), os bloqueadores GP IIb/IIa tiveram seu uso fundamentado em estudos nos quais, além de heparinização, a ativação plaquetária era antagonizada, sistematicamente, apenas por AAS.^163^^-^^170^ Metanálise que incluiu > 30.000 pacientes demonstrou redução de 9% no risco relativo de óbito ou infarto aos 30 dias de seguimento (p = 0,015),^169^ sendo o benefício restrito aos pacientes de maior risco, fundamentalmente com troponina elevada e/ou com depressão do segmento ST e/ou submetidos à ICP (24% aos 30 dias de seguimento).

Diversos estudos testaram o papel dos bloqueadores GP IIb/IIIa no contexto de pacientes submetidos à ICP.^171^^-^^177^ Os resultados desses estudos são mais homogêneos, demonstrando invariavelmente benefício com o uso destes medicamentos, porém à custa de importante aumento na incidência de sangramento. Salienta-se que esses estudos foram desenvolvidos sem o uso rotineiro de tienopiridínico quando da chegada do paciente ao hospital. Metanálise que incluiu estudos que analisaram o papel dos bloqueadores IIb/IIIa em pacientes com SCA sem ou com supradesnível de ST demonstrou, além de diminuições significativas nas incidências de óbito ou (re)infarto (p < 0,0001), diminuição de 21% (IC de 95%: 0,64-0,67) no risco relativo de óbito aos 6 meses de seguimento.^178^

Em pacientes em uso de dupla antiagregação plaquetária com AAS + clopidogrel, a adição de abciximab foi comparada a placebo no estudo ISAR-REACT-2, que envolveu 2.022 pacientes com SCASSST. Nesse estudo, o emprego do abiciximab associou-se a menor incidência do desfecho composto de óbito, IAM e revascularização urgente (RR 0,75; IC 0,58-0,97; p = 0,03), sem aumento significativo na incidência de desfechos hemorrágicos graves ou não graves. Vale ressaltar que o benefício do emprego do abciximab só foi encontrado nos indivíduos que apresentavam elevação de troponina.^179^

O estudo ACUITY-TIMING incluiu 9.207 pacientes com SCASSST (98% com AAS e 65% com bloqueador P2Y_12_ pré-angiografia) e avaliou qual o melhor momento para o emprego do bloqueador GP IIb/IIIa, randomizando os pacientes para o emprego rotineiro de tirofiban ou eptifibatide quando da randomização do paciente ou de forma eletiva quando da realização de ICP, já na sala de hemodinâmica. Nesse estudo, o emprego rotineiro dos bloqueadores GP IIb/IIIa não foi capaz de reduzir desfechos isquêmicos, e aumentou de forma significativa a incidência de sangramento importante (RR = 0,80, p < 0,001).^180^

Resultados semelhantes foram encontrados no estudo EARLY-ACS, em que 9.492 pacientes com SCASSST foram randomizados para emprego de antiagregação plaquetária com epitifibatide iniciado antes do cateterismo versus uso somente em casos selecionados antes da angioplastia (a partir de aspectos angiográficos). Novamente, a estratégia de uso rotineiro do bloqueador da GP IIb/IIIa não foi capaz de reduzir complicações isquêmicas; contudo, resultou em aumento na incidência de sangramentos e necessidade de transfusões.^181^

Com base no conjunto dessas evidências, o emprego dos bloqueadores da GP IIb/IIIa, como um terceiro antiagregante plaquetário, deve ser reservado para pacientes que não apresentem alto risco hemorrágico e que tenham alto risco isquêmico/trombótico por critérios clínicos e angiográficos.

###### 6.2.5.3. Anticoagulantes

A terapia anticoagulante deve ser administrada o mais rápido possível em todos os pacientes com SCASSST, visto que a utilização desses compostos reduz as incidências de IAM e óbito, sendo empregada poucas horas após o diagnóstico.^182^

A escolha e o momento do anticoagulante são determinados por estratégia de tratamento (abordagem invasiva ou conservadora), gravidade da apresentação clínica e particularidades de cada serviço. Nos pacientes com proposta de tratamento conservador inicial, recomenda-se o uso preferencial de enoxaparina ou fondaparinux. As doses preconizadas estão disponíveis na Tabela 1.9. A enoxaparina não deve ser administrada em pacientes com *clearance* de creatinina < 15mL/min e o fondaparinux com *clearance* de creatinina < 20mL/min. Nos pacientes com *clearance* entre 15 e 30mL/min e nos obesos (IMC > 30kg/m^2^ ou peso > 100kg, o monitoramento do fator anti-Xa é recomendado nas diretrizes para o uso de enoxaparina, com a justificativa de minimizar o risco de doses excessivas e consequências hemorrágicas graves.^183^^-^^185^

**Tabela 1.9:** Dose dos anticoagulantes conforme função renal

Medicamento	Recomendação		
	Função renal normal ou IRC estágio 1-3 (*clearance* de creatinina ≥ 30mL/min/1,73m²)	IRC estágio 4 (*clearance* de creatinina entre 15 e 29mL/min/1,73m²)	IRC estágio 5 (*clearance* de creatinina < 15mL/min/1,73m²)
Heparina não fracionada (HNF)	Antes da coronariografia: 60 a 70UI/kg *bolus* IV (máx. 5.000UI) e infusão (12 a 15UI/kg/h) com alvo de PTT 1,5-2,5× controle Durante coronariografia: 70 a 100UI/kg IV em pacientes não anticoagulados ou 50 a 70UI/kg se uso concomitante com inibidores GPIIbIIIa	Sem ajuste de dose	Sem ajuste de dose
Enoxaparina	1mg/kg SC 12/12h > 75 anos: 75% da dose	1mg/kg 24/24h	Não recomendado
Fondaparinux	2,5mg SC 24/24h	Não recomendado se *clearance* < 20mL/min/1,73m²	Não recomendado

Nos obesos mórbidos, estudos de farmacodinâmica e dados observacionais identificaram a frequente necessidade de ajuste de doses para alcance de meta terapêutica (IMC > 40kg/m^2^) guiada pela dosagem do fator anti-Xa, sugerindo utilidade do monitoramento quando disponível.^186^^,^^187^

No entanto, na avaliação de obesos com SCA no registro norte-americano CRUSADE, o uso de enoxaparina em dose padrão (1mg/kg/peso) naqueles com peso > 150kg esteve associado a um maior risco de sangramento em comparação com subgrupo entre 120 e 150kg. (11,4% *vs.* 5,6%, p < 0,001). Nesses pacientes, o uso alternativo de heparina não fracionada (HNF) é uma opção.^188^^,^^189^

Nos pacientes alocados para estratégia invasiva, existe a opção de uso da enoxaparina, fondaparinux e HNF. No subgrupo de pacientes de muito alto risco, com proposta de cateterismo imediato, apesar da semelhante eficácia da enoxaparina e HNF demostrada no estudo SINERGY, escolha da HNF iniciada na emergência ou, preferencialmente, na sala de cateterismo, pode reduzir a chance da ocorrência de transição de enoxaparina para HNF (*cross over*) e o conhecido maior risco hemorrágico dessa prática.^190^

Nos demais pacientes, a escolha deve ser orientada por características de risco isquêmico e hemorrágico do paciente e na experiência do serviço.

O uso preferencial da enoxaparina *versus* HNF é sugerido por dados de revisão sistemática dos ensaios randomizados em todo o espectro das SCA, sendo demonstrado que a enoxaparina é mais eficaz que a HNF em prevenir o desfecho combinado de morte por qualquer causa ou sangramento grave em pacientes com SCASSST submetidos à ICP.^191^^-^^193^

Fondaparinux é uma opção segura conforme proposta inicial de tratamento não invasivo, e, se posteriormente indicado cateterismo, está recomendado o uso de HNF durante o procedimento pelo risco de trombose de cateter.^194^^,^^195^

Paciente em uso prévio de anticoagulantes orais de ação direta, em vigência de SCA, deve ser manejado com cautela visando não incrementar o risco hemorrágico. Não existem evidências se a anticoagulação parenteral ou ICP podem ser realizadas precocemente ou não. Em situações de urgência, a ICP deve ser realizada independentemente do momento de última dose do anticoagulante oral direto (DOAC). Nos pacientes com risco isquêmico mais baixo, o procedimento deve ser postergado. Em pacientes com função renal normal (*clearance* de creatinina > 50mL/min), o efeito do DOAC é reduzido após 24h da administração da última dose. Nos pacientes com disfunção renal, o prazo é de 48h. Assim, o paciente pode ser submetido à ICP com menor risco de sangramento. A anticoagulação parenteral está indicada em pacientes submetidos à ICP de urgência independentemente do horário da última dose de DOAC.^196^

Dentre as diversas estratégias no sentido de diminuir sangramento, a utilização da via radial quando da realização do estudo hemodinâmico/ICP é a que se mostrou mais efetiva, com evidências de redução de mortalidade nos pacientes com SCA.^197^

**Table:** 

Anticoagulantes - Sumário de recomendações e evidências		
Uso de HNF em pacientes com disfunção renal grave *(clearance* < 15mL/min).	I	A
Uso de enoxaparina até a revascularização, por 8 dias ou até a alta hospitalar.	I	A
Uso de fondaparinux por 8 dias ou até a alta hospitalar como alternativa à enoxaparina, especialmente no paciente de elevado risco hemorrágico.	I	B
Nos pacientes em uso de fondaparinux, administrar HNF 85UI/kg IV no momento da ICP ou 60UI/kg naqueles que estiverem recebendo inibidores da GP IIb/IIIa.	I	B
Uso de HNF em pacientes com peso > 150kg.	IIa	B
Uso de enoxaparina preferencialmente à HNF em pacientes com *clearance ≥* 15mL/min/1,73m^2^), a não ser que cirurgia de revascularização miocárdica esteja planejada para as próximas 24h.	IIa	B
Uso preferencial de HNF na emergencia em pacientes com estudo hemodinâmico de imediato.	IIa	C
Troca de heparinas (HNF e enoxaparina).	III	
Uso de enoxaparina em pacientes com *clearance* de creatinina < 15mL/min e peso > 150kg.	III	
Uso de fondaparinux em pacientes com clearance de creatinina < 20mL/min.	III	
Uso de fondaparinux isoladamente na ICP	III	

A escolha individualizada dos fármacos antiplaquetários e anticoagulantes e de evidências que fundamentam o melhor perfil de utilização na SCASSST será discutida de de forma detalhada na Parte 2 desta Diretriz.

## Parte 2 – Condutas Durante a Hospitalização

### 1. Internação e Alta da Unidade Coronária de Terapia Intensiva

Pacientes com SCASSST de risco intermediário e alto devem permanecer hospitalizados em unidade coronariana (UCO), sempre que possível, até que a conduta definitiva seja implementada. Após a efetivação de uma ICP, o paciente deverá retornar à UCO e permanecer de 12 a 24h, se não houver complicações. As complicações após a ICP são a ocorrência de desfechos graves como oclusão do vaso-alvo, necessidade de cirurgia de revascularização de emergência ou nova ICP não programada, angina recorrente e óbito. Nos casos em que a decisão for de revascularização miocárdica cirúrgica, o paciente deverá permanecer em ambiente hospitalar (unidade semi-intensiva ou no quarto) até que a cirurgia seja realizada. Na indicação de tratamento medicamentoso intravenoso, também deverá permanecer na UCO até que o paciente esteja estável e em condições de ser liberado com medicações por via oral.

### 2. Nitratos

Não existem estudos clínicos controlados que tenham testado os efeitos dos nitratos em desfechos clínicos e mortalidade na angina instável (AI), embora seu uso seja universalmente aceito. Os estudos em AI que os avaliaram foram pequenos e do tipo observacional.^128^^-^^130^^,^^198^ Estudo de Borzak et al. avaliou, em 410 pacientes, os efeitos da medicação antianginosa pré e intra-hospitalar (antagonista do cálcio, betabloqueador e nitrato) nos desfechos clínicos, mortalidade e infarto do miocárdio não fatal. Não houve benefício na evolução. Análise multivariada também não mostrou haver correlação com a incidência de morte, infarto não fatal ou angina recorrente.^199^

**Table:** 

Nitratos na fase hospitalar - Sumário de recomendações e evidências		
Uso de nitrato em pacientes com risco intermediário e alto que persistem sintomáticos ou para controle da pressão arterial.	I	C
Uso associado a hipotensão arterial, infarto do ventrículo direito e uso dos inibidores da fosfodiesterase sildenafil e vardenafila dentro de 24h e tadalafila dentro de 48h com a dose de 20mg.	III	A

### 3. Betabloqueadores Adrenérgicos

A experiência clínica com os betabloqueadores é limitada a estudos clínicos controlados de pequeno porte e na extrapolação de resultados em pacientes com AI e síndrome coronariana com supradesnível de ST (SCACSST). Apesar da inexistência de estudos randomizados em larga escala, são amplamente utilizados, como os nitratos. Mais detalhes sobre esta classe são mencionados na Parte 1 desta Diretriz.

### 4. Antagonistas dos canais de cálcio

Os antagonistas dos canais de cálcio constituem um grupo heterogêneo de fármacos que têm em comum uma ação vasodilatadora coronária e cronotrópica negativa (para alguns agentes da classe). A ação anti-isquêmica se deve à redução do influxo de cálcio pela membrana celular, reduzindo a contratilidade miocárdica e o tônus vascular, a velocidade de condução atrioventricular (AV) e a atividade do nó sinusal.

Esses agentes se diferenciam em relação à sua capacidade de produzir vasodilatação arterial, reduzir a contratilidade miocárdica e retardar a condução AV. Os efeitos benéficos nas SCASSST se devem a uma combinação das suas ações, diminuindo o consumo miocárdico de oxigênio, a pós-carga, a contratilidade miocárdica e a FC, além de aumentarem a oferta de oxigênio ao miocárdio, pela vasodilatação coronária que promovem. A vasodilatação coronária é semelhante e independe do agente usado. Os di-hidropiridínicos ocasionam maior vasodilatação arterial periférica e tendem a produzir taquicardia reflexa (mais evidente com a nifedipina de ação curta); o verapamil e o diltiazem tendem a causar bradicardia por reduzirem o cronotropismo e o dromotropismo, podendo levar a bloqueios atrioventriculares (mais evidente com verapamil). Em pacientes com comprometimento da função ventricular esquerda e/ou alterações na condução AV, esses medicamentos devem geralmente ser evitados. Outro efeito colateral, mas que costuma aparecer apenas após uso prolongado desses agentes, é a retenção hídrica.

Para controlar os sintomas,^200^^,^^201^ os antagonistas dos canais de cálcio são tão eficientes quanto os betabloqueadores; entretanto, não reduzem a incidência de angina refratária, infarto ou óbito – ao contrário, parecem acentuar a incidência dessas complicações, como sugerido por metanálise.^202^ Até o momento, foram avaliados na AI apenas os representantes de primeira geração. Essas ações deletérias foram observadas com todas as classes de antagonistas do cálcio^135^^,^^136^^,^^203^ testados nessa indicação, mas inexistem dados conclusivos em relação aos di-hidropiridínicos. Por outro lado, em pacientes com IAMSSST, existem evidências de que o diltiazem e o verapamil (que não se associam à taquicardia reflexamente induzida) possam ter efeito protetor.^204^^,^^205^ Esses agentes podem ser usados para controlar sintomas isquêmicos refratários em pacientes já em uso de nitratos e betabloqueadores em doses adequadas, ou em pacientes que não toleram o uso destes medicamentos (principalmente nos casos de contraindicação), ou, ainda, nos casos de angina variante (Prinzmetal). Contudo, não é recomendado o emprego rotineiro de antagonistas dos canais de cálcio, sendo contraindicado, em particular, o uso isolado da nifedipina de ação rápida.

**Table:** 

Antagonistas dos canais de cálcio - Sumário de recomendações e evidências		
Pacientes com risco intermediário e alto. Uso de derivado não di-hidropiridínico em casos de contraindicação aos betabloqueadores e sem disfunção ventricular esquerda.	I	B
Pacientes com angina variante (Prinzmetal).	I	B
Di-hidropiridínicos de ação prolongada na presença de isquemia refratária para pacientes em uso adequado de nitratos e betabloqueadores e sem disfunção ventricular.	IIa	B
Derivados di-hidropiridínicos de início de ação rápida para pacientes de alto risco, já em uso adequado de betabloqueadores.	IIb	B
Derivados di-hidropiridínicos de início de ação rápida em pacientes sem uso adequado de betabloqueadores.	III	B

### 5. Antiplaquetários

#### 5.1. Ácido Acetilsalicílico

O AAS é o antiplaquetário de excelência, devendo ser sempre prescrito, exceto em casos de intolerância ou eventos adversos.^206^^-^^209^

Sua importância no tratamento das SCASSST está embasada, fundamentalmente, em estudos publicados nos anos 1980,^210^^,^^211^ que, em sua maioria, dispõem de um número relativamente pequeno de pacientes, com baixa incidência de desfechos, mas que, de forma geral, evidenciaram nítido impacto tanto na redução de IAM não fatal quanto na mortalidade nos curto e médio prazos.

A posologia preconizada do AAS é de 150 a 300mg para a dose de ataque, seguida por 75 a 100mg ao dia para a dose de manutenção. O estudo CURRENT OASIS-7 testou, em um de seus braços, a hipótese do uso de dose alta de manutenção do AAS em pacientes com SCA (cerca de 70% de pacientes sem supra de ST). Não houve diferença entre a dose de manutenção habitual (75 a 100mg ao dia) e a dose elevada (300 a 325mg/dia) na ocorrência de eventos cardiovasculares graves (mortalidade, IAM não fatal ou AVC, p = 0,61). Também não houve diferença em relação à ocorrência de sangramentos graves (p = 0,90).^212^

#### 5.2. Inibidores do Receptor P2Y_12_ (iP2Y_12_)

Os derivados tienopiridínicos (clopidogrel e prasugrel) são fármacos antagonistas da ativação plaquetária mediada pelo difosfato de adenosina (ADP), que agem bloqueando de maneira irreversível o receptor P2Y_12_ plaquetário; e também reduzem o nível de fibrinogênio circulante e bloqueiam parcialmente os receptores de glicoproteína IIb/IIIa, dificultando sua ligação ao fibrinogênio e ao fator de von Willebrand. Em conjunto com o ticagrelor (derivado da ciclopentiltriazolopirimidina – “não tienopiridínico”), que é um inibidor reversível da agregação plaquetária induzida por ADP, estes são, atualmente, os três iP2Y_12_ plaquetários disponíveis para uso no Brasil (preferencialmente em associação ao AAS).

##### 5.2.1. Clopidogrel

No contexto de SCASSST, o primeiro grande estudo clínico – CURE (Clopidogrel in Unstable angina to prevent Recurrent Events) – testou o papel do clopidogrel em adição ao AAS em mais de 12 mil pacientes, sendo acompanhados por 3 a 12 meses (média de 9 meses). Ao final do seguimento, houve redução de 20% (RR 0,80; IC 95% 0,72-0,89; p = 0,00005) na incidência dos eventos (óbito cardiovascular, IAM ou AVC) a favor do grupo clopidogrel + AAS em relação ao grupo AAS + placebo, à custa de um aumento na incidência de sangramentos no grupo clopidogrel + AAS (RR 1,38, p = 0,001).^213^ Os efeitos benéficos da dupla antiagregação (DAP) ocorreram tanto em pacientes de alto risco como naqueles de risco intermediário ou baixo. Outras análises do estudo CURE demonstraram que a DAP também teria benefícios tanto nos pacientes submetidos à revascularização miocárdica cirúrgica como nos submetidos ao tratamento medicamentoso isolado.^214^

Quando os pacientes com SCASSST são tratados com ICP, o duplo bloqueio da agregação plaquetária torna-se imperativo, tanto com base em estudo que focalizou primariamente o contexto da ICP^215^ como também em trabalhos que abordaram as SCA *per se*. Nesta última condição, se inserem o subestudo do CURE (PCI-CURE), prospectivamente desenvolvido,^151^ e o estudo CREDO (Center for Research on Education Outcomes).^156^

Por conta das importantes limitações do clopidogrel, relacionadas ao seu metabolismo e interações medicamentosas, o fármaco foi testado também com dose dobrada no estudo CURRENT OASIS-7.^216^ Neste estudo, 25.086 pacientes com SCA (71% sem supra de ST) e programação de estratificação invasiva foram randomizados para receber dose em dobro do clopidogrel (600mg de ataque seguido por 150mg ao dia por 6 dias e 75mg ao dia após a primeira semana) ou a posologia habitual (300mg de ataque seguidos por 75mg ao dia). Ao final de 30 dias, não houve diferença significativa na ocorrência do desfecho primário composto de óbito cardiovascular, IAM não fatal ou AVC (HR 0,94; 4,2% *vs.* 4,4%, p = 0,30). No entanto, a dose dobrada de clopidogrel foi associada a aumento significativo na incidência de sangramentos maiores (HR 1,24; 2,5% *vs.* 2%, p = 0,01). Em análise pré-especificada,^212^ considerando-se apenas os pacientes submetidos à ICP (n = 17.263), demonstrou-se redução de 14% na incidência do desfecho composto primário do estudo aos 30 dias de evolução (HR 0,86; 3,9% *vs.* 4,5%, p = 0,039, número necessário para tratar [NNT] = 167), além de redução significativa de trombose definitivamente comprovada de *stents* (HR 0,54; 0,7% *vs.* 1,3%, p = 0,0001), porém à custa de maior incidência de sangramentos graves (HR 1,41; 1,6% *vs.* 1,1%, p = 0,009, NNH = 200). Considerando o NNT elevado, recomenda-se a avaliação cautelosa do risco individual prévio de sangramento para guiar uma decisão de dose neste contexto.

A “má resposta” (ou “resistência” para alguns) ao clopidogrel, expressão utilizada para caracterizar os pacientes que não atingem o nível de inibição plaquetária esperado, é identificada por meio de ensaios laboratoriais *in vitro* que quantificam a intensidade da agregabilidade plaquetária mediada pela via do ADP. Dados consistentes associam a má resposta ao clopidogrel à maior incidência de eventos trombóticos, principalmente em pacientes submetidos à ICP com implante de *stent.*^217^^-^^219^ Atualmente, três principais fatores estão relacionados com a má resposta ao clopidogrel: 1) variabilidade genética, caracterizada por polimorfismos associados às enzimas do citocromo P450 envolvidas no processo de metabolização hepática, notadamente a CYP2C19;^220^ 2) alteração no processo de absorção intestinal do medicamento relacionado com a expressão da glicoproteína P nas células epiteliais intestinais;^221^ 3) utilização concomitante de outros fármacos que podem interferir no metabolismo hepático mediado por enzimas do citocromo P450, como, por exemplo, o cetoconazol (que inibe o citocromo P450 e reduz a ação do clopidogrel) e a rifampicina (que estimula o citocromo P450 e acentua a ação do clopidogrel).

A associação dos inibidores de bomba de prótons (IBP) ao clopidogrel merece destaque. Diversos estudos *in vitro* indicam a ocorrência de redução na inibição plaquetária induzida por clopidogrel quando da associação deste ao IBP, e sugerem que isso seria especialmente frequente com o omeprazol.^222^^-^^225^ Entretanto, os estudos que a analisaram com a ocorrência de eventos isquêmicos mostraram resultados conflitantes^226^^-^^228^ e encontraram associação entre aumento de eventos isquêmicos e uso concomitante de clopidogrel + IBP. Já uma subanálise do estudo TRITON (Optimizing Platelet Inhibition with Prasugrel Thrombolysis in Miocardial Infarction) não encontrou qualquer associação^229^ e, interessantemente, subanálise similar do estudo PLATO (Platelet Inhibition and Patient Outcomes)^230^ encontrou aumento na incidência de eventos isquêmicos tanto no grupo clopidogrel quanto no grupo ticagrelor, quando utilizados em conjunto com IBP. Mais recentemente, análise do estudo TRILOGY demonstrou que, entre pacientes com SCA manejados sem revascularização do miocárdio, o uso de IBP não resultou em resposta antiplaquetária diferente entre prasugrel *versus* clopidogrel, mas foi associado com menor incidência de infarto no grupo prasugrel.^228^

O único estudo clínico randomizado que testou diretamente tal hipótese, o COGENT (Clopidogrel and the Optimization of Gastrointestinal Events),^231^ avaliou 3.761 pacientes com indicação de dupla terapia antiagregante por pelo menos 12 meses (um grupo recebendo clopidogrel e omeprazol *versus* um grupo recebendo clopidogrel e placebo). Entretanto, tal estudo foi interrompido precocemente por questões de financiamento e não teve o seguimento previsto dos pacientes (consequentemente, obteve-se número bastante reduzido de eventos), o que comprometeu de forma importante seu poder estatístico. De qualquer modo, até a finalização do número de pacientes randomizados no estudo, não foi verificada diferença na incidência de eventos isquêmicos (4,9% no grupo clopidogrel + omeprazol *vs.* 5,7% no grupo clopidogrel + placebo, p = 0,96). Como esperado, demonstrou-se maior incidência de sangramentos digestivos no grupo placebo (2,9% *vs.* 1,1%, com p < 0,001). Assim, um contingente apreciável de médicos e pesquisadores sugere que, em princípio, o uso de IBP (principalmente omeprazol), em conjunto com o clopidogrel, deva ser evitado. Os pacientes com maior risco de sangramento gastrintestinal (antecedente de hemorragia digestiva, úlcera péptica diagnosticada, infecção por *H. pylori*, idade ≥ 65 anos, uso concomitante de anticoagulantes ou esteroides) podem empiricamente receber bloqueadores dos receptores H2 (p. ex., ranitidina). Caso seja necessário o uso de um IBP, sugere-se o pantoprazol, cujo metabolismo via CYP P450 é menos pronunciado.

Portanto, o uso do clopidogrel está indicado para as SCASSST de risco moderado e alto para novos eventos isquêmicos. A administração consiste em uma dose de ataque de 300mg e manutenção com 75mg ao dia. Em pacientes submetidos à ICP e com baixo risco de sangramento, pode-se considerar a dose de ataque de 600mg, com manutenção de 150mg nos primeiros 7 dias e 75mg ao dia após esse prazo. O tempo de uso do medicamento deve ser de 12 meses, independentemente do tratamento recebido (clínico, percutâneo ou cirúrgico). Quando houver indicação de revascularização cirúrgica eletiva, o clopidogrel deverá ser suspenso pelo menos 5 dias antes do procedimento devido ao risco de sangramento. Não há demonstração de que o ajuste da terapia antiplaquetária guiada por métodos de avaliação da agregabilidade plaquetária seja superior à terapia antiplaquetária padrão, portanto, essa estratégia não deve ser utilizada rotineiramente, sendo reservada para situações especiais.^232^^-^^233^

Por conta das limitações do clopidogrel, novos fármacos foram desenvolvidos no sentido de se obterem bloqueios de agregação plaquetária mais rápidos, mais efetivos e mais consistentes.

##### 5.2.2. Prasugrel

O prasugrel é um tienopiridínico de geração mais recente e que cumpre tais objetivos. Isso ocorre basicamente porque esse medicamento tem um metabolismo mais simples em relação ao clopidogrel, com apenas uma fase de metabolização hepática. Como consequência, seu metabólito ativo atinge pico plasmático em apenas 30min, além de apresentar menor interação com medicações metabolizadas pelo citocromo P450.^234^

O estudo TRITON incluiu 13.608 pacientes com SCA, sem uso recente de tienopiridínico, anatomia coronariana conhecida (nos pacientes com SCASSST) e ICP planejada. Desses pacientes, 74% se apresentaram com SCASSST de risco moderado ou alto para complicações isquêmicas/trombóticas. Foram excluídos indivíduos plaquetopênicos, anêmicos ou com risco alto de sangramento. Os pacientes foram randomizados para clopidogrel (ataque de 300mg e manutenção de 75mg ao dia) ou prasugrel (60mg de ataque e manutenção de 10mg ao dia) após realização da coronariografia e indicação de ICP, sendo que o seguimento médio da população foi de 14,5 meses. O desfecho primário de eficácia do estudo, composto de óbito cardiovascular, (re)infarto e AVC, apresentou redução de 19% no grupo prasugrel (RR 0,81; 12,1% *vs.* 9,9%, p < 0,001), em comparação com o grupo tratado com clopidogrel. Com relação aos desfechos secundários de eficácia, o grupo tratado com prasugrel apresentou 24% de redução de IAM (RR 0,76; 9,5% *vs.* 7,3%, p < 0,001), 34% de diminuição da necessidade de revascularização urgente (RR 0,76; 3,7% *vs.* 2,5%, p < 0,001) e 52% de redução de trombose de *stent* (RR 0,48; 2,4% *vs.* 1,1%, p < 0,001).

A análise dos desfechos de segurança do TRITON revelou, no grupo prasugrel, aumento de 32% na incidência de sangramento maior pelo critério TIMI não relacionado com a cirurgia de revascularização (RR 1,32; 1,8% *vs.* 2,4%, p = 0,03), 52% de aumento de sangramento ameaçador à vida (RR 1,52; 0,9% *vs.* 1,4%, p = 0,01), além de um aumento significativo de sangramentos fatais (RR 4,1; 0,1% *vs.* 0,4%, p < 0,002).

O benefício líquido total do estudo mostrou que o prasugrel foi inferior ao clopidogrel em indivíduos com passado de AVC ou AIT prévio (RR 1,54, p = 0,04), foi neutro em indivíduos com > 75 anos de idade ou peso < 60kg, e superior em indivíduos sem passado de AVC ou AIT, com < 75 anos de idade e peso > 60kg (RR 0,8; p < 0,001).^154^

Aplicando a nova classificação de IAM ao material científico do TRITON-TIMI, Morrow et al. demonstraram que o prasugrel se mostrou mais eficaz que o clopidogrel na redução de diversos tipos de infarto.^235^

Subanálises do estudo evidenciaram resultados favoráveis ao prasugrel em pacientes diabéticos^236^ e também naqueles submetidos à cirurgia de revascularização miocárdica.^237^

O prasugrel também foi avaliado no estudo TRILOGY (TaRgeted platelet Inhibition to cLarify the Optimal strateGy), que incluiu 9.326 pacientes com SCASSST e um fator de risco adicional (idade mínima de 60 anos, diabetes melito, antecedente de IAM, ICP ou revascularização miocárdica), submetidos ou não à coronariografia, mas com indicação específica de tratamento clínico (sem revascularização miocárdica após o evento índice para entrada no estudo). Os pacientes foram randomizados para clopidogrel (300mg de ataque com 75mg ao dia de manutenção) ou prasugrel (30mg de ataque com manutenção de 10mg ao dia se idade < 75 anos, ou 5mg ao dia se idade ≥ 75 anos ou peso < 60kg), tendo sido acompanhados em média por 17 meses; o tempo médio de uso da medicação foi de 15 meses.^238^

Com relação ao desfecho primário de eficácia, composto de morte cardiovascular, IAM ou AVC, não houve diferença significativa entre os grupos prasugrel e clopidogrel (p = 0,21). Tampouco foram detectadas diferenças significativas entre os grupos no que se refere aos desfechos principais de segurança (sangramento grave ou ameaçador à vida pelo critério GUSTO ou sangramento grave pelo critério TIMI).

Finalmente, no TRILOGY, aproximadamente um terço dos pacientes foi submetido a análises de agregabilidade plaquetária e, apesar de um claro bloqueio mais efetivo da agregabilidade plaquetária no grupo prasugrel, não se conseguiu demonstrar significativa associação entre reatividade plaquetária e ocorrência de eventos isquêmicos/trombóticos.^238^

Assim como para o clopidogrel, o prasugrel, caso indicado, deve ser em princípio continuado por 12 meses. Salienta-se que a necessidade de suspensão do prasugrel é de pelo menos 7 dias antes de revascularização miocárdica eletiva.

##### 5.2.3. Ticagrelor

O ticagrelor também é inibidor da agregação plaquetária induzida por ADP, via bloqueio do receptor P2Y_12_, porém não pertence à classe dos tienopiridínicos. O ticagrelor é uma ciclopentiltriazolopirimidina (CPTP) com meia-vida de cerca de 12h e que, ao contrário dos tienopiridínicos, exerce bloqueio reversível dos receptores P2Y_12_ e não depende da metabolização hepática para o início de sua ação. Com tais características, o ticagrelor exerce efeito antiagregante plaquetário mais intenso, rápido e consistente em relação ao clopidogrel.^239^

No estudo PLATO,^152^ cerca de 18 mil pacientes admitidos por SCA de risco intermediário e alto com até 24h do início dos sintomas foram randomizados para receber ticagrelor (180mg de ataque seguidos por 90mg a cada 12h de manutenção) ou clopidogrel (300mg de ataque seguidos por 75mg ao dia de manutenção). Os pacientes que foram submetidos à ICP receberam dose adicional de 90mg de ticagrelor e poderiam, a critério do investigador, receber dose adicional de 300mg de clopidogrel. Todos os pacientes, exceto se houvesse contraindicação, receberam AAS, e a medicação do estudo foi mantida durante 12 meses, independentemente da estratégia de tratamento empregada (revascularização percutânea, cirúrgica ou tratamento clínico exclusivo). Nos pacientes com indicação de CRVM, o clopidogrel deveria ser suspenso 5 dias antes do procedimento, e o ticagrelor, em um intervalo de 24 a 72h antes da cirurgia. O desfecho primário de eficácia do estudo foi composto de óbito por causas vasculares, (IAM não fatal ou AVC em 12 meses; o desfecho primário de segurança foi a ocorrência de sangramento grave pelo critério do próprio estudo. Do número total de pacientes incluídos, a prevalência de SCASSST foi de aproximadamente 60%, sendo cerca de 43% dos pacientes com IAM sem supradesnível de ST e 17% daqueles com AI de risco intermediário e alto. A mediana de idade dos pacientes incluídos foi de 62 anos e 15% tinham mais de 75 anos. Aproximadamente 64% dos pacientes foram submetidos à ICP e 10%, à CRVM, permanecendo os demais em tratamento clínico exclusivo. Ao final do estudo, o uso do ticagrelor levou à redução significativa de 16% na incidência do desfecho primário composto de eficácia (RR 0,84; 9,8% *vs.* 11,7%, p < 0,001). Nas análises isoladas dos componentes do desfecho composto (metas secundárias principais), evidenciaram-se reduções significativas nas incidências de IAM (RR 0,84; 5,8% *vs*. 6,9%, p = 0,005) e óbitos por causas vasculares (RR 0,79; 4,0% *vs.* 5,1%, p < 0,001), não havendo diferenças significativas em relação à incidência de AVC (p = 0,22). Em relação ao desfecho primário de segurança, não se observou diferença significativa na incidência de sangramento grave avaliado tanto pelo critério elaborado pelo próprio estudo (HR = 1,04; p = 0,43) quanto pelo critério TIMI (HR = 1,03, p = 0,57). Apesar de não haver diferença na incidência de sangramento fatal (HR = 0,87; p = 0,66) ou na necessidade de transfusões (HR = 1; p = 0,96), o uso de ticagrelor levou a um aumento absoluto discreto, mas significativo, na incidência de sangramento intracraniano fatal (0,1 *vs*. 0,01; p = 0,02) e na incidência de sangramento maior não relacionado à CRVM (4,5% *vs*. 3,8%, p = 0,03). Outros efeitos adversos apresentaram maior incidência no grupo ticagrelor. Houve aumento significativo na ocorrência de dispneia (RR 1,84; 13,8% *vs.* 7,8%, p < 0,001), que, em geral, foi transitória e levou à suspensão do medicamento em menos de 1% dos pacientes. Existe controvérsia se este efeito colateral seria explicado por aumento nos níveis plasmáticos de adenosina ou não.^240^^-^^242^ Também houve aumento na incidência de bradicardia transitória, com elevação significativa na ocorrência de pausas ventriculares > 3 segundos nos primeiros 7 dias de uso da medicação (5,8% *vs.* 3,6%, p = 0,01), mas que perde tal significância após 30 dias de utilização do medicamento (2,1% *vs.* 1,7%, p= 0,52).^243^ Não houve diferença entre os grupos, no global da população estudada, quanto à necessidade de implante de MP, ocorrência de síncope ou bloqueio cardíaco.^243^ Houve elevação dos níveis de creatinina (10% *vs*. 8%) e de ácido úrico (14% *vs*. 7%), que foram revertidos 1 mês após o final do tratamento.^244^

O banco de dados o PLATO propiciou a publicação de diversas análises de subgrupos pré-especificados, como, por exemplo: diabéticos^245^ doença renal crônica,^246^ AVC prévio,^247^ utilização de IBP,^230^ estratégia invasiva ou conservadora inicial,^248^ revascularização miocárdica cirúrgica,^249^ recorrência de eventos,^250^ custo-efetividade,^251^ entre outros. De forma resumida, sugere-se que o ticagrelor seja custo-efetivo e que os resultados das subanálises realizadas ficaram muito próximos aos demonstrados na publicação original, com toda a população incluída. O medicamento deve ser suspenso pelo menos 3 dias antes de um procedimento cirúrgico de revascularização miocárdica eletiva.

Mais recentemente, foi publicado estudo (ISAR-REACT 5) multicêntrico, não cego, que comparou o uso de ticagrelor (180mg na chegada ao hospital seguido de 90mg 2 vezes/dia) *versus* prasugrel (60mg na sala de hemodinâmica seguido de 10mg ou 5mg nos ≥ 75 anos ou < 60kg) em 4.018 pacientes com SCA que seriam submetidos a tratamento invasivo precoce (41,1% de IAM com supra e 58,9% de SCASSST). O desfecho primário, composto de morte, IAM ou AVC em 1 ano de seguimento, ocorreu em 184 pacientes (9,3%) no grupo ticagrelor e em 137 pacientes (6,9%) no grupo prasugrel (HR 1,36; IC 95%, 1,09 a 1,70; p = 0,006); o sangramento maior (desfecho de segurança) foi semelhante em ambos os grupos (5,4% no grupo ticagrelor *vs.* 4,8% no grupo prasugrel – HR, 1,12; 95% CI, 0,83 a 1,51; p = 0,46). Ressaltam-se algumas limitações no estudo: estudo foi aberto (não cego); 90% dos contatos com os pacientes foram realizados de forma telefônica (83%) ou por carta (7%); ambas as medicações não foram dispensadas pelo estudo (paciente precisava adquiri-las por conta própria); a estratificação invasiva na SCASSST foi feita de forma bastante rápida, o que não é nossa prática; ocorrência do desfecho primário (6,9%) no grupo prasugrel foi mais baixa que no estudo TRITON TIMI 38 (9,9%) e bem abaixo do que se imaginava neste grupo (12,9%) durante o cálculo amostral.^252^

#### 5.3. Terapia antiplaquetária ajustada por testes de agregação plaquetária ou por testes genéticos

Três estudos clínicos randomizados buscaram demonstrar o impacto clínico de terapias antiplaquetárias diferenciadas em pacientes com má resposta ao clopidogrel. O estudo GRAVITAS (Gauging Responsiveness With A Verify Now Assay-Impact On Thrombosis And Safety)^232^ avaliou se uma dose elevada de clopidogrel (600mg de ataque seguidos por 150mg/dia de manutenção) seria superior à terapia padrão (75mg/dia sem dose de ataque adicional) na prevenção de eventos cardiovasculares após a ICP e implante de *stent* farmacológico. Os pacientes a serem randomizados foram selecionados de acordo com a presença de elevada reatividade plaquetária com o uso do clopidogrel, avaliada pelo método laboratorial do VerifyNow® P2Y_12_; dos 5.429 pacientes avaliados inicialmente, 2.214 (40,8%) apresentaram hiper-reatividade plaquetária (pela via ADP) e foram incluídos no estudo (40% com diagnóstico de SCASSST). Apesar de uma redução absoluta de 22% no número de pacientes que mantinham hiper-reatividade plaquetária (*P2Y*_12_ *reactionunit* [PRU] ≥ 230), o tratamento com dose alta de clopidogrel não foi capaz de reduzir o risco de óbito por causas cardiovasculares, IAM não fatal ou trombose de *stent* em 6 meses de evolução após a ICP (HR 1,01, IC 95% 0,58 a 1,76, p = 0,97). Tampouco houve aumento de hemorragias graves na comparação entre os grupos. No estudo ARCTIC,^233^ 2.440 pacientes com programação de ICP e implante de *stent* farmacológico (27% com SCASSST) foram randomizados para duas distintas estratégias de antiagregação plaquetária: uma convencional, em que a administração de AAS e iP2Y_12_ utilizava a posologias habitualmente empregadas, e outra na qual as doses eram ajustadas de acordo com os resultados da agregabilidade plaquetária obtidos pelo método laboratorial do VerifyNow® Aspirinand P2Y_12_. A randomização, a avaliação da reatividade plaquetária e a intervenção, quando indicadas ao grupo de terapia monitorada, foram realizadas antes do procedimento programado para implante do *stent*. A incidência de hiper-reatividade plaquetária nos pacientes em uso de clopidogrel foi de 34,5% e a resistência ao AAS foi observada em 7,6% dos mesmos. No grupo de terapia monitorada, nova avaliação da função plaquetária foi realizada 2 e 4 semanas após o implante do *stent*, e novos ajustes terapêuticos foram realizados quando necessário. Nessa nova avaliação, houve redução significativa de aproximadamente 50% (15,6% *vs*. 34,5% no momento da ICP; p < 0,001) no número de pacientes com hiper-reatividade plaquetária definida como valor de PRU ≥ 235 ou 15% ou menos de inibição plaquetária em comparação com valores de controle. No entanto, o desfecho primário composto de óbito por qualquer causa, IAM, AVC, AIT, revascularização coronária de urgência ou trombose de *stent* em até 1 ano foi de 34,6% nos pacientes que receberam terapia monitorada e 31,1% naqueles tratados de forma convencional (HR 1,13; IC 95% 0,98 a 1,29; p = 0,10). A taxa de eventos hemorrágicos graves também não diferiu significativamente entre os grupos. No estudo ANTARTIC, pacientes idosos (idade ≥ 75 anos) submetidos à ICP após SCA foram randomizados para uma estratégia guiada por testes de agregação plaquetária *versus* estratégia convencional. Todos os pacientes eram inicialmente tratados com prasugrel 5mg, e, no grupo de terapia guiada, a dose poderia ser incrementada para 10mg em caso de inibição plaquetária inadequada (definido como PRU ≥ 208) ou desescalonada para clopidogrel 75mg em caso de inibição plaquetária excessiva (definido como PRU ≤ 85). O desfecho foi composto de morte cardiovascular, AVC, infarto, revascularização urgente, trombose de *stent* ou sangramento clinicamente relevante em 12 meses (HR 1,00, IC 95% 0,78 a 1,29).^253^ Desse modo, a terapêutica antiplaquetária guiada por testes de agregabilidade só deve ser utilizada em casos selecionados.

Em relação aos testes genéticos, o estudo POPULAR Genetics testou a terapia guiada pela avaliação do polimorfismo do citocromo 2C19. Neste estudo, 2.488 pacientes com SCA foram randomizados para uma estratégia padrão com uso de ticagrelor ou prasugrel *versus* uma estratégia personalizada, em que os pacientes não portadores de polimorfismos, levando à perda de função do 2C19 (e, portanto, com metabolização normal do clopidogrel), recebiam clopidogrel em vez dos outros dois antiplaquetários. A estratégia personalizada foi não inferior à convencional para o desfecho composto de morte, infarto, AVC, trombose de *stent* ou sangramento maior, ao mesmo tempo em que reduziu sangramento maior (HR 0,78; IC 95% 0,61 a 0,98; p = 0,04).^254^ Já o estudo TAILOR PCI incluiu pacientes submetidos à ICP (aproximadamente 80% com SCA e 60% apresentando SCASSST) e avaliou a estratégia de terapia guiada por genotipagem. Tal estratégia não foi superior ao tratamento convencional na redução do desfecho composto de morte cardiovascular, infarto, AVC, trombose de *stent* ou isquemia grave recorrente (HR 0,66; IC 95% 0,43-1,02; p = 0,06) em pacientes carreadores do alelo que leva à perda de função do 2C19 (aproximadamente 35% dos pacientes incluídos).^255^ Novos estudos, inclusive com análise de custo-efetividade, são necessários para confirmar tais resultados antes de uma decisão definitiva quanto ao rotineiro uso da genotipagem.

**Table:** 

Antiplaquetários - Sumário de recomendações e evidências		
AAS (162-300mg em dose de ataque, com dose de manutenção de 81-100mg/dia) em todos os pacientes, salvo contraindicações, independentemente da estratégia de tratamento, continuando por tempo indeterminado.	I	A
Tienopiridínicos em pacientes com intolerância ao AAS.	I	B
Uso de terapia antiplaquetária dupla por 12 meses após o evento agudo (independentemente da estratégia inicial - clínico, percutâneo ou cirúrgico), salvo contraindicações.	I	A
Clopidogrel (300mg em dose de ataque, com manutenção de 75mg/dia) em adição ao AAS, em pacientes portadores de SCASSST de risco intermediário ou alto por 12 meses, em pacientes com risco muito alto de sangramento, em uso concomitante de anticoagulantes orais ou quando prasugrel ou ticagrelor não estiverem disponíveis.	I	B
Ticagrelor (180mg de ataque seguidos por 90mg 2 vezes / dia) em pacientes portadores de SCASSST de risco intermediário ou alto, independente da estratégia de tratamento posterior (clínico, cirúrgico ou percutâneo), preferencialmente ao clopidogrel, por 12 meses.	I	B
Prasugrel 60mg de ataque seguidos por 10mg ao dia em pacientes portadores de SCASSST de risco moderado ou alto, com anatomia coronariana conhecida, tratados com ICP e sem fatores de risco para sangramento (idade ≥ 75 anos; com < 60kg; AVC ou AIT prévios), preferencialmente ao clopidogrel, por 12 meses.	I	B
Clopidogrel 600mg em dose de ataque seguidos por 150mg ao dia por 7 dias e dose posterior de 75mg ao dia), em adição ao AAS, em pacientes submetidos à ICP com alto risco de eventos isquémicos e baixo risco de sangramento.	IIa	B
Reinicio de ticagrelor, prasugrel ou clopidogrel após cirurgia de revascularização miocárdica, assim que seguro.	IIa	B
Uso de testes de agregabilidade plaquetária ou testes genéticos (genotipagem) em casos selecionados.	IIb	B
Combinação de AAS com outros anti-inflamatórios não esteroidais (AINE).	III	C
Utilização de prasugrel em pacientes com histórico de AIT ou AVC prévio ou pacientes com SCASSST sem anatomia conhecida.	III	B

### 6. Antitrombínicos

#### 6.1. Heparina Não Fracionada

Entre os anticoagulantes injetáveis, a HNF foi a primeira sintetizada. A heparina é um polissacarídeo sulfatado com uma faixa de peso molecular de 3.000 a 30.000Da (média, 15.000Da). Sua ação se deve a inativação da trombina e fator ativado X (fator Xa) por um mecanismo dependente da antitrombina (AT). A heparina se liga à AT por meio de um pentassacarídeo de alta afinidade e, para inibir a trombina, esta ligação se faz tanto à AT quanto à enzima de coagulação. Ao inativar a trombina, previne a formação de fibrinas e inibe a ativação induzida pelas trombinas de plaquetas e dos fatores V e VIII. Sua resposta variável se faz pela ligação independente da AT de heparina a proteínas plasmáticas, pelas proteínas liberadas pelas plaquetas e células endoteliais, ocasionando a resistência à heparina e uma resposta farmacológica variável. Por essa razão, é importante o monitoramento por meio do tempo de ativação da tromboplastina (TTPa). Devido à sua meia-vida curta, a HNF deve ser administrada com uma infusão intravenosa contínua para garantir níveis estáveis de anticoagulação.^256^^,^^257^

As evidências para o uso de HNF no tratamento do IAMSSST são provenientes de estudos randomizados e metanálises. Eikelboom JW et al., em uma metanálise de seis estudos randomizados, comprovaram que o uso de HNF, ou heparinas de baixo peso molecular (HBPM), reduz os desfechos cardiovasculares maiores nas SCA.^182^ A HNF permanece um anticoagulante amplamente utilizado no IAMSSST quando a cineangiocoronariografia ocorre nas primeiras 2h da chegada ao hospital, apesar de evidências consistentes de maior risco de sangramento em comparação com outros antitrombóticos.^191^

O regime terapêutico proposto para o uso de HNF deve ser ajustado ao peso corporal. Deve-se administrar por via intravenosa uma dose inicial de 60 unidades/kg, e seguir a infusão de 12 unidades/kg/hora. A intensidade e o ajuste da anticoagulação é realizado pelo monitoramento de TTPa. A primeira amostra de sangue deve ser coletada na terceira hora de infusão e, posteriormente, a cada 6h, até que a faixa-alvo seja atingida, a partir do que se pode coletar a cada 24h. O intervalo terapêutico é estreito, por isso deve ser mantido valores de TTPa entre 1,5 e 2,0 vezes.^198^

Durante intervenção coronariana percutânea, deve ser administrada uma dose de HNF para manter o tempo de coagulação ativado (TCA) entre 250 e 350 segundos. A titulação da dose deve ser orientada pelo peso corporal. A administração de um *bolus* de 70-100UI/kg de HNF é suficiente para uma anticoagulação adequada. Na vigência de uso concomitante de inibidores dos receptores IIb/IIIa, a dose inicial deve ser de 60-70UI/kg.^258^

Os efeitos colaterais mais frequentes da HNF são os sangramentos maiores ou menores, que ocorrem principalmente quando o tempo de coagulação está acima da faixa terapêutica. A HNF pode desencadear uma reação imunológica intensa, a trombocitopenia induzida por heparina (TIH) – condição clínica que pode ser fatal, pois induz, concomitantemente, sangramento e trombose.^258^

Na vigência de sangramentos que ameaçam a vida, ou na necessidade de cirurgia de urgência, o uso de um reversor do efeito anticoagulante é imperativo O reversor específico do efeito da HNF é o sulfato de protamina. Uma vez que a meia-vida da HNF é de aproximadamente 1 a 1,5h, a dose de protamina necessária para reverter o efeito da HNF deve se basear na dose total de HNF administrada nas últimas 2 a 3h. Aproximadamente 1mg neutraliza 100 unidades de HNF. Recomenda-se uma infusão IV lenta para evitar hipotensão arterial e bradicardia.^258^

#### 6.2. Heparinas de Baixo Peso Molecular

Durante investigações para melhor compreensão da estrutura da heparina convencional (HNF), verificou-se que suas cadeias de polissacarídeos podem sofrer despolimerização por meio de vários processos físicos e químicos para obter compostos também heterogêneos, porém de mais baixo peso molecular, que recebem o nome genérico de heparinas fracionadas ou de baixo peso molecular (HBPM).^256^^,^^257^

As formas de heparina de baixo peso molecular são constituídas de cadeias curtas de polissacarídeos, o que resulta em um efeito anticoagulante mais previsível quando comparada à HNF. A HBPM tem várias vantagens potenciais sobre HNF:^257^

- Maior atividade antifator Xa em relação ao fator IIa. Proporciona uma inibição mais efetiva na geração de trombina.
- Promove uma inibição maior do que as HNF na via do fator tecidual.
- Induz menos frequentemente plaquetopenia.
- Administração via subcutânea por sua grande biodisponibilidade.
- Promove uma anticoagulação previsível em virtude de menor ligação em proteínas plasmáticas.
- Não necessita de monitoramento de níveis plasmáticos.

A fraxiparina, dalteparina e a enoxaparina são exemplos de HBPM que foram testadas em SCA. A enoxaparina permaneceu como fármaco eleito para o uso nesse contexto, devido ao maior corpo de dados experimentais; e os estudos prévios evidenciaram que a fraxiparina e a nadroparina são similares à HNF.

Dois estudos foram publicados (TIMI 11B e ESSENCE) comparando a enoxaparina com a HNF, em termos de eficácia clínica e segurança, no tratamento de pacientes com AI e IAM sem supradesnível de ST. Em resumo, demonstraram pela primeira vez que uma HBPM (no caso, a enoxaparina) era superior à HNF, além de demonstrar que não existe benefício adicional com a utilização do medicamento após a fase de hospitalização (TIMI 11B); durante a fase ambulatorial, ocorreu hemorragia importante em 1,5% do grupo tratado com placebo e 2,9% no grupo tratado com enoxaparina (p = 0,021).^259^^,^^260^ Talvez mais importante, a análise conjunta dos dois estudos demonstrou diminuição significativa na incidência de eventos “duros”, óbito ou (re)infarto, a favor da enoxaparina em relação à HNF, sendo essas vantagens ainda perceptíveis 1 ano após o tratamento inicial.^261^

O estudo SINERGY foi desenhado para definir o uso da enoxaparina comparada com a HNF em pacientes com SCASSST de alto risco. Foram randomizados 10.027 pacientes, cujo objetivo primário foi o composto de morte cardiovascular, IAM, AVC e revascularização de urgência. Os resultados demonstraram para o braço da enoxaparina que a taxa de eventos foi de 14,0% (696/4.993); nos pacientes randomizados à HNF, a taxa de eventos foi 14,5% (722/4.985) (RR, 0,96; IC 95%, 0,86-1,06). Em relação aos achados de segurança, observou-se mais sangramento com enoxaparina, com aumento estatisticamente significativo no sangramento maior (9,1% *vs.* 7,6%, p = 0,008), (critérios TIMI) e um incremento não significativo de sangramento graves (critérios GUSTO) (2,7% *vs.* 2,2%, p = 0,08) e transfusões de sangue (17,0% *vs.* 16,0%, p = 0,16). A conclusão dos autores foi que a enoxaparina não foi superior à HNF, tampouco, não foi inferior para o tratamento de pacientes de alto risco com SCASSST.^262^ A enoxaparina é uma alternativa segura e eficaz à HNF; as vantagens da conveniência do uso por via subcutânea e o fato de não necessitar de monitoramento da anticoagulação rotineiramente devem ser considerados com o excesso modesto de sangramento maior. Outra consideração originada de uma análise não pré-especificada foi a que devemos evitar a troca de classes de anticoagulantes durante o manejo da fase hospitalar dos pacientes com SCA.^262^

A dose de enoxaparina é de 1mg/kg administrado por via subcutânea (SC) duas vezes ao dia. Na idade superior a 75 anos, a dose deve ser reduzida para 0,75mg/kg duas vezes ao dia. Em pacientes com a taxa de filtração glomerular estimada (TFGe) igual ou inferior a 30mL/min/1,73m^2^, a dose deve ser reduzida pela metade, sendo então administrado 1mg/kg (SC) uma vez ao dia. Quando a TFGe chegar em 15mL/min/1,73m^2^, devemos evitar o uso de enoxaparina. Uma alternativa nessa condição é a HNF, pois tem metabolismo exclusivamente hepático. Nos pacientes pré-tratados com enoxaparina, não é recomendado o uso de enoxaparina adicional durante a intervenção coronariana percutânea se a última injeção SC de enoxaparina tiver sido administrada 8h antes do procedimento. Um *bolus* adicional 0,3mg/kg IV é recomendado se a última dose SC de enoxaparina tiver sido administrada ≥ 8h antes da angioplastia.^263^

#### 6.3. Fondaparinux

O fondaparinux é um pentassacarídeo sintético, constituído por uma sequência de açúcares (A B C D F) – a menor sequência capaz de se ligar diretamente a trombina. Esta descoberta foi um marco no desenvolvimento farmacológico das heparinas. Forneceu as ferramentas para o conhecimento da biologia e para o avanço da farmacologia dessa classe de medicamentos. Permitiu o conhecimento de que o bloqueio seletivo e direto do fator Xa promove um importante efeito antitrombótico, abrindo o caminho para o desenvolvimento dos novos anticoagulantes orais diretos.^264^ Pentassacarídeo sintético que se liga seletivamente à trombina leva à inibição do fator Xa. Devido à sua discreta interação com componentes do plasma, possui ação anticoagulante previsível e pouca variabilidade individual. Apresenta boa biodisponibilidade, favorecendo a administração por via subcutânea. Atinge seu pico plasmático rapidamente em 2h, e tem meia-vida longa de 17h, o que permite o uso de dose única diária. A eliminação do fondaparinux é exclusivamente pelos rins, sendo necessário o monitoramento renal. Quando TFGe for menor que 20/mL/1,73m^2^, devemos suspender seu uso.^265^

O estudo clínico que testou a eficácia e a segurança do fondaparinux em pacientes com SCASSST em comparação com a enoxaparina foi OASIS-5. Ensaio clínico foi desenhado para avaliar se o fondaparinux preservaria os benefícios anti-isquêmicos da enoxaparina enquanto reduziria o sangramento em pacientes com SCA. Foram randomizados 20.078 pacientes com SCASSST que receberam fondaparinux (2,5mg/dia) ou enoxaparina (1mg/kg, duas vezes ao dia) por uma média de exposição de 6 dias. O desfecho composto primário de eficácia foi: morte cardiovascular, IAM ou isquemia refratária no 9º dia. O sangramento maior foi o desfecho primário de segurança, e sua combinação o benefício clínico líquido. Pacientes foram seguidos por até 6 meses. Os resultados demostraram que a taxa de eventos do desfecho composto de eficácia foi semelhante nos dois grupos (579 com fondaparinux [5,8%] *vs.* 573 com enoxaparina [5,7%]; RR: 1,01 no grupo fondaparinux; 95% (IC 90-1.13; p para não inferioridade = 0,007).^194^ Subanálises do estudo foram publicadas na sequência, sendo a principal aquela que analisou os 12.715 pacientes submetidos à angiografia durante a hospitalização (6.238 tratados por ICP). Durante o andamento do OASIS 5 (após relatos de alguns centros sobre a incidência de trombose de cateteres) ocorreu uma emenda ao protocolo que orientava a lavagem dos cateteres de hemodinâmica com 200UI de HNF previamente ao procedimento. Entre os pacientes submetidos à ICP, o fondaparinux mostrou eficácia similar à enoxaparina para o desfecho primário em 9 dias (6,2% *vs.* 6,3%; RR 1,09; p = 0,79), com redução na incidência de sangramentos graves (10,4% *vs.* 8,2%; RR = 0,78; p = 0,004); entretanto, registrou-se maior incidência de trombose de cateteres (0,4% *vs.* 0,9%), sendo essa complicação associada a taxas mais elevadas de AVC e IAM.^266^^,^^267^

**Table:** 

Antitrombínicos - Sumário de recomendações e evidências		
Uso rotineiro de HNF.	I	A
Uso rotineiro de HBPM.	I	A
Uso de fondaparinux 2,5mg SC, 1 vez ao dia por 8 dias ou até a alta hospitalar.	I	B
Aos pacientes em uso de fondaparinux, administrar HNF 85UI / kg IV, no momento da ICP ou 60UI/kg àqueles que estiverem recebendo IGPs IIb/IIIa.	I	B
Uso de enoxaparina preferencialmente à HNF, a não ser que cirurgia de revascularização miocárdica esteja planejada para as próximas 24h.	IIa	A
Considerar interrupção da anticoagulação após a ICP, exceto se houver outra indicação para mantê-la.	IIa	C
Troca de heparinas (HNF e enoxaparina).	III	B

### 7. Associação de Antiplaquetários e Anticoagulantes Orais Diretos em Situações Especiais

Nos últimos anos, os quatro DOAC foram estudados no cenário de fibrilação atrial em pacientes submetidos à angioplastia, e passaram a ser ótimas opções ao uso da varfariana.

Os estudos clínicos que testaram o uso dos DOAC no contexto de DAC (crônica e aguda) associada à fibrilação atrial apresentaram os seguintes resultados quando comparados com a terapia tríplice (AAS + clopidogrel + varfarina):

• Estudo PIONNER AF-PCI utilizou rivaroxabana 15mg (reduzido para 10mg no pacientes com “*clearance* de creatinina” entre 30 a 50mL;min) + iP2Y_12_ (clopidogrel/ticagrelor) por 12 meses e foi associado a menores taxas de sangramentos clinicamente significantes (meta primária); sendo que o componente de eficácia (morte cardiovascular, infarto e AVC) foi semelhante (desfecho secundário).^268^

• Estudo REDUAL PCI utilizou dabigatrana 110mg ou 150mg 2 vezes/dia + iP2Y_12_ (clopidogrel/ticagrelor) por 12 meses e foi associado a menores taxas de sangramentos clinicamente significantes (meta primária), sendo o “p” significativo para não inferioridade em ambos os grupos com dabigatrana e significativo para superioridade somente no grupo dabigatrana 110mg; a terapia dupla (ambos os grupos dabigatrana) foi não inferior à terapia tríplice em relação ao desfecho composto de eventos tromboembólicos.^269^

• Estudo AUGUSTUS utilizou apixabana 5mg 2 vezes/dia (ou 2,5mg 2 vezes/dia caso houver duas ou mais características – idade >80 anos, peso < 60kg ou creatinina >1,5) + iP2Y_12_ (clopidogrel) e foi associado a menor taxa de sangramento (meta primária) e menor taxa de morte ou hospitalização (desfecho secundário) sem diferença significativa na incidência de eventos isquêmicos. Ressalta-se que, no estudo da apixabana, foram incluídos também pacientes que apresentavam SCA sem realização de ICP.^270^

• Estudo ENTRUST AF-PCI utilizou a edoxabana 60mg em associação a iP2Y_12_ (n = 751) e, no outro grupo, varfarina associada à dupla antiagregação plaquetária (DAPT = AAS + iP2Y_12_), totalizando 1.506 pacientes. O desfecho primário foi o de sangramento maior, (HR = 0,83; IC 95% 0,65-1,05; p = 0,0010 para não inferioridade e p = 0,1154 para superioridade). Para o desfecho secundário de morte cardiovascular, AVC, embolia sistêmica, IAM e trombose de *stent*, não houve diferença significativa.^271^

Cerca da metade dos pacientes era portadora de SCA, e o uso do clopidogrel como iP2Y_12_ foi bem mais frequente que o ticagrelor. Esses estudos tiveram, sempre, como objetivo primário, o desfecho de segurança (sangramento). Com diferentes desenhos, demonstraram segurança dos DOAC em relação à varfarina quando associados a antiplaquetários; e os desfechos isquêmicos (como desfechos secundários) não apresentaram diferenças significativas.^272^ O tempo total de utilização da terapia tripla, seja com os DOACs ou com varfarina, e de suspensão de AAS deverá ser feito conforme o risco de eventos isquêmicos e o risco de eventos hemorrágicos^273^

### 8. Hipolipemiantes

Vários estudos comprovam os benefícios das terapias para a redução do colesterol em pacientes em prevenção secundária.^274^^,^^275^ Nos pacientes de alto risco, o benefício é independente dos níveis basais do LDL-C.^276^ Estudo de Dondo et al. com mais de 389 mil pacientes com SCASSST e seguimento médio de 2,2 anos mostrou que a não aderência ao tratamento com estatinas foi um dos fatores de maior impacto relacionados à redução da sobrevida. Se as diretrizes fossem aplicadas e seguidas corretamente, a aderência ao uso das estatinas, juntamente com a realização de angiografia coronária quando indicada, reabilitação cardiovascular e cessação do tabagismo, poderia evitar 28,9% dos óbitos.^277^

As metas lipídicas variam conforme as diretrizes, porém, nos últimos anos, foi incorporada uma estratégia de redução mais rigorosa dos níveis de LDL, sendo preconizados, em 2017, pela Diretriz brasileira sobre dislipidemias e prevenção da aterosclerose, níveis de LDL menores que 50mg/dL e não HDL < 80mg/dL nos pacientes classificados de muito alto risco.^278^ Quando as estatinas não são toleradas em doses maiores, uma opção é a associação da ezetimiba.^279^

Admite-se que o ideal seja iniciar terapia redutora de lípides desde a hospitalização, desde que não haja contraindicação. O estudo SECURE-PCI avaliou o uso de dose de ataque de atorvastatina 80mg antes da coronariografia em 4.191 pacientes com SCA submetidos à estratégia invasiva. O uso da dose de ataque não reduziu desfechos cardiovasculares maiores em 30 dias comparado ao placebo (HR 0,88; IC 95% 0,69 a 1,11; p = 0,27), embora tenha havido possível benefício no subgrupo de pacientes que foram submetidos à ICP (HR 0,72; IC 95% 0,54 a 0,96; p = 0,02).^280^

**Table:** 

Hipolipemiantes - Sumário de recomendações e evidências		
Iniciar tratamento precoce com dose alta de estatina em todos os pacientes, independentemente dos níveis de LDL, desde que não existam contraindicações.	I	A
Associar ezetimiba em pacientes em uso de dose máxima tolerada de estatina se os níveis de LDL não atingirem as metas estabelecidas.	I	A

### 9. Inibidores do Sistema Renina-Angiotensina-Aldosterona

Este grupo de fármacos é muito importante no tratamento de hipertensão arterial, insuficiência cardíaca e alguns grupos de pacientes com DAC. Não há evidências conclusivas de benefícios na utilização precoce dos mesmos em pacientes com SCASSST, mas alguns estudos sugerem que podem ser úteis na fase crónica após o episódio agudo. O estudo HOPE (The Heart Outcomes Prevention Evaluation)^281^ incluiu pacientes de alto risco para eventos cardiovasculares, frequentemente com doença arterial aterosclerótica importante (geralmente atingindo o território coronário) e independentemente da fase em que se encontravam e mostrou benefício com o uso de ramipril (dose alvo de 10mg/dia) a longo prazo. Em 5 anos de seguimento observou-se redução do risco relativo de óbito em 26% (p < 0,001); infarto, 20% (p < 0,001); e AVC, 32% (p < 0,001). Resultados similares também foram demonstrados em pacientes com coronariopatia crónica com o uso de perindopril.^282^

O benefício é maior nos pacientes com disfunção de ventrículo esquerdo (VE), e IAM anterior, em que colaboram na melhora do remodelamento cardíaco, melhora da função do VE, com diminuição da progressão para insuficiência cardíaca.^283^

O objetivo do tratamento é chegar a maior dose tolerável das medicações, até captopril 50mg de 8/8h (podendo ser substituído na sequência por ramipril 10mg/dia ou enalapril 20mg/dia 12/12h), losartana 50mg 12/12h ou valsartana 320mg/dia.

No caso dos antagonistas de aldosterona, o estudo EPHESUS mostrou redução de mortalidade com uso do eplerrenone (não disponível em nosso meio) em pacientes com IAM (com ou sem supra de ST) e disfunção ventricular esquerda apresentando sintomas de IC ou diabetes (RR 0,85; IC 95% 0,75-0,96; p = 0,008), sendo o benefício evidente já nos primeiros 30 dias de tratamento.^284^^,^^285^ Previamente, o estudo RALES já havia demonstrado diminuição da mortalidade em pacientes com insuficiência cardíaca crônica e fração de ejeção do ventrículo esquerdo (FEVE) ≤ 35%.^286^ Dessa maneira, o uso da espironolactona (antagonista de aldosterona disponível em nosso meio), dose-alvo de 25mg uma vez ao dia (podendo ser aumentada para 50mg após 8 semanas de tratamento se houver sinais de progressão da insuficiência cardíaca sem hipercalemia). O uso do bloqueador de aldosterona está contraindicado em pacientes com disfunção renal importante (Cr > 2,5mg/dL) ou níveis de K > 5,5mEq/L. Deve haver monitoramento cuidadoso de K e função renal, sobretudo quando do uso concomitante com IECA/BRA.

**Table:** 

Inibidores do sistema renina-angiotensina-aldosterona - Sumário de recomendações e evidências		
Administrar inibidores da enzima de conversão da angiotensina (IECA) a pacientes de risco intermediário e alto com disfunção ventricular esquerda, hipertensão arterial ou diabetes melito.	I	A
Administrar bloqueadores dos receptores da angiotensina II a pacientes de risco intermediário e alto com contraindicação aos IECA.	I	C
Uso de espironolactona em pacientes com SCASSST, FEVE ≤ 35%, e sintomas de IC ou histórico de diabetes.	I	C
Administrar IECA a todos os pacientes de risco intermediário e alto.	IIb	B

### 10. Estratificação de Risco com os Métodos Complementares

Em pacientes com SCA, a estratificação de risco deve ser um processo contínuo, desde a avaliação clínica inicial, passando pelos exames subsidiários já discutidos nestas Diretrizes, e culminando com os métodos complementar expostos a seguir.

### 11. Exames Não Invasivos para Diagnóstico de Isquemia e Avaliação Prognóstica

Os exames funcionais são indicados para avaliação da isquemia miocárdica e são fundamentais para a tomada de decisão em alguns casos, principalmente no diagnóstico e prognóstico dos pacientes com risco intermediário e alto. Na abordagem terapêutica conservadora, utilizam-se mais os métodos não invasivos prognósticos.

#### 11.1. Teste Ergométrico

Na fase de hospitalização, os parâmetros medidos no teste determinam o grau residual de isquemia e servem para avaliar o desempenho cardíaco. A isquemia residual é estimada por meio do comportamento do segmento ST durante o exercício ou na fase de recuperação e da presença de dor anginosa. O desempenho cardíaco é avaliado por meio do comportamento da pressão arterial e do duplo produto. O desempenho cardíaco e a resposta autonômica relacionada com o comportamento da redução da FC na fase de recuperação têm apresentado melhor correlação com mortalidade em comparação com outros parâmetros. Seu valor preditivo negativo é muito elevado, de 98% a 100%, embora com valor preditivo positivo modesto de aproximadamente 50%, sendo os TE anormais infrequentes na população indicada para esse procedimento estratificado nas unidades de dor torácica.^287^^-^^289^

Os principais preditores independentes de sobrevida livre de eventos (morte e IAM) em 1 ano, em análise de regressão multivariada, foram o número de derivações com depressão do segmento ST e a carga máxima alcançada. Quanto à segurança, o TE em pacientes estabilizados tem a incidência de 0,5% de complicações (óbito ou IAM) em até 24h após o exame.^290^^-^^292^

Com o objetivo de estimar o prognóstico e auxiliar na decisão clínica em pacientes de risco intermediário, o TE está indicado a esse grupo de pacientes 24 a 48h após completa estabilização clínica (estabilidade hemodinâmica, ausência de isquemia ativa clínica ou eletrocardiográfica, de novas ondas Q, de sinais clínicos de insuficiência cardíaca e marcadores de lesão miocárdica normais) e desde que haja capacidade para o exercício.^51^^,^^198^

Assim, o TE como estratégia precoce (< 48h) é contraindicado formalmente a pacientes de alto risco. Entretanto o TE realizado após 48h de plena estabilização do quadro clínico, ainda durante a internação, poderá ser indicado a pacientes submetidos à cinecoronariografia, quando é necessária avaliação funcional de lesão conhecida ou estabelecimento de risco antes da alta hospitalar e para orientar adequadamente programas de reabilitação cardíaca.^293^^,^^294^

**Table:** 

Teste ergométrico - Sumário de recomendações e evidências		
Realização de teste ergométrico em pacientes de risco intermediário, 24 a 48h após completa estabilização clínica, desde que sem sintomas isquêmicos ativos, sem sinais de insuficiência cardíaca ou comprometimento hemodinâmico, com ECG de repouso sem isquemia, e marcadores de necrose miocárdica normais.	I	B
Realização de teste ergométrico em pacientes de alto risco após 48h.	IIb	C
Realização de teste ergométrico em pacientes de alto risco antes de 48h.	III	C

#### 11.2. Métodos ecocardiográficos (isquemia, viabilidade – estresse, contraste / microbolhas, strain etc.)

##### 11.2.1. Ecocardiograma transtorácico

A nova análise da alteração contrátil do VE pode, de fato, ajudar na determinação de diagnóstico e prognóstico da SCA.^295^^-^^299^ O índice de motilidade segmentar (WMSI; do inglês, *wallmotion score index*) é o parâmetro de referência para expressar a função segmentar do VE, e seu valor de normalidade é 1 (o VE é dividido em 16 ou 17 segmentos que recebem classificação com base no espessamento sistólico); valores entre 1 e 1,6 evidenciam alteração contrátil de grau discreto; enquanto valores de WMSI superiores a 1,6 apontam maior acometimento e pior prognóstico. Obviamente, a ausência de alterações da contratilidade segmentar no ETT de repouso não exclui a presença de DAC.^300^

Vale lembrar que a avaliação da função ventricular pelo ETT, na fase aguda da isquemia, pode estar comprometida pelo fenômeno do *stunning* (atordoamento) miocárdico. Após um período de 2 semanas, pode ocorrer melhora acentuada da função ventricular.^301^

Com as novas tecnologias desenvolvidas nos aparelhos de ecocardiografia, como *speckle-tracking* 2D, tem sido possível, por meio da análise do *strain global longitudinal* (SGL) das paredes do VE, diagnosticar precocemente alterações isquêmicas em pacientes com alteração de troponina, porém sem alteração do ECG ou ecocardiograma de repouso (25% a 75% dos paciente com SCA têm ecocardiograma normal).^302^

O SGL (quando < 16,5%) pode complementar algoritmos diagnósticos existentes e agir como marcador adjunto precoce de isquemia.^303^ Publicação recente propõe que o SGL seja uma ferramenta utilizada na procura de pacientes candidatos à estratégia invasiva.^304^ Pequenos centros têm utilizado SGL rotineiramente na unidade de emergência e há registros de alteração do padrão de *strain* (< 18%) antes mesmo da alteração da troponina.^305^

Em circunstâncias especiais, quando a janela acústica é subótima, precisa ser complementado pelo exame de ecocardiografia contrastada por microbolhas, a fim de melhor se delimitar os bordos endocárdicos ou de avaliar eventuais defeitos de perfusão miocárdica.^306^

**Table:** 

Ecocardiograma transtorácico - Sumário de recomendações e evidências		
Avaliação da função ventricular global e segmentar.	I	C
Diagnóstico diferencial de causas alternativas de dor torácica: estenose aórtica grave, cardiomiopatia hipertrófica, embolia pulmonar, dissecção de aorta, pericardite e presença de tumores cardíacos.	I	C
Dor torácica com instabilidade hemodinâmica e suspeita de origem cardíaca.	I	C
Suspeita de complicações mecânicas no infarto do miocárdio: aneurisma do ventrículo esquerdo, ruptura de parede livre ou músculo papilar, comunicação interventricular, derrame pericárdico.	I	C
Cálculo do SGL por meio do *speckle tracking* como adjunto aos algoritmos existentes de diagnóstico e classificação de risco em pacientes com suspeita de doença coronariana.	IIa	B

##### 11.2.2. Ecocardiografia de estresse

A ecocardiografia sob estresse permite a verificação das anormalidades regionais transitórias da contração, indicativas de isquemia induzida tanto por exercício quanto farmacologicamente.^307^

O ecocardiograma sob estresse é um preditor independente de morte cardiovascular, de valor adicional aos demais métodos e pode evitar a cinecoronariografia. Seu uso pode ser recomendado para estratificação de risco de pacientes em unidades de dor torácica, especialmente quando o ECG não define o diagnóstico e o teste ergométrico é submáximo, de realização não factível ou com resultado inconclusivo, uma vez aliviada a dor por pelo menos 24h em pacientes de baixo a moderado risco e sem alterações isquêmicas evidentes no ECG e marcadores de necrose.^307^^,^^308^

Respostas de melhora de contração segmentar em áreas dissinérgicas, com doses iniciais de dobutamina (5 a 10g/kg/min), identificam viabilidade miocárdica nessas regiões “atordoadas” pela isquemia pregressa.^307^^-^^309^

**Table:** 

Ecocardiograma de estresse - Avaliação pelo ecocardiograma sob estresse - Sumário de recomendações e evidências		
Pacientes com angina instável ou SCASSST de baixo risco controlada clinicamente* antes de decidir a estratégia invasiva.	IIa	A
Para avaliar o significado funcional de obstrução coronariana moderada na angiografia, desde que o resultado interfira na conduta.	IIa	C
Estratificação de risco após infarto do miocárdio não complicado.	IIa	A
Investigação de pacientes com suspeita de doença microvascular para estabelecer se ocorre alteração segmentar em conjunção com a angina e as alterações eletrocardiográficas.	IIa	C
Parâmetros de *strain* e *strain rate* derivados do *speckle tracking* como ferramenta adjunta ao *wall motion score index* para diagnóstico e/ou prognóstico de doença coronariana aguda.	IIa	B
Angina instável de alto risco ou infarto agudo do miocárdio.	III	C

* Ausência de recorrência da angina, sem sinais de insuficiência cardíaca, sem alterações no ECG inicial/seriado e troponina normal; dor anginosa típica com alteração ao ECG ou prova funcional, na vigência de cinecoronariografia normal.

##### 11.2.3. Avaliação da perfusão miocárdica pela ecocardiografia

Em pacientes com dor torácica aguda e ECG não diagnóstico, o uso da ecocardiografia com contraste aumenta a sensibilidade para o diagnóstico da SCA.^310^

Pacientes com perfusão e função miocárdica normais ao repouso têm bom prognóstico, enquanto a presença de defeitos de perfusão ao repouso identifica um subgrupo de alto risco para a SCA.^306^^,^^311^

Os agentes de contraste ecocardiográfico são soluções contendo microbolhas de gás do tamanho das hemácias, cuja interface com o meio líquido é altamente refringente, melhorando o sinal ecocardiográfico do meio que as contém. Por este método, à beira de leito, pode-se avaliar a microcirculação miocárdica, pois as microbolhas são marcadores microvasculares que se comportam como as hemácias, portanto, não atingem áreas de obstrução microvascular. Desta forma, áreas com infarto não apresentam este contraste e são facilmente detectadas pela ecocardiografia.^306^ Por meio dessa modalidade ecocardiográfica, obtém-se simultânea e instantaneamente as alterações da contração segmentar do VE e da perfusão miocárdica.^312^

O contraste ecocardiográfico possibilita uma melhor definição das margens endocárdicas, permitindo uma avaliação mais adequada do espessamento miocárdico parietal e das funções contrátil global e segmentar do VE em repouso e sob estresse. Adicionalmente, os agentes de contraste permitem medida mais acurada dos volumes ventriculares e da FEVE, principalmente em casos de imagens subótimas, e têm comprovada utilidade na definição de alterações da anatomia.^306^^,^^313^

Defeitos regionais de perfusão transitórios extensos durante o estresse são indicativos de DAC grave. Respostas de melhora de contração segmentar em áreas dissinérgicas e com perfusão presente, durante doses iniciais da infusão de dobutamina (5 a 10*µ*g/kg/min), identificam viabilidade miocárdica nessas regiões “atordoadas” pela isquemia pregressa.

Em suma, em pacientes com dor torácica atendidos no centro de emergência, a ecocardiografia contrastada em repouso adiciona dados de diagnóstico e prognóstico a esses pacientes. Se o preenchimento completo do miocárdio pelo contraste (perfusão) for observado dentro de 4 segundos, isso identifica pacientes com motilidade e perfusão normais, portanto, de baixo risco. Já quando a perfusão é normal, mas há alteração segmentar do movimento de parede, identifica miocárdio atordoado, enquanto um defeito fixo de perfusão mapeia toda a área em risco durante a lesão isquêmica aguda.^314^^,^^315^ Nos pacientes com IAM, a ecocardiografia sob estresse com contraste miocárdico identifica não somente as áreas de infarto ou de atordoamento em repouso, mas pode identificar as áreas isquêmicas à distância pela análise das alterações transitórias da motilidade e perfusão miocárdicas.

###### 11.2.3.1. Avaliação da perfusão miocárdica pelo ecocardiograma | Sumário de recomendações e evidências

**Table:** 

Perfusão miocárdica pelo ecocardiograma - Sumário de recomendações e evidências		
Ecocardiografia transtorácica contrastada para melhora do sinal Doppler em pacientes com imagem subótima ou ecocardiografia transtorácica contrastada para delineamento de margens endocárdicas durante a ecocardiografia sob estresse em pacientes com imagens subótimas em repouso.	IIa	B
Ecocardiograma sob estresse com microbolhas em pacientes com risco intermediário nos quais persistem dúvidas após a realização de teste ergométrico.	IIb	B
Ecocardiograma sob estresse com microbolhas em pacientes com alto risco.	III	C

#### 11.3. Métodos de Cardiologia Nuclear

A cintilografia miocárdica de perfusão (CMP) é indicada fundamentalmente aos casos de impossibilidade de realização do teste ergométrico e a pacientes nos quais há dificuldades para a interpretação adequada do ECG de esforço. Uma das principais indicações da CMP é a possibilidade da realização das técnicas não invasivas mais utilizada no cenário da AI de baixo risco. Permite avaliar os pacientes incapazes de realizar esforço físico por meio do estresse farmacológico e apresenta melhor correlação com os dados anatômicos em comparação com o teste ergométrico.

A CMP e a ventriculografia nuclear radioisotópica têm um grande valor diagnóstico e prognóstico nas coronariopatias aguda e crônica.^316^^-^^318^ Uma das principais indicações da CMP é a possibilidade da realização precoce nas SCA, com ampla margem de segurança, empregando-se agentes vasodilatadores como o dipiridamol e a adenosina. Em relação a outros exames, a CMP mostrou-se superior nessa aplicação. Deve ser ainda destacada a possibilidade de, sincronizando-se o estudo cintilográfico tomográfico com o ECG (*gated*-SPECT), avaliar-se a função sistólica regional e medir-se a FE ventricular com exame único.^319^ Os vários estudos são consistentes na demonstração de que pacientes com diagnóstico de AI que apresentem cintilografia normal durante estresse pertencem a subgrupo com risco notadamente reduzido de eventos graves, de cerca de 1% em 1 ano; enquanto a detecção de defeitos reversíveis expressa prognóstico desfavorável, com taxa de eventos da ordem de 20% para o mesmo prazo de seguimento.^320^^-^^323^

A tomografia por emissão de pósitrons (PET; *positron emission tomography*) é uma técnica não invasiva de doença coronariana obstrutiva que apresenta acurácia bastante elevada para detecção de isquemia miocárdica e viabilidade miocárdica. Sua utilidade foi demonstrada para avaliação diagnóstica e prognóstica da DAC. Além disso, possibilita a avaliação muito precisa da reserva do fluxo coronariano por quantificação absoluta. Essa informação pode ser útil na avaliação de quadro de infarto em pacientes sem obstruções coronárias significativas (MINOCA).^324^^,^^325^ No Brasil, temos dificuldade para obter os radiotraçadores (rubídio e amônia) que permitem essa quantificação, mas novas gamacâmaras de detectores de cádmio-zinco-telúrio (CZT) também têm mostrado possibilidade de realizar quantificação da reserva de fluxo com traçadores de tecnécio.^326^

**Table:** 

Cintilografia miocárdica de perfusão - Sumário de recomendações e evidências		
Em pacientes com risco intermediário, nos quais persistem dúvidas após a realização de TE, ou impossibilitados de submeter-se ao TE.	I	B
Para identificação da presença/extensão de isquemia em pacientes que não podem realizar cateterismo, ou quando os resultados deste não são suficientes para o estabelecimento de condutas.	I	B
Após o cateterismo, para identificação da artéria relacionada com o evento (região a ser revascularizada), e / ou estratificação complementar de risco.	I	A
Em pacientes com regiões ventriculares dissinérgicas, em que se torna necessário comprovar ou excluir a presença de miocárdio viável para guiar a conduta terapêutica.	I	A
Como primeira opção na avaliação de isquemia, mesmo em pacientes em que o TE seja factível e interpretável.	IIb	B
Angiocardiografia nuclear em pacientes de risco intermediário e alto para identificação de envolvimento do ventrículo direito.	IIa	C
Em pacientes de alto risco antes das primeiras 48h de estabilização do paciente.	III	C

#### 11.4. Ressonância Magnética Cardiovascular

A ressonância magnética cardiovascular (RMC) é um método bastante útil para a avaliação cardíaca morfológica e funcional, fornecendo informações diagnósticas e prognósticas importantes tanto na doença isquêmica quanto nas miocardiopatias não isquêmicas. O exame é capaz de fornecer informações precisas sobre os aspectos morfológicos do coração, a quantificação dos volumes, a massa e a função ventricular global e regional, a avaliação de isquemia miocárdica (pela análise da contratilidade segmentar sob estresse com dobutamina e sem uso de contraste ou pela técnica de perfusão miocárdica sob estresse com vasodilatadores, como o dipiridamol e a adenosina, e com uso de contraste com base em gadolínio) e a avaliação da fibrose/necrose miocárdica pela técnica do realce tardio miocárdico.^327^ Todas essas informações são fornecidas de maneira integrada em um único exame. O método ainda permite, com a combinação destas e de outras formas de aquisição de imagem, a visualização do pericárdio e dos grandes vasos e a análise de fluxos e função valvar.

Vários estudos clínicos e metanálises mostram que a ressonância cardíaca é um método altamente acurado para a detecção de isquemia miocárdica, mesmo em comparação com o ecocardiograma de estresse,^328^ a cintilografia miocárdica^329^^,^^330^ ou a reserva de fluxo fracional (*fractional flow reserve -* FFR).^331^

A avaliação da viabilidade miocárdica pela técnica do realce tardio também já foi estudada e validada nas SCA e fornece importantes informações diagnósticas e prognósticas, além de ser considerada por muitos o padrão-ouro para a avaliação de viabilidade miocárdica e detecção de infartos.^332^^-^^336^ Estudos uni e multicêntricos demonstraram que a RMC é uma técnica extremamente sensível para detecção e localização do infarto, mesmo os pequenos infartos subendocárdicos,^337^^-^^339^ que também têm importância prognóstica demonstrada, talvez pela sua frequente associação a estenoses coronárias críticas e que podem passar despercebidos tanto à avaliação clínica quanto a outros exames diagnósticos.^340^^-^^342^ Adicionalmente, a medida da área infartada obtida pela RMC tem grande impacto prognóstico independente de outros fatores clínicos, tendo sido, em alguns estudos, mais importante que a própria FEVE.^343^^,^^344^ Utilizando técnicas de identificação de zonas de edema associadas à mensuração das zonas de realce tardio, pode-se obter também a taxa de miocárdio preservado, elemento que tem valor prognóstico, em especial em pacientes submetidos a tratamento trombolítico.^345^

A técnica do realce tardio também permite a detecção de áreas de hipossinal (áreas escuras) no meio da área de hipersinal (infarto), o que se relaciona com áreas de obstrução microvascular (*no-reflow phenomenon*) e também acrescenta informação prognóstica nessa população.^343^^-^^347^

Além dessas aplicações, a ressonância é bastante útil na diferenciação das miocardiopatias isquêmicas das não isquêmicas,^327^^,^^348^ sendo utilizada para o diagnóstico de miocardite^349^^,^^350^ e síndrome de Takotsubo.^351^^,^^352^ Ademais, na situação de elevação de marcadores de necrose miocárdica e cateterismo “normal”, a RMC pode confirmar a presença do infarto, que poderia estar relacionado com espasmo ou síndromes trombofílicas, entre outros. Outros diagnósticos diferenciais em que a RMC pode ajudar são a hipertrofia ventricular esquerda (primária da cardiomiopatia hipertrófica ou secundária) e a pericardite aguda isolada, sem miocardite associada.

Pela sua sensibilidade em encontrar áreas de infarto e de realizar o diagnóstico de outras anormalidades cardíacas, a ressonância tem sido preconizada para a avaliação de pacientes com quadro de infarto em pacientes sem obstruções coronárias significativas (MINOCA). Nesta condição, o exame permitiu a diferenciação correta entre processos inflamatórios e necrose isquêmica real, mesmo na ausência de placas de ateroma significativa, e pode fornecer dados fundamentais para a condução desses casos. Tal abordagem pode ser particularmente relevante no caso de mulheres que apresentam maior incidência deste evento.^353^

Nos casos de DAC confirmada, a RMC pode ainda ajudar a definir a coronária relacionada com o infarto em casos nos quais este achado não é claro no cateterismo, como, por exemplo, em pacientes com lesões triarteriais graves e ECG inespecífico. Nessa situação, a RMC pode ainda diferenciar infartos antigos de infartos recentes, relacionados com o quadro atual. Finalmente, a RMC pode fornecer imagens de alta resolução para a avaliação de complicações mecânicas pós-infarto, como ruptura do VE, insuficiência mitral, extensão do infarto para o ventrículo direito e comunicação interventricular (CIV).^340^^,^^354^

**Table:** 

Ressonância magnética cardiovascular - Sumário de recomendações e evidências		
Na avaliação da função ventricular, da presença/extensão da área de necrose e viabilidade.	I	A
Na pesquisa de eventuais alterações mecânicas.	I	A
Avaliação de pacientes com apresentação clínica de SCA, sem obstruções coronárias significativas.	I	A
No diagnóstico diferencial de pacientes que apresentam quadro clínico compatível com coronariopatia aguda, porém com ECG apresentando alterações inespecíficas e marcadores bioquímicos de necrose negativos.	IIa	B
Como adjuvante no diagnóstico de SCA, principalmente nos pacientes com probabilidade intermediária ou alta.	IIb	B

### 12. Cinecoronariografia e Avaliações Coronárias Intravasculares (FFR, IVUS, OCT)

A coronariografia comumente utilizada para diagnosticar e definir o tratamento para os pacientes com IAMCSST pode apresentar dificuldades em mostrar a lesão culpada na AI e no IAMSSST. Tal dificuldade ocorre especialmente porque este exame não é 100% sensível às rupturas de placas.^355^ O fator desencadeador das SCA vai desde doença obstrutiva aterosclerótica (que pode ser incipiente, com placa rota ou não), espasmo, embolia, hematoma coronário intramural, dissecção espontânea da coronária até dissecção aórtica.

Barcarawi et al. demonstraram, em uma metanálise recente, que, para os pacientes com SCASSST, a abordagem invasiva precoce de rotina resultou em uma menor incidência de eventos cardiovasculares maiores (definidos por cada um dos estudos incluídos) quando comparada com a estratégia invasiva tardia (RR 0,65, IC 95% 0,49-0,87; p = 0,003), sendo que este benefício persistiu apenas em pacientes com um escore GRACE > 140.^356^ Contudo, nesse estudo, não houve diferenças significativas na mortalidade por todas as causas, mortalidade cardiovascular, infarto do miocárdio ou eventos hemorrágicos entre os grupos. O tratamento invasivo precoce também foi associado a menor risco de desfechos isquêmicos no estudo SWEDEHEART.^357^

O rumo terapêutico a ser tomado, especialmente o momento da avaliação invasiva, depende não só do diagnóstico anatômico e funcional mas também das comorbidades, apresentação clínica, fragilidade, estado cognitivo e expectativa de vida do paciente e especialmente da estratificação de risco, pois quando se trata de pacientes de “muito alto risco”, esses casos devem ser avaliados rapidamente, em menos de 2h, sendo em até 72h para os pacientes de “risco intermediário”, o que é apresentado no Tabela 2.1.^358^

**Tabela 2.1:** Seleção da estratégia de tratamento da SCASSST de acordo com a estratificação de risco inicial

Avaliação invasiva na SCASSST		
Muito alto risco	Alto risco	Risco intermediário
Instabilidade hemodinâmica ou choque cardiogênico	Troponina positiva	DM ou insuficiência renal
Angina recorrente ou persistente refratária ao tratamento clínico	Alteração dinâmica ST/T	ICC ou FEVE < 40%
Arritmia ventricular maligna ou PCR	GRACE >140	Angina pós-IAM
Complicações mecânicas		ICP ou CRVM prévios
IC aguda		GRACE 109-140 ou sintomas recorrentes ou teste funcional positivo
Alterações dinâmicas ST/T recorrentes		
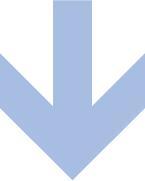	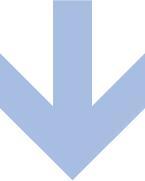	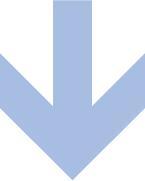
Invasiva imediataIC	Invasiva precoce(menor que 24h)IA	Invasiva(menor que 72h)IA

Fonte: adaptada de Neumann FJ, et al.^358^

CRVM: cirurgia de revascularização do miocárdio; DM: diabetes melito; GRACE: Global Registry of Acute Coronary Events; ICP: intervenção coronária percutânea; PCR: parada cardiorrespiratória; IAM: infarto agudo do miocárdio; FEVE: fração de ejeção de ventrículo esquerdo.

A estratégia invasiva permite o diagnóstico da DAC subjacente, a identificação da lesão, a orientação para o tratamento com medicamentos antitrombóticos e a avaliação da anatomia coronariana para ICP ou CRVM, pois até 40% desses pacientes com SCASSST demonstram múltiplas placas com morfologia complexas, sendo que até um quarto dos pacientes apresenta uma coronária com oclusão aguda.

A Sociedade Europeia de Cardiologia e a Associação Europeia de Cirurgia Cardiotorácica (ESC/EACTS) consideram a FFR o atual padrão diagnóstico para a avaliação funcional da gravidade da lesão em pacientes com estenose de grau intermediário (tipicamente em torno de 40% a 50%) sem evidência de isquemia em testes não invasivos ou naqueles com doença multiarterial. Nesta Diretriz, a introdução da razão instantânea de ondas livres (iFR), uma nova medida que não requer hiperemia induzida por adenosina, está incluída na recomendação como classe IA. As diretrizes consideram o FFR e o iFR como equivalentes.

Para a doença do tronco da coronária esquerda (TCE), a avaliação funcional por FFR ou iFR pode ser tecnicamente complexa, e as evidências que sustentam seu uso nesse cenário são escassas. Consequentemente, a ultrassonografia intracoronária (USIC) é uma recomendação da classe IIaB e a revascularização deve ser excluída quando a área luminal mínima for >6mm^2^. Para todas as lesões fora do TCE, a avaliação funcional é preferível à imagem intracoronariana. O uso do USIC e da tomografia de coerência óptica (OCT; *optial coherence tomography*) é recomendado para otimizar o implante de *stent* (classe IIaB).

A avaliação da FFR necessita da administração de adenosina para obter hiperemia máxima e estável, diferente da razão instantânea de onda livre (iwFR), que avalia os índices de repouso derivados apenas dos gradientes de repouso, ou seja, da pressão coronariana distal para aórtica (Pd/Pa). O estudo iFR-SWEDEHEART, que avaliou a revascularização guiada por iFR, tinha 17,5% dos pacientes com SCA no momento desta avaliação e demostrou que esta estratégia foi considerada não inferior à estratégia de revascularização guiada por FFR em relação à taxa de eventos cardíacos adversos maiores e eventos até 12 meses após o procedimento.^359^

O estudo DEFINE–FLAIR,^360^ que avaliou 2.492 pacientes com DAC, mostrou que a revascularização coronariana guiada pelo iRF foi não inferior à revascularização guiada pelo FFR com relação ao risco de eventos cardíacos adversos maiores em 1 ano, ainda com o benefício que o iFR foi mais econômico quando comparado com o FFR. O ponto de corte para definir uma lesão como hemodinamicamente significativa foi: iFR ≤ 0,89 e FFR ≤ 0,8.

A OCT utiliza luz infravermelha para fornecer imagens *in vivo* transversais de alta resolução da artéria coronária. Esta técnica de imagem permite uma avaliação detalhada da morfologia da placa em pacientes com SCA e pode ajudar a entender os mecanismos subjacentes, incluindo ruptura ou erosão da placa e calcificação. Portanto, é útil para otimizar a intervenção coronária percutânea e avaliar a resposta vascular à intervenção coronária e terapia farmacológica.^361^

Ao identificar trombo e delinear a ruptura ou erosão da placa, a OCT é útil na identificação de lesões e os mecanismos subjacentes na SCA, especialmente quando as lesões são ambíguas na angiografia.^362^

Pacientes com quadro de SCA, lesão única na artéria culpada sem doença difusa no mesmo vaso considerado responsável pela SCA e com indicação de angioplastia e implante de *stent* foram avaliados pelo estudo DOCTORS.^363^ Esse estudo foi desenhado com o objetivo de avaliar a eficácia da OCT na otimização da intervenção coronária percutânea entre pacientes com SCASSST e demonstrou que a subexpansão do *stent* foi comum, aproximadamente 42%, e foi um preditor independente de resultados adversos. Concluiu afirmando que a intervenção com OCT resulta em um maior benefício funcional, avaliado por FFR após a intervenção, em comparação à intervenção guiada por angiografia de rotina nesses pacientes com diagnóstico de SCASSST.

Apesar de os dados de longo prazo demonstrarem melhor resultado para tratamento da DAC com o auxílio da avaliação fisiológica, o FFR e o iFR permanecem subutilizados na prática atual.^364^ Mesmo assim, avanços adicionais em outras abordagens alternativas de FFR, incluindo TC coronariana não invasiva (FFRCT), angiografia invasiva (angioFFR) e tomografia de coerência óptica (FFROCT), já estão sendo realizados guiados por algoritmos de inteligência artificial e ferramentas robustas que permitem a intervenção detalhada pré-procedimento.

**Table:** 

Estudo hemodinâmico e cineangiocoronariográfico - Sumário de recomendações e evidências		
Nos pacientes de muito alto risco, realização da cineangiocoronariografia imediata.	I	A
Nos pacientes de alto risco, realização da cineangiocoronariografia precoce (em até 24h).	I	A
Nos pacientes de risco intermediário ou que apresentam recorrência de sintomas, ou ainda que têm teste não invasivo positivo para isquemia, realização da cineangiocoronariografia em até 72h.	I	C

### 13. Revascularização miocárdica (cirurgia de revascularização miocárdica e intervenção coronária percutânea)

A diferença entre o tratamento da AI e do IAMSSST em relação à DAC crônica é a decisão rápida da indicação de revascularização, evitando assim complicações cardiovasculares.^365^^-^^367^ A estratificação de risco é fundamental para a escolha adequada do tratamento conservador ou invasivo. Os escores mais usados são o TIMI e GRACE.^28^^,^^368^

Na escolha do tratamento, a angiografia coronária determina a estratégia anatômica, cuja revascularização deve ser imediata nos casos de angina refratária e instabilidade elétrica ou hemodinâmica. Além disso, as abordagens mais precoces têm desfechos favoráveis e estão indicadas nos pacientes de alto risco.^28^^,^^368^^-^^370^ Em uma coorte de 363.500 pacientes diabéticos com IAMSSST, a estratégia de avaliação invasiva precoce foi utilizada em 164.740 pacientes (45,3%). Em uma análise ajustada por escore de propensão, 21.681 diabéticos foram pareados em cada braço. Houve uma associação de menor mortalidade intra-hospitalar com a estratégia invasiva para o IAMSSST, mas não para a AI (2,2% *vs.* 3,8%; RR 0,57, IC 95%; 0,50-0,63, p < 0,01).^371^

As estratégias invasivas incluem a angioplastia ou a cirurgia de revascularização miocárdica.^372^ Uma ferramenta fundamental para esta decisão é baseada da anatomia coronária utilizando-se o SYNTAX Score.^373^ Pacientes com SYNTAX Score >22 (intermediário ou alto) têm maior benefício a longo prazo com a revascularização cirúrgica.^374^^,^^375^

Aproximadamente 5% a 10% dos pacientes com SCASSST necessitam de CRVM^376^ e representam um subgrupo desafiador devido às suas características de alto risco em comparação com pacientes submetidos à revascularização miocárdica eletiva.^377^

Em pacientes com isquemia em curso ou instabilidade hemodinâmica com indicação de CRVM, a cirurgia deve ser realizada o quanto antes, e não adiada como consequência da exposição ao tratamento antiplaquetário.

Na ausência de dados randomizados, o tempo ideal para revascularização miocárdica deve ser determinado individualmente. O risco de eventos isquêmicos, possivelmente relacionados à terapia antiplaquetária subótima enquanto aguarda a cirurgia é < 0,1%, enquanto o de complicações hemorrágicas perioperatórias associadas a inibidores de plaquetas é > 10%.^378^

Se o quadro clínico do paciente possibilitar a espera da cirurgia, sugere-se que a antiagregação plaquetária seja suspensa em tempo hábil. Dependendo do tipo e da dosagem do agente, deve-se aguardar a redução do seu efeito, por exemplo, 5 dias antes do procedimento cirúrgico para o clopidogrel, 5 dias para o ticagrelor e 7 dias para o prasugrel. Nesta situação, o tempo de espera para a cirurgia poderia ser abreviado testando-se a agregação plaquetária diariamente para verificar se o paciente recuperou ou não a inibição e liberá-lo com segurança para a cirurgia. Um estudo brasileiro está testando esta hipótese (NCT 02516267).

Atualmente, o manuseio cirúrgico na coronariopatia aguda sem supradesnivelamento do segmento ST é semelhante ao da doença estável. Portanto, após discussão com o Heart Team e indicação cirúrgica apropriada, o mais importante seria avaliar o momento ideal para a realização do procedimento. Isso estaria mais relacionado com a repercussão sistêmica do evento agudo em relação à otimização ao quadro clínico do paciente, na procura pelos melhores resultados. Isso significa que pacientes poderiam ir para cirurgia na mesma internação de forma imediata (urgência/emergência), aguardar a redução da ação de antiagregantes plaquetários ou obter alta hospitalar com data agendada para a cirurgia.

Quando há urgência ou emergência, dá-se preferência ao uso de enxertos venosos aos arteriais. A cirurgia poderá ser realizada com ou sem o auxílio da circulação extracorpórea, de acordo com as condições técnicas pertinentes a cada caso. Em pacientes em vigência de choque cardiogênico, a revascularização completa por meio de procedimento de angioplastia constitui-se como opção inicial; porém, diante de sua limitação, a cirurgia poderá ser indicada de acordo com avaliação multiprofissional.^358^

Os pacientes operados em 1 a 2 dias e 3 a 7 dias após o IAM tiveram uma taxa de mortalidade semelhante, sugerindo que, para alguns pacientes, seria possível reduzir o intervalo IAM-CRVM, sem comprometimento dos resultados. Nos pacientes operados no primeiro dia após o IAM, observou-se maior índice de mortalidade.^379^

Estudo realizado em pacientes acima dos 80 anos, portadores de IAMSSST e AI realizado em 16 centros na Noruega, denominado After Eighty Study, foram randomizados para a estratégia invasiva (ATC ou CRVM + tratamento clínico otimizado) *versus* estratégia conservadora com tratamento clínico otimizado isolado. Os desfechos maiores ocorreram em 93 pacientes (40,6%) de 229 pacientes no grupo de estratégia invasiva e em 140 (61,4%) de 228 pacientes no grupo da estratégia não invasiva (RR 0,53 [IC 95% 0,41-0,69], p = 0,0001). Não houve diferença quanto as complicações hemorrágicas entre os dois grupos.^380^

Estudo multicêntrico com 1.810 casos com IAM sem supra de ST e AI, dentro das 48h do início da dor, comparou também a estratégia invasiva com a conservadora. No primeiro ano, não houve diferença quanto aos desfechos primários de infarto e morte. Porém, em 5 anos de evolução, a estratégia invasiva teve um índice significativamente menor desses desfechos.^381^

Uma revisão sistemática incluindo 8.915 pacientes, sendo 4.545 submetidos à estratégia invasiva e 4.370 a uma estratégia conservadora, também observou a redução de mortalidade com a estratégia invasiva no período de 6 a 12 meses.^382^ Técnicas mais avançadas auxiliam a estratificação de risco e a abordagem terapêutica dos pacientes, como a fração de reserva de fluxo (FFR) e o ultrassom intracoronário.^383^^-^^385^

**Table:** 

Revascularização miocárdica (cirurgia de revascularização miocárdica e intervenção coronária percutânea) - Sumário de recomendações e evidências		
ICP ou CRVM nas lesões de múltiplos vasos, de acordo com o quadro clínico e o Escore SYNTAX.	I	B
Revascularização (ICP) de rotina em lesões não relacionadas ao IAM no choque cardiogênico.	III	B

## Parte 3 – Recomendações na Alta e Cuidados Pós-Alta Hospitalar

### 1. Mudança de Estilo de Vida

A prevenção secundária é parte fundamental no cuidado de pacientes pós-SCA. Esta etapa compreende mudanças no estilo de vida, reabilitação cardiovascular, educação quanto aos fatores de risco e promoção da melhor adesão ao tratamento. Apesar de eficácia comprovada na prevenção de desfechos cardiovasculares, sua implementação ainda é subótima.^386^^,^^387^ Pacientes e familiares devem ser orientados de forma clara e compreensível quanto aos benefícios comprovados pelas evidências científicas.^388^^-^^390^ Aumentar a abrangência da prevenção secundária é importante objetivo nesta população de pacientes.

Orientação adequada deve ser dada ao paciente quanto ao retorno às suas atividades laborais e sexuais e quanto à capacidade de conduzir veículos, trabalhando a reinserção do paciente paulatinamente à sua rotina. Além disso, atenção especial deve ser dispensada às questões psicossociais e socioeconômicas do paciente, incluindo atenção ao risco de depressão e isolamento social.^391^^-^^398^

#### 1.1. Cessação do Tabagismo

Este item ocupa destaque especial na mudança de estilo de vida do paciente. O abandono do tabagismo é medida muito efetiva para a redução da mortalidade em pacientes pós-SCASSST.^399^ O cigarro tem cerca de 7.000 componentes químicos, dos quais muitos interferem diretamente nas doenças cardiovasculares, além de 69 deles serem carcinogênicos.^400^ O cigarro interfere no sistema cardiovascular de várias formas, aumentando a frequência cardíaca e a demanda de oxigênio pelo miocárdico, além de diminuir a sua oferta.^401^ Aumenta a incidência de morte súbita,^402^ o risco de trombose, inflamação, vasoconstrição e aumento da oxidação do LDL colesterol.^403^ Estima-se que o risco de fibrilação atrial aumente 1,5 a 2 vezes entre os tabagistas.^404^

Em pacientes após SCASSST, o encaminhamento a um programa de cessação do tabagismo e a utilização de agentes farmacológicos, incluindo nicotina (adesivos ou goma), são comprovadamente úteis.^405^ Em estudo observacional com 12.656 pacientes, Parasuraman et al. identificaram que pacientes tabagistas, comparados com ex-tabagistas e pessoas que nunca fumaram, foram submetidos à intervenção coronariana em idade mais precoce, e a intervenção esteve mais associada a uma SCA. Ex-tabagistas tiveram resultados semelhantes ao grupo de pessoas que nunca fumaram.^406^

A abordagem para abandono do tabagismo deve ocupar papel central na estratégia de reabilitação do paciente, o quanto mais precoce possível, sendo o ideal ainda na fase hospitalar. Familiares que residem na mesma casa do paciente também devem ser encorajados a participar de programas com vistas à cessação do tabagismo, reforçando, desta maneira, o empenho do paciente, além de diminuir o risco do fumo passivo.^407^

A estratégia de abordagem da dependência a nicotina consiste em quatro etapas. O primeiro passo é determinar a dependência. Utiliza-se mais comumente o teste de Fagerström (Tabela 3.1). As etapas seguintes consistem em aconselhamento, tratamento medicamentoso e comportamental e seguimento clínico.

**Tabela 3.1:** Questionário de tolerância de fagerström

Quantos cigarros você fuma por dia?
(0) menos de 11
(1) de 11 a 20
(2) de 21 a 30
(3) mais de 30
**2. Quanto tempo depois de acordar você fuma o primeiro cigarro?**
(0) mais de 60min
(1) entre 31 e 60min
(2) entre 6 e 30min
(3) menos de 6min
**3. Você tem dificuldade de ficar sem fumar em locais proibidos?**
(0) não
(1) sim
**4. O primeiro cigarro da manhã é o que traz mais satisfação?**
(0) não
(1) sim
**5. Você fuma mais nas primeiras horas da manhã do que no restante do dia?**
(0) não
(1) sim
**6. Você fuma mesmo quando acamado por doença?**
(0) não
(1) sim
Interpretação do resultado (soma dos pontos obtidos em cada questão): 0 a 2 pontos = muito baixa dependência física 3 a 4 pontos = baixa dependência física 5 pontos = média dependência física 6 a 7 pontos = elevada dependência física 8 a 10 = muito elevada dependência física

Fonte: adaptada de Ranney L et al.^407^

Para prevenir recaídas, é importante identificar as situações de alto risco e agir para enfrentá-las. As estratégias consistem, basicamente, em evitar, escapar, distrair e adiar. Deve-se evitar festas, ingestão de álcool ou cafeína, encontro com fumantes, pelo menos durante as primeiras semanas de parada.

O uso de medicações como bupropiona, vareniclina e a suplementação de nicotina podem aumentar as chances de abstinência, associadas a outras terapias como grupos de apoio e aconselhamento, psicoterapia e abstinência de álcool.

A bupropiona é um antidepressivo de ação ansiolítica que diminuiu os sintomas de abstinência ao desejo de fumar em vários estudos. Um deles, que comparou a cessação do tabagismo entre pacientes com tratamento de bupropiona 300mg/dia *versus* placebo, mostrou abstinência de 44,2% *versus* 9,2% em favor da bupropiona.^408^ Em levantamento da literatura, foi encontrado que a bupropiona parece ser mais efetiva em pacientes que abandonaram e/ou falharam no tratamento com a terapia de reposição de nicotina.^409^ Por outro lado, dois estudos em fumantes hospitalizados por eventos coronarianos agudos não encontraram aumentos na cessação do tabagismo em fumantes que receberam bupropiona *versus* placebo.^410^^,^^411^

A reposição de nicotina é tratamento eficaz e apresenta benefícios no abandono ao tabagismo. Em um estudo, o uso de alguma forma de reposição de nicotina levou a um aumento da abstinência em até 60% (RR: 1,60; IC 95%: 1,53 a 1,68) quando comparada ao placebo. O aumento da eficácia do tratamento ocorre na associação de modos de reposição de nicotina se comparado com apenas um modo (RR: 1,34; IC 95%: 1,18 a 1,51).^412^ A reposição de longa duração mais utilizada são os adesivos transdérmicos e as de curta duração são a goma de mascar, *sprays* nasais e inaladores, alguns ainda não disponíveis no Brasil.

A vareniclina é um agonista parcial do receptor nicotínico e, ao substituir a ação da nicotina, reduz a intensidade dos sintomas de abstinência. Sua ligação ao receptor reduz também os efeitos de recompensa e prazer associados ao cigarro, o que pode reduzir alguns dos efeitos da privação. Sua eficácia foi demonstrada em seis estudos clínicos envolvendo um total de 3.659 fumantes crônicos.^413^^-^^416^ A vareniclina duplicou as taxas de abstinência em relação ao placebo (RR: 2,24; IC 95%: 2,06 a 2,43).^417^ Estudo envolvendo mais de 8.000 fumantes comparou o uso de vareniclina, bupropiona, adesivos de nicotina e placebo, randomizados na razão de 1:1:1:1, e encontrou maior taxa de abstinência do grupo vareniclina, em 6 meses, do que todos nos outros grupos.^418^ Em um estudo randomizado em pacientes com coronariopatia crônica, o uso de vareniclina mostrou aumento da chance de abstinência contínua em 1 ano, em comparação com o placebo (OR: 3,14; IC 95%: 1,94 a 5,11).^419^ Dois estudos randomizados em fumantes com SCA encontraram taxas mais altas de abstinência contínua em 24 semanas e mais alta prevalência de abstinência em 52 semanas naqueles que tomaram vareniclina *versus* placebo.^420^^,^^421^ A vareniclina tem como uma de suas principais limitações o alto custo e a ocorrência de náusea como efeito colateral limitante em alguns pacientes.

A associação entre os métodos parece ser mais efetiva que o tratamento isolado. Em recente metanálise envolvendo quatro estudos, a associação entre vareniclina e bupropiona mostrou maiores taxas de abstinência em 6 meses quando comparada à vareniclina em monoterapia. Os efeitos eram mais observados nos tabagistas com alto grau de dependência e nos com alta carga tabágica. O benefício, entretanto, não se manteve no seguimento de 12 meses. O uso da associação aumentou significativamente os sintomas de ansiedade nos pacientes.^422^ Portanto, a associação entre os métodos parece ser segura e mais efetiva quando são utilizadas vareniclina ou bupropiona em associação com a terapia de reposição de nicotina.

A bupropiona e a vareniclina também parecem ser seguras para pacientes com doenças cardiovasculares. O achado de alguns estudos aumentando a ocorrência de eventos neuropsiquiátricos em usuários dessas medicações levou a Food and Drug Administration (FDA) a exigir um aviso em bula sobre esses riscos. Após novos estudos, esse risco foi reavaliado, sendo retirada a observação em bula pela própria FDA em 2016.^423^

Uma polêmica questão na abordagem do tabagista é a substituição do cigarro tradicional por cigarro com menor quantidade de nicotina ou por dispositivos vaporizadores de nicotina, estes últimos conhecidos como cigarros eletrônicos.^424^ Ambas as estratégias são classificadas apenas como potenciais controles de danos.

A avaliação dos cigarros com menor quantidade de nicotina apresentou pouca efetividade, visto que os tabagistas, em geral, compensam a menor concentração de nicotina do cigarro com o consumo maior de cigarros diários, ou outras formas de adição de nicotina.^425^ Já os dispositivos vaporizadores de nicotina, que aparentemente expõem o paciente a menor quantidade de agentes cancerígenos que o cigarro tradicional, tem por outro lado a grande variabilidade de combinações de substâncias tóxicas, além do explosivo aumento do uso por jovens, principalmente nos EUA.^426^ No Brasil, a venda desse tipo de dispositivo encontra-se proibida. Recentemente, várias mortes nos EUA foram relacionadas ao uso desses dispositivos, por problemas pulmonares agudos, advindos de substâncias inaladas. O número de doentes relacionados ao *Vaping* atingiu um total de 2.807 que necessitou de hospitalização, além de 68 mortes confirmadas pelo Center for Disease Control (CDC; Centro de Controle de Doenças dos EUA). Até o presente momento, não há evidências que suportem o uso dessas estratégias, sendo o uso de cigarros eletrônicos, em particular, potencialmente perigoso.

**Table:** 

Cessação do tabagismo - Sumário de recomendações e evidências		
Recomenda-se cessar o tabagismo e evitar exposição a ambientes com fumantes, tanto no trabalho quanto no lar.	I	A
Acompanhamento no longo prazo, encaminhamento a programas específicos ou farmacoterapia (incluindo reposição de nicotina) são úteis quando associados às clássicas estratégias não farmacológicas.	I	A
Uso de estratégias contra o tabagismo para controle de danos como cigarros com menor concentração de nicotina.	III	C
Uso de dispositivos vaporizadores de nicotina para controle de danos e estratégia para abandono do tabagismo em pacientes resistentes	III	B

#### 1.2. Recomendações Alimentares

Os pacientes que tiveram SCASSST classificam-se, obviamente, como de muito alto risco cardiovascular e, portanto, uma redução considerável nos valores do colesterol é absolutamente indispensável. Além da terapia com estatinas, que é suportada por diversas metanálises^427^^,^^428^ e estudos randomizados,^429^^-^^432^ a terapia dietética é ponto de central importância e traz expressivo número de evidências.^433^^,^^434^

É recomendada uma dieta padrão que enfatiza a ingestão de vegetais, frutas, grãos integrais, leguminosas, fontes de proteínas saudáveis (produtos lácteos, aves com baixo teor de gordura [sem pele], peixe/frutos do mar e nozes) e óleos vegetais não tropicais. Deve-se evitar a ingestão de doces, bebidas açucaradas e o próprio açúcar, além do excesso de carnes vermelhas.

O estudo PREDIMED testou o uso de uma dieta do tipo mediterrânea, mostrando redução em eventos cardiovasculares, especialmente AVC.^435^

Esse padrão alimentar deve ser ajustado para requisitos calóricos apropriados, padrões culturais, preferências alimentares e terapia nutricional para outras condições incluindo diabetes. A ingestão calórica deve ser ajustada para evitar ganho de peso e em caso de pacientes com sobrepeso/obesidade, para promover perda de peso. O uso de suplementos alimentares vitamínicos ou antioxidantes não deve ser prescrito de rotina para prevenção secundária.^436^

### 2. Reabilitação Cardiovascular

O exercício físico reduz a aterogênese, promove ação anti-inflamatória, melhora a função endotelial, diminui o tônus simpático, aumenta o HDL, reduz a pressão arterial e a resistência à insulina, dentre outros efeitos benéficos, não devendo ser negado ao paciente após SCA.^437^ Nos programas de reabilitação cardiovascular, a atividade física está inserida no contexto terapêutico. O sedentarismo é problema de ordem mundial e está entre os principais fatores de risco passíveis de intervenção na prevenção primária, sendo ainda mais importante no âmbito da prevenção secundária de eventos coronarianos.

O processo de reabilitação cardiovascular do paciente acometido por SCA inicia-se ainda durante a internação, na fisioterapia hospitalar, com o processo de deambulação precoce e a realização de movimentos passivos e ativos dos principais grupos musculares. Ainda durante o período da internação, o paciente deve ser orientado sobre escalas de percepção do esforço (p. ex., escala de BORG), que será útil na sua orientação após a alta.

Na ocasião da alta, é importante que todo paciente receba orientação adequada sobre a prática de atividade física que, inicialmente, no domicílio, deverá ser de leve intensidade, como continuidade aos exercícios que já vinham sendo realizados na fase intra-hospitalar (fase 1), e sobre como se monitorar. Deve ser indicada avaliação precoce em programa formal de reabilitação ou, quando não disponível, ser o paciente orientado sobre programa de exercícios que possam ser acompanhados pelo fisioterapeuta ou educador físico, de acordo com os limites propostos pelo médico (fase 2).

Alguns estudos demonstram que pacientes encaminhados a programas de exercício após um evento coronariano agudo cursaram com melhor qualidade de vida, menor recorrência de sintomas e de novos eventos cardiovasculares.^438^^-^^440^

Apesar dos efeitos positivos bem determinados dos programas de reabilitação, o encaminhamento sistemático e a aderência ainda são um grande desafio.^441^ Falta de educação médica continuada em reabilitação cardíaca, falta de conhecimento sobre a segurança dos programas (taxa de eventos de 1 para cada 112 mil pacientes/hora) e dificuldade de tornar esses programas custo-efetivos são parte importante do problema.

#### 2.1. Orientação sobre Atividade Física na Ocasião da Alta

O primeiro passo na avaliação do paciente para programa de reabilitação cardiovascular, após avaliação clínica e dos exames complementares disponíveis, será o de verificar se existe alguma contraindicação absoluta à prática de atividade física.^442^^,^^443^ A Tabela 3.2 enumera as contraindicações para a prática de atividade física regular.

**Tabela 3.2:** Classificação de risco para exercício em pacientes cardiopatas

Baixo risco	Risco moderado	Alto risco
FEVE > 50% Ausência de arritmias complexas Ausência de sintomas de insuficiência cardíaca congestiva Ausência de angina com o esforço ou no período de recuperação Ausência de lesão valvular grave ou moderada Capacidade funcional ≥7 METS	FEVE: 40% a 49% Sinais/sintomas, incluindo angina em níveis moderados de exercício (5 a 6,9 METS) ou no período de recuperação Lesão valvar moderada	FEVE < 40% Sobreviventes de parada cardíaca ou morte súbita Arritmias ventriculares complexas em repouso ou no exercício Doença valvular grave Cardiopatia congênita não corrigida Capacidade funcional <5 METSInfradesnível do segmento ST isquêmico durante exercício >2mm

FEVE: fração de ejeção do ventrículo esquerdo.

De posse dos dados clínicos com foco na ocorrência de angina, classificação funcional, função ventricular, presença de lesões coronárias residuais e de arritmias, segue-se com a classificação do risco para a prática de atividade física no cardiopata. Esta classificação é útil na determinação do nível de suporte a ser ofertado durante o programa e a necessidade de monitoramento durante as sessões de exercícios.^442^^,^^443^

Pacientes de baixo risco podem iniciar exercícios físicos em ambiente não necessariamente supervisionado pelo médico acompanhado pelo fisioterapeuta ou educador físico. Já os de risco moderado, se disponível, preferencialmente, devem iniciar o programa de exercícios em ambiente com supervisão médica e com recursos de suporte avançado de vida e evoluir para programa externo semissupervisionado, caso não apresentem intercorrências após 12 semanas de exercícios. Quando um programa de reabilitação cardiovascular formal e sob supervisão médica no local não é disponível, estes podem ser realizados sob supervisão do educador físico ou fisioterapeuta, obedecendo a limites predeterminados de segurança de acordo com a avaliação do cardiologista. O local e a equipe devem ter capacitação ao menos em suporte básico de vida e um desfibrilador externo automático disponível nas proximidades. Os pacientes de alto risco são preferencialmente conduzidos em ambiente sob supervisão médica, com equipe de fisioterapia e/ou educação física treinada em suporte básico de vida e com recursos locais para suporte avançado de vida.

Em todas as situações, o monitoramento da frequência cardíaca deve ser estimulado como guia da intensidade do esforço. A necessidade de monitoramento eletrocardiográfico é definida caso a caso pela equipe médica do programa de reabilitação.

As zonas de exercício de segurança podem ser definidas idealmente com base em uma prova funcional (teste cardiopulmonar ou teste ergométrico), ou, em casos específicos, podem ser guiadas apenas pela percepção do esforço. Os exercícios aeróbicos devem ser mantidos, em geral, com a frequência cardíaca entre a indicada pelo limiar anaeróbico e pelo ponto de compensação respiratória, quando disponível um teste cardiopulmonar (zona em que, apesar da produção de ácido láctico, não há acidose metabólica descompensada, relacionando-se assim a uma menor chance de eventos arrítmicos ou de sobrecarga cardiovascular). Quando um teste cardiopulmonar não for disponível, pode-se trabalhar entre 70% e 85% da frequência cardíaca (FC) máxima atingida em um teste ergométrico ou 50% a 80% da FC de reserva adicionada à FC de repouso, em que: FC de reserva = FC pico esforço – FC repouso. Nos pacientes que apresentem alteração eletrocardiográfica ou sintomas sugestivos de isquemia ao esforço, o limite deve estar, em geral, em 10 batimentos a menos que o de início do sintoma ou da alteração de ST. Em casos específicos e sob supervisão médica, o paciente pode ser treinado no limite de isquemia.

O médico deve idealmente determinar os seguintes parâmetros para o exercício:

- Frequência semanal.
- Intensidade (utilizando a escala de Borg e a frequência cardíaca ou a carga determinada em um teste cardiopulmonar ou no teste ergométrico).
- Modalidade (p. ex., caminhada, bicicleta, remo).
- Duração da sessão.
- Exercícios resistidos devem também ser estimulados. Recomendar 40% a 60% da contração voluntária máxima (baixa a moderada intensidade), 8 a 15 repetições, 1 a 3 séries.^443^^,^^444^

**Table:** 

Reabilitação cardiopulmonar - Sumário de recomendações e evidências		
Orientação sobre atividade física na ocasião da alta e programas de reabilitação cardíaca devem ser estimulados em todos os pacientes após SCA.	I	B
A intensidade e o grau de supervisão do programa de reabilitação devem ser estabelecidos de acordo com a classificação de risco do paciente.	I	B
Reabilitação cardiopulmonar nas seguintes condições (a menos que devidamente resolvidas): infarto agudo do miocárdio com <72h; angina instável em curso; arritmias complexas graves não controladas; insuficiência cardíaca descompensada; lesão de tronco de coronária esquerda grave instável ou com indicação de intervenção; hipertensão arterial descompensada (sistólica >190mmHg ou diastólica >120mmHg).	III	A

### 3. Medicamentos a Serem Prescritos por Ocasião da Alta Hospitalar

#### 3.1. Antitrombóticos

##### A. Troca de Antiplaquetários

A abordagem de como selecionar o segundo antiplaquetário a ser usado em conjunto com o AAS já foi feita previamente. Por ocasião da alta hospitalar, contudo, pode ser necessária a troca do segundo antiplaquetário por algum motivo, entre os quais:

- Custo.
- Efeitos adversos limitantes (p. ex., dispneia com o ticagrelor, sangramento em vigência dos fármacos mais potentes como ticagrelor e prasugrel).
- Preferência do paciente por usar medicações com tomada única diária (p. ex., prasugrel, se tratado com ICP e sem AVC/AIT prévio) em vez de duas vezes por dia (ticagrelor).

A transição de clopidogrel para ticagrelor foi a única estudada em trabalho com poder suficiente para avaliar desfechos clínicos, apesar de o estudo não ter sido desenvolvido com este objetivo. No estudo PLATO, 46% dos pacientes randomizados para receber ticagrelor haviam sido pré-tratados com clopidogrel (dose de 300 a 600mg).^152^ A segurança e a eficácia do ticagrelor não foram afetadas pelo uso prévio de clopidogrel.^248^ Já no estudo TRITON-TIMI-38, que avaliou o uso de prasugrel, a utilização prévia de outro inibidor P2Y_12_ foi considerada critério de exclusão para o estudo.^154^ As outras trocas entre inibidores P2Y_12_ (p. ex., entre ticagrelor e prasugrel ou de ticagrelor/prasugrel para clopidogrel) foram avaliadas por estudos de farmacodinâmica e por registros observacionais, os quais servem mais como geradores de hipótese, uma vez que não foram desenhados para avaliar desfechos clínicos da forma adequada.^445^^-^^449^ Dessa forma, existem menos evidências para embasar tais trocas.

**Figura 3.1:**
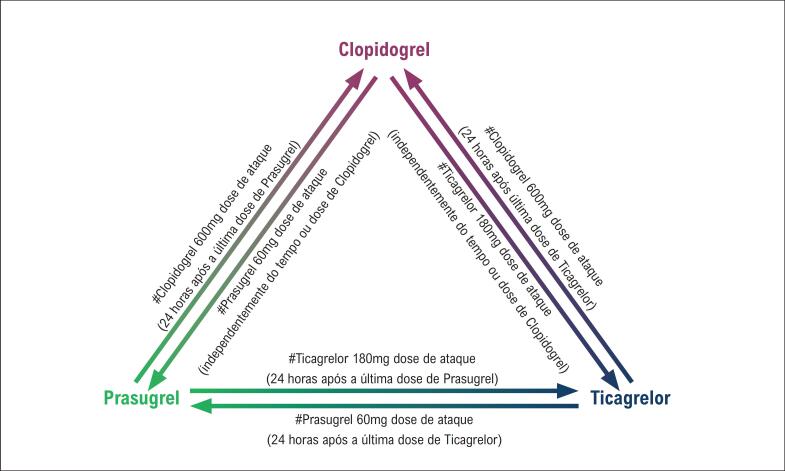
Recomendações em relação à troca de inibidores P2Y_12_ em pacientes nos primeiros 30 dias após síndrome coronariana aguda. Recomenda-se dose de ataque do novo agente de forma rotineira. *Se estiver havendo descalonamento da terapia com prasugrel ou ticagrelor para clopidogrel devido a sangramento ou a risco de sangramento aumentado, pode-se considerar fazer apenas a dose de manutenção do clopidogrel. Adaptada de Valgimigli et al.^451^

No paciente com SCA há menos de 30 dias, recomendamos que, caso seja necessária a troca entre agentes P2Y_12_, seja seguido o esquema da Figura 3.1.^450^^,^^451^

Assim, como regra geral, em todos cenários de troca de antiplaquetários P2Y_12_ ainda na fase aguda da doença coronariana (< 30 dias), deve-se utilizar dose de ataque da nova medicação, seguida de dose de manutenção. Exceção a esta regra se faz quando a mudança é em função de descalonamento da terapia com prasugrel ou ticagrelor, para clopidogrel, devido a sangramento ou de risco de sangramento aumentado.

Quanto ao horário de administração do novo fármaco, recomenda-se esperar 24h após a administração do primeiro medicamento. Exceção a esta regra se faz quando o medicamento a ser substituído é o clopidogrel.

**Figura 3.2:**
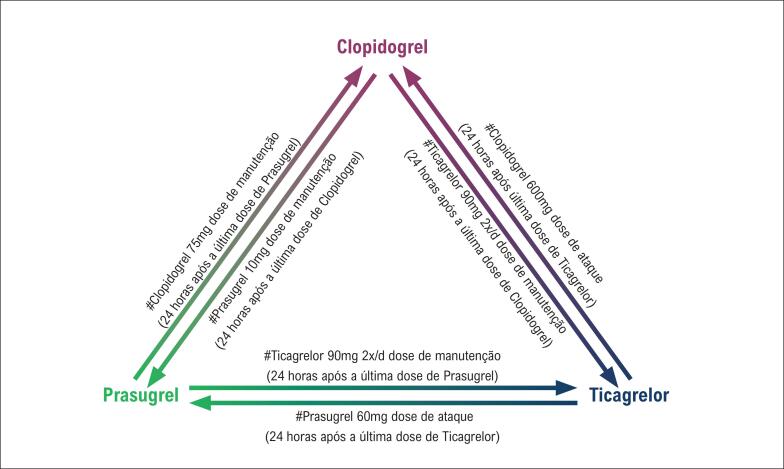
Recomendações em relação à troca de inibidores P2Y_12_ em pacientes após 30 dias de síndrome coronariana aguda. *Se estiver havendo descalonamento da terapia com ticagrelor para clopidogrel devido a sangramento ou a risco de sangramento aumentado, pode-se considerar fazer apenas a dose de manutenção do clopidogrel. Adaptado de Valgimigli et al.^451^

Nos casos em que o paciente tenha apresentado SCA há mais de 30 dias e seja necessária a troca entre agentes, recomenda-se o esquema da Figura 3.2.

Neste cenário, há algumas diferenças:

- Em todas as situações, recomenda-se aguardar 24h entre a última dose da medicação prévia para então iniciar-se a medicação nova.
- Dose de ataque do novo agente é recomendada somente quando o fármaco a ser substituído é o ticagrelor. Estudos clínicos demonstraram maior reatividade plaquetária nas primeiras 48h quando da transição de ticagrelor para clopidogrel^448^^,^^449^ ou para prasugrel.^447^ Essa diferença se deve ao fato de o ticagrelor ser um bloqueador reversível do P2Y_12_, enquanto os tienopiridínicos (clopidogrel e prasugrel) são bloqueadores não reversíveis.

**Table:** 

Mudança de inibidores P2Y_12_ - Sumário de recomendações e evidências		
Em pacientes admitidos com SCA nos quais foi administrado clopidogrel e que se deseja trocar para ticagrelor nos primeiros 30 dias, deve ser feita dose de ataque de 180mg deste, independentemente do momento e da dose do clopidogrel administrada.	I	B
Em relação às outras trocas entre inibidores P2Y_12_ orais, as mesmas devem ser consideradas em casos de efeitos adversos ou outros fatores, e seguir os esquemas recomendados por esta Diretriz.	IIb	C

##### B. Duração da Dupla Terapêutica Antitrombótica

###### B.1. Além de 12 Meses

####### B.1.1. Antiplaquetários

Os estudos que avaliaram o uso de clopidogrel, prasugrel e ticagrelor em pacientes com SCA mantiveram a dupla terapêutica antiplaquetária (DAP) por um tempo médio de 12 meses. Assim, este é o tempo padrão recomendado, independentemente da estratégia terapêutica adotada (tratamento clínico, angioplastia ou cirurgia de revascularização miocárdica). No caso de pacientes tratados clinicamente, as opções de antiplaquetários são apenas o ticagrelor e o clopidogrel, visto que o prasugrel não se mostrou superior ao clopidogrel neste cenário.^452^

O uso de DAP é uma forma muito eficaz de se prevenir trombose de *stent*. O risco de trombose de *stent* tardia (entre 1 mês e 1 ano da implantação do dispositivo) e muito tardia (> 1 ano após a angioplastia) vem diminuindo consideravelmente com o uso dos *stents* farmacológicos mais modernos. Assim, a manutenção de DAP além de 1 ano após evento de SCA e angioplastia parece carregar um aumento do risco de sangramento mais deletério do que o potencial benefício relacionado à prevenção de tromboses muito tardias de *stent*. Contudo, o estudo PEGASUS demonstrou que, em pacientes com histórico de infarto >1 ano e critérios de alto risco adicionais (diabetes, idade > 65 anos, doença renal crônica, doença coronária multiarterial ou dois ou mais infartos prévios), o uso prolongado de DAP com AAS e ticagrelor reduziu a incidência de desfechos isquêmicos, incluindo novos casos de infarto não relacionados com trombose de *stent* e de AVC, em relação à monoterapia com AAS (HR 0,84; IC 95% 0,74 a 0,95; p = 0,004 e HR 0,85; IC 95% 0,75 a 0,96; p = 0,008, para as doses de 60mg e 90mg, respectivamente).^453^ O uso da DAP além de 12 meses estaria, portanto, mais associado à proteção sistêmica contra eventos trombóticos do que à prevenção de tromboses mais tardias de *stent*. Tal argumento foi reforçado por subanálise do mesmo estudo em que os pacientes sem histórico de angioplastia com *stent* tiveram benefício similar na redução de eventos isquêmicos cardiovasculares quando comparados a pacientes com angioplastia prévia.^454^ A diminuição de desfechos cardiovasculares foi antagonizada por um aumento do número de sangramento maiores (HR 2,32; IC 95% 1,68 a 3,21; p < 0,001 e HR 2,69, IC 95% 1,96 a 3,70; p < 0,001, para as doses de 60 e 90mg, respectivamente). Não houve aumento de sangramento fatal ou de hemorragia intracraniana com o ticagrelor. O grupo que usou a dose mais elevada de ticagrelor (90mg, 2 vezes/dia) não teve benefícios adicionais em relação à redução do risco isquêmico e apresentou maiores taxas de sangramento. A dose de 60mg, portanto, é a aprovada para esta indicação, em casos selecionados de pacientes após 1 ano de IAMSSST que toleraram a DAP no primeiro ano.

O DAPT *trial* foi outro estudo que avaliou o uso de terapia antiplaquetária dupla por tempo superior a 12 meses.^455^ Todos os pacientes incluídos haviam sido submetidos à angioplastia com *stent* farmacológico e cerca de um terço teve a angioplastia realizada no contexto de uma SCASSST. Nesse estudo, os pacientes usavam DAP pelo tempo padrão de 12 meses e, caso tivessem sido bem aderentes aos antiplaquetários e não tivessem apresentado sangramentos relevantes, eram então randomizados para continuar com a terapia dupla por mais 18 meses ou continuar apenas com o AAS em associação a placebo. De forma similar ao PEGASUS, foi notada redução de eventos isquêmicos, mas este benefício foi contrabalanceado pelo aumento do risco de sangramento. Neste estudo, houve a redução de um caso de trombose de *stent* para cada 100 pacientes tratados com DAP prolongada, mas ao custo da ocorrência de um evento de sangramento moderado ou importante em cada 111 pacientes submetidos a este mesmo tratamento. No DAPT *trial*, os inibidores P2Y_12_ usados foram o clopidogrel (65,2% dos casos) e o prasugrel (34,8% dos pacientes).

Metanálise com > 32.000 pacientes antes da publicação do PEGASUS encontrou aumento significativo na incidência de infarto no grupo que usou DAP por 12 meses (em comparação com >12 meses OR = 1,57, IC 95% 1,30 a 1,90), com menor incidência de sangramento (OR = 0,65, IC 95% 0,52 a 0,81).^456^ Metanálise posterior, incluindo o PEGASUS, encontrou diminuição significativa de mortalidade por qualquer causa a favor do grupo com DAP por > 12 meses (HR = 0,89, IC 95% 0,79 a 0,99).^457^ Conclui-se assim que os três inibidores da P2Y_12_ disponíveis no Brasil (clopidogrel, ticagrelor e prasugrel) têm potencial em reduzir eventos isquêmicos quando usados associados a AAS por período superior a 12 meses, porém com aumento do risco de sangramento. Faz-se necessário, portanto, avaliar de formada individualizada o risco do paciente tanto em relação a eventos isquêmicos quanto em relação a sangramentos. Escores clínicos permitem estimar, com parâmetros mais objetivos, o risco isquêmico e o risco hemorrágico conforme as características clínicas do paciente. Podem, portanto, auxiliar na tomada de decisão quanto à duração da DAP.

O estudo PRECISE-DAPT (Predicting bleeding complications in patients undergoing stent implantation and subsequent dual antiplatelet therapy) avaliou 14.963 pacientes que haviam sido submetidos à angioplastia e gerou um escore constituído de cinco variáveis (idade, *clearance* de creatinina, hemoglobina, contagem de leucócitos e história de sangramento espontâneo) para predizer o risco de sangramento neste cenário.^458^ Além de ser avaliado nesta população, o escore foi validado em um grupo de mais de 14.000 pacientes. Pacientes com escore baixo (< 25) apresentaram benefício com a DAP prolongada, demonstrando redução do desfecho isquêmico composto (IAM, trombose definitiva de *stent*, AVC e necessidade de revascularização de órgão-alvo), com NNT de 65, sem aumentar significativamente o risco de sangramento. Por outro lado, em pacientes com escore alto (≥ 25), a DAPT prolongada foi associada a maior risco de sangramento (NNH 38), sem proporcionar benefício em relação à prevenção quanto a desfechos isquêmicos. Vale ressaltar, no entanto, que este escore foi derivado de estudos randomizados com ICP (com ou sem SCA), e não exclusivamente de uma população com SCA, em que o risco de eventos isquêmicos tende a ser maior comparado a pacientes com DAC estável.

O escore DAPT (Tabela 3.3) foi derivado de uma coorte do *trial* de mesmo nome.^459^ Nesta análise, pacientes com escore de risco alto (≥ 2) apresentaram importante redução de desfechos isquêmicos com o uso de DAPT por 30 meses (NNT de 34), à custa de um pequeno aumento no risco de sangramento (NNH de 272). Por outro lado, pacientes com escore baixo (< 2) não mostraram redução do risco isquêmico com a DAP prolongada e ainda evoluíram com aumento considerável no risco de sangramento (NNH de 64).

**Tabela 3.3:** Escore DAPT (Dual Antiplatelet Therapy) trial

Variáveis	Pontos
Idade ≥ 75 anos	–2
Idade entre 65 e 74 anos	–1
Idade < 65 anos	0
Tabagismo atual	1
Diabetes melito	1
IAM na apresentação inicial	1
ICP ou IAM prévio	1
Diâmetro do *stent* < 3mm	1
*Stent* eluído com paclitaxel	1
IC ou FEVE < 30%	2
ICP em enxerto de veia safena	2

IAM: infarto agudo do miocárdio; ICP: intervenção coronária percutânea; IC: insuficiência cardíaca; FEVE: fração de ejeção do ventrículo esquerdo.

####### B.1.2. Anticoagulantes

O estudo COMPASS incluiu > 27.000 pacientes com doença coronariana crônica para utilizarem rivaroxabana 2,5mg 2 vezes/dia + AAS 100mg 1 vez/dia (n = 9152) ou rivaroxabana 5mg 2 vezes/dia (n = 9117) ou AAS 100mg/dia (n = 9126). Em um prazo médio de acompanhamento de 23 meses, demonstrou superioridade do grupo rivaroxabana + AAS sobre AAS isolado (HR = 0,76, IC 95% 0,66 a 0,86 para o desfecho primário composto de óbito CV, IAM ou AVC), à custa de aumento na incidência de sangramento (HR = 1,70, IC 95% 1,40 a 2,05). Importante: houve diminuição significativa na incidência de óbito por qualquer causa (HR = 0,82, IC 95% 0,71 a 0,96) e ausência de diferença significativa na incidência de sangramento fatal ou intracerebral.^460^ Considerando apenas os pacientes incluídos nos grupos rivaroxabana + AAS e AAS isolado, 2.423 apresentavam história de IAM < 2 anos, 3.279 entre 2 e 5 anos, e 5673 > 5 anos. A comparação entre os grupos apresentou HR = 0,70 para pacientes com IAM < 2 anos, HR = 0,81 para aqueles com IAM entre 2 e 5 anos, e HR = 0,72 para aqueles com > 5 anos (P-interação = 0,93).^461^

###### B.2. Menos de 12 Meses

O uso de monoterapia com ticagrelor foi avaliado no estudo GLOBAL LEADERS^462^ em 15.968 pacientes submetidosàa ICP (34% com SCASSST). Os grupos recebiam AAS + ticagrelor por 1 mês, seguido de ticagrelor monoterapia por 23 meses, ou DAP padrão com AAS + clopidogrel (coronariopatia estável) ou AAS + ticagrelor (coronariopatia aguda) por 12 meses, seguido de monoterapia com AAS monoterapia por mais 12 meses. Ao final de 2 anos de seguimento, a meta principal do estudo (óbito por qualquer causa ou infarto) foi similar nos dois grupos (RR 0,87, IC 95% 0,75 a 1,01). A meta secundária principal (sangramento BARC 3 ou 5) também foi similar entre os grupos (RR = 0,97, IC 95% 0,78 a 1,20). Análise secundária em publicação posterior encontrou diminuição significativa na incidência da meta primária do estudo em pacientes com coronariopatia aguda e ICP em mais de uma coronária.^463^

Já o estudo TWILIGHT avaliou se, em pacientes submetidos à ICP e com características clínicas e angiográficas de alto risco de trombose ou sangramento, a descontinuação precoce de AAS seria segura.^464^ Foram incluídos 7.119 pacientes (4.614 com SCASSST), todos tratados por 3 meses após a ICP com AAS e ticagrelor. Após este período, foram randomizados para continuar a DAP ou para o uso de monoterapia com ticagrelor por mais 9 meses. O desfecho principal do estudo foi sangramento BARC 2, 3 ou 5. Ao final do seguimento, o grupo monoterapia com ticagrelor apresentou redução de 44% (HR 0,56; IC 95%: 0,45-0,68; p < 0,001) no desfecho principal do estudo, sem que isso fosse acompanhado de aumento no risco isquêmico (desfecho secundário, estudo sem poder amostral para esta conclusão).

O’Donoghue et al., em metanálise recém-publicada, analisando monoterapia com iP2Y_12_ *versus* DAP em aproximadamente 17.000 pacientes com coronariopatia aguda, encontraram HR de 0,50 (IC 95% 0,41 a 0,61) para sangramento, e de 0,85 (IC 95% 0,70 a 1,03) para MACE.^465^

**Table:** 

Duração de dupla terapêutica antitrombótica em pacientes com SCASSST em ritmo sinusal - Sumário de recomendações e evidências		
Após SCASSST, é recomendado que a DAPT seja mantida por 12 meses, independentemente da estratégia clínica adotada (angioplastia, cirurgia de revascularização miocárdica ou tratamento clínico).	I	A
Em pacientes com SCASSST e risco aumentado de sangramento, pode-se considerar manter o tempo de dupla antiagregação plaquetária por apenas 6 meses, suspendendo-se o inibidor P2Y_12_ após este período, independentemente da estratégia clínica adotada (angioplastia, cirurgia de revascularização miocárdica ou tratamento clínico)	IIa	B
Em pacientes com SCASSST submetidos à ICP, pode-se considerar manter a DAPT por 3 meses seguido de monoterapia com iP2Y_12_, (preferencialmente ticagrelor).	IIa	A
Associar uma segunda medicação antitrombótica (ver tabela abaixo) ao AAS após os 12 meses de DAP em pacientes com alto risco isquêmico e baixo risco de sangramento.	IIa	A

**Table:** 

Opções de terapia antitrombótica/antiplaquetária estendida			
Fármaco	Trial	Dose	Indicação
Clopidogrel	DAPT	75mg por dia	Pós-angioplastia que tolerou DAP por 1 ano
Prasugrel	DAPT	10mg por dia (5mg se peso < 60kg ou idade > 75 anos)	Pós-angioplastia que tolerou DAP por 1 ano
Ticagrelor	PEGASUS-TIMI-54	60mg 2 vezes/dia	Pós-infarto com risco isquêmico aumentado
Rivaroxabana	COMPASS	2,5mg 2 vezes/dia	Pacientes coronariopatas ou com doença arterial periférica com alto risco trombótico

Atenção especial deve ser dada quando da prescrição de antiplaquetários a pacientes admitidos com SCA que, durante a internação hospitalar, são submetidos à cirurgia de revascularização miocárdica. Subanálises dos estudos CURE,^466^ PLATO^249^ e TRITON^237^ mostraram que pacientes submetidos à revascularização cirúrgica parecem se beneficiar de forma similar aos outros indivíduos incluídos nestes *trials*. Dessa forma, o tienopiridínico deve ser reintroduzido assim que considerado seguro após a revascularização cirúrgica.

##### C. Manejo de Antiagregantes no Paciente que Necessita de Anticoagulação Cronicamente

A evidência que existe sobre o assunto se restringe praticamente a pacientes com fibrilação atrial (FA). A incidência de FA em pacientes com SCASSST varia de 5% a 23%.^467^ A terapia padrão para SCA é o uso de DAP por pelo menos 12 meses, com objetivo de prevenção de eventos isquêmicos. Em pacientes com FA e escore CHA_2_DS_2_VASC ≥ 2, é recomendado o uso de anticoagulação crônica, com objetivo de prevenção de eventos tromboembólicos. Quando um paciente apresenta essas duas situações associadas, surge a questão de qual a melhor combinação de antiagregantes e antitrombóticos, qual a melhor dose e qual a duração ideal de cada etapa do tratamento. Essas perguntas foram avaliadas em uma série de estudos clínicos de fase 3.

O primeiro desses estudos foi o trial WOEST,^468^ que recrutou 573 pacientes com FA em uso de anticoagulantes e que foram submetidos à angioplastia. Os pacientes foram então randomizados em dois grupos: um que usava clopidogrel associado ao anticoagulante e outro que usava terapia tripla (anticoagulante + clopidogrel + AAS). Observou-se que a terapia dupla foi associada a menor risco de sangramentos em 1 ano (19,4% *vs.* 44,4%, HR 0,36, IC 95% 0,26-0,50, p < 0,0001) sem que isso estivesse associado a maior risco de eventos isquêmicos.

O primeiro estudo randomizado a avaliar o uso de DOAC no contexto de terapia dupla e tripla no paciente com FA submetido à angioplastia foi o PIONEER AF-PCI.^268^ Neste, foram comparadas três estratégias antitrombóticas: rivaroxabana 15mg/dia associada a um inibidor P2Y_12_; terapia tripla com DAP e rivaroxabana em baixas doses (2,5mg 12/12h); terapia tripla com DAP e antagonista da vitamina K. As estratégias com rivaroxabana mostraram causar menor risco de sangramento do que a terapia tripla com antagonistas da vitamina K. Não houve diferença de desfechos isquêmicos, mas o tamanho amostral de 2.124 pacientes não gerava poder suficiente para este desfecho. Uma crítica a este estudo foi o fato de as doses reduzidas de rivaroxabana utilizadas não serem as aprovadas para tratamento antitrombótico na FA.

No ano seguinte, foi publicado o estudo RE-DUAL PCI, que randomizou 2.725 pacientes com FA submetidos à angioplastia.^269^ Terapia dupla com dabigatrana em diferentes doses (150mg 2 vezes/dia e 110mg 2vezes/dia) associada a inibidor da P2Y_12_ foi comparada a terapia tripla com varfarina. No grupo da terapia tripla, o AAS era mantido por 1 mês nos casos que haviam recebido *stent* não farmacológico, e por 3 meses no grupo tratado com *stent* farmacológico. Os dois esquemas com dabigatrana causaram menor risco de sangramentos do que o esquema com varfarina na terapia tripla. Adicionalmente, demonstrou-se que não existe interação entre inibidores de bomba de próton e o efeito do medicamento, na medida em que o uso dessa classe de medicamentos não influenciou os resultados principais do estudo.^469^ O desenho do RE-DUAL PCI e do PIONEER AF-PCI não permitia avaliar se a redução do risco de sangramento observado com a terapia dupla era secundária à substituição de antagonistas da vitamina K por DOAC ou ao fato de se excluir o AAS do tratamento.

Para responder a esse questionamento, no ano de 2019, foi publicado o estudo AUGUSTUS,^270^ que randomizou 4.614 pacientes com FA que haviam sido submetidos à ICP ou apresentado SCA nos últimos 14 dias. O estudo usou um desenho fatorial 2x2 de forma que os pacientes foram randomizados em grupos que compararam apixabana 5mg 2 vezes por dia à varfarina, e grupos que compararam AAS a placebo. Todos os pacientes recebiam um inibidor P2Y_12_, sendo utilizado o clopidogrel em mais de 90% dos casos. Foram estudados, portanto, quatro grupos: (1) inibidor P2Y_12_ + varfarina + AAS; (2) inibidor P2Y_12_ + varfarina + placebo; (3) inibidor P2Y_12_ + apixabana + AAS; (4) inibidor P2Y_12_ + apixabana + placebo. Na comparação entre apixabana e varfarina, notou-se menor risco de sangramento com o uso do DOAC. A cada 24 pacientes tratados com apixabana, evitou-se um sangramento clinicamente relevante durante o período do estudo. Já na comparação de AAS *versus* placebo, foi demonstrado que o uso de AAS aumentou o risco de sangramento. A cada 14 pacientes tratados com terapia tripla incluindo AAS, houve um sangramento relevante a mais no período de 6 meses. Não houve aumento de desfechos isquêmicos com o uso de terapia dupla em relação à terapia tripla neste estudo, devendo-se considerar que a amostra não foi calculada objetivando este desfecho. Importante salientar que, no AUGUSTUS, os pacientes foram randomizados em média 6 dias após a ICP ou a SCA. Dessa forma, os pacientes receberam ao menos um breve curso de AAS e terapia tripla, antes da randomização. Outro dado relevante sobre este estudo é que ele também incluiu pacientes com FA e SCA tratada clinicamente (cerca de um quarto dos pacientes incluídos).

O estudo ENTRUST randomizou 1.506 pacientes portadores de FA e submetidos à angioplastia coronariana, com seguimento de 12 meses.^271^ O delineamento comparou a terapia dupla com edoxabana 60mg/dia associada a clopidogrel 75mg/dia à terapia tripla tradicional (varfarina e dupla antiagregação plaquetária com AAS e clopidogrel). A terapia dupla com edoxabana foi não inferior em relação ao desfecho primário sangramento. Este foi, portanto, o único ensaio clínico que não demonstrou, no que se refere ao desfecho sangramento, superioridade da terapia dupla com DOAC sobre a terapia tripla com varfarina.

**Figura 3.3:**
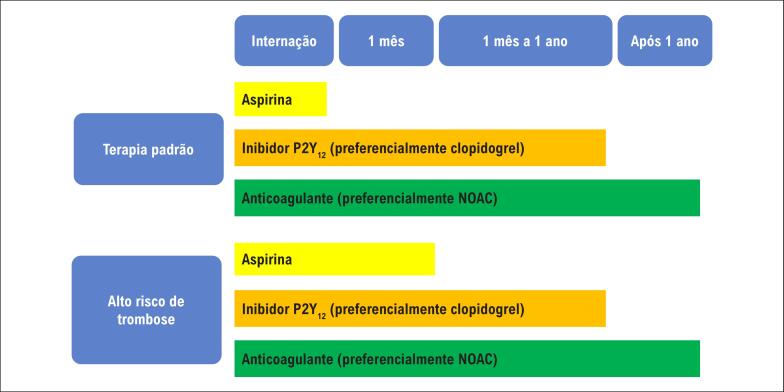
Esquema antitrombótico proposto para pacientes com síndrome coronariana aguda sem supra de ST e que apresentam FA com indicação de anticoagulação.

Uma possível preocupação em se utilizar terapia dupla no paciente com FA submetido à angioplastia seria o aumento do risco isquêmico, principalmente de trombose de *stent*. Os cinco *trials* citados previamente (WOEST, PIONEER AF-PCI, RE-DUAL PCI, AUGUSTUS e ENTRUST) foram desenhados para mostrar redução de sangramento com o uso de terapia antitrombótica menos agressiva. Nenhum deles, contudo, tinha poder suficiente para se avaliar de forma adequada aumento de risco isquêmico. Para avaliar esse aspecto, duas metanálises foram desenvolvidas, ambas demonstrando que, em comparação com varfarina + DAP, o uso de dupla terapêutica antitrombótica diminui eventos hemorrágicos sem aumentar eventos.^272^^,^^470^^,^^471^ Entretanto, ao comparar dupla ou tripla terapêutica antitrombótica, Gargiulo et al.^472^ demonstraram diminuição significativa de sangramento à custa de aumento significativo na incidência de trombose de *stent* (RR = 1,59, IC 95% 1,01 a 2,50) e uma tendência a aumento de IAM, com o uso da dupla terapêutica antitrombótica, sugerindo que alguns pacientes de muito alto risco de eventos isquêmicos, principalmente se com baixo risco de eventos hemorrágicos, podem se beneficiar da terapêutica antitrombótica tripla por até 30 dias apos o evento-índice (Figura 3.3).

**Table:** 

Escolha de terapia antitrombótica em pacientes após SCA e que apresentam FA com indicação de anticoagulação - Sumário de recomendações e evidências		
A utilização de DOAC deve ser preferida em relação à varfarina	I	A
O clopidogrel deve ser o inibidor P2Y_12_ preferido, uma vez que é o que foi mais estudado nesse contexto	I	A
O uso de terapia tripla deve ser mantido o menor tempo possível devido ao alto risco de sangramento associado.	IIa	A
Quando optado pelo uso da varfarina, deve-se almejar um INR entre 2 e 2,5.	IIa	C
O AAS deve ser usado em doses baixas, preferencialmente ≤ 100mg/dia.	IIa	C
Durante a internação hospitalar, deve-se utilizar terapia tripla com AAS + inibidor P2Y_12_ + anticoagulante.	IIa	C
Após a alta hospitalar, a rotina é manter inibidor P2Y_12_ e anticoagulante até 12 meses do evento. Após 12 meses do evento, retira-se o inibidor P2Y_12_, mantendo-se apenas o anticoagulante.	IIa	A
Em pacientes com risco isquêmico aumentado, pode-se manter a terapia tripla por um período maior.	IIa	B
O uso de inibidores de bombas de prótons como profilaxia de úlcera de estresse nesse grupo de pacientes deve ser considerado como primeira escolha devido ao risco elevado de sangramento gastrintestinal.	IIa	C

#### 3.2. Inibidores do Sistema Renina-Angiotensina-Aldosterona

Em pacientes pós-SCA, recomenda-se o uso de inibidores de ECA para aqueles com disfunção sistólica de VE/insuficiência cardíaca, hipertensão arterial sistêmica ou diabetes. Bloqueadores dos receptores de angiotensina (BRA) podem ser usados em pacientes intolerantes aos inibidores de ECA.

Antagonistas de mineralocorticoides estão indicados nos pacientes pós-SCA que apresentam FEVE ≤ 40% associada a evidências de insuficiência cardíaca ou a diabetes.^283^

No manejo da hipertensão arterial dos pacientes, após internação por SCA e a longo prazo, há de se ter um cuidado especial ao adequado controle da pressão sanguínea, conforme as recomendação da Diretriz Brasileira de Hipertensão Arterial.^473^ No entanto, é importante considerar a relevância da curva J, não obstante, toda a polêmica de sua existência e papel. Os dados do estudo ONTARGET^474^^,^^475^ demonstraram que reduções excessivas da pressão arterial sistólica (PAS) foram associadas ao aumento de eventos isquêmicos, sendo observado que o valor de PAS de 126mmHg teve a menor ocorrência de IAM. Análise do estudo TNT^476^ avaliou o impacto dos níveis de pressão arterial em mais de 10.000 pacientes coronariopatas. Foi observada associação com mais eventos isquêmicos quando a PAS atingiu níveis inferiores a 110-120mmHg e a pressão arterial diastólica (PAD), abaixo de 60-70mmHg. Entretanto, o estudo SPRINT, que randomizou 9.361 pacientes não diabéticos e com alto risco cardiovascular para uma meta de controle intensivo (PAS < 120mmHg) *versus* uma meta convencional (PAS < 140mmHg), mostrou que o tratamento intensivo reduziu o desfecho composto de morte cardiovascular, infarto, AVC, SCA ou insuficiência cardíaca (HR 0,75; IC 95% 0,64 a 0,89; p < 0,001), além de ter reduzido mortalidade por todas as causas (HR 0,73; IC 95% 0,60 a 0,90; p = 0,003). Houve aumento na incidência de eventos adversos de quedas, síncope, hipotensão e insuficiência renal aguda no grupo intensivo. Neste estudo, não houve aumento na incidência de infarto nem de SCA com o tratamento intensivo. Entretanto, o estudo incluiu menos de 20% de pacientes com doença cardiovascular manifesta.^477^ Não existem estudos semelhantes especificamente em pacientes com histórico de SCA. Por conta disso, em virtude do potencial de piora da isquemia, recomenda-se cautela em reduzir a PA, devendo-se evitar uma PAD inferior a 60mmHg, a fim de evitar redução da pressão de perfusão coronária.

**Table:** 

Uso de inibidores do sistema renina-angiotensina-aldosterona nos pacientes pós-síndrome coronariana aguda sem supradesnível do segmento ST - Sumário de recomendações e evidências		
O uso de inibidores da ECA está recomendado por tempo indeterminado em pacientes com disfunção sistólica do VE, insuficiência cardíaca, hipertensão arterial sistêmica ou diabetes. Os bloqueadores do receptor de angiotensina devem ser utilizados em pacientes intolerantes aos inibidores da ECA.	I	A
Espironolactona está recomendada em pacientes com FEVE ≤ 40% associada à insuficiência cardíaca ou a diabetes.	I	A

#### 3.3. Betabloqueadores

O uso de betabloqueadores a longo prazo é recomendado em pacientes com insuficiência cardíaca e fração de ejeção ≤ 40%. Já em pacientes com função sistólica de ventrículo esquerdo preservada, os dados são menos robustos. As evidências favoráveis ao uso dessas medicações surgiram no início dos anos 1980, quando o tratamento das SCA era bastante distinto do atual.^478^^,^^479^ O uso de betabloqueadores em pacientes com SCASSST e sem insuficiência cardíaca não foi avaliado por ensaios clínicos randomizados. Os dados disponíveis nessa população vêm de estudos observacionais que sugerem não ser útil o uso de betabloqueador a longo prazo pós-IAM.^480^^,^^481^ Experiência nacional especificamente analisando pacientes com SCASSST seguidos por aproximadamente 17 anos sugere benefício no uso do betabloqueador a longo prazo na população com disfunção ventricular esquerda, mas não naqueles com fração de ejeção normal.^137^ Atualmente, o estudo DANBLOCK (NCT 03778554) está randomizando pacientes com infarto do miocárdio recente e FE > 40% para avaliar se o uso de betabloqueadores reduz desfechos isquêmicos no contexto da terapia atual de SCA. Os resultados devem estar disponíveis em 2023.

**Table:** 

Uso de betabloqueadores em pacientes pós-síndrome coronariana aguda sem supradesnível do segmento ST - Sumário de recomendações e evidências		
O uso de betabloqueadores é recomendando por tempo indeterminado em pacientes com disfunção ventricular esquerda. Deve-se usar os agentes com comprovada eficácia neste cenário.	I	B

#### 3.4. Antidiabéticos

Mais de 30% dos pacientes admitidos com SCASSST apresentam diabetes.^482^ O tratamento desta comorbidade sofreu grandes mudanças nos últimos anos com o advento de medicamentos que não apenas diminuem os níveis glicêmicos, mas também reduzem a incidência de desfechos cardiovasculares. Há evidências de que o controle mais intensivo da hemoglobina glicada (HbA1C) em pacientes diabéticos reduz o risco de complicações microvasculares.^483^^,^^484^ O impacto sobre eventos macrovasculares foi mais heterogêneo, parecendo haver benefício no seguimento de longo prazo (> 10 anos), desde que seja realizado precocemente após o diagnóstico.^485^ Com base nos achados em relação a eventos microvasculares, recomenda-se um alvo de HbA1C inferior a 7% na maioria dos pacientes diabéticos. É importante notar, contudo, que este número serve como um princípio geral, mas deve ser individualizado para cada paciente. No estudo ACCORD, por exemplo, a adoção de um controle glicêmico mais agressivo, além de não trazer redução de desfechos cardiovasculares maiores, foi associada a uma maior mortalidade total, levando à interrupção precoce do estudo.^486^ Os pacientes incluídos no estudo apresentavam diabetes de longa duração (10 anos, em média), além da prevalência de doença cardiovascular elevada (cerca de 35%). Por isso, em pacientes com baixa expectativa de vida, diabetes de longa duração, histórico de hipoglicemias graves, doença microvascular ou macrovascular avançada, alvos menos rigorosos de HbA1C (p. ex., < 8%) podem ser considerados. De modo inverso, em pacientes com diabetes de início recente, sem comorbidades relevantes e com bom entendimento da doença, alvos mais intensivos (p. ex., HbA1C < 7%) podem ser almejados.

Uma vez determinados os alvos glicêmicos a serem atingidos, é necessário escolher os medicamentos a serem usados. Todo paciente com diagnóstico de diabetes deve receber metformina a menos que esta esteja formalmente contraindicada.^487^ Trata-se de uma medicação de baixo custo, amplamente disponível na rede pública, com efeitos favoráveis na redução de peso e controle glicêmico, além de potencialmente reduzir a incidência de eventos cardiovasculares.^484^ Além disso, os estudos randomizados que surgiram nos últimos anos mostrando redução de desfechos cardiovasculares com os novos antidiabéticos utilizaram como terapia de base a metformina em mais de 70% a 80% dos pacientes.

Os principais efeitos colaterais da metformina são relacionados ao trato gastrintestinal (sensação de empachamento, dor abdominal, diarreia) e podem ser evitados com a progressão mais lenta da dose. Pacientes com *clearance* de creatinina abaixo de 30mL/min têm contraindicação formal ao fármaco.^488^ Pacientes que fazem uso crônico de metformina apresentam maior incidência de baixos níveis de vitamina B_12_, devendo-se considerar o monitoramento desta nesta população, principalmente se houver anemia ou neuropatia associadas.^489^

Em pacientes com histórico de coronariopatia, recomenda-se considerar terapia com inibidores da SGLT2 ou agonistas do receptor GLP1, independentemente dos níveis de HbA1C. Recomenda-se que se opte por alguma das medicações que já mostraram ter benefício na redução de eventos cardiovasculares em estudos de fase 3.

A decisão da escolha entre inibidores da SGLT2 e agonistas dos receptores GLP1 depende de vários fatores, entre os quais expomos alguns na Tabela 3.4. Ambas as classes de medicações mostraram ter benefícios similares em relação à redução de desfechos cardiovasculares maiores em pacientes de prevenção secundária.^490^ O ideal é que seja feita uma decisão compartilhada com o paciente pesando aspectos práticos como via de administração (VO *vs.* SC) e preço, bem como os benefícios clínicos demonstrados pelos estudos randomizados. Vale ressaltar que, em virtude do benefício além do controle glicêmico, estes medicamentos devem ser prescritos o quanto antes após a SCA, uma vez que pacientes diabéticos com SCA constituem um grupo de muito alto risco, evitando-se a inércia terapêutica. Em uma subanálise do estudo DECLARE de pacientes com IAM prévio, o uso da dapagliflozina em relação ao placebo reduziu o composto de morte CV, infarto e AVC isquêmico (principalmente à custa de redução de reinfarto), e este benefício pareceu ser maior quanto mais próximo da fase aguda do evento coronário.^491^

**Tabela 3.4:** Fatores a serem considerados para decidir qual o melhor novo antidiabético no paciente coronariopata

	iSGLT2	GLP1-RA
Modo de administração	VO Administração diária	SC (já existe semaglutida VO, mas ainda não está disponível no Brasil) Administração diária ou semanal
Vantagens	Reduz MACE Reduz morte cardiovascular (empagliflozina) Reduz IC Reduz (discretamente) PA Previne progressão de nefropatia	Reduz MACE Reduz morte cardiovascular (liraglutida) Perda de peso Previne progressão de nefropatia (liraglitida)
Precauções	ClCr < 30mL/min/1,73m^2^ Histórico de infecções genitais Histórico de cetoacidose Osteoporose/Fraturas (canagliflozina) Doença arterial periférica (canagliflozina)	ClCr < 30mL/min/1,73m^2^ Náuseas História de pancreatite Retinopatia (semaglutida)

É importante enfatizar que, no caso da dapagliflozina, o estudo DAPA-HF mostrou benefício cardiovascular em pacientes com insuficiência cardíaca e fração de ejeção do VE inferior a 40% independentemente da presença de diabetes.^492^ Nesse *trial*, 55% dos pacientes apresentavam coronariopatia como causa da IC e a redução observada de 23% no desfecho composto de piora de IC e morte cardiovascular ocorreu independentemente dos níveis de HbA1C do paciente. Dessa forma, em pacientes com disfunção de VE por coronariopatia e com diabetes associado, os inibidores da SGLT2 têm mais evidências de benefícios do que os agonistas de receptores GLP1.

Outro cenário em que os iSGLT2 mostraram benefício foi no de pacientes diabéticos com doença renal crônica. No estudo CREDENCE, o uso de canagliflozina na dose de 100mg por dia mostrou reduzir progressão de nefropatia em pacientes diabéticos que apresentavam ClCr entre 30 e 90mL/min associado a proteinúria (relação albumina sobre creatina urinária ≥ 300mg/g).^493^ O desfecho primário de doença renal terminal, elevação superior a 2 vezes nos níveis de creatinina ou morte de causas renais ou cardiovasculares foi reduzido em 30% com o uso do iSGLT2 (HR 0,70; IC 95% 0,59 a 0,82; p = 0,00001).

A maioria dos outros antidiabéticos mostrou-se neutra em relação a eventos cardiovasculares. Contudo, em alguns casos, houve piora de desfechos. No estudo SAVOR-TIMI 53, o uso do inibidor de DPP-4 saxagliptina foi testado contra o placebo em uma população de 16.492 pacientes diabéticos.^494^ Além de não ter havido redução de desfechos cardiovasculares com a medicação, observou-se aumento na taxa de internações por insuficiência cardíaca (de 2,8% para 3,5%, HR 1,27; IC 95% 1,07 a 1,51; p = 0,007). Esta medicação deve, portanto, ser evitada em pacientes com IC.

**Table:** 

Manejo ambulatorial de pacientes diabéticos com passado de síndrome coronariana aguda - Sumário de recomendações e evidências		
Alvo de hemoglobina glicada < 7% deve ser usado na maioria dos pacientes diabéticos visando à redução de eventos microvasculares.	I	A
O alvo de hemoglobina glicada deve ser individualizado, levando em conta características como presença de comorbidades, expectativa de vida e duração do DM	I	C
Metformina é o fármaco de escolha para o tratamento inicial de diabetes, salvo contraindicações.	I	A
Em pacientes após SCA, deve-se considerar a associação de um iSGLT2 ou de um GLP1-RA que comprovadamente têm benefícios cardiovasculares independentemente dos níveis de hemoglobina glicada.	I	A
Em pacientes com insuficiência cardíaca ou com nefropatia (ClCr entre 30 e 90mL/min e/ou com proteinúria), os iSGLT2 são a opção de escolha.	I	A
Uso de saxagliptina e pioglitazona em pacientes diabéticos com insuficiência cardíaca.	III	A

#### 3.5. Hipolipemiantes

O tratamento com estatinas deve ser iniciado precocemente, preferencialmente com agentes de alta potência (*i. e.*, rosuvastatina 20 a 40mg ou atorvastatina 40 a 80mg). Nos pacientes que já usavam esta medicação, o tratamento não deve ser interrompido. Caso a estatina usada previamente seja de baixa ou moderada potência, a modificação para estatinas de alta potência deve ser considerada.

No seguimento ambulatorial, deve-se atentar às metas terapêuticas de LDL-c e não HDL-c, respectivamente, < 50mg/dL e < 80mg/dL. Caso as metas não sejam atingidas e o paciente ainda não esteja em uso de estatinas de alta potência, recomenda-se que se prescreva uma medicação deste grupo. Se, mesmo assim, os alvos de LDL não forem atingidos, recomenda-se a associação com ezetimiba 10mg/dia. Se após essas medidas o LDL continuar acima da meta, deve-se considerar o uso dos inibidores da PCSK9.

O primeiro estudo avaliando desfechos clínicos associados a inibidores da PCSK9 foi o FOURIER,^495^ publicado em 2017. Foram avaliados 27.564 pacientes com histórico de doença cardiovascular que mantinham LDL ≥70mg/dL apesar do uso de estatinas. Os indivíduos eram randomizados para receberem evolocumab (140mg SC a cada 2 semanas ou 420mg SC 1 vez por mês, de acordo com a preferência do paciente) ou placebo. Observou-se no grupo evolucumab uma redução significativa do desfecho composto de morte cardiovascular, IAM, AVC, necessidade de revascularização coronária ou internação devido à AI. Este desfecho ocorreu em 11,3% dos pacientes do grupo controle e em 9,8% do grupo evolocumab ao longo de um seguimento médio de 26 meses. Não houve diferença de mortalidade.

Em 2018, foi publicado o estudo ODISSEY-OUTCOMES que avaliou o uso do alirocumab em pacientes com passado de SCA no ano anterior à inclusão.^496^ Os pacientes apresentavam LDL ≥70mg/dL apesar de estarem usando estatinas de alta potência e eram randomizados para receber alirocumab 75mg SC a cada 2 semanas ou placebo. Em um seguimento médio de 2,8 anos, foi observada uma redução de 14,4% no desfecho composto de morte por coronariopatia, IAM, AVC e hospitalização por AI. Neste *trial* houve uma aparente redução de mortalidade global (de 4,1% para 3,5%), embora não tenha havido redução de morte CV. Os benefícios da nova medicação foram mais proeminentes em pacientes com LDL basal acima de 100mg/dL. Enquanto no grupo geral a redução de risco absoluto de mortalidade foi de 0,6%, no grupo com LDL acima de 100mg/dL, foi de 1,7%.

Um dado relevante é que, no ODISSEY-OUTCOMES, o inibidor de PCSK9 foi iniciado em média 2,6 meses após o episódio de SCA. Já nos pacientes com histórico de infarto do estudo FOURIER (cerca de 80% da população total), o evolocumab foi iniciado em média 3,4 anos após o último IAM. Ou seja, com base nas evidências desses estudos, o uso de inibidores de PCSK9, via de regra, deve ser aventado no acompanhamento ambulatorial de pacientes infartados e não na fase aguda intra-hospitalar.

Outro hipolipemiante que mostrou redução de eventos cardiovasculares no contexto de prevenção secundária foi a suplementação de ômega 3 à base de EPA (icosapenta-etil 4g/dia). Tal estratégia foi avaliada no estudo REDUCE-IT, que randomizou pacientes de alto risco (prevenção secundária ou diabetes com outro fator de risco associado), os quais apresentavam hipertrigliceridemia apesar do uso de estatinas.^497^ A maioria dos pacientes (70,7%) era de prevenção secundária. Foi observada uma redução no desfecho primário composto de morte cardiovascular, IAM, AVC, necessidade de revascularização ou AI de 22% para 17,2%. Houve redução de morte cardiovascular de 5,2% para 4,3% (IC 0,66 a 0,98; p = 0,03). A cada 21 pacientes tratados durante o seguimento médio de 4,9 anos do estudo, um evento cardiovascular foi prevenido. Uma crítica feita ao REDUCE-IT foi a de que o placebo utilizado (óleo mineral) aumentou o LDL 5mg/dL a mais do que o grupo que usou o EPA. Isso poderia ter aumentado o risco de eventos no grupo placebo.

Deve ser enfatizado que, no estudo REDUCE-IT, foi utilizado um tipo de formulação específica de ômega 3 que contava apenas com EPA (icosapenta-etil), o qual não teve impacto relevante sobre o LDL no *trial* (aumento de 2mg/dL). Há evidências de que formulações de ômega 3 contendo ácido docosa-hexaenoico (DHA) elevam níveis de LDL.^498^ Assim, a diretriz recomenda que, em caso de uso de ômega 3 no paciente pós-SCA, seja utilizada a formulação usada no REDUCE-IT. Esta não está disponível no Brasil até a data da publicação desta Diretriz.

**Table:** 

Uso de hipolipemiantes após a alta de pacientes com síndrome coronariana aguda sem supra de ST - Sumário de recomendações e evidências		
Em indivíduos com diagnóstico prévio de SCA, o LDL-c deve ser reduzido para < 50mg/dL e o não HDL-c para < 80mg/dL.	I	B
Sempre que possível e tolerado, deve-se dar preferência para o uso de estatina de alta intensidade.	I	A
Ezetimiba deve ser associada quando a meta do LDL-c não for atingida com o tratamento com estatinas na dose máxima tolerada.	I	A
Inibidores da PCSK9 podem ser considerados em pacientes em tratamento otimizado com estatinas na maior dose tolerada, associado ou não à ezetimiba, e que não tenham alcançado as metas de LDL-c ou não HDL-c recomendadas.	IIa	A

#### 3.6. Outros Medicamentos

Pacientes pós-SCA apresentam risco aumentado de sangramento devido ao uso de terapia antitrombótica. O trato gastrintestinal é o sítio mais frequente de sangramentos clinicamente relevantes em pacientes usando terapia antiplaquetária crônica.^499^ Uma forma de reduzir o risco destes eventos gastrintestinais é o uso de protetores gástricos. Inibidores de bombas de prótons se mostraram mais eficazes que antagonistas de receptores H2 em estudos observacionais.^500^ Contudo, alguns estudos sugeriram que o uso de inibidores de bombas de prótons em conjunto com o clopidogrel poderia reduzir a atividade antiplaquetária deste (*in vitro*).^225^^,^^226^ O estudo randomizado COGENT (ver Parte 2) mostrou que a associação de omeprazol ao clopidogrel reduziu o risco de complicações gastrintestinais sem aumento aparente de eventos isquêmicos (embora a interrupção precoce do estudo não consiga excluir um aumento de eventos isquêmicos com o omeprazol).^231^

Recomenda-se, portanto, o uso de inibidores de bomba de prótons em pacientes que usam DAP e que tenham risco aumentado de sangramento (história de sangramento gastrintestinal, história de doença ulcerosa péptica, uso de anticoagulantes, uso crônico de AINE, uso crônico de corticosteroides ou que apresentem dois ou mais dos seguintes fatores: idade ≥ 65 anos, dispepsia, doença do refluxo gastresofágico, infecção por *Helicobacter pylori*, uso crônico de álcool).

Os pacientes devem ser orientados após a alta para que, caso apresentem dor anginosa em repouso, utilizem nitrato sublingual (dinitrato, mononitrato ou nitroglicerina), desde que não existam contraindicações. Importante orientar que, caso a dor não melhore em 3 a 5min, o paciente ou seu acompanhante deve acionar o serviço de emergência.

Infecções virais e bacterianas podem servir de gatilho para desencadear um evento cardiovascular agudo em pacientes cardiopatas. Há evidências de que a vacinação para influenza e para pneumococo podem reduzir internações e até mesmo mortalidade nestes pacientes.^501^ Desta forma, recomenda-se a vacinação anual para influenza em pacientes portadores de coronariopatia. O ideal é que a vacina seja administrada na época da campanha vacinal (abril e maio). Em relação à vacina pneumocócica, recomenda-se a aplicação de uma dose da “Pneumo 23” com reforço após 5 anos.^502^ Um estudo randomizado brasileiro em andamento está testando se a vacina anti-influenza durante a hospitalização por SCA em dose dobrada é superior à vacinação em dose convencional 30 dias após o evento, a fim de reduzir desfechos cardiovasculares (NCT 04001504).

**Figura 3.4:**
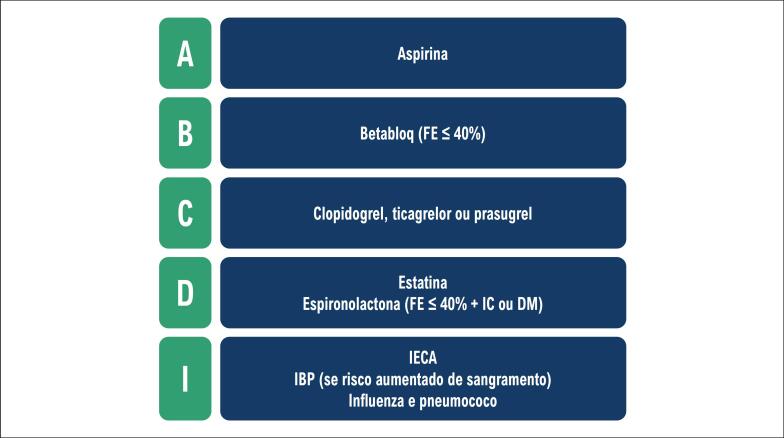
Resumo da prescrição de alta pós-síndrome coronariana aguda sem supradesnível de ST

A terapia direcionada a reduzir a inflamação na placa tem começado a trazer resultados promissores. No estudo CANTOS, em pacientes com infarto prévio (a partir de 30 dias após o evento-índice) e proteína C-reativa elevada, o canaquinumabe, um anticorpo monoclonal anti-interleucina 1-β, reduziu os eventos cardiovasculares maiores, ou seja, o composto de morte CV, reinfarto ou AVC (HR 0,88; IC 95% 0,79 a 0,97; p = 0,02).^503^ Entretanto, houve aumento de morte por infecção. O custo proibitivo fez que com este medicamento não tenha sido incorporado à prática clínica para esta indicação. Já a colchicina, um medicamento amplamente disponível e de baixo custo, na dose de 0,5mg uma vez ao dia, reduziu de maneira significativa o desfecho composto de morte CV, reinfarto, hospitalização por AI com necessidade de revascularização, AVC ou parada cardíaca reanimada (HR 0,77; IC 95% 0,61 a 0,96; p = 0,02) em pacientes com IAM recente (até 30 dias do evento-índice). Houve aumento na incidência de pneumonia.^504^ A Figura 3.4 traz um resumo dos medicamentos que são recomendados após alta por SCASSST.

**Table:** 

Outras intervenções farmacológicas em pacientes após a alta por uma síndrome coronariana aguda - Sumário de recomendações e evidências		
Orientar o paciente a usar nitrato sublingual caso apresente dor torácica em repouso, desde que não apresente contraindicações à medicação.	I	C
Vacinação contra influenza e pneumococo, a fim de reduzir morbimortalidade.	I	B
Uso de inibidores de bomba de prótons em pacientes em uso de DAP e que apresentem risco aumentado de sangramento (história de sangramento gastrintestinal, história de doença ulcerosa péptica, uso de anticoagulantes, uso crônico de AINE, uso crônico de corticosteroides ou que tenham dois ou mais dos seguintes fatores: idade ≥65 anos, dispepsia, doença do refluxo gastresofágico, infecção por *Helicobacter pylori,* uso crônico de álcool).	IIa	B

### 4. Rastreamento Clínico Pós-alta

Os pacientes de baixo risco e os revascularizados após SCASSST têm, por recomendação de uma reavaliação precoce, entre 2 e 6 semanas inicialmente. Naqueles pacientes com maior gravidade, recomenda-se a avaliação dentro de 14 dias.^505^ Obviamente que esses prazos devem levar em consideração as particularidades de cada situação, uma vez que as intercorrências dos procedimentos, condições de acesso ao serviço de saúde e características individuais de cada paciente são muito variáveis.

O risco de complicações existe mesmo em pacientes assintomáticos e, portanto, a avaliação deste risco se aplica a pacientes sintomáticos e assintomáticos.

Após revascularização e/ou após SCA estabilizada nos primeiros 12 meses, os pacientes devem ser monitorados com mais vigilância, porque correm maior risco por complicações e porque estão sujeitos a alterações no tratamento farmacológico.^506^ Portanto, são recomendadas pelo menos duas visitas no primeiro ano de acompanhamento, sendo a primeira, idealmente, dentro de 3 meses.

Em um paciente com disfunção sistólica do VE antes do procedimento de revascularização ou após a SCA, uma reavaliação da função do VE deve ser considerada 8 a 12 semanas após a intervenção.^507^^,^^508^

A função cardíaca pode ter melhorado devido a mecanismos como recuperação de atordoamento do miocárdio ou hibernação, que pode ser revertida por revascularização ou por outro lado piorado em virtude de outras comorbidades, como valvopatias, arritmias ou inflamações etc. Esses achados precisam ser identificados e tratados.^509^

De forma similar, provas não invasivas de isquemia podem ser realizadas após revascularização, para identificação de isquemia residual e também servindo de referência para comparativos subsequentes.^509^ Essa abordagem deve ser individualizada, uma vez que cada situação de revascularização traz consigo peculiaridades anatômicas e difere muito de paciente para paciente. Ressaltamos que as recomendações são, em sua maioria, baseadas em opiniões de especialistas.

Em pacientes que estejam assintomáticos há mais de 1 ano, uma reavaliação pelo menos anual é recomendada.^509^ Deve-se avaliar o estado clínico geral do paciente e adesão ao tratamento medicamentoso, bem como o perfil de risco estabelecidos pelos escores. Exames laboratoriais que incluem perfil lipídico, função renal, um hemograma completo e também biomarcadores devem ser realizados pelo menos a cada 2 anos.^507^^,^^508^

O perfil lipídico e o *status* glicêmico devem ser avaliados periodicamente e, apesar de não haver evidências que suportem a periodicidade dessa avaliação, de forma geral, recomenda-se uma avaliação anual.^509^

Alguns biomarcadores de inflamação parecem estar associados como marcadores de eventos. A proteína C-reativa de alta sensibilidade parece ser o de maior evidência em múltiplos estudos, mesmo em pacientes em prevenção primária. NT-proBNP, fator de Von Willenbrand e interleucina- 6, também parecem ser preditores de eventos.^510^ Escores estabelecidos com associação de biomarcadores (LDL, NT-proBNP, troponina T, produtos de degradação de fibrina), mostraram-se com melhor poder de reclassificação e aumento da estatística-C do que os escores com base em modelos clínicos, e podem ser promissoras ferramentas de predição de eventos em pacientes coronariopatas.^511^^,^^512^ No entanto, a falta de evidências mais robustas deve limitar a utilização desses biomarcadores a casos selecionados, e não é aplicada de rotina.

Um ECG deve ser solicitado em cada visita para determinar o ritmo e a frequência cardíaca. O ecocardiograma pode ser benéfico para avaliar a função do VE (diastólica e sistólica), *status* valvar e dimensões cardíacas em pacientes aparentemente assintomáticos a cada 3 a 5 anos. Da mesma forma, pode ser benéfico avaliar não invasivamente a presença de isquemia silenciosa em um paciente aparentemente assintomático a cada 3 a 5 anos, preferencialmente com exames de estresse com imagens.^513^

A angioTC de coronárias não deve ser utilizada para avaliação em virtude da falta de informações funcionais. Em casos específicos, ela pode ajudar na avaliação da patência de coronárias e enxertos.^512^

Para pacientes com histórico de SCASSST e/ou já revascularizados, com sintomas inequívocos de angina, de acordo com a grande maioria das evidências, a estratificação invasiva é a melhor opção. Para pacientes que tenham sintomas duvidosos, a utilização de exames de estresse com imagem é recomendada.^513^

**Table:** 

Rastreamento clínico pós-alta - Sumário de recomendações e evidências		
Pacientes assintomáticos		
Uma visita periódica a um cardiologista (preferencialmente em até 3 meses), para reavaliar qualquer alteração potencial no risco da situação dos pacientes, com avaliação clínica das medidas de modificação do estilo de vida, adesão aos alvos fatores de risco e o desenvolvimento de comorbidades que podem afetar tratamentos e resultados.	I	C
Em pacientes com sintomas leves ou inexistentes recebendo tratamento médico, nos quais a estratificação de risco não invasiva indica um alto risco, e para quem a revascularização é considerada para melhorar o prognóstico, a angiografia coronária invasiva (FFR, quando necessário e disponível) é recomendada.	I	C
Pacientes de alto risco, submetidos à revascularização há cerca de 6 meses, podem realizar estratificação de risco por exame de imagem sob estresse.	IIb	C
Em pacientes submetidos à revascularização de alto risco (p. ex., tronco de coronária esquerdo desprotegido), pode- se considerar nova angiografia, independentemente dos sintomas.	IIb	C
Exame de imagem com estresse de rotina em pacientes revascularizados de forma percutânea há mais de 1 ano e cirurgicamente há mais de 5 anos.	IIb	C
Uso de angioTC de coronária como rotina para estratificação isoladamente.	III	C
Uso de angiografia coronariana apenas para estratificação de risco em paciente assintomático.	III	C
Pacientes assintomáticos		
Avaliação do *status* da doença coronária em pacientes com deterioração da função sistólica do VE sem causa identificável.	I	C
Realização de exame de imagem com estresse (preferencialmente) em pacientes com sintomas novos e / ou piora dos sintomas prévios.	I	C
Realização de angiografia (com FFR ou iFR, se necessário) em pacientes com sintomas inequívocos de coronariopatia, principalmente se refratários ao tratamento medicamentoso, ou que se enquadrarem no perfil de alto risco.	I	C
Angiografia em pacientes com achados de alto risco em exames de imagem com estresse.	I	C
Em paciente revascularizado previamente, deve fazer exame de imagem sob estresse em detrimento de teste ergométrico isolado 12.	IIa	C
