# Supplementary material for: Diretrizes da Sociedade Brasileira de Cardiologia sobre Angina Instável e Infarto Agudo do Miocárdio sem Supradesnível do Segmento ST – 2021
Source: Arq Bras Cardiol. 2021 Jul 15;117(1):181–264. [Article in Portuguese] doi: 10.36660/abc.20210180 (PMC8294740; doi:10.36660/abc.20210180)
Supplement: Translation

# Translation: Brazilian Society of Cardiology Guidelines on Unstable Angina and Acute Myocardial Infarction without ST-Segment Elevation – 2021

**Authors:** José Carlos Nicolau, Gilson Soares Feitosa, João Luiz Petriz, Remo Holanda de Mendonça Furtado, Dalton Bertolim Précoma, Walmor Lemke, Renato Delascio Lopes, Ari Timerman, José A. Marin, Luiz Bezerra, Bruno Ferraz de Oliveira Gomes, Eduardo Cavalcanti Lapa Santos, Leopoldo Soares Piegas, Alexandre de Matos Soeiro, Alexandre Jorge de Andrade Negri, Andre Franci, Brivaldo Markman, Bruno Mendonça Baccaro, Carlos Eduardo Lucena Montenegro, Carlos Eduardo Rochitte, Carlos José Dornas Gonçalves Barbosa, Cláudio Marcelo Bittencourt das Virgens, Edson Stefanini, Euler Roberto Fernandes Manenti, Felipe Gallego Lima, Francisco das Chagas Monteiro, Harry Correa, Henrique Patrus Mundim Pena, Ibraim Masciarelli Francisco Pinto, João Luiz de Alencar Araripe Falcão, Joberto Pinheiro Sena, José Maria Peixoto, Juliana Ascenção de Souza, Leonardo Sara da Silva, Lilia Nigro Maia, Louis Nakayama Ohe, Luciano Moreira Baracioli, Luís Alberto de Oliveira Dallan, Luis Augusto Palma Dallan, Luiz Alberto Piva e Mattos, Luiz Carlos Bodanese, Luiz Eduardo Fonteles Ritt, Manoel Fernandes Canesin, Marcelo Bueno da Silva Rivas, Marcelo Franken, Marcos José Gomes Magalhães, Múcio Tavares de Oliveira, Nivaldo Menezes Filgueiras, Oscar Pereira Dutra, Otávio Rizzi Coelho, Paulo Ernesto Leães, Paulo Roberto Ferreira Rossi, Paulo Rogério Soares, Pedro Alves Lemos, Pedro Silvio Farsky, Rafael Rebêlo C. Cavalcanti, Renato Jorge Alves, Renato Abdala Karam Kalil, Roberto Esporcatte, Roberto Luiz Marino, Roberto Rocha Corrêa Veiga Giraldez, Romeu Sérgio Meneghelo, Ronaldo de Souza Leão Lima, Rui Fernando Ramos, Sandra Nivea dos Reis Saraiva Falcão, Talia Falcão Dalçóquio, Viviana de Mello Guzzo Lemke, William Azem Chalela, Wilson Mathias

**Affiliations:** 1 São PauloSP Brazil Instituto do Coração (InCor), Hospital das Clínicas da Faculdade de Medicina da Universidade de São Paulo (HCFMUSP), São Paulo, SP – Brazil; 2 SalvadorBA Brazil Escola Bahiana de Medicina e Saúde Pública, Salvador, BA – Brazil; 3 SalvadorBA Brazil Centro Universitário de Tecnologia e Ciência (UniFTC), Salvador, BA – Brazil; 4 Rio de JaneiroRJ Brazil Hospital Barra D'Or, Rede D'Or São Luiz, Rio de Janeiro, RJ – Brazil; 5 São PauloSP Brazil Hospital Israelita Albert Einstein, São Paulo, SP – Brazil; 6 Campina Grande do SulPR Brazil Sociedade Hospitalar Angelina Caron, Campina Grande do Sul, PR – Brazil; 7 CuritibaPR Brazil Clínica Cardiocare, Curitiba, PR – Brazil; 8 CuritibaPR Brazil Hospital das Nações, Curitiba, PR – Brazil; 9 Durham USA Duke University, Durham – USA; 10 São PauloSP Brazil Instituto Dante Pazzanese de Cardiologia, São Paulo, SP – Brazil; 11 Ribeirão PretoSP Brazil Hospital das Clínicas da Faculdade de Medicina da Universidade de São Paulo, Ribeirão Preto, SP – Brazil; 12 TeresinaPI Brazil Centro Universitário UNINOVAFAPI, Teresina, PI – Brazil; 13 Rio de JaneiroRJ Brazil Universidade Federal do Rio de Janeiro (UFRJ), Rio de Janeiro, RJ – Brazil; 14 RecifePE Brazil Hospital das Clínicas da Universidade Federal de Pernambuco (UFPE), Recife, PE – Brazil; 15 São PauloSP Brazil Hospital do Coração (HCor), São Paulo, SP – Brazil; 16 São PauloSP Brazil Faculdade de Medicina da Universidade de São Paulo (HCFMUSP), São Paulo, SP – Brazil; 17 João PessoaPB Brazil Hospital Universitário Lauro Wanderley, João Pessoa, PB – Brazil; 18 São PauloSP Brazil Hospital Sírio-Libanês, São Paulo, SP – Brazil; 19 RecifePE Brazil Pronto Socorro Cardiológico de Pernambuco (PROCAPE), Recife, PE – Brazil; 20 BrasíliaDF Brazil Hospital do Coração do Brasil, Rede D'Or São Luiz, Brasília, DF – Brazil; 21 SalvadorBA Brazil Universidade Federal da Bahia (UFBA), Salvador, BA – Brazil; 22 São PauloSP Brazil Escola Paulista de Medicina da Universidade Federal de São Paulo (UNIFESP), São Paulo, SP – Brazil; 23 Porto AlegreRS Brazil Instituto de Medicina Vascular Mãe de Deus, Porto Alegre, RS – Brazil; 24 São LuísMA Brazil Universidade Federal do Maranhão (UFMA), São Luís, MA – Brazil; 25 FlorianópolisSC Brazil Clínica Unicardio, Florianópolis, SC – Brazil; 26 Belo HorizonteMG Brazil Faculdade de Ciências Médicas de Minas Gerais (FELUMA), Belo Horizonte, MG – Brazil; 27 São PauloSP Brazil Grupo Fleury, São Paulo, SP – Brazil; 28 FortalezaCE Brazil Universidade Federal do Ceará (UFC), Fortaleza, CE – Brazil; 29 SalvadorBA Brazil Hospital Santa Izabel da Santa Casa da Bahia, Salvador, BA – Brazil; 30 Belo HorizonteMG Brazil Universidade José do Rosário Vellano (UNIFENAS), Belo Horizonte, MG – Brazil; 31 GoiâniaGO Brazil Centro de Diagnóstico por Imagem (CDI), Goiânia, GO – Brazil; 32 São José do Rio PretoSP Brazil Faculdade de Medicina de São José do Rio Preto (FAMERP), São José do Rio Preto, SP – Brazil; 33 RecifePE Brazil Rede D'Or São Luiz, Recife, PE – Brazil; 34 Porto AlegreRS Brazil Pontifícia Universidade Católica do Rio Grande do Sul (PUC-RS), Porto Alegre, RS – Brazil; 35 SalvadorBA Brazil Hospital Cárdio Pulmonar, Salvador, BA – Brazil; 36 LondrinaPR Brazil Universidade Estadual de Londrina, Londrina, PR – Brazil; 37 Rio de JaneiroRJ Brazil Rede D'Or São Luiz, Rio de Janeiro, RJ – Brazil; 38 Rio de JaneiroRJ Brazil Universidade do Estado do Rio de Janeiro (UERJ), Rio de Janeiro, RJ – Brazil; 39 São PauloSP Brazil Real Hospital Português, São Paulo, SP – Brazil; 40 SalvadorBA Brazil Universidade do Estado da Bahia (UNEB), Salvador, BA – Brazil; 41 SalvadorBA Brazil Universidade Salvador (UNIFACS), Salvador, BA – Brazil; 42 SalvadorBA Brazil Hospital EMEC, Salvador, BA – Brazil; 43 Porto AlegreRS Brazil Instituto de Cardiologia – Fundação Universitária de Cardiologia do Rio Grande do Sul, Porto Alegre, RS – Brazil; 44 CampinasSP Brazil Faculdade de Ciências Médicas da Universidade Estadual de Campinas (UNICAMP), Campinas, SP – Brazil; 45 Porto AlegreRS Brazil Santa Casa de Misericórdia de Porto Alegre, Porto Alegre, RS – Brazil; 46 CuritibaPR Brazil Faculdade de Medicina Evangélica Mackenzie, Curitiba, PR – Brazil; 47 MaceióAL Brazil Hospital do Coração de Alagoas, Maceió, AL – Brazil; 48 São PauloSP Brazil Santa Casa de Misericórdia da São Paulo, São Paulo, SP – Brazil; 49 Porto AlegreRS Brazil Universidade Federal de Ciências da Saúde de Porto Alegre, Porto Alegre, RS – Brazil; 50 Belo HorizonteMG Brazil Hospital Madre Teresa, Belo Horizonte, MG – Brazil; 51 Rio de JaneiroRJ Brazil Universidade Federal do Rio de Janeiro (UFRJ), Rio de Janeiro, RJ – Brazil; 52 FortalezaCE Brazil Universidade de Fortaleza (UNIFOR), Fortaleza, CE – Brazil; 53 CuritibaPR Brazil Cardiocare Clínica Cardiológica, Curitiba, PR – Brazil

**Table:** Brazilian Society of Cardiology Guidelines on Unstable Angina and Acute Myocardial Infarction without ST-Segment Elevation – 2021

The report below lists declarations of interest as reported to the SBC by the experts during the period of the development of these update, 2020.	
Expert	Type of relationship with industry
Alexandre de Matos Soeiro	Nothing to be declared
Alexandre Jorge de Andrade Negri	Financial declaration A - Economically relevant payments of any kind made to (i) you, (ii) your spouse/partner or any other person living with you, (iii) any legal person in which any of these is either a direct or indirect controlling owner, business partner, shareholder or participant; any payments received for lectures, lessons, training instruction, compensation, fees paid for participation in advisory boards, investigative boards or other committees, etc. From the brazilian or international pharmaceutical, orthosis, prosthesis, equipment and implants industry: - AMIB: Instructor of postgraduate courses.
Andre Franci	Nothing to be declared
Ari Timerman	Financial declaration A - Economically relevant payments of any kind made to (i) you, (ii) your spouse/partner or any other person living with you, (iii) any legal person in which any of these is either a direct or indirect controlling owner, business partner, shareholder or participant; any payments received for lectures, lessons, training instruction, compensation, fees paid for participation in advisory boards, investigative boards or other committees, etc. From the brazilian or international pharmaceutical, orthosis, prosthesis, equipment and implants industry: - Sanofi e Daiichi Sankyo: Aulas sobre Síndromes Coronárias Agudas. Other relationships Funding of continuing medical education activities, including travel, accommodation and registration in conferences and courses, from the brazilian or international pharmaceutical, orthosis, prosthesis, equipment and implants industry: - Sanofi: Travel to Congress.
Brivaldo Markman Filho	Nothing to be declared
Bruno Ferraz de Oliveira Gomes	Nothing to be declared
Bruno Mendonça Baccaro	Nothing to be declared
Carlos Eduardo Lucena Montenegro	Financial declaration A - Economically relevant payments of any kind made to (i) you, (ii) your spouse/partner or any other person living with you, (iii) any legal person in which any of these is either a direct or indirect controlling owner, business partner, shareholder or participant; any payments received for lectures, lessons, training instruction, compensation, fees paid for participation in advisory boards, investigative boards or other committees, etc. From the brazilian or international pharmaceutical, orthosis, prosthesis, equipment and implants industry: - Servier: Vastarel/Procoralan; Novartis: Entresto; Merk: Concor; Astrazeneca. Other relationships Funding of continuing medical education activities, including travel, accommodation and registration in conferences and courses, from the brazilian or international pharmaceutical, orthosis, prosthesis, equipment and implants industry: - Pfizer: Amyloidosis; Servier: CAD/HF; Novartis: HF.
Carlos Eduardo Rochitte	Nothing to be declared
Carlos José Dornas Gonçalves Barbosa	Financial declaration A - Economically relevant payments of any kind made to (i) you, (ii) your spouse/partner or any other person living with you, (iii) any legal person in which any of these is either a direct or indirect controlling owner, business partner, shareholder or participant; any payments received for lectures, lessons, training instruction, compensation, fees paid for participation in advisory boards, investigative boards or other committees, etc. From the brazilian or international pharmaceutical, orthosis, prosthesis, equipment and implants industry: - Daiichi Sankyo: Effient; Servier: Brilinta. Other relationships Funding of continuing medical education activities, including travel, accommodation and registration in conferences and courses, from the brazilian or international pharmaceutical, orthosis, prosthesis, equipment and implants industry: - Bayer: Xarelto; Daiichi Sankyo: Lixiana.
Cláudio Marcelo Bittencourt das Virgens	Financial declaration A - Economically relevant payments of any kind made to (i) you, (ii) your spouse/partner or any other person living with you, (iii) any legal person in which any of these is either a direct or indirect controlling owner, business partner, shareholder or participant; any payments received for lectures, lessons, training instruction, compensation, fees paid for participation in advisory boards, investigative boards or other committees, etc. From the brazilian or international pharmaceutical, orthosis, prosthesis, equipment and implants industry: - Novo Nordisk: Semaglutida; Daiichi Sankyo: Edoxabana.
Dalton Bertolim Précoma	Financial declaration A - Economically relevant payments of any kind made to (i) you, (ii) your spouse/partner or any other person living with you, (iii) any legal person in which any of these is either a direct or indirect controlling owner, business partner, shareholder or participant; any payments received for lectures, lessons, training instruction, compensation, fees paid for participation in advisory boards, investigative boards or other committees, etc. From the brazilian or international pharmaceutical, orthosis, prosthesis, equipment and implants industry: - Servier: Doença Coronariana; Bayer: Anticoagulates; Daiichi Sankyo: Anticoagulantes; Novo Nordisk. B - Research funding under your direct/personal responsibility (directed to the department or institution) from the brazilian or international pharmaceutical, orthosis, prosthesis, equipment and implants industry: - Novartis: Insuficiência Cardíaca; Apellis: Antiinflamatório para Covid; Astrazeneca: Antidiabético/Insuficiência Cardíaca; Boehringer. Other relationships Funding of continuing medical education activities, including travel, accommodation and registration in conferences and courses, from the brazilian or international pharmaceutical, orthosis, prosthesis, equipment and implants industry: - Bayer: Anticoagulant; Novo Nordisk: Antidiabetic; Servier: CAD; Daiichi Sankyo.
Edson Stefanini	Nothing to be declared
Eduardo Cavalcanti Lapa Santos	Nothing to be declared
Euler Roberto Fernandes Manent	Financial declaration A - Economically relevant payments of any kind made to (i) you, (ii) your spouse/partner or any other person living with you, (iii) any legal person in which any of these is either a direct or indirect controlling owner, business partner, shareholder or participant; any payments received for lectures, lessons, training instruction, compensation, fees paid for participation in advisory boards, investigative boards or other committees, etc. From the brazilian or international pharmaceutical, orthosis, prosthesis, equipment and implants industry: - Bayer e Pfizer: Anticoagulant; Daiichi Sankyo: Antiplatelet agents; Boeheringer. B - Research funding under your direct/personal responsibility (directed to the department or institution) from the brazilian or international pharmaceutical, orthosis, prosthesis, equipment and implants industry: - Astrazeneca: Antiplatelet agents, ISGLT2; Daiichi Sankyo: Antiplatelet agents; Astra Zeneca: Heart Failure, Antiplatelet agents. C - Personal research funding paid by the brazilian or international pharmaceutical, orthosis, prosthesis, equipment and implants industry: - Boerinhger: Heart failure; AstraZeneca: Dapaglifozina. Other relationships Funding of continuing medical education activities, including travel, accommodation and registration in conferences and courses, from the brazilian or international pharmaceutical, orthosis, prosthesis, equipment and implants industry: - Bayer: Anticoagulant.
Felipe Gallego Lima	Financial declaration A - Economically relevant payments of any kind made to (i) you, (ii) your spouse/partner or any other person living with you, (iii) any legal person in which any of these is either a direct or indirect controlling owner, business partner, shareholder or participant; any payments received for lectures, lessons, training instruction, compensation, fees paid for participation in advisory boards, investigative boards or other committees, etc. From the brazilian or international pharmaceutical, orthosis, prosthesis, equipment and implants industry: - Sanofi: Clexane; Servier: Ticagrelor. Other relationships Funding of continuing medical education activities, including travel, accommodation and registration in conferences and courses, from the brazilian or international pharmaceutical, orthosis, prosthesis, equipment and implants industry: - Sanofi: Enoxaparina; Servier: Ticagrelor.
Francisco das Chagas Monteiro Júnior	Nothing to be declared
Gilson Soares Feitosa Filho	Financial declaration A - Economically relevant payments of any kind made to (i) you, (ii) your spouse/partner or any other person living with you, (iii) any legal person in which any of these is either a direct or indirect controlling owner, business partner, shareholder or participant; any payments received for lectures, lessons, training instruction, compensation, fees paid for participation in advisory boards, investigative boards or other committees, etc. From the brazilian or international pharmaceutical, orthosis, prosthesis, equipment and implants industry: - Bayer: COMPASS Study.
Harry Correa Filho	Financial declaration A - Economically relevant payments of any kind made to (i) you, (ii) your spouse/partner or any other person living with you, (iii) any legal person in which any of these is either a direct or indirect controlling owner, business partner, shareholder or participant; any payments received for lectures, lessons, training instruction, compensation, fees paid for participation in advisory boards, investigative boards or other committees, etc. From the brazilian or international pharmaceutical, orthosis, prosthesis, equipment and implants industry: - Bayer: Anticoagulation.
Henrique Patrus Mundim Pena	Financial declaration A - Economically relevant payments of any kind made to (i) you, (ii) your spouse/partner or any other person living with you, (iii) any legal person in which any of these is either a direct or indirect controlling owner, business partner, shareholder or participant; any payments received for lectures, lessons, training instruction, compensation, fees paid for participation in advisory boards, investigative boards or other committees, etc. From the brazilian or international pharmaceutical, orthosis, prosthesis, equipment and implants industry: - Lilly: Diabetes and Cardiovascular Risk; Daiichi Sankyo: Antiaggregator Platelet; Pfizer and Bayer: Oral Anticoagulant. Other relationships Funding of continuing medical education activities, including travel, accommodation and registration in conferences and courses, from the brazilian or international pharmaceutical, orthosis, prosthesis, equipment and implants industry: - Pfizer and Bayer: Oral Anticoagulant; Daiichi Sankyo: Platelet Antiaggregator.
Ibraim Masciarelli Francisco Pinto	Financial declaration A - Economically relevant payments of any kind made to (i) you, (ii) your spouse/partner or any other person living with you, (iii) any legal person in which any of these is either a direct or indirect controlling owner, business partner, shareholder or participant; any payments received for lectures, lessons, training instruction, compensation, fees paid for participation in advisory boards, investigative boards or other committees, etc. From the brazilian or international pharmaceutical, orthosis, prosthesis, equipment and implants industry: - Novo Nordisk: Diabetes.
João Luiz de Alencar Araripe Falcão	Nothing to be declared
João Luiz Petriz	Financial declaration A - Economically relevant payments of any kind made to (i) you, (ii) your spouse/partner or any other person living with you, (iii) any legal person in which any of these is either a direct or indirect controlling owner, business partner, shareholder or participant; any payments received for lectures, lessons, training instruction, compensation, fees paid for participation in advisory boards, investigative boards or other committees, etc. From the brazilian or international pharmaceutical, orthosis, prosthesis, equipment and implants industry: - Daiichi Sankyo e Servier: Cardiology.
Joberto Pinheiro Sena	Financial declaration A - Economically relevant payments of any kind made to (i) you, (ii) your spouse/partner or any other person living with you, (iii) any legal person in which any of these is either a direct or indirect controlling owner, business partner, shareholder or participant; any payments received for lectures, lessons, training instruction, compensation, fees paid for participation in advisory boards, investigative boards or other committees, etc. From the brazilian or international pharmaceutical, orthosis, prosthesis, equipment and implants industry: - Servier: Lectures on SCA; Bayer: Classes in the management of anticoagulants and antithrombotics; Daiichi Sankyo: Antiplatelet management in ACS.
José A. Marin-Neto	Nothing to be declared
José Carlos Nicolau	Financial declaration A - Economically relevant payments of any kind made to (i) you, (ii) your spouse/partner or any other person living with you, (iii) any legal person in which any of these is either a direct or indirect controlling owner, business partner, shareholder or participant; any payments received for lectures, lessons, training instruction, compensation, fees paid for participation in advisory boards, investigative boards or other committees, etc. From the brazilian or international pharmaceutical, orthosis, prosthesis, equipment and implants industry: - Amgen: Lipid-lowering; Bayer: Anticoagulant; Daiichi Sankyo: Antiplatelet, anticoagulant; Novartis: SRA inhibitor, lipid-lowering; Sanofi: Anticoagulant, lipid-lowering, antiplatelet; Servier: antiplatelet. B - Research funding under your direct/personal responsibility (directed to the department or institution) from the brazilian or international pharmaceutical, orthosis, prosthesis, equipment and implants industry: - Astrazeneca: Antiplatelet, hypoglycemic; Bayer: Anticoagulant; Esperion: Lipid-lowering; CSL Behring: lipid-lowering; Dalcor: HDL increase; Jansen: Anticoagulant; Novartis: SARS inhibitor; Novo Nordisk: Hypoglycemic; Sanofi: Anticoagulant, lipid-lowering; Vifor: Iron Deficiency. C - Personal research funding paid by the brazilian or international pharmaceutical, orthosis, prosthesis, equipment and implants industry: - Roche: Laboratory kits for platelet aggregability assessment. Other relationships Funding of continuing medical education activities, including travel, accommodation and registration in conferences and courses, from the brazilian or international pharmaceutical, orthosis, prosthesis, equipment and implants industry: - Sanofi: Anticoagulant, antiplatelet, lipid-lowering; Bayer: Anticoagulant; Servier: Antiplatelet.
José Maria Peixoto	Nothing to be declared
Juliana Ascenção de Souza	Nothing to be declared
Leonardo Sara da Silva	Nothing to be declared
Leopoldo Soares Piegas	Nothing to be declared
Lilia Nigro Maia	Nothing to be declared
Louis Nakayama Ohe	Financial declaration A - Economically relevant payments of any kind made to (i) you, (ii) your spouse/partner or any other person living with you, (iii) any legal person in which any of these is either a direct or indirect controlling owner, business partner, shareholder or participant; any payments received for lectures, lessons, training instruction, compensation, fees paid for participation in advisory boards, investigative boards or other committees, etc. From the brazilian or international pharmaceutical, orthosis, prosthesis, equipment and implants industry: - Daiichi Sankyo: Edoxabana. Other relationships Funding of continuing medical education activities, including travel, accommodation and registration in conferences and courses, from the brazilian or international pharmaceutical, orthosis, prosthesis, equipment and implants industry: - Abbott e Meryl: Hemodynamic. Participation in procurement committees for supplies or drugs in health institutions or any similar roles taken: - Committee for the purchase of appliances for Covid-19 units.
Luciano Moreira Baracioli	Financial declaration A - Economically relevant payments of any kind made to (i) you, (ii) your spouse/partner or any other person living with you, (iii) any legal person in which any of these is either a direct or indirect controlling owner, business partner, shareholder or participant; any payments received for lectures, lessons, training instruction, compensation, fees paid for participation in advisory boards, investigative boards or other committees, etc. From the brazilian or international pharmaceutical, orthosis, prosthesis, equipment and implants industry: - Sandoz: Clopidogrel; Bayer: Rivaroxabana. Other relationships Funding of continuing medical education activities, including travel, accommodation and registration in conferences and courses, from the brazilian or international pharmaceutical, orthosis, prosthesis, equipment and implants industry: - Bayer: Anticoagulation.
Luís Alberto de Oliveira Dallan	Other relationships Participation in procurement committees for supplies or drugs in health institutions or any similar roles taken: - Proclamation.
Luis Augusto Palma Dallan	Nothing to be declared
Luiz Alberto Piva e Mattos	Nothing to be declared
Luiz Bezerra Neto	Financial declaration A - Economically relevant payments of any kind made to (i) you, (ii) your spouse/partner or any other person living with you, (iii) any legal person in which any of these is either a direct or indirect controlling owner, business partner, shareholder or participant; any payments received for lectures, lessons, training instruction, compensation, fees paid for participation in advisory boards, investigative boards or other committees, etc. From the brazilian or international pharmaceutical, orthosis, prosthesis, equipment and implants industry: - Pfizer and Bayer: Anticoagulant class; Libbs: Antihypertensive class. Other relationships Any economically relevant equity interest in companies in the healthcare or education industry or in any companies competing with or supplying to SBC: - Partner at L&P Cardio, a medical services company.
Luiz Carlos Bodanese	Nothing to be declared
Luiz Eduardo Fonteles Ritt	Financial declaration A - Economically relevant payments of any kind made to (i) you, (ii) your spouse/partner or any other person living with you, (iii) any legal person in which any of these is either a direct or indirect controlling owner, business partner, shareholder or participant; any payments received for lectures, lessons, training instruction, compensation, fees paid for participation in advisory boards, investigative boards or other committees, etc. From the brazilian or international pharmaceutical, orthosis, prosthesis, equipment and implants industry: - Pfizer: Amyloid.
Manoel Fernandes Canesin	Nothing to be declared
Marcelo Bueno da Silva Rivas	Nothing to be declared
Marcelo Franken	Nothing to be declared
Marcos José Gomes Magalhães	Nothing to be declared
Múcio Tavares de Oliveira Júnior	Nothing to be declared
Nivaldo Menezes Filgueiras Filho	Financial declaration A - Economically relevant payments of any kind made to (i) you, (ii) your spouse/partner or any other person living with you, (iii) any legal person in which any of these is either a direct or indirect controlling owner, business partner, shareholder or participant; any payments received for lectures, lessons, training instruction, compensation, fees paid for participation in advisory boards, investigative boards or other committees, etc. From the brazilian or international pharmaceutical, orthosis, prosthesis, equipment and implants industry: - Pfizer: Eliquis, Apixabana; Boehringer: Pradaxa, Praxbind, Jardiance; Astrazenca: Forxiga; Novo Nordisk; Daiichi Sankyo; Novartis; Bayer. B - Research funding under your direct/personal responsibility (directed to the department or institution) from the brazilian or international pharmaceutical, orthosis, prosthesis, equipment and implants industry: - Astrazeneca: Dapaglifozina. Other relationships Funding of continuing medical education activities, including travel, accommodation and registration in conferences and courses, from the brazilian or international pharmaceutical, orthosis, prosthesis, equipment and implants industry: - Pfizer: Eliquis; Daiichi Sankyo: Effient; Bayer: Xarelto.
Oscar Pereira Dutra	Financial declaration A - Economically relevant payments of any kind made to (i) you, (ii) your spouse/partner or any other person living with you, (iii) any legal person in which any of these is either a direct or indirect controlling owner, business partner, shareholder or participant; any payments received for lectures, lessons, training instruction, compensation, fees paid for participation in advisory boards, investigative boards or other committees, etc. From the brazilian or international pharmaceutical, orthosis, prosthesis, equipment and implants industry: - Astrazeneca: Antiglycemic; Daiichi Sankyo: Anticoagulants; Sandoz: Antihypertensives. B - Research funding under your direct/personal responsibility (directed to the department or institution) from the brazilian or international pharmaceutical, orthosis, prosthesis, equipment and implants industry: - Boheringer: Antiglycemic; Novartis: Heart Failure; Sanofi: Antilipemics. C - Personal research funding paid by the brazilian or international pharmaceutical, orthosis, prosthesis, equipment and implants industry: - Sanofi e EMS: Anticoagulants; Amgen: Antilipemics. Other relationships Funding of continuing medical education activities, including travel, accommodation and registration in conferences and courses, from the brazilian or international pharmaceutical, orthosis, prosthesis, equipment and implants industry: - Bayer: Anticoagulants.
Otávio Rizzi Coelho	Financial declaration B - Research funding under your direct/personal responsibility (directed to the department or institution) from the brazilian or international pharmaceutical, orthosis, prosthesis, equipment and implants industry: - Boheringer: Empaglifozina. Other relationships Funding of continuing medical education activities, including travel, accommodation and registration in conferences and courses, from the brazilian or international pharmaceutical, orthosis, prosthesis, equipment and implants industry: - Daiichi Sankyo: Prasugrel; Bayer: Rivaroxabana; Astrazeneca: Dapaglifozina.
Paulo Ernesto Leães	Other relationships Funding of continuing medical education activities, including travel, accommodation and registration in conferences and courses, from the brazilian or international pharmaceutical, orthosis, prosthesis, equipment and implants industry: - Boehringer: Anticoagulantes Participation in procurement committees for supplies or drugs in health institutions or any similar roles taken: Comitê de Padronização da Santa Casa de Porto Alegre
Paulo Roberto Ferreira Rossi	Financial declaration A - Economically relevant payments of any kind made to (i) you, (ii) your spouse/partner or any other person living with you, (iii) any legal person in which any of these is either a direct or indirect controlling owner, business partner, shareholder or participant; any payments received for lectures, lessons, training instruction, compensation, fees paid for participation in advisory boards, investigative boards or other committees, etc. From the brazilian or international pharmaceutical, orthosis, prosthesis, equipment and implants industry: - Novartis: Sacubitril, Valsartan.
Paulo Rogério Soares	Financial declaration A - Economically relevant payments of any kind made to (i) you, (ii) your spouse/partner or any other person living with you, (iii) any legal person in which any of these is either a direct or indirect controlling owner, business partner, shareholder or participant; any payments received for lectures, lessons, training instruction, compensation, fees paid for participation in advisory boards, investigative boards or other committees, etc. From the brazilian or international pharmaceutical, orthosis, prosthesis, equipment and implants industry: - Astrazeneca: Dapagliflozina; Servier: Ticagrelor; Bayer: Rivaroxabana.
Pedro Alves Lemos Neto	Nothing to be declared
Pedro Silvio Farsky	Financial declaration A - Economically relevant payments of any kind made to (i) you, (ii) your spouse/partner or any other person living with you, (iii) any legal person in which any of these is either a direct or indirect controlling owner, business partner, shareholder or participant; any payments received for lectures, lessons, training instruction, compensation, fees paid for participation in advisory boards, investigative boards or other committees, etc. From the brazilian or international pharmaceutical, orthosis, prosthesis, equipment and implants industry: - Alliance Boehringer Ingelheim/Lilly: I -SGLT2.
Rafael Rebêlo C. Cavalcanti	Nothing to be declared
Remo Holanda de Mendonça Furtado	Financial declaration A - Economically relevant payments of any kind made to (i) you, (ii) your spouse/partner or any other person living with you, (iii) any legal person in which any of these is either a direct or indirect controlling owner, business partner, shareholder or participant; any payments received for lectures, lessons, training instruction, compensation, fees paid for participation in advisory boards, investigative boards or other committees, etc. From the brazilian or international pharmaceutical, orthosis, prosthesis, equipment and implants industry: - Astra Zeneca: Dapagliflozin (Diabetes, Heart Failure); Servier: Ticagrelor; Bayer: Rivaroxabana. B - Research funding under your direct/personal responsibility (directed to the department or institution) from the brazilian or international pharmaceutical, orthosis, prosthesis, equipment and implants industry: - Bayer: Rivaroxabana (Covid); Astra Zeneca: Dapagliflozina (Covid), Dapagliflozina (HF); Pfizer: Tofacitinib (Covid), Apixabana (AF); EMS, Aché, Servier, Health Canada, GSK, Servier.
Renato Abdala Karam Kalil	Nothing to be declared
Renato Delascio Lopes	Financial declaration A - Economically relevant payments of any kind made to (i) you, (ii) your spouse/partner or any other person living with you, (iii) any legal person in which any of these is either a direct or indirect controlling owner, business partner, shareholder or participant; any payments received for lectures, lessons, training instruction, compensation, fees paid for participation in advisory boards, investigative boards or other committees, etc. From the brazilian or international pharmaceutical, orthosis, prosthesis, equipment and implants industry: - Bayer: Anticoagulante; Boehringer Ingleheim: Anticoagulação e Diabetes; Pfizer: Anticoagulação; Bristol-Myers Squibb; Daiichi Sankyo; Glaxo Smith Kline; Medtronic; Merck; Portola; Sanofi. B - Research funding under your direct/personal responsibility (directed to the department or institution) from the brazilian or international pharmaceutical, orthosis, prosthesis, equipment and implants industry: - Pfizer: Apixaban; Bayer: Rivaroxaban; Novartis: Sacubitril, Valsartan. Other relationships Funding of continuing medical education activities, including travel, accommodation and registration in conferences and courses, from the brazilian or international pharmaceutical, orthosis, prosthesis, equipment and implants industry: - Bayer: Rivaroxabana; Pfizer: Apixabana.
Renato Jorge Alves	Financial declaration A - Economically relevant payments of any kind made to (i) you, (ii) your spouse/partner or any other person living with you, (iii) any legal person in which any of these is either a direct or indirect controlling owner, business partner, shareholder or participant; any payments received for lectures, lessons, training instruction, compensation, fees paid for participation in advisory boards, investigative boards or other committees, etc. From the brazilian or international pharmaceutical, orthosis, prosthesis, equipment and implants industry: - Amgen: Evolocumabe; PTC: Volanesorsen; Pfizer: Apixaban.
Roberto Esporcatte	Financial declaration A - Economically relevant payments of any kind made to (i) you, (ii) your spouse/partner or any other person living with you, (iii) any legal person in which any of these is either a direct or indirect controlling owner, business partner, shareholder or participant; any payments received for lectures, lessons, training instruction, compensation, fees paid for participation in advisory boards, investigative boards or other committees, etc. From the brazilian or international pharmaceutical, orthosis, prosthesis, equipment and implants industry: - Bayer: Anticoagulation.
Roberto Luiz Marino	Financial declaration B - Research funding under your direct/personal responsibility (directed to the department or institution) from the brazilian or international pharmaceutical, orthosis, prosthesis, equipment and implants industry: - CSL Behring:Myocardial Infarction.
Roberto Rocha Corrêa Veiga Giraldez	Nothing to be declared
Romeu Sérgio Meneghelo	Nothing to be declared
Ronaldo de Souza Leão Lima	Other relationships Any economically relevant equity interest in companies in the healthcare or education industry or in any companies competing with or supplying to SBC: - Fonte Imagem: Minority partner.
Rui Fernando Ramos	Financial declaration A - Economically relevant payments of any kind made to (i) you, (ii) your spouse/partner or any other person living with you, (iii) any legal person in which any of these is either a direct or indirect controlling owner, business partner, shareholder or participant; any payments received for lectures, lessons, training instruction, compensation, fees paid for participation in advisory boards, investigative boards or other committees, etc. From the brazilian or international pharmaceutical, orthosis, prosthesis, equipment and implants industry: - Aspen: Fondaparinux. Other relationships Funding of continuing medical education activities, including travel, accommodation and registration in conferences and courses, from the brazilian or international pharmaceutical, orthosis, prosthesis, equipment and implants industry: - Aspen: Fondaparinux.
Sandra Nivea dos Reis Saraiva Falcão	Nothing to be declared
Talia Falcão Dalçóquio	Financial declaration B - Research funding under your direct/personal responsibility (directed to the department or institution) from the brazilian or international pharmaceutical, orthosis, prosthesis, equipment and implants industry: - Amgen, DalCor Pharma UK Ltd, CSL Behring LLC: Pharmaceutical.
Viviana de Mello Guzzo Lemke	Nothing to be declared
Walmor Lemke	Nothing to be declared
William Azem Chalela	Nothing to be declared
Wilson Mathias Júnior	Nothing to be declared

**Table:** Brazilian Society of Cardiology Guidelines on Unstable Angina and Acute Myocardial Infarction without ST-Segment Elevation – 2021

**General Coordinator:** José C. Nicolau; **Assistant and Coordination:** Remo H.M. Furtado; **Supervisor of the English version:** Renato D. Lopes; Secretary: Daniele Gullo		
**Part 1 - Emergency Assessment and Treatment Protocols**	**Part 2 - Treatment Protocols during Hospitalization**	**Part 3: Recommendations at Discharge and Post-Discharge Care**
Coordinators:	Coordinators:	Coordinators:
Ari Timerman	Leopoldo S. Piegas	Gilson Feitosa Filho
João Luiz Petriz	José A. Marin-Netto	Luiz Bezerra Neto
Bruno Ferraz	Dalton Précoma	Eduardo Lapa
	Walmor Lemke	
**Reviewers Part 1**	**Reviewers Part 2**	**Reviewers Part 3**
Brivaldo Markman Filho	Andre Franci	Alexandre de Matos Soeiro
Bruno Mendonça Baccaro	Edson Stefanini	Alexandre Jorge de Andrade Negri
Carlos Eduardo Rochitte	Euler Roberto Fernandes Manenti	Carlos Eduardo Lucena Montenegro
Leonardo Sara	Felipe Galego Lima	Cláudio Marcelo Bittencourt das Virgens
Carlos Jose Dornas Gonçalves Barbosa	Harry Correa Filho	Francisco das Chagas Monteiro Júnior
Louis Nakayama Ohe	Ibraim Masciarelli Francisco Pinto	Henrique Patrus Mundim Pena
Luiz Carlos Bodanese	Lilia Nigro Maia	João Luiz de Alencar Araripe Falcão
Marcelo Bueno da Silva Rivas	Luciano Moreira Baracioli	Joberto Pinheiro Sena
Paulo Roberto Ferreira Rossi	Luís Alberto de Oliveira Dallan	José Maria Peixoto
Paulo Rogério Soares	Luis Augusto P. Dallan	Juliana Ascenção de Souza
Pedro Alves Lemos Neto	Luiz Alberto Piva e Mattos	Luiz Eduardo Fonteles Ritt
Renato Delascio Lopes	Marcelo Franken	Manoel Fernandes Canesin
Roberto Esporcatte	Oscar Pereira Dutra	Marcos José Gomes Magalhães
Roberto Luiz Marino	Otávio Rizzi Coelho	Múcio Tavares de Oliveira Júnior
Roberto Rocha Corrêa Veiga Giraldez	Paulo Ernesto Leães	Nivaldo Menezes Filgueiras Filho
Rui Fernando Ramos	Renato Kalil	Pedro Silvio Farsky
Talia Falcão Dalçóquio	Romeu Sérgio Meneghelo	Rafael Rebêlo C. Cavalcanti
William Azem Chalela	Ronaldo de Souza Leão Lima	Renato Jorge Alves
	Vivivana Lemke	Sandra Nives dos Reis Saraiva Falcão
	Wilson Mathias Júnior	

## Definitions of Recommendations and Evidence

### Recommendations

**Class I:** Conclusive evidence, or, failing that, general consensus that the procedure is safe and useful/effective.
**Class II:** Conflicting evidence and/or divergence of opinion about the procedure's safety and usefulness/effectiveness.
**Class IIa:** Evidence/opinion is in favor of the procedure. Most approve of it.
**Class IIb:** The procedure's safety and utility/effectiveness is less well established, opinion is not predominantly in favor of it.
**Class III:** Evidence and/or consensus that the procedure is not useful/effective and, in some cases, is harmful.

### Evidence

**Level A:** Data obtained from multiple large randomized studies, concordant and/or robust meta-analysis of randomized clinical studies.
**Level B:** Data obtained from less robust meta-analysis, from a single randomized study or from non-randomized (observational) studies.
**Level C:** Data obtained from expert consensus.

## Changes in recommendations

**Table:** 

Changes in recommendations - Biochemical Markers					
2014 Guideline			2020 Guideline		
Biomarkers of myocardial necrosis should be measured in all patients suspected of having non-ST-elevation acute coronary syndromes (NSTE-ACS). The markers should be measured at admission and repeated at least once 6 to 9 hours (preferably 9 to 12 hours) after symptom onset if the first dosage is normal or slightly elevated.	I	C	Biomarkers of myocardial necrosis should be measured in all patients with suspected NSTE-ACS. When highly sensitive troponin assays are available, serum levels should be measured at admission and, ideally, reassessed in 1 h or up to 2 h. If unavailable, conventional troponin measurement should be performed at admission and repeated at least once, 3 to 6 hours later, if the initial results are normal or slightly elevated.	I	B
Creatine kinase-MB (CK-MB) mass and troponins are the biochemical markers of choice.	I	A	CK-MB mass levels can be used if troponin levels are not available.	IIb	B
For patients who arrive early at the emergency department (6 h prior to symptom onset), myoglobin and highly sensitive troponin may be considered in addition to a later marker (CK-MB or troponin).	IIb	B	Troponins are the biomarkers of choice in patients with suspected AMI.	I	A
			Myoglobin can be used to detect myocardial necrosis in patients with suspected NSTE-ACS.	III	

**Table:** 

Changes in recommendations - Exercise test					
2014 Guideline			2020 Guideline		
Low-risk patients (clinical and electrocardiogram [ECG]) with normal biochemical markers should be referred for exercise testing (ET) after 9 h of observation, ideally by 12 h, on an outpatient basis.	I	B	Low-risk patients (clinical and electrocardiogram [ECG]) with normal biomarkers should be referred for ET after 9 to 12 h of observation. These exams can be performed the in chest pain unit as a discharge criterion.	I	B

**Table:** 

Changes in recommendations - Nuclear cardiology					
2014 Guideline			2020 Guideline		
Stress-rest myocardial perfusion scintigraphy (MPS) is an alternative for patients unable to perform ET.	I	C	Stress myocardial perfusion scintigraphy can be used as a stratification method in patients without recurrent chest pain and without ECG evidence of ischemia and/or troponin elevation.	I	B
Patients with chest pain can be evaluated by rest MPS to determine whether the origin of the pain is ischemic or non-ischemic	IIa	A	Patients with chest pain but without ischemic ECG changes can be evaluated by myocardial perfusion scintigraphy at rest to determine whether the origin of the pain is ischemic or not.	IIa	A

**Table:** 

Changes in recommendations - Analgesia and sedation					
2014 Guideline			2020 Guideline		
Administer morphine sulfate to intermediate- and high-risk patients.	I	C	Administer morphine sulfate to patients with continuous pain despite optimized anti-ischemic therapy	IIb	B
Administer benzodiazepines to high-risk patients.	I	C	Administer benzodiazepines to patients with signs and symptoms of persistent anxiety.	IIb	C
Administer benzodiazepines to intermediate-risk patients.	IIa	C			

**Table:** 

Changes in recommendations - Nitrates					
2014 Guideline			2020 Guideline		
Nitrates can be used in intermediate- and high-risk patients.	I	C	Sublingual nitrates can be used to relieve angina.	I	C
			Intravenous nitrates can be used to control persistent angina, high blood pressure or signs of congestion.	I	C

**Table:** 

Changes in recommendations - Beta-blockers					
2014 Guideline			2020 Guideline		
Administer oral beta-blockers to intermediate- and high-risk patients.	I	B	Administer oral beta-blockers in the first 24 hours to patients without contraindications (signs of heart failure (HF), signs of low cardiac output, increased risk of cardiogenic shock or other contraindications to beta-blockers).	IIa	B

**Table:** 

Changes in recommendations - Use of antiplatelet devices in the Emergency Department					
2014 Guideline			2020 Guideline		
Thienopyridines should be used in patients contraindicated to acetylsalicylic acid (ASA).	I	B	In patients allergic to ASA, initial monotherapy with a P2Y_12_ inhibitor (preferentially ticagrelor or prasugrel) is indicated.	I	C

**Table:** 

Changes in recommendations - Glycoprotein IIb/IIIa Inhibitors					
2014 Guideline			2020 Guideline		
Glycoprotein (GP) IIb/IIIa inhibitors should be used in patients with recurrent ischemic symptoms despite double oral platelet antiaggregation and anticoagulation.	IIa	C	In the conservative strategy, adjunct tirofiban can be used in patients with recurrent ischemic symptoms despite treatment with double oral platelet antiaggregation and anticoagulation.	IIb	C

**Table:** 

Changes in recommendations - Anticoagulants					
2014 Guideline			2020 Guideline		
Unfractionated heparin (UFH) can be used in all patients.	I	A	UFH is preferential in patients with severe renal dysfunction (< 15 mL/min) and obese patients (weighing > 150 kg).	IIa	B
Low-molecular-weight heparins (LMWH) can be used in all patients.	I	A	Enoxaparin can be used in patients without severe renal dysfunction (creatinine clearance < 15 mL/min/1.73 m²), until revascularization, for 8 days, or until hospital discharge, 1 mg/kg every 12 h (0.75 mg/kg, every 12 h, if ≥ 75 years old; use 1 mg/kg every 24 h if creatinine clearance is between 15 and 30 mL/min/1.73 m², with a maximum dose of 150 mg.	I	A

## New recommendations

**Table:** 

History and physical examination		
Patients with suspected NSTE-ACS and persistent pain, dyspnea, palpitations or syncope should be referred to the emergency department, ideally monitored in an ambulance.	I	C
Patients with suspected NSTE-ACS with less severe findings (ie, without persistent pain, dyspnea, palpitations or syncope) should be instructed to seek out an emergency department that can perform ECG and troponin measurement.	IIb	C

**Table:** 

Electrocardiogram		
ECG should be repeated if symptoms recur.	I	C
V3R-V4R and V7-V9 lead ECGs should be performed on patients with a non-diagnostic 12-lead ECG who remain symptomatic.	I	C

**Table:** 

Biochemical markers		
When troponin is available, no other marker should be requested for diagnostic purposes.	I	B

**Table:** 

Coronary computed tomography angiography		
Investigate acute chest pain with the triple rule-out technique.	IIb	B

**Table:** 

Diagnostic routine and hospitalization criteria		
The initial screening should be based on clinical history, physical examination, 12-lead ECG in up to 10 min, and troponin level.	I	A
HEART scores should be used to stratify risk and assist in hospital discharge decisions.	I	B
Patients with HEART scores ≤3 associated with negative troponin results, ECG without ischemic change, and no history of coronary artery disease (CAD) can be released from the emergency department for outpatient reassessment.	I	B
Hospital screening can be performed by qualified nurses for early recognition of high-risk patients.	IIa	B
EDACS and ADAPT scores for clinical risk stratification can be used as alternatives to the HEART score.	IIa	B
The HEART score can be used to determine early release for patients treated in an ambulance.	IIb	C

**Table:** 

Early invasive strategy		
An invasive strategy is recommended for patients with NSTE-ACS and refractory angina and/or hemodynamic and/or electrical instability (without serious comorbidities or contraindications for these procedures).	I	A

**Table:** 

Glycemic control		
It is recommended to measure the glycemic levels of all patients with suspected AMI at admission; the blood glucose of diabetic patients and those with hyperglycemia during hospitalization should be monitored.	I	C
Glycemic control with intermittent insulin use protocols should be carefully considered in patients with glycemic levels > 180 mg/dL to avoid episodes of hypoglycemia.	IIa	C
In patients at increased risk of hypoglycemia, such as older adults, nephropaths, and patients with residual effects from oral and/or fasting hypoglycemic agents, glycemic control should be adjusted to allow slightly higher blood glucose levels, thus preventing hypoglycemia.	IIa	C

**Table:** 

Beta-blockers	
Administer beta-blockers intravenously in patients with risk factors for cardiogenic shock.	III

**Table:** 

Initial antiplatelet therapy		
Do not perform pretreatment with a P2Y_12_ inhibitor in unstable and/or high-risk patients indicated for immediate invasive strategy; it is recommended instead for the catheterization room after the coronary anatomy has been determined and percutaneous coronary intervention (PCI) has been scheduled.	I	B
P2Y_12_ inhibitors should not be routinely used as a pretreatment in patients indicated for early invasive strategy (<24 h).	I	B
In patients with a very high risk of bleeding or who need long-term oral anticoagulation, clopidogrel (loading dose) can be used regardless of an initial conservative or invasive strategy.	IIa	C
Pretreatment with prasugrel is not recommended.	III	

**Table:** 

Anticoagulants		
Fondaparinux 2.5 mg subcutaneously once a day can be used for 8 days or until hospital discharge as an alternative to enoxaparin, especially in patients with a high risk of bleeding.	I	B
Monitor anti-factor Xa in patients with creatinine clearance between 15 and 30 mL/min and in obese patients (100 to 150 kg) who are using enoxaparin.	IIa	B
Enoxaparin is preferential to UFH in patients with creatinine clearance ≥15 mL/min/1.73 m²), unless myocardial revascularization surgery is scheduled in the next 24 h.	IIa	B
UFH is preferential in the emergency department or hemodynamics unit in very high-risk patients with immediate catheterization scheduled (< 2 h).	IIa	C
Enoxaparin can be used in patients with creatinine clearance < 15 mL/min and weight > 150 kg.	III	
Fondaparinux can be used in patients with creatinine clearance < 20 mL/min.	III	

## Part 1 – Emergency Assessment and Management

### 1. Introduction

Acute chest pain is responsible for more than 5% of all emergency department visits and up to 10% of non-trauma related visits. The incidence of chest pain varies between 9 and 19 per 1,000 people/year seen in emergency departments and is involved in up to 40% of hospitalizations. Although most of these patients are discharged with a diagnosis of unspecified or non-cardiac chest pain, about 25% are diagnosed with acute coronary syndrome (ACS).^1^^,^^2^

The subgroup of patients whose electrocardiography (ECG) results show no ST-segment elevation but whose symptoms or complementary exam results are compatible with a coronary etiology are classified as having non-ST-elevation acute coronary syndromes (NSTE-ACS), the subject of the present guideline. NSTE-ACS can cause significant morbidity and mortality if not promptly and appropriately treated. Since delaying appropriate treatment can result in serious adverse events, it is important to verify the presence of NSTE-ACS by assessing clinical data and complementary exams.^3^

### 2. Definitions

Chest pain is the main symptom in patients with ACS. ECG must be performed and interpreted within the first 10 minutes of medical contact in patients suspected of ACS, and the results may differentiate patients into two groups:

- ST-elevation ACS: Patients with acute chest pain and persistent ST-segment elevation or new or presumably new left bundle branch block, which is a condition generally related to coronary occlusion and the need for immediate reperfusion.
- NSTE-ACS: Patients with acute chest pain without persistent ST-segment elevation, whether associated or not with other ECG changes suggestive of some type of myocardial ischemia, including a broad spectrum of severity: transient ST-segment elevation, transient or persistent ST-segment depression, T-wave inversion, other nonspecific T-wave changes (flat or pseudonormalization), and even normal ECG results. This group includes patients with unstable angina (UA), ie, with no changes in myocardial necrosis markers and those with non-ST-segment elevation myocardial infarction (NSTEMI) when there is an increase in myocardial necrosis markers.

A NSTEMI diagnosis is confirmed when there is an acute myocardial injury, which is confirmed by an increase in troponin levels, and the injury is suspected to be caused by ischemia. Based on its pathophysiology and clinical context, acute myocardial infarction (AMI) is classified into several subtypes (Tables 1.1 and 1.2). Increased troponin levels can be secondary to myocardial ischemia, but they can also occur in other clinical situations (Table 1.3).

**Table 1.1:** Myocardial injury and infarction^1^

Definition of myocardial injury
The term myocardial injury should be used in patients with cardiac troponin values > 99th percentile of the upper reference limit. The injury is considered acute if there is dynamic behavior (rising and/or falling from baseline). Persistently high troponin values are considered chronic myocardial injury.
**Definition of acute myocardial infarction (AMI types 1, 2 and 3)**
AMI implies an acute myocardial injury in a clinical context of myocardial ischemia: Symptoms suggestive of acute myocardial ischemia New ischemic change in ECG New pathological Q-wave in ECG Imaging results showing a change in contractility or loss of viable myocardium consistent with ischemic etiology Intracoronary thrombus identified by angiography or necropsy (type 1 only)

**Table 1.2:** Classification of acute myocardial infarction according to triggering factors

Classification (types)	Description
**1**	Spontaneous AMI related to myocardial ischemia secondary to a coronary event, such as rupture or erosion of coronary atherosclerotic plaque
2	AMI secondary to ischemia due to a myocardial oxygen supply/demand imbalance that is not directly related to coronary atherothrombosis
3	Sudden death with symptoms suggestive of ischemia accompanied by new ischemic changes in ECG or ventricular fibrillation before biomarkers are collected, if they are elevated, or in AMI confirmed by necropsy
4a	AMI associated with PCI ≤ 48 h, which is defined as a troponin increase > 5 times the 99th percentile of the upper reference limit or 20% over already elevated baseline levels, associated with one of the following findings: New ischemic change in ECG New pathological Q-wave in ECG Imaging results of a new change in contractility or loss of viable myocardium in a pattern consistent with myocardial ischemia Angiographic findings of complications related to coronary flow limitations (dissection, epicardial vessel occlusion, loss of collateral circulation and distal embolization)
4b	AMI associated with stent thrombosis confirmed by angiography or necropsy
4c	AMI related to intrastent or post-angioplasty restenosis without other lesions or intracoronary thrombi that can explain it
5	AMI associated with myocardial revascularization surgery ≤ 48 h, defined by a > 10-fold increase in the 99th percentile of the upper reference limit or 20% over already elevated baseline levels, associated with one of the following findings: New pathological Q-wave in ECG Imaging results of a new change in contractility or loss of viable myocardium in a pattern consistent with ischemic etiology Angiographic results showing the occlusion of a new graft or native coronary artery

*Source:* adapted from Thygesen K et al.^4^ AMI: acute myocardial infarction; ECG: electrocardiogram; PCI: percutaneous coronary intervention.

**Table 1.3:** Causes of myocardial injury

Myocardial injury with an ischemic etiology
**• Rupture or erosion of atherosclerotic plaque with thrombosis**

**• Myocardial injury related to myocardial ischemia due to oxygen supply/consumption imbalance**
Reduction in myocardial perfusion:
• Coronary artery spasm
• Coronary microvascular disease
• Coronary embolism
• Coronary dissection
• Sustained bradyarrhythmia
• Hypotension or shock
• Respiratory failure
• Severe anemia
Increase in oxygen consumption:
• Sustained tachyarrhythmia
• Hypertensive crisis
**Myocardial injury due to other nonischemic cardiac conditions**
• Heart failureMyocarditis
• Any type of cardiomyopathy
• Takotsubo syndrome
• Myocardial revascularization surgery
• Catheter ablation
• Defibrillation or electrical cardioversion
• Myocardial contusion
• Síndrome de Takotsubo
**Myocardial injury due to systemic conditions**
• Sepsis or active infectious process
• Chronic kidney disease
• Stroke or subarachnoid hemorrhage
• Pulmonary embolism and pulmonary hypertension
• Infiltrative myocardial disease (eg, amyloidosis, sarcoidosis)
• Chemotherapeutic agents
• Critical patient
• Extreme physical activity

Situations in which there is an increase in myocardial necrosis markers but ischemia is not detected in the clinical picture, ECG or imaging tests should be defined as acute myocardial injury, not AMI. They can be secondary to cardiac causes (such as cardiovascular procedures, myocarditis, arrhythmias, or decompensated HF) or extracardiac causes (such as shock, severe anemia, sepsis and hypoxia).^4^

Myocardial injury is often related to a worse prognosis. It is necessary to differentiate between ischemic and nonischemic causes to avoid unnecessary invasive interventions and direct treatment toward other possible etiologies (Table 1.3).

Figure 1.1 summarizes the interpretation of elevated troponin in coronary injury and ischemia scenarios.

**Figure 1.1:** Algorithm for interpreting elevated troponin levels. The troponin “curve” indicates elevation greater than 20%

#### 2.1. Myocardial Infarction with Nonobstructive Coronary Arteries (MINOCA)

Cases of AMI without obstructive coronary artery disease (CAD) are classified as myocardial infarction with nonobstructive coronary arteries (MINOCA). Approximately two-thirds of patients with MINOCA have a clinical presentation of NSTEMI.^5^

According to a document prepared by a European Society of Cardiology working group^6^ and the Fourth Universal Definition of Myocardial Infarction,^4^ the diagnostic criteria for MINOCA are: AMI, angiographic documentation without obstructive CAD (atheromatosis with <50% stenosis or normal coronary arteries) or a clinically evident non-coronary cause for acute presentation.

Different pathophysiological mechanisms can cause MINOCA:

- Dysfunction of epicardial coronary arteries (eg, atherosclerotic plaque rupture, ulceration, cracking, erosion or coronary dissection).
- Imbalance between oxygen supply and consumption (eg, coronary spasm and coronary embolism).
- Coronary endothelial dysfunction (eg, microvascular disease).

MINOCA prognosis varies greatly, depending on the underlying mechanisms and associated risk factors, such as age and female gender. Some studies indicate that MINOCA has lower hospital mortality than AMI with obstructive coronary artery disease and similar 1-year mortality to AMI with univascular obstruction. However, when analyzing patients with NSTEMI in the ACUITY study, the MINOCA subgroup had the highest 1-year mortality (4.7% vs. 3.6%), which was linked to an increase in non-cardiac deaths.^7^^-^^9^

The large group of patients presenting with elevated troponin but no coronary obstruction or clinical signs of infarction is classified as TINOCA (troponin-positive nonobstructive coronary arteries), which includes those with ischemic injury (MINOCA) and the other described etiologies of nonischemic myocardial injury (eg, myocarditis or Takotsubo syndrome; see Figure 1.2).

**Figure 1.2:** Myocardial infarction with nonobstructive coronary arteries (MINOCA) and troponin-positive nonobstructive coronary arteries (TINOCA): conceptual paradigm. Adapted from Pasupathy et al., 2017.^10^

#### 2.2. Unstable Angina

UA is defined as myocardial ischemia without myocardial necrosis, ie, with negative biomarkers. During the initial management of ACS, it is often difficult to differentiate UA from NSTEMI on clinical criteria alone (i.e., before levels of myocardial necrosis biomarkers are available), and both conditions should be treated similarly at this stage. Increased troponin sensitivity decreases the percentage of patients diagnosed with UA and increases the percentage of patients with NSTEMI. The prognosis of patients with UA ranges from relatively low risk to high risk. UA classifications based on clinical presentation and prognostic information facilitate therapeutic management, which will be discussed throughout this document.^11^^-^^16^

### 3. Epidemiology

AMI is the leading cause of death in Brazil and worldwide.^17^ In 2017, according to DATASUS, 7.06% (92,657 patients) of all deaths were caused by AMI. AMI, which accounted for 10.2% of hospitalizations in the Brazilian Unified Health System (SUS), was more prevalent in patients over 50 years of age, accounting for 25% of all hospitalizations).^18^

In the Brazilian Registry of Acute Coronary Syndromes (BRACE), which evaluated ACS hospitalizations in 72 hospitals, NSTE-ACS was responsible for 45.7%, of which about 2/3 involved AMI and 1/3 UA. The study found generally low use of therapies that impact the prognosis of patients with ACS, including important regional differences. It also developed a performance score, which showed that the greater the adherence to proven treatments, the lower the mortality.^19^

### 4. Pathophysiology

The main pathophysiological characteristic of ACS is the instability of atherosclerotic plaque, involving erosion or rupture and subsequent formation of an occlusive or subocclusive thrombus. Such flow limitations, however, can be due to other mechanisms, such as vasospasm, embolism, or coronary dissection. Other factors may be involved in the pathophysiology of ACS by altering the supply and/or consumption of myocardial oxygen, such as anemia, hypertension, tachycardia, hypertrophic cardiomyopathy, aortic stenosis, etc..^20^

In animals, ischemia has been observed to progress from the subendocardium to the subepicardium. This progression can be delayed when there is efficient collateral irrigation, reduced oxygen consumption in the myocardium, and intermittent flow (generating ischemic preconditioning). Changes in ventricular function due to the progression of myocardial ischemia initially involve diastolic dysfunction, which may be followed or not by systolic dysfunction. A shorter duration of ischemic injury is associated with a smaller area of myocardial necrosis. Several studies have also suggested that restoring perfusion results in some degree of myocardial injury (reperfusion injury), especially in situations of total coronary occlusion.^21^

### 5. Initial Assessment

#### 5.1. Initial Approach

Patients with suspected ACS and signs of severity (persistent pain, dyspnea, and palpitations due to potentially severe arrhythmias and syncope) should be referred to emergency departments, ideally monitored while in the ambulance. Patients without signs of severity (listed above) should be instructed to seek out an emergency department that can perform ECG and cardiac biomarker measurement, preferably troponin.

Several tools (scores) have been devised for this purpose and, in conjunction with clinical judgment, can help define which patients could benefit from hospitalization, complementary exams, and specific treatment.

#### 5.2. Diagnosis and Prognostic Assessment

##### 5.2.1. History and Clinical Data

###### 5.2.1.1. Characterization of Chest Pain and Angina

The clinical picture of angina can be defined by four main pain characteristics: location, type, duration, and intensification or relief factors.

- Location: usually in the chest, close to the sternum. However, it can affect or radiate from the epigastrium to the mandible, the interscapular region and the arms (more commonly to the left, less commonly to both arms or the right arm).
- Type: the discomfort is usually described as pressure, tightness or weight. Sometimes it involves a feeling of strangulation, compression or burning. It may be accompanied by dyspnea, sweating, nausea, or syncope.
- Duration: the duration of stable anginal pain is generally short (<10 min); episodes of ≥10 min suggest ACS. However, prolonged continuous pain (hours or days) or ephemeral pain (few seconds) is less likely to indicate ACS.
- Intensification or relief factors: one important feature of angina is its relationship with physical exertion. Symptoms classically appear or intensify on exertion. In patients with no history of angina, a reduction in the effort threshold required to trigger angina suggests ACS. ACS discomfort does not usually vary with changes in breathing or position.

The clinical history of patients with NSTE-ACS plays an important role in risk stratification. In the presence of angina, patients with NSTE-ACS can present in four ways:

- Prolonged resting angina (> 20 min).
- Recent onset angina (class II or III according to Canadian Cardiovascular Society classification): in general, these patients manifest typical symptoms of angina in less than 2 months that begin appearing after minimal exertion.
- Recent worsening of previously stable angina (crescendo angina). Minimal exertion triggers angina, increased pain intensity and/or duration, changes in pain radiation patterns, changes in response to the use of nitrates.
- Post-infarction angina.

Some characteristics, antecedents and comorbidities are related to a higher probability of ACS:

- Advanced age and male sex.
- Risk factors for atherosclerosis: smoking, diabetes mellitus (DM), dyslipidemia, arterial hypertension and chronic renal failure.
- Family history of CAD.
- Previous symptomatic atherosclerosis, such as peripheral arterial obstructive disease, carotid disease, previous coronary disease.
- Chronic inflammatory diseases, such as lupus or rheumatoid arthritis.

ACS patients may present with atypical symptoms, such as isolated epigastric pain, feelings of gastric fullness, lancinating pain, pleuritic pain or dyspnea. Although ACS typically presents in women and older adults (> 75 years) as angina, atypical presentations are also higher in these groups, as well as in patients with DM, renal failure and dementia.

The Coronary Artery Surgery Study proposed a chest pain classification system,^22^ which is shown in Table 1.4.

**Table 1.4:** Coronary Artery Surgery Study angina classification system

Definitely anginal pain	Retrosternal pain due to exertion, radiating to the shoulder, neck or left arm and attenuated by rest or nitrates in <10 min
Probably anginal pain	Most of the characteristics of definite angina pain
Probably not anginal pain	Atypical pain that does not meet the criteria for anginal pain
Definitely not anginal pain	Pain uncorrelated with physical activity (suggestive of non-cardiac origin) that is unmitigated by nitrates

Source: Adapted from the National Heart Lung and Blood Institute's Coronary Artery Surgery Study.^22^

###### 5.2.1.2. NSTE-ACS in Older Adults

Older adults with ACS generally have a different risk profile from younger adults: they have a higher prevalence of hypertension, DM, previous AMI, angina, peripheral vascular disease, stroke, multivessel disease, and HF. Older adults generally wait longer to seek medical care after symptom onset. In NSTE-ACS, instead of pain, patients often have so-called “ischemic-equivalent” symptoms, such as dyspnea, malaise, mental confusion, syncope or pulmonary edema. Older adults have a higher incidence of complications in NSTE-ACS, which implies the need for more intensive treatment. However, especially in adults over 75 years of age, the most appropriate therapy, ie, beta-blockers, acetylsalicylic acid (ASA), anticoagulants and lipid-lowering agents, is not used. Of the 3,318 patients with NSTE-ACS included in the TIMI III study,^23^ 828 were over 75 years old. These individuals received anti-ischemic therapy and underwent cardiac catheterization at a lower percentage than younger patients. Although they had more severe and extensive CAD, they less frequently underwent myocardial revascularization procedures and had more adverse events within 6 weeks of onset. According to a national database study, the use of proven efficacious therapies after ACS has increased in the last 15 years, both among the oldest old (age > 80 years) and in younger adults (< 50 years), and this increase has been associated with improved survival in both groups.^24^

###### 5.2.1.3. History

####### 5.2.1.3.1. Patients Undergoing Myocardial Revascularization Procedures: Percutaneous Coronary Intervention and/or Myocardial Revascularization Surgery

The recurrence of angina after coronary artery bypass graft (CABG) surgery or percutaneous coronary intervention (PCI) can indicate acute complications, new injuries, stent thrombosis, or restenosis. Chest pain up to 48 hours after percutaneous intervention suggests acute obstruction, transient coronary spasm, non-occlusive thrombus, branch occlusion or distal embolization. Recurrent chest pain approximately 6 months after conventional stenting or later, after drug-eluting stenting, is most likely related to restenosis. On the other hand, angina onset 1 year after stenting is usually related to a new coronary lesion or stent restenosis due to neoaterosclerosis. After a CABG, the early appearance of pain is usually associated with thrombotic obstruction of the graft. In the first 12 months after CABG, the mechanism is usually fibrous intima hyperplasia; after this period, pain is indicative of a new atherosclerotic lesion and/or non-thrombic graft degeneration. The TIMI III registry compared the incidence of death or nonfatal infarction among NSTE-ACS patients with or without previous CABG. Those with previous CABG had higher rates of complications up to 10 days after admission (4.5% vs. 2.8% with or without CABG, respectively) and after 42 days (7.7% vs. 5.1%, respectively), which suggests that this is a higher risk group, mainly due to more extensive atherosclerosis.^25^

####### 5.2.1.3.2. Risk Factors for Coronary Artery Disease

Some studies have suggested that smokers have a better prognosis, probably due to the fact that they suffer ACS at an earlier age and have less atherosclerotic burden than non-smokers.^26^^,^^27^ On the other hand, Antman et al. showed that having three or more risk factors for CAD (systemic arterial hypertension, DM, dyslipidemia, family history and smoking) is an independent marker of a worse prognosis.^28^

##### 5.2.2. Physical examination

During assessment of individuals with NSTE-ACS, a physical examination can help identify individuals at higher risk (those with signs of severe ventricular dysfunction or mechanical complications) and help in the differential diagnosis of chest pain unrelated to ACS.

As a rule, a normal or slightly modified physical examination is insufficient to stratify patient risk, since even patients with multivessel or left main coronary lesions can appear normal upon physical examination.^29^^-^^31^ However, when changes are found in a physical examination, they can be important for categorizing patients as high risk.

Some findings that indicate a poor prognosis include systolic murmur in the mitral focus tachycardia, tachypnea, hypotension, sweating, weak pulse, third heart sound, and pulmonary rales.

Changes in physical examination results allow differential diagnosis between ACS and other causes of chest pain:

**• Cardiac**: pericarditis (pericardial friction), cardiac tamponade (paradoxical pulse), aortic stenosis (aortic systolic murmur), and hypertrophic cardiomyopathy (ejection systolic murmur in the parasternal area that increases with the Valsalva maneuver).
**• Non-cardiac**: aortic dissection (pulse and pressure divergence between the arms and a diastolic murmur of aortic insufficiency), pulmonary embolism/pulmonary infarction (pleural friction), pneumothorax (decreased breath sounds and hyperresonance to percussion), and musculoskeletal symptoms (pain on palpation).

**Table:** 

History and physical examination - Summary of recommendations and evidence		
Patients with suspected NSTE-ACS and persistent pain, dyspnea, palpitations or syncope should be referred to emergency departments, ideally monitored in the ambulance.	I	C
Patients suspected of NSTE-ACS with less severe findings (i.e., without persistent pain, dyspnea, palpitations or syncope) should be instructed to seek out an emergency department that can perform ECG and troponin measurement.	IIb	C

##### 5.2.3. Electrocardiogram

Twelve-lead ECG is the initial diagnostic tool for patients with suspected ACS. Ideally, it should be performed and interpreted during pre-hospital care or within 10 minutes of hospital admission.

About 1% to 6% of patients with NSTE-ACS have normal (non-diagnostic) ECG results at admission. The ECG should be repeated after 15 to 30 minutes, especially in individuals who are still symptomatic. Normal or non-diagnostic ECG results can occur even with left circumflex or right coronary artery occlusion. Thus, additional V3R, V4R, V7, V8, and V9 derivations are recommended to increase the method's sensitivity.

More than 1/3 of patients will have typical ACS changes, such as ST-segment depression, transient ST-segment elevation, and T-wave inversion. Dynamic changes in the ST-segment (depression or elevation) or T-wave inversions during a painful episode that resolve at least partially when the symptoms are relieved are important markers of adverse prognosis, ie, subsequent AMI or death.^32^ Patients with ST changes in anterior leads often have significant stenosis of the anterior descending coronary artery and are a high-risk group.

ST-segment and T-wave changes are not specific to NSTE-ACS and can occur in several conditions, including: ventricular hypertrophy, pericarditis, myocarditis, early repolarization, electrolyte alteration, shock, metabolic dysfunction, and digitalis effect.

The diagnostic accuracy of an abnormal ECG is increased when previous ECG results are available for comparison.

Transient ST-segment elevation is consistent with Prinzmetal or vasospastic angina. Concomitant ST elevation in the anterior and inferior leads (reflecting extensive ischemia) is associated with an increased risk of sudden death.^33^

###### 5.2.3.1. Electrocardiogram Findings and Prognosis

Patients with NSTE-ACS are at high risk of ischemic ECG changes (elevated or depressed ST-segment), atrial fibrillation, and ventricular arrhythmias. These changes imply a worse prognosis.^31^ In the GUSTO II trial, certain baseline ECG factors in patients with ACS were prognostic of early mortality:^34^ left bundle branch block, left ventricular (LV) hypertrophy or pacemaker rhythm (11.6%); ST-segment depression (8%); ST-segment elevation (7.4%); and normal T-wave inversion or normal ECG (1.2%).

Moreover, in 1,416 patients with ACS, the TIMI III Registry ECG Ancillary Study^35^ found the following ECG presentations: ST-segment deviation > 1 mm (14.3%), left bundle branch block (19%), isolated T-wave inversion (21.9%), or none of these changes (54.9%).

**Table:** 

Electrocardiogram - Summary of recommendations and evidence		
All patients with suspected NSTE-ACS should undergo ECG. Ideally, an ECG should be performed within 10 minutes of the patient's arrival at the hospital.	I	B
The ECG should be repeated at least once within 6 hours in non-diagnostic cases.	I	C
The ECG should be repeated in case of symptom recurrence.	I	C
The V3R-V4R, and V7-V9 leads should be recorded in patients who remain symptomatic and have a non-diagnostic 12-lead ECG.	I	C

##### 5.2.4. Biomarkers

In patients with ACS, biomarkers are useful for both diagnosis and prognosis. When myocardial cells are injured, their cell membranes lose integrity, intracellular proteins diffuse across the interstitium and into the lymphatic system and capillaries. After a myocardial injury, marker kinetics depend on several factors: intracellular protein degradation, molecule size, regional lymphatic and blood flow, as well as marker clearance rate. Such factors, together with the characteristics of each marker, differentiate each patient's diagnostic AMI performance.^36^

In patients with symptoms suggestive of ACS but without diagnosed AMI, cardiac biomarkers are useful to confirm the diagnosis. They also provide important prognostic information, since there is a direct association between elevated serum markers and the risk of cardiac events in the short and medium- term.^37^ The necrosis marker results must be available within 60 minutes of collection. If the clinical analysis laboratory cannot provide this, point-of-care technologies should be considered.^38^

###### 5.2.4.1. Troponins

Troponins are a complex of myofibrillar regulatory proteins in striated cardiac muscle that include troponin T, troponin I, and troponin C. Troponin C is coexpressed in slow-twitch skeletal muscle fibers and is not considered as a specific cardiac marker. In recent decades, immunoassay techniques with specific monoclonal antibodies for cardiac troponin T and cardiac troponin I have been developed. Meta-analyses have shown that the sensitivity and specificity of cardiac troponin I for diagnosing AMI was approximately 90% and 97%, respectively. Cardiac troponins remain elevated for a longer time, up to 7 days after AMI. Troponins are the biomarkers of choice for diagnostic evaluation in patients with suspected AMI since their diagnostic accuracy is higher than that of creatine kinase MB (CK-MB) mass and other biomarkers of myocardial injury. Patients with elevated troponins are at increased risk of cardiac events during the first days of hospitalization and are the NSTE-ACS subgroup that can most benefit from an invasive strategy.^39^ Although troponins can accurately identify myocardial injury, they cannot identify the mechanism of injury(ies). The mechanisms can include non-coronary etiologies, such as tachyarrhythmias and myocarditis, or non-cardiac conditions, such as sepsis, pulmonary embolism, and renal failure.^40^ Thus, in cases where the clinical presentation is not typical of ACS, other causes of cardiac injury related to increased troponins should be considered.

Troponins have recognized value when evaluating patients with ischemic ECG changes or with clinical symptoms suggestive of anginal pain. The major limitation of conventional troponins is their low sensitivity in patients whose time to onset is less than 6 hours. With the introduction of highly sensitive troponin assays, it became possible to detect lower troponin levels in a shorter time after ischemic myocardial injury.^41^^,^^42^ The unit used to express the values of conventional troponin is ng/mL, and in highly sensitive troponins, the values can be expressed in ng/L, since their detection power is 10 to 100 times greater than conventional troponins.

Highly sensitive troponins are significantly more sensitive than conventional troponins in ACS diagnosis, improving the diagnostic power for AMI by 61% if collected less than 3 hours after onset and 100% if collected 6 hours after onset.^43^ Due to the highly sensitive troponin assay's increased sensitivity and diagnostic accuracy for AMI, accelerated diagnosis algorithms have been proposed. Thus, the time to diagnosis can be shortened, which means less time in the emergency department and lower costs.^44^^-^^46^ It is recommended that the investigation algorithm be used within 3 hours (see routine diagnostic flowchart and hospitalization criteria).

###### 5.2.4.2. Creatine Kinase: Isozymes and Isoforms

Before troponins emerged as more accurate biomarkers in AMI diagnosis, CK-MB was the most common biomarker in chest pain protocols. Ideally, CK-MB should be measured by immunoassay for plasma concentration (CK-MB mass) rather than activity. This change in measurement patterns is due, in part, to studies that demonstrated that CK-MB mass has greater sensitivity and specificity for AMI.^47^ CK-MB subforms have emerged as early markers (< 6 hours) of myocardial injury and early inference of AMI severity, according to data from a necropsy study that demonstrated a better correlation between CK-MB mass and AMI size.^48^ However, the main limitation of CK-MB mass is damage to other non-cardiac tissues (false positives), especially after injuries to smooth or skeletal muscle. In about 4% of patients, false-positive results can occur, with positive CK-MB and negative troponin.^49^ In cases where CK-MB is elevated and troponin is normal, both within their kinetic windows, clinical decisions should be based on the troponin results.

**Table:** 

Biochemical markers - Summary of recommendations and evidence		
Troponins are the biomarkers of choice for diagnosing patients with suspected AMI.	I	A
When highly sensitive troponin assays are available, no other marker should be requested for routine AMI diagnosis.	I	B
Biochemical biomarkers of myocardial necrosis should be measured in all patients with suspected NSTE-ACS. When highly sensitive troponin assays are available, serum levels should be measured on admission and, ideally, reassessed in 1 h or up to 2 h. If unavailable, conventional troponin measurement should be performed at admission and repeated at least once, 3 to 6 hours later, if the initial results are normal or slightly elevated.	I	B
CK-MB mass levels can be used if troponin assays are unavailable.	IIb	B
Myoglobin can be used to detect myocardial necrosis in patients with suspected NSTE-ACS.	II	

##### 5.2.5. Non-Invasive Imaging in the Emergency Department

###### 5.2.5.1. Functional Assessment

Non-invasive exams play an essential role in the diagnosis (especially in patients with normal ECG and biomarker results) and risk stratification of patients with suspected ACS. The choice for each patient – whether ET, myocardial perfusion scintigraphy, cardiac resonance, or coronary computed tomography angiography – will depend on the objectives and clinical questions to be answered.^50^

###### 5.2.5.2. Exercise Testing in the Emergency Department

In the emergency department, patients whose chest pain is identified as low or intermediate risk can undergo ET. Normal results indicate a low risk of cardiovascular events, allowing for earlier and safer hospital discharge.^50^ National and international guidelines recommend ET as the first choice for risk stratification in patients who can exercise since it is a low-cost procedure, is widely available, and has a low frequency of complications.^51^ However, patients with moderate- to high-risk ACS, acute aortic diseases, pulmonary thromboembolism, myocarditis, or pericarditis must be ruled out since these conditions are absolute contraindications. The testing protocol should be determined according to the patient's clinical condition, with the most recommended being a modified Naughton or Bruce ramp test.

ET indications in ACS (to characterize low risk after initial clinical stratification) include:

- Baseline ECG and necrosis biomarkers without changes.
- Absence of symptoms (chest pain or dyspnea).
- Hemodynamic stability and adequate conditions for physical exertion.

If the ET results are normal and the patient has shown adequate functional capacity, other procedures may not be necessary due to the test's high negative predictive value.^51^

**Table:** 

Exercise testing - Summary of recommendations and evidence		
Low-risk patients (according to clinical and ECG results) with normal biomarkers should be referred for ET after 9 to 12 hours of observation. In chest pain unit routines, these exams can be performed as a discharge criterion.	I	B
If an ET cannot be performed or the ECG is uninterpretable (left bundle branch block, artificial pacemaker, atrial fibrillation, LV overload, etc.), the patient can undergo ischemia tests associated with non-invasive imaging.	I	B

###### 5.2.5.3. Echocardiography

Echocardiography is a useful complementary method for evaluating chest pain in emergencies.^52^^-^^54^ This non-invasive exam provides quick diagnostic information.^55^^-^^59^ When performed during an episode of chest pain, a lack of abnormal ventricular segmental contraction indicates a nonischemic cause. Although echocardiography cannot determine whether the segmental change is recent or pre-existing, the presence of segmental contraction abnormalities reinforces the probability of CAD being indicative of infarction, ischemia, or both. However, it can also appear in cases of myocarditis.^60^^-^^62^

Other no less important etiologies of chest pain (e.g., aortic dissection, aortic stenosis, hypertrophic cardiomyopathy, and pericardial disease) can also be assessed with echocardiography. Significant coronary heart disease is commonly found in patients with UA. These patients are generally identified by clinical history, and reversible ECG changes can be detected with pain episodes. When the patient's history and ECG are atypical, documenting a segmental contraction abnormality with echocardiography during or immediately after a pain episode strongly suggests the diagnosis.^63^ Echocardiography also determines the presence and extent of ventricular dysfunction and the presence and severity of valve abnormalities.

Other parameters are also important in determining prognosis in addition to the LV ejection fraction (LVEF) and segmental motility. Ersbǿll et al. prospectively studied patients with infarction and LVEF > 40% within 48 hours of admission. All patients underwent ECG with semi-automated global longitudinal strain (GLS) assessment. Of the 849 patients, 57 (6.7%) had severe cardiac events, and GLS > −14% was associated with a 3-fold increase in the risk of severe events.^63^

###### 5.2.5.4. Stress Echocardiography

Stress echocardiography is being increasingly used in emergency departments and soon after hospitalization.^64^ One study analyzed 108 patients who were observed for 4 hours with serial biomarkers and ECG and underwent stress ET or dobutamine stress ECG. Ten patients had positive stress ET results, and 8 had positive stress ECG results. The exams were consistent in 4 patients. All patients whose stress ECG results showed no evidence of ischemia were cardiac event-free at the end of 12 months of follow-up, as were 97% of the patients with negative stress ET results.^65^

A Brazilian study evaluated 95 patients with low- and moderate-risk UA with echocardiography under dobutamine stress, most of whom were assessed in the first 72 hours of hospitalization. For the clinical outcomes (death, AMI, new hospitalization due to UA and myocardial revascularization procedures), the exam proved to be safe and had an excellent negative predictive value (96%), allowing early discharge without the need for other tests.^66^ Using perfusion techniques to perform stress echocardiography can increase diagnostic sensitivity; their applications are discussed in Part 2 of this Guideline.

Because it is an accessible, fast, low-cost, and non-invasive test, echocardiography can provide additional prognostic information about the previously mentioned parameters by assessing global and regional ventricular function and identifying associated valvulopathy. It can be routinely used in the investigation of these patients. Its disadvantages are its limited acoustic window in some patients and reduced sensitivity to dobutamine stress in patients on beta-blockers.

**Table:** 

Echocardiography - Summary of recommendations and evidence		
Transthoracic echocardiography must be performed for a differential diagnosis when there is clinical suspicion of aortic disease, pericardial disease, pulmonary embolism, or heart valve disease.	I	C
Emergency echocardiography should be performed when there are complications resulting from NSTE-ACS, such as interventricular communication and mitral regurgitation.	I	C
Stress echocardiography can be used for functional stratification in patients without recurrent chest pain or ECG evidence of ischemia and/or troponin elevation.	I	B
In patients with chest pain, resting echocardiography can identify changes compatible with ischemic etiology.	IIa	C

###### 5.2.5.5. Nuclear Cardiology

Several studies have suggested that low-risk results for resting myocardial scintigraphy performed in the emergency department indicate a very low risk of subsequent cardiac events. On the other hand, there is a much greater probability that patients with high-risk myocardial perfusion scintigraphy results will develop AMI, be revascularized (surgery or angioplasty), or have coronary lesions a coronary angiogram.^67^^-^^69^ The ERASE trial evaluated care strategies for ACS patients with normal or non-diagnostic ECG, finding admission rates of 54% for patients who underwent MPS and 63% for all others, which suggests that an initial strategy involving resting scintigraphy is an acceptable risk stratifier.^70^

International guidelines recommend rest myocardial perfusion imaging during acute chest pain to stratify risk in patients with suspected ACS who have non-diagnostic ECG results.^71^^-^^73^

####### Radiotracer injection:

The main applications of MPS in the first hours after the patient's hospital admission include:

- Injection of the radiotracer (sestamibi/MIBI or technetium-99m-labeled tetrofosmin, or 99mTc-sestamibi, and 99mTc-tetrofosmin) at rest during a chest pain episode in patients with normal or nonspecific ECG, aiming at a rapid, definitive diagnosis.
- Injection of the radiotracer at rest in patients without chest pain, normal or nonspecific ECG, and symptom cessation less than 6 hours, but preferably within the previous 2 hours. Wackers et al. found that when the injection was performed in the first 6 hours of pain in ACS patients, the incidence of perfusion abnormalities was 84%, which decreased to 19% when the radiotracer was intravenously administered between 12 and 18 hours after the last pain episode.^74^

Overall, radiotracers can be used to obtain resting perfusion images for acute chest pain assessment without formal contraindications, and most patients well tolerate them. MPS with physical or pharmacological stress in low- or intermediate-risk ACS patients is recommended after stabilizing the acute condition and is usually performed during hospitalization. Stable clinical and hemodynamic conditions are paramount in both situations. The limitations of this method are its availability and cost.

**Table:** 

Nuclear cardiology - Summary of recommendations and evidence		
MPS at rest during acute chest pain to stratify risk in patients with suspected ACS who have non-diagnostic ECG results	I	A
MPS can be used for functional stratification in patients without recurrent chest pain, without ECG evidence of ischemia and/or elevated troponin levels	I	B
Patients with chest pain and no ECG evidence of ischemic changes can be assessed with MPS at rest to determine whether the pain has an ischemic origin	IIa	A

###### 5.2.5.6. Anatomical Assessment: Coronary Computed Tomography Angiography

In recent years, coronary computed tomography angiography (CCTA) has been increasingly used to assess patients with suspected obstructive coronary disease. It has been demonstrated that the method is more accurate in diagnosing luminal stenosis than invasive coronary angiography, especially its high negative predictive value.^75^^-^^77^ Several studies in different clinical situations have also shown the prognostic value of CCTA regarding the presence and extent of obstructive and non-obstructive CAD, which can help with decision-making.^78^^,^^79^

Three large multicenter randomized controlled trials have evaluated CCTA for chest pain in emergency departments, including a total of more than 3,000 patients. The multicenter CT-STAT study randomized 699 patients with low-risk chest pain to stratification with CCTA or rest-stress myocardial scintigraphy.^80^ The CCTA strategy reduced diagnosis time by 54% and hospitalization costs by 38%, with no difference in the rate of adverse events between the two methods. The ACRIN-PA study primarily aimed at assessing the safety of CCTA for evaluating patients with low- and intermediate-risk chest pain compared to the traditional approach.^81^ None of the patients with negative CCTA had the primary outcome (cardiac death or infarction) in the first 30 days after admission. Also, patients in the CCTA group had a higher rate of discharge from emergency departments (49.6% vs. 22.7%) and fewer days of hospitalization (18 hours vs. 24.8 hours, p < 0.001), with no significant differences in the incidence of coronary angiography or revascularization in the first 30 days after admission. Finally, the ROMICAT II study evaluated the emergency department length of stay and hospital costs in similar groups of patients.^82^ It included 1000 patients with an average age of 54 years (46% female). The length of hospital stay was significantly shorter in patients stratified by CCTA than the traditionally-assessed group (23.2 ± 37 hours vs. 30.8 ± 28 hours; p = 0.0002). The time until an ACS diagnosis was also shorter in the CCTA group (17.2 ± 24.6 hours vs. 27.2 ± 19.5 hours; p < 0.0001). There were no significant safety differences between the groups. In the CCTA group, there was a substantial increase in patients discharged directly from the emergency department (46.7% vs. 12.4%; p = 0.001), but significantly more diagnostic tests were used in this group (97% vs. 82%, p < 0.001). Despite the higher cost associated with CCTA and a tendency toward more catheterizations and revascularizations, the overall costs were similar between the two groups (p = 0.65).

A subsequently published meta-analysis confirmed that using CCTA to assess patients with acute chest pain is associated with reduced cost and length of hospital stay, with an apparent increase in invasive angiographies and myocardial revascularizations compared to traditional approaches.^83^

####### 5.2.5.6.1. Triple Rule-out

CCTA can be used in the emergency department to visualize the coronary arteries and obtain information about the aorta and pulmonary arteries, allowing assessment for acute aortic syndromes, pulmonary thromboembolism, or other thoracic changes that may be differential diagnoses of ACS (pneumonia and trauma).^84^^,^^85^ Through specific acquisition protocols, all of this information can be obtained in a single exam. This approach is called the triple rule-out, but it should only be used in specific situations where clinical evaluation cannot direct the diagnosis.^86^

In summary, CCTA is a safe and efficient strategy for evaluating patients with acute low- and intermediate-risk chest pain in emergency departments, reducing the time until correct diagnosis and length of hospital stay.^87^ The method's disadvantages include the use of ionizing radiation, its high cost, the need for iodinated contrast media, its limited availability in Brazil, and the fact that it cannot be used in patients with heart rates above 80 beats per minute or who cannot use beta-blockers.

**Table:** 

Coronary computed tomography angiography - Summary of recommendations and evidence		
In patients with acute chest pain and a low to intermediate probability of CAD, non-diagnostic ECG, and negative markers of myocardial necrosis.	I	A
Acute chest pain can be investigated with the triple rule-out technique.	IIb	B

#### 5.3. Risk Stratification

##### 5.3.1. Risk Stratification of Ischemic Cardiovascular Events

Using the TIMI IIB database, Antman et al. found the following independent markers of worse prognosis in patients with NSTE-ACS (“TIMI group risk score”): age ≥65 years, elevated biochemical markers, ST-segment depression ≥0.5 mm, ASA use in the 7 days prior to symptom onset, three or more traditional risk factors for CAD (hypertension, hypercholesterolemia, DM, smoking, or family history), known CAD, and recent severe angina (< 24 hours).^28^ With one point for each item, a score of 0 to 2 is classified as low risk, 3 to 4 as intermediate risk, and 5 to 7 as high risk. This risk score has been validated in other NSTE-ACS studies. An association has been found between each marker and a higher incidence of events (death, reinfarction, and recurrent ischemia requiring revascularization) and higher risk scores. (Figure 1.3).

**Figure 1.3:** Clinical risk stratification scores for chest pain.

Due to its good discriminatory power, the Global Registry of Acute Coronary Events (GRACE) risk score allows more accurate stratification at both hospital admission and discharge (Figure 1.4). However, it is complex, requiring a personal computer or digital device to calculate the risk.^88^ The original GRACE score provides an estimate of in-hospital death or AMI and death 6 months after discharge. The score was later validated for risk estimation at 1 and 3 years. Eight prognostic variables of hospital mortality have been identified for this score, and the total score for a given patient is the sum of each variable:

1. Age in years - ranging from 0 (< 30) to 100 points (> 90).
2. Heart rate (bpm) - ranging from 0 (< 50) to 46 points (> 200).
3. Systolic blood pressure (mmHg) - ranging from 0 (> 200) to 58 points (< 80).
4. Creatinine levels (mg/dL) - ranging from 1 (< 0.40) to 28 points (> 4.0).
5. Heart failure (Killip class) - ranging from 0 (class I) to 59 points (class IV).
6. Cardiac arrest at admission - ranging from 0 (no) to 39 points (yes).
7. ST-segment deviation - ranging from 0 (no) to 28 points (yes).
8. Increased levels of cardiac injury biomarkers - ranging from 0 points (no) to 14 points (yes).

**Figure 1.4:** GRACE risk score.

Source: adapted from Granger et al.^89^ BP: blood pressure; ICC: intracluster correlation coefficient; PCR: polymerase chain reaction

The incidence of hospital death is ≤ 1% for low-risk patients (total scores ≤ 108), between 1% and 3% for intermediate-risk scores (between 109 and 140), and over 3% for high-risk patients (> 140).^89^ A new version of the GRACE score (GRACE 2.0), developed using the same predictor variables for treatment outcome, has expanded the risk estimates for in-hospital death to 6 months, 1 year, and 3 years risk of death or AMI to 1 year.^90^ Table 1.5 shows the risk stratification based on clinical, ECG, and laboratory variables.

**Table 1.5:** Risk stratification for death or infarction in patients with acute ischemic syndrome without ST-segment elevation^91^

	High	Moderate	Low
Prognostic Variable	At least one of the following characteristics must be present	No high-risk features, but any of the following	No intermediate or high risk characteristics, but any of the following
History	Symptom worsening in the last 48 hours. Age > 75 years	Age = 70 to 75 years,previous infarction, cerebrovascular or peripheral disease, DM, revascularization surgery, previous ASA therapy	
Precordial pain	Prolonged pain (> 20 min), at rest.	Resting angina > 20 min resolved, with a moderate-to-high probability of CAD Angina at rest ≤20 min, with spontaneous relief or through nitrates	New episode of CCS class III or IV angina in the last 2 weeks without prolonged pain at rest, but with moderate or high probability of CAD
Physical exam	Pulmonary edema, worsening or appearance of mitral murmur or regurgitation, S_3_, new rales, hypotension, bradycardia or tachycardia		
Electrocardiogram	ST-segment depression > 0.5 mm (associated or not with angina), dynamic ST change, new or presumably new complete branch block, sustained ventricular tachycardia	T-wave inversion > 2 mm; pathological Q waves.	Normal or unchanged during the pain episode
Serum markers of ischemia*	Markedly high	High	Normal

* High cardiac troponin I, cardiac troponin T, or creatine kinase-MB (preferentially mass): above the 99th percentile; slight elevation: above the detection level and below the 99th percentile. ASA: acetylsalicylic acid; CAD: coronary artery disease; CCS: Canadian Cardiovascular Society.

The HEART score (discussed in item 5.4) assesses the risk of a major cardiac event (infarction, revascularization, or death) 6 weeks after initial presentation in patients with chest pain.

##### 5.3.2. Bleeding Risk Stratification

Bleeding is associated with an adverse prognosis in NSTE-ACS, and every effort should be made to reduce it. Certain variables may help classify patients at different risk levels for major bleeding during hospitalization. Bleeding risk scores have been developed based on cohort studiesand ACS clinical studies. The CRUSADE score (www.crusadebleedingscocre.org) was developed from a cohort of 71,277 patients and subsequently validated in a cohort of 17,857 patients^92^ (Table 1.6). In a Brazilian population, it predicted not only bleeding but in-hospital mortality (area under the ROC curve = 0.753, p < 0.001).^93^ The rate of major bleeding gradually increased as the bleeding risk score increased. By incorporating admission and treatment variables, this score's accuracy for estimating bleeding risk is relatively high. Although age is not listed among the prognostic factors in this score, it is included in the creatinine clearance calculations.

**Table 1.6:** CRUSADE bleeding risk score (reference b) | Algorithm used to determine the CRUSADE risk score for major in-hospital bleeding

Prognostic factor	Score
**Baseline hematocrit (%)**	
< 31	9
31-33.9	7
34-36.9	3
37-39.9	2
> 40	0
**Creatinine clearance (mL/min)**	
< 15	39
16-30	35
31-60	28
61-90	17
91-120	7
> 120	0
**Heart rate (bpm)**	
< 70	0
71-80	1
81-90	3
91-100	6
101-110	8
111-120	10
> 120	11
**Sex**	
Male	0
Female	8
**Heart failure signs at presentation**	
No	0
Yes	7
**Previous vascular disease**	
No	0
Yes	6
**Diabetes mellitus**	
No	0
Yes	6
**Systolic blood pressure (mmHg)**	
< 90	10
91-100	8
101-120	5
121-180	1
181-200	3
> 200	5

Another score was derived from the ACUITY and HORIZONS studies. Six independent variables (female sex, advanced age, elevated serum creatinine, elevated white blood cell count, anemia, ACS with or without ST elevation) and one treatment-related variable (heparin and GP IIb/IIIa inhibitor use instead of bivalirudin alone) were identified (Table 1.7). This score identified patients at increased risk of bleeding (unrelated to CABG) and mortality after 1 year.^94^

**Table 1.7:** Bleeding risk score proposed by Mehran et al.^94^ Algorithm used to determine the risk of major in-hospital bleeding

Sum		
Sex	Male, Female 0, +8	
Age (years)	<50, 50-69, 60-69, 70-79, ≥80 0, +3, +6, +9, +12	
Serum creatinine (mg/dL)	<1.0, 1.0, 1.2, 1.4, 1.6, 1.8, ≥2.0 0, +2, +3, +5, +6, +8, +10	
Total leukocytes (10^6^/mL)	<10, 10, 12, 14, 16, 18, ≥20 0, +2, +3, +5, +6, +8, +10	
Anemia	No, Yes 0, +6	
ACS Presentation	STEMI, NSTEMI, unstable angina +6, +2, 0	
Antithrombotic medications	Heparin + GP IIb/IIIa inhibitor, bivalirudin 0, −5	
	Total score	

NSTEMI: non-ST-segment elevation myocardial infarction; STEMI: ST-segment elevation myocardial infarction

Both scores were derived from cohorts in which femoral access was predominantly or exclusively used. Their prognostic value may be reduced regarding radial access.^95^

Finally, the authors point out that no score (either for ischemic events or for hemorrhagic events) should replace clinical evaluation. Rather, they are merely tools to help doctors in their decision-making process.

**Table:** 

Risk stratification - Summary of recommendations and evidence		
All patients should be stratified as having a high, intermediate or low risk of major cardiac events. Classification by more than one method is recommended, and the worst case scenario must be considered in treatment decisions.	I	B
All patients must be stratified as high, intermediate or low risk of bleeding.	I	B

#### 5.4. Emergency Diagnostic Flowchart and Hospitalization Criteria

It is essential to develop a diagnosis flowchart for patients with chest pain, determining the criteria for early discharge and hospitalization. This can identify low-risk patients to be investigated in an outpatient setting, as well as more serious cardiac conditions that require hospitalization.

Emergency department chest pain screening is based on a brief clinical history, physical examination, 12-lead ECG within 10 minutes of arrival, and biomarker measurement. This assessment protocol is mainly aimed at early identification of high-risk patients who require hospitalization or urgent transfer for hemodynamic monitoring.

To avoid premature discharge, these patients should remain monitored in an environment where an accelerated diagnostic protocol can be used. This protocol involves serial ECG and highly sensitive troponin measurement upon arrival at the emergency department and 3 hours later. In addition to this protocol, risk scores that include demographic data, symptoms, ECG findings and biomarkers are important tools for assessing chest pain patients in the emergency department. The most commonly-used scores (HEART, ADAPT, and EDACS)^96^^-^^98^ are summarized in Figure 1.3.

Greenslade et al. compared the effectiveness of these scores in relation to premature discharge from the emergency department, finding that all scores were effective and had very high sensitivity.^99^

The HEART score was developed using conventional troponin as a biomarker. However, retrospective studies that used highly sensitive troponin found results similar to those of validation studies.^100^^,^^101^ No studies have validated this tool for care outside the hospital. However, an ongoing study (ARTICA) aims to validate this score for pre-hospital care.^102^

A HEART score from 0 to 3 identifies 35% to 46% of patients at low risk, showing high sensitivity and negative predictive value, while patients with a score of 7 to 10 are at high risk, with an event rate > 50% in 6 weeks.^103^^-^^106^ The HEART score could better distinguish low-risk patients for major cardiac events than the GRACE and TIMI scores, with a lower loss rate and greater accuracy for initial risk stratification in the emergency department.^107^^,^^108^

The growing joint use of clinical data, ECG, highly sensitive troponin, and the systematized use of scores has amplified the effectiveness of diagnostic assessment protocols and the risk stratification of chest pain in the emergency department, which promotes safe early discharge and reduction of additional exams within 30 days.^109^

A randomized study that compared emergency care HEART scores with chest pain found a high negative predictive value for major cardiovascular events in the first year, with no differences observed regarding hospitalization or readmission to the emergency department.^110^

Thus, the impact of additional routine imaging exams on low-risk patients has been reconsidered because, despite the reduced diagnosis time, current evidence has shown that these additional tests have no verified benefit regarding the occurrence of AMI or relevant clinical events.^111^

#### 5.5. Nurses and the Chest Pain Protocol

When performed by qualified nurses, hospital screening improves the identification of high-risk patients and reduces door-to-ECG time.^112^ It has been shown that, in rural hospitals, emergency nurses have a high level of accuracy, which reduces waiting time and length of stay without compromising patient safety.^113^ Thus, nurses should be actively encouraged to participate in the screening of patients with chest pain, and the multidisciplinary team should receive continuing training about patients with chest pain.

A flowchart for emergency department treatment of patients with chest pain is shown in Figure 1.5.

**Table:** 

Rotina diagnóstica e critérios de internação — Sumário de recomendações e evidências		
A triagem inicial deve ser realizada com base em história clínica, exame físico, ECG de 12 derivações em até 10min e troponina.	I	A
O escore HEART deve ser utilizado para estratificação de risco e auxílio na decisão de alta hospitalar precoce.	I	B
Pacientes com escore HEART ≤ 3 associado à troponina em tempo hábil negativa, ECG sem alteração isquêmica e ausência de antecedentes de DAC podem ser liberados do serviço de emergência com segurança para reavaliação ambulatorial.	I	B
Triagem hospitalar realizada por enfermeiros habilitados, visando ao reconhecimento precoce de pacientes sob maior risco.	IIa	B
Uso de escore EDACS e ADAPT para estratificação de risco clínico como opção ao escore HEART.	IIa	B
Utilização do escore HEART visando à liberação precoce em pacientes atendidos por ambulância.	IIb	C

**Figure 1.5:** Flowchart of routine diagnosis of the patient with acute chest pain in the Emergency Department. HS: highly sensitive; TTE: transthoracic echocardiogram.

### 6. Emergency Procedures After Risk Stratification

#### 6.1. Indications for Early Invasive Strategy

An invasive strategy is indicated for patients with a very high risk of death (Table 1.8). This patient profile is not widely represented in randomized studies. Coronary intervention within 2 hours is indicated for these patients. When interventional cardiology service is not available, these patients should ideally be transferred to other centers.

**Table 1.8:** Indications for an invasive strategy

Hemodynamic instability or cardiogenic shock
Chest pain refractory to drug treatment
Malignant arrhythmias or cardiorespiratory arrest
Mechanical complications of infarction
Acute HF
Recurrent ST-T segment changes with intermittent ST-segment elevation

**Table:** 

Early invasive strategy - Summary of recommendations and evidence		
An early invasive strategy is indicated for patients with NSTE-ACS and refractory angina or hemodynamic or electrical instability (without severe comorbidities or contraindications for these procedures).	I	A

#### 6.2. Initial Treatment (Emergency Department/Ambulance)

##### 6.2.1. Supplemental Oxygen

Hypoxemia and myocardial ischemia can occur due to changes in the ventilation-perfusion ratio secondary to pulmonary arteriovenous shunting (due to an increase in LV end-diastolic pressure) and interstitial and/or pulmonary alveolar edema. Hypoxemia worsens myocardial ischemia, increasing myocardial injury. O2 saturation (SaO2) should be measured using digital oximetry in pre-hospital and/or ambulance care for early diagnosis of hypoxemia.

Supplemental oxygen therapy is indicated for patients with AMI who present hypoxia with SaO2 <90% or clinical signs of respiratory distress.^114^ However, in a randomized study of more than 6000 patients, oxygen therapy in patients with suspected ACS and SaO2 ≥90% was not associated with reduced mortality or other cardiovascular outcomes in 1 year of follow-up. ^115^

Oxygen therapy should not eliminate hypoxic respiratory stimulation when there is a chronic obstructive pulmonary disease or other causes of hypercapnia. Patients with pulmonary congestion, cyanosis, arterial hypoxemia, or respiratory failure should receive oxygen supplementation and be carefully monitored with serial blood gas analysis. Non-invasive ventilatory support should be used in certain cases with signs of respiratory failure, persistent pulmonary congestion, and hypoxemia. Treatment should progress to invasive ventilation in the event of circulatory shock, non-invasive ventilatory support failure, and in unstable patients indicated for urgent myocardial revascularization (PCI or CABG). The unnecessary administration of oxygen for a prolonged period can cause systemic vasoconstriction and can even be harmful.

**Table:** 

Oxygen therapy - Summary of recommendations and evidence		
Oxygen therapy (2 to 4 L/min) should be performed for intermediate- and high-risk patients with SaO2 <90% and clinical signs of respiratory distress.	I	C

##### 6.2.2. Analgesia and Sedation

The precordial pain and anxiety usually present in ACS lead to hyperactivity of the sympathetic nervous system. This hyperadrenergic state, in addition to increasing myocardial oxygen consumption, predisposes patients to atrial and ventricular tachyarrhythmias. Initial antianginal therapy with beta-blockers and nitrates should be performed as long as there are no contraindications, such as cardiogenic shock and/or hypotension. Morphine sulfate may be used in refractory cases or when there is a contraindication to nitrates or beta-blockers. The therapy should be administered intravenously, at a dose of 2 to 4 mg (diluted) every 5 minutes, up to a maximum of 25 mg. The low dosage is to avoid adverse effects such as hypotension and respiratory depression. Morphine derivatives should be avoided, except in cases of hypersensitivity, for which meperidine sulfate can be used in fractional doses of 20 to 50 mg intravenously.

Despite its efficacy in controlling angina, there is evidence that morphine reduces the antiplatelet effect of P2Y_12_ inhibitors, such as clopidogrel,^116^ and more potent antiplatelet drugs, such as prasugrel and ticagrelor.^117^^,^^118^

Regarding clinical events, a subanalysis of the CRUSADE^119^ registry and, more recently, a meta-analysis that included almost 70,000 patients found that early routine use of morphine may be associated with increased in-hospital mortality and major cardiovascular events.^120^ In a *post-hoc* analysis of the EARLY-ACS trial, it was found that morphine was associated with a greater risk of early ischemic events when used concomitantly with clopidogrel pretreatment. However, in the group of patients who did not receive clopidogrel before catheterization, morphine did not worsen the clinical outcome.^121^

Although the routine use of anxiolytics has become common practice in Brazil, it often seems excessive and should be reserved for special situations. A randomized, double-blind clinical trial with 131 male AMI patients found that the degree of anxiety, blood pressure, heart rate, and precordial discomfort did not differ between diazepam and placebo groups^122^. Diazepine derivatives have been the most commonly used drug in this indication.

Nonsteroidal anti-inflammatory drugs (NSAIDs) should not be used (except ASA) to control pain in AMI patients since they increase the risk of major cardiovascular events. NSAID use should be suspended in patients who used them prior to hospitalization due to the worsening cardiovascular prognosis associated with elevated blood pressure, the risk of acute kidney injury, and blood hyperviscosity.^123^

**Table:** 

Analgesia and sedation - Summary of recommendations and evidence		
Administer morphine sulfate in patients who have continuous pain despite optimized anti-ischemic therapy.	IIb	B
Administer benzodiazepines to patients with signs and symptoms of anxiety.	IIb	C
NSAIDs should not be administered (with the exception of ASA) in patients with suspected AMI.	III	

##### 6.2.3. Glycemic Control

More than 30% of AMI patients have DM or are unaware of their diagnosis. These individuals have higher bleeding rates and a worse 30-day prognosis than patients with normal blood glucose levels.^124^^-^^127^ Thus, glycemic control protocols should be used for AMI patients with significant hyperglycemia (> 180 mg/dL). This therapy aims to reduce blood glucose levels and avoid hypoglycemic episodes (< 70 mg/dL), which can cause several harmful effects, including the expansion of the AMI area. In patients at higher risk of hypoglycemia, such as older adults, those with chronic kidney disease, and those with residual effects from oral and/or fasting hypoglycemic agents, glycemic control should be adjusted to tolerate slightly higher blood glucose levels and thus prevent hypoglycemia.

**Table:** 

Glycemic control - Summary of recommendations and evidence		
The blood glucose levels of all patients with suspected ACS at admission should be measured, and the blood glucose of diabetic patients or those who present hyperglycemia during hospitalization should be monitored.	I	C
Glycemic control with intermittent insulin protocols should be considered carefully in patients with glycemic levels > 180 mg/dL to avoid episodes of hypoglycemia.	IIa	C
In patients at higher risk of hypoglycemia, such as older adults, those with kidney disease, and those with residual effects from oral and/or fasting hypoglycemic agents, glycemic control should be adjusted to tolerate slightly higher blood glucose levels and thus prevent hypoglycemia.	IIa	C

##### 6.2.4. Anti-ischemic Therapy

The goal of anti-ischemic therapy is to reduce oxygen consumption (by decreasing the heart rate, blood pressure, and myocardial contractility) or to increase oxygen supply (by administering oxygen or promoting vasodilation).

###### 6.2.4.1. Nitrates

The therapeutic benefits of nitrates are related to their effects on peripheral and coronary circulation. Their vasodilator effect, which decreases venous return to the heart and LV end-diastolic volume, reduces oxygen consumption by the myocardium. Additionally, they have vasodilatory effects on coronary arteries (whether normal or atherosclerotic) and redirect intracoronary flow, with increased collateral circulation and platelet aggregation inhibition. In addition to their symptomatic effects, nitrates reduce pulmonary congestion, mainly by lowering systemic venous return.

No controlled clinical studies have tested nitrates' effects on clinical outcomes and mortality in NSTE-ACS, although their use is universally accepted. UA studies that have evaluated them have been small and observational.^128^^-^^132^ Nitrates are associated with the improvement of both anginal and congestive symptoms, although they have no impact on mortality, infarction, or the need for revascularization.

Treatment is initiated in the emergency department, with sublingual administration (nitroglycerin, mononitrate, or isosorbide dinitrate). If rapid pain relief does not occur, these patients may benefit from intravenous administration (nitroglycerin and isosorbide mononitrate are available in Brazil).

Nitrates are contraindicated in the presence of significant arterial hypotension (systolic blood pressure [SBP] < 100 mmHg), the use of sildenafil in the last 24 hours, or the use of tadalafil in the previous 48 hours. A common side effect is a headache. The sublingual use of nitroglycerin (0.4 mg tablet), isosorbide dinitrate (5 mg tablet), or isosorbide mononitrate (5 mg tablet) should not exceed three tablets, administered at 5-min intervals. Intravenous nitroglycerin is used at a dose of 10 *µ*g/min in increments of 10 *µ*g every 5 min until symptoms improve, blood pressure is reduced (the drop in SBP should not exceed 30% or reach < 110 mmHg), or the heart rate increases (> 10% of baseline). Tolerance to the hemodynamic effects of this medication can be expected after the first 24 hours of use. Intravenous treatment should continue 24 to 48 hours after the last angina attack and should be suspended gradually.

**Table:** 

Nitrates - Summary of recommendations and evidence		
Sublingual nitrates can be used to relieve angina.	I	C
Intravenous nitrates can be used to control persistent angina, high blood pressure, or signs of congestion.	I	C

###### 6.2.4.2. Beta-blockers

As with nitrates, few controlled clinical trials have been conducted on beta-blockers in UA. Evidence for their beneficial effects is based on their mechanism of action, the results of small controlled clinical trials, and the extrapolation of results from studies on stable angina and STEMI. Beta-blockers competitively inhibit the effects of circulating catecholamines. In UA, their benefits are related to their action on beta-1 receptors. They decrease heart rate, blood pressure, and myocardial contractility, causing reduced myocardial oxygen consumption. Despite the lack of large-scale randomized trials evaluating their action on hardclinical outcomes, such as mortality in patients with NSTE-ACS, these drugs, together with nitrates, are considered agents of the first choice for ACS treatment the emergency department in patients without contraindications. Only a few small studies have compared beta-blockers with placebo in UA.^133^^-^^135^ Although no mortality-reducing effect was found, a meta-analysis of five small studies ^136^ on beta-blocker therapy in 4,700 UA patients showed a 13% reduction in the relative risk of progression to AMI. A Brazilian study in patients with NSTE-ACS found a significant association between oral beta-blocker therapy in the first 24 hours of hospitalization and lower in-hospital mortality, regardless of LV dysfunction.^137^

Despite involving patients with STEMI, the COMMIT trial ^138^ suggested that the routine use of a high intravenous dose of beta-blocker followed by oral administration could increase the incidence of cardiogenic shock, mainly when used in the first 24 to 48 hours or in patients with LV dysfunction or risk factors for cardiogenic shock (age over 70 years, heart rate > 110 bpm/min or systolic pressure < 120 mmHg). Although no randomized clinical trial specifically for a population with NSTE-ACS has been conducted, a database study with more than 20,000 patients found results similar to the COMMIT study.^139^

Thus, the routine use of oral beta-blockers is recommended in patients without contraindications (Part 2 of this Guideline provides further details). If the patient has persistent ischemic pain and/or tachycardia (non-compensatory), the intravenous formulation can be used. Various therapeutic regimens can be used depending on the selected beta-blocker. There is no evidence that one beta-blocker is superior to others. Beta-blockers (carvedilol, bisoprolol, and metoprolol succinate) are highly recommended for patients who develop ventricular dysfunction (LVEF <40%) without pulmonary congestion.

The following is a dosage list for metoprolol and atenolol, the most commonly used beta-blockers in Brazil:

Metoprolol: Intravenous – 5 mg (1 to 2 min) every 5 min until a maximum dose of 15 mg is reached.

Oral - 50 to 100 mg every 12 hours, beginning 15 min after the last intravenous dose.

Atenolol: Intravenous – 5 mg (1 to 2 min) every 5 min until a maximum dose of 10 mg is reached.

Oral - 25 to 50 mg every 12 hours, beginning 15 min after the last intravenous dose.

During intravenous administration, careful monitoring of heart rate, blood pressure, ECG, and pulmonary auscultation should be performed.

Beta-blockers should not be used during acute intoxication in patients with symptoms of vasospasm due to cocaine use. In patients with asthma or chronic obstructive pulmonary disease, beta-blockers are contraindicated only in the presence of bronchospasm; in such cases, beta-1 selective beta-blockers are recommended.

**Table:** 

Beta-blockers - Summary of recommendations and evidence		
Administer oral beta-blockers in the first 24 hours to patients without contraindications (signs of HF, low output symptoms, increased risk of cardiogenic shock, or other contraindications to beta-blockers).	IIa	B
Administer intravenous beta-blockers in intermediate- and high-risk patients with persistent ischemia, tachycardia, and hypertension provided that they do not show clinical/radiological signs of HF.	IIb	B
Administer intravenous beta-blockers to patients with risk factors for cardiogenic shock.	III	A

##### 6.2.5. Initial Antithrombotic Therapy (Ambulance/Emergency Department)

###### 6.2.5.1. Oral Antiplatelet Therapy

The phenomenon of coronary atherosclerotic plaque instability, a recognized mechanism of ACS, results from rupture or erosion, with subsequent platelet activation and thrombosis.^140^

This concept has guided advances in antiplatelet therapy and the current idea of dual therapy with ASA and P2Y_12_ inhibitors in patients with ACS, determining which should receive invasive strategy and the initially proposed clinical treatment.^141^

The characteristics of and scientific evidence for antiplatelet drugs are described in detail in Part 2. However, a critical decision in the emergency department is for the time for beginning P2Y_12_ inhibitors, particularly in patients undergoing early invasive strategy with cardiac catheterization and PCI. The term "pre-treatment" generally refers to administering P2Y_12_ inhibitors before determining the coronary anatomy, either in the ambulance or the emergency department.

####### 6.2.5.1.1. The Basics of Pretreatment with P2Y12 Inhibitors

The goal of pre-treatment with a second potent antiplatelet agent in NSTE-ACS is to achieve effective platelet inhibition and prevent further aggregation, which would prepare the patient for invasive strategy, potentially reducing the extent of thrombosis, as well as the risk of recurrent AMI and PCI-related complications, such as stent thrombosis and periprocedural infarction.^142^

In contrast, pre-treatment involves an increased risk of bleeding, associated or not with coronary artery bypass surgery. This factor is associated with a relevant increase in morbidity and mortality in ACS.^143^^-^^145^

One important issue is determining the minimum time required for clinically useful action, knowing that even the most potent oral antiplatelet agents need at least 30 to 60 minutes for effective platelet inhibition, thus providing "appropriate pretreatment" before catheterization and PCI.^146^^-^^149^

Following pretreatment with P2Y_12_ inhibitors, a second antiplatelet agent was used in the CURE (clopidogrel)^150^^,^^151^ and PLATO (ticagrelor) trials;^152^^,^^153^ in the TRITON trial, prasugrel was only administered after the coronary anatomy had been determined.^154^

####### 6.2.5.1.2. Comparative Studies on Pretreatment vs. Second Antiplatelet Use in the Catheterization Unit

The ARMYDA-5 PRELOAD trial assessed clopidogrel pretreatment in NSTE-ACS (n = 409) and stable angina patients and found no reduction in adverse cardiovascular events in either group.^155^

The CREDO trial was designed to assess the benefits of pretreatment with clopidogrel and ASA in patients undergoing PCI. In this controlled trial, 2116 patients (39% with ACS and 61% with stable CAD) received 300 mg of clopidogrel (n = 1053) or placebo (n = 1063) 3 to 24 hours before PCI. The average time between the loading dose of clopidogrel and PCI was 9.8 hours, with 51% and 49% of the patients receiving the loading dose 3 to 6 hours and 6 to 24 hours before PCI, respectively. No significant reduction was found in the combined outcome (death, infarction, or urgent revascularization of the target vessel) in 28 days. However, when analyzed according to treatment time (3 to 6 hours, 6 to 12 hours, or 12 to 24 hours before PCI), a significant interaction was observed: clopidogrel pretreatment < 6 hours before PCI had no effect, but treatment 6 to 12 hours and 12 to 24 hours before PCI resulted in 35.5% and 40.1% relative reductions in the combined final outcome, respectively. These findings support the hypothesis that clopidogrel requires more time to act when used as a pretreatment.^156^

The ACCOAST clinical trial (n = 4,033) was a randomized, multicenter controlled study that compared administration of 60 mg of prasugrel in the catheterization room at the time of PCI versuspretreatment with 30 mg of prasugrel and a second 30 mg dose during coronary angiography within 2 to 48 hours after randomization.^157^ The primary composite endpoint was death from cardiovascular causes, AMI, stroke, urgent revascularization, or rescue therapy with GP IIb/IIIa inhibitors by the seventh day. PCI was performed in 69% of the patients, a mean of 4.3 hours after the initial loading dose. Only 9% of the patients had a GRACE score > 140. The incidence of the primary endpoint at seven at 30 days did not differ significantly between the pretreatment and placebo groups (10.0% vs. 9.8%, hazard ratio [HR] 1.02, 95% CI 0.84-1.25, p = 0.81). A higher rate of major bleeding (related or not to CABG) was found in the pretreatment group (HR, 1.90; 95% CI, 1.19-3.02; p = 0.006). The rates of major bleeding (TIMI) or life-threatening bleeding unrelated to CABG were 3 and 6 times higher in the pretreatment and placebo groups, respectively. Half of all episodes were related to urgent revascularization surgery, and half of the major bleeding events unrelated to CABG were caused by vascular bleeding at the access site.

The DUBIUS randomized clinical trial evaluated 1,449 NSTE-ACS patients whose coronary angiography was scheduled within 72 hours, comparing pretreatment with ticagrelor versus. a second antiplatelet agent, either prasugrel or ticagrelor (randomization 1:1), administered in the hemodynamics unit in patients indicated for PCI.^158^ The population had a moderate ischemic risk profile (mean GRACE score = 122) and did not have a high hemorrhagic risk (mean CRUSADE score = 22). Pre-specified interim futility analysis guided early interruption of the study. After 30 days, the primary outcome, i.e., death due to vascular causes, non-fatal infarction or non-fatal stroke, and hemorrhagic phenomena (Bleeding Academic Research Consortium [BARC] types 3, 4, and 5), did not differ significantly between the groups (2.9% and 3.3%, respectively, for no pretreatment vs. pretreatment). This finding was consistent in the subgroup that underwent PCI (72% of the population); the meantime until PCI was 23.3 hours, which was notably longer than the ACCOAST study (4 hours). The findings, however, were similar to those who underwent coronary angiography after 24 hours. An original strategy of this study was that the non-pretreatment group still received ticagrelor, although a drug comparison could not be performed. Despite the analysis limitations due to early interruption, pretreatment in NSTE-ACS with invasive handling showed no benefits, although the results indicate that an individualized choice of drugs is possible in the hemodynamics unit.

####### 6.2.5.1.3. Pretreatment Data in Meta-analysis

A meta-analysis that included 16 studies, including 61,517 patients with ACS with or without ST-segment elevation, found a significantly lower incidence of major adverse cardiac events in the subgroup with NSTE-ACS allocated to pretreatment with clopidogrel (OR = 0.83; 95% CI 0.71- 0.96, p = 0.01), and no increase in major bleeding in patients who underwent PCI within 48 hours.^159^

Another meta-analysis of 23 studies and 60,907 patients aimed to compare the efficacy and safety of P2Y_12_ inhibitors (clopidogrel, ticagrelor, and prasugrel) administered at two different periods compared to PCI: early (< 2 hours pre-PCI) vs. late (> 2 hours pre-PCI or post-PCI). In studies that included patients with NSTE-ACS pretreatment with ticagrelor or prasugrel showed no benefit, but prasugrel increased the risk of bleeding in this population.^160^

As previously explained, the routine use of a second oral antiplatelet agent in the initial treatment of NSTE-ACS has shown no clear benefits, especially for invasive strategies in emergencies, where the drug can be determined in the catheterization room. However, pretreatment can be considered in patients without a critical hemorrhagic risk, with moderate or high ischemic risk, when a coronary angiography has been scheduled, or during initial conservative treatment in full anti-ischemic therapy.

The challenge of balancing ischemic and bleeding risks requires a personalized approach to the use and type of second antiplatelet. The patient's clinical characteristics, the availability of coronary angiography, efficacy and safety data, and the pharmacodynamic properties of P2Y_12_ inhibitors available in Brazil must be considered. However, it should be pointed out that prasugrel is contraindicated for pretreatment when the coronary anatomy has not yet been determined. Although any P2Y_12_ inhibitor can be used in the catheterization room, in the absence of contraindications, prasugrel or ticagrelor are preferred due to their rapid onset and greater antithrombotic efficacy. Clopidogrel can be used when there is a very high hemorrhagic risk (Figure 1.6).

**Table:** 

Initial antiplatelet therapy - Summary of recommendations and evidence		
In all patients without contraindications, ASA should be used in the emergency department as early as possible at an initial dose of 150 to 300 mg (in patients who have not previously used it) and a maintenance dose of 75 to 100 mg.	I	A
A P2Y_12_ inhibitor is recommended in addition to ASA, except when contraindicated or when there is an increased risk of bleeding.	I	A
Pretreatment with a P2Y_12_ inhibitor is recommended in unstable and/or high-risk patients indicated for immediate invasive strategy; it is recommended for use in the catheterization room when coronary anatomy has been determined, and PCI has been scheduled.	I	B
There is no routine indication for P2Y_12_ inhibitors as a pretreatment in patients indicated for early invasive strategy (< 24 h).	I	B
In patients with no history of previous stroke or transient ischemic loading (TIA), prasugrel (loading dose) should be used in the hemodynamics unit only after angiography and if the coronary intervention has been scheduled	I	B
Ticagrelor (loading dose) should be used regardless of the initial strategy (conservative or invasive).	I	B
Clopidogrel (loading dose) should be used regardless of the initial strategy (conservative or invasive) when ticagrelor or prasugrel is unavailable or contraindicated.	I	B
In patients allergic to ASA, initial monotherapy with a P2Y_12_ inhibitor (preferentially ticagrelor or prasugrel) is recommended.	I	C
Clopidogrel (loading dose) should be used regardless of the initial strategy (conservative or invasive) in patients at very high risk of bleeding or who need long-term oral anticoagulation.	IIa	C
Prasugrel can be used in the pretreatment	III	

**Figura 1.6:** Initial antithrombotic therapy in non-ST-elevation acute coronary syndromes. CC: cardiac catheterization; HU: hemodynamics unit; SC: subcutaneously; UFH: unfractionated heparina.

###### 6.2.5.2. Glycoprotein IIb/IIIa Inhibitors

Activation of platelet surface receptors, called GP IIb/IIIa, is the final mechanism of platelet aggregation. It results from a morphological alteration in the receptor, which increases its affinity to bind to fibrinogen, a protein that acts as a bridge between two platelets. These drugs have been used in clinical situations that have great potential for platelet activation, such as complex PCIs and thrombotic complications, e.g., no-reflow after angioplasty.

Abciximab is a monoclonal antibody that acts as a non-competitive and irreversible GP IIb/IIIa inhibitor. It has a short plasma half-life of 5 to 10 minutes, and its biological half-life is 6 to 12 hours after injection of an isolated bolus. These therapeutic doses block 80% to 90% of surface receptors. Fifty percent of these receptors still remain blocked one week after use. The recommended dose is 0.25 mg/kg in a bolus, followed by 0. 25*µ*g/kg for 12 hours.

Tirofiban, a synthetic derivative, is a non-peptide, small molecule GP IIb/IIIa inhibitor whose molecular structure includes the RGD sequence (arginine-glycine-aspartate), an integrin recognition site present in adhesive proteins of fibrinogen, von Willebrand factor, vetronectin, etc. Tirofiban also acts competitively at GP IIb/IIIa receptors, preventing them from binding to fibrinogen. The recommended dose is 0.4 *µ*g/kg/min for 30 min, followed by a maintenance dose of 0.11 *µ*g/kg/min for 12 to 24 hours after the procedure (maximum 96 hours). If the first administration is in the catheterization room, it should begin at a dose of 1010 *µ*g/kg, administered in a bolus in 3 minutes, followed by 0.15 *µ*g/kg/min.

The efficacy of abciximab and tirofiban is comparable when a large bolus of tirofiban (25 *µ*g/kg) is injected. However, abciximab is superior to tirofiban when used with a normal bolus of 10g/kg.^161^^,^^162^

Generally speaking, GP IIb/IIIa inhibitors tend to increase bleeding risk, and thrombocytopenia, although rare, is a non-negligible complication.

In patients with NSTE-ACS who undergo essentially "conservative" strategies (i.e., without early intervention), the use of GP IIb/IIa inhibitors is based on studies in which, in addition to heparinization, platelet activation was systematically antagonized only by ASA.^163^^-^^170^ A meta-analysis of > 30,000 patients found a 9% reduction in the relative risk of death or infarction at 30 days of follow-up (p = 0.015) with beta-blockers,^169^ although the benefits were restricted to higher-risk patients, mainly with elevated troponin and/or depressed ST-segment and/or undergoing PCI (24% at 30 days of follow-up).

Several studies have tested the role of GP IIb/IIIa inhibitors in patients undergoing PCI.^171^^-^^177^ The results of these studies are more homogeneous, invariably demonstrating benefits, although there is a significant increase in bleeding. It should be noted that these studies did not involve routine use of thienopyridine upon patient arrival at the hospital. A meta-analysis of studies that analyzed the role of GP IIb/IIIa inhibitors in ACS patients with or without ST elevation found, in addition to significant decreases in death and (re) infarction (p <0.0001), a 21% decrease (95% CI: 0.64-0.67) in the relative risk of death at six months of follow-up.^178^

The ISAR-REACT-2 trial involved 2,022 patients with NSTE-ACS who received dual antiplatelet therapy (DAPT) with ASA + clopidogrel, adding abciximab was compared to placebo. Abciximab was associated with a lower incidence of the composite outcome, including death, AMI, and urgent revascularization (risk ratio [RR] 0.75; CI 0.58-0.97; p = 0.03), and no significant increase in serious or non-serious hemorrhagic outcomes. It should be pointed out that abciximab only benefitted individuals with troponin elevation.^179^

The ACUITY-Timing trial included 9207 patients with NSTE-ACS (98% with pre-angiography ASA and 65% with pre-angiography P2Y_12_ inhibitor) and investigated the best time to administer GP IIb/IIIa inhibitors, randomizing patients to the routine early tirofiban or eptifibatide in the emergency room before coronariography versus the selective use at the cath lab whenPCI is about to be performed. It was found that the routine use of GP IIb/IIIa inhibitors did not reduce ischemic outcomes, and the incidence of significant bleeding increased significantly (RR = 0.80, p < 0.001).^180^

Similar results were found in the EARLY-ACS study, in which 9,492 patients with NSTE-ACS were randomized to platelet anti-aggregation with eptifibatide before catheterization vs. selected use before angioplasty (based on angiographic aspects). Again, the routine use of GP IIb/IIIa inhibitors did not reduce ischemic complications and increased the incidence of bleeding, as well as the need for transfusions.^181^

Based on this body of evidence, the use of GP IIb/IIIa inhibitors as a third antiplatelet agent should be reserved for patients who do not have a high risk of hemorrhaging but are at high ischemic/thrombotic risk according to clinical and angiographic criteria.

###### 6.2.5.3. Anticoagulants

Anticoagulant therapy should be administered as quickly as possible to all patients with NSTE-ACS since the use of these compounds reduces the incidence of AMI and death when used within hours of diagnosis.^182^

The choice and timing of the anticoagulant are determined by the treatment strategy (invasive or conservative), the severity of the clinical presentation, and the particularities of each hospital department. In patients with an initial conservative treatment, enoxaparin or fondaparinux is preferential. The recommended doses are shown in Table 1.9. Enoxaparin should not be administered to patients with creatinine clearance < 15 mL/min, and fondaparinux should not be administered to patients with creatinine clearance < 20 mL/min. For patients whose clearance is between 15 and 30 mL/min and in obese patients (BMI > 30 kg/m^²^ or weight > 100 kg), the enoxaparin usage guidelines recommended anti-factor Xa monitoring to minimize the risk of excessive doses and severe bleeding.^183^^-^^185^

**Table 1.9:** Anticoagulant dosage according to renal function

Medication	Recommendation		
	Normal renal function or stage 1-3 CRF (creatinine clearance ≥ 30 mL/min/1.73 m²)	Stage 4 CRF (creatinine clearance between 15 and 29 mL/min/1.73 m²)	Stage 5 CRF (creatinine clearance < 15 mL/min/1.73 m²)
Unfractionated heparin	Before coronary angiography: 60 to 70 IU/kg IV bolus (max. 5000 IU) and infusion (12 to 15 IU/kg/h) with PTT target 1.5-2.5 × controlDuring coronary angiography: 70 to 100 IU/kg IV in non-anticoagulated patients or 50 to 70 IU/kg if concomitant use with GP IIb/IIIa inhibitors	No dose adjustment	No dose adjustment
Enoxaparin	1 mg/kg SC every 12 h> 75 years: 75% of the dose	1 mg/kg every 24 h	Not recommended
Fondaparinux	2.5 mg SC every 24 h	Not recommended if clearance < 20 mL/min/1.73 m²	Not recommended

CRF: chronic renal failure; IV: intravenously; PTT: Partial thromboplastin time; SC: subcutaneously.

In morbidly obese patients, pharmacodynamic studies and observational data identified a frequent need for dose adjustment according to anti-factor Xa level to achieve the therapeutic goal (BMI > 40 kg/m^²^), which suggests the usefulness of monitoring when available.^186^^,^^187^

However, in the CRUSADE registry, which assessed obese patients with ACS, a standard dose of enoxaparin (1 mg/kg/weight) in patients > 150 kg was associated with a greater risk of bleeding than in a subgroup of patients between 120 and 150 kg. (11.4% vs. 5.6%, p < 0.001). UFH is an alternative for these patients.^188^^,^^189^

Enoxaparin, fondaparinux, and UFH are options for invasive strategy. Despite the fact that enoxaparin and UFH had similar efficacy in the SINERGY study, for very high-risk patients who require immediate catheterization, using UFH in the emergency department or, preferably, in the catheterization room can prevent crossing over from enoxaparin to UFH and the greater hemorrhagic risk that results from this practice.^190^

For other patients, the choice should be guided by the patient's ischemic and hemorrhagic risk and the department's own experience.

Enoxaparin is preferred to UFH because systematic review data from randomized trials across the ACS spectrum show that enoxaparin is more effective than UFH at preventing the composite outcome of all-cause mortality or severe bleeding in patients with NSTE-ACS who undergo PCI.^191^^-^^193^

Fondaparinux is a safe option for non-invasive treatment. If catheterization is subsequently indicated, UFH is recommended during the procedure due to the risk of catheter thrombosis.^194^^,^^195^

Patients who have previously used direct-acting oral anticoagulants (DOAC) in ACS should be handled with caution to avoid increasing the risk of hemorrhage. There is no evidence that early parenteral anticoagulation or PCI can be performed. In urgent situations, PCI should be performed regardless of when the last dose of DOAC was administered. The procedure should be postponed in patients with lower ischemic risk. In patients with normal renal function (creatinine clearance > 50 mL/min), the effect of DOAC is reduced 24 hours after the last dose. In patients with renal dysfunction, this period is 48 hours. Thus, the patient can undergo PCI with less risk of bleeding. Parenteral anticoagulation is indicated in patients who undergo emergency PCI regardless of when the last dose of DOAC was administered.^196^

For hemodynamic/PCI examinations, it has been shown that the radial approach is the most effective strategy to reduce bleeding, resulting in reduced mortality in patients with ACS.^197^

**Table:** 

Anticoagulants - Summary of recommendations and evidence		
UFH should be used in patients with severe renal dysfunction (clearance < 15 mL/min).	I	A
Enoxaparin should be used until revascularization (for eight days or until hospital discharge).	I	A
Fondaparinux can be used for eight days or until hospital discharge as an alternative to enoxaparin, especially in patients at high bleeding risk.	I	B
In patients using fondaparinux, administer UFH 85 IU/kg intravenously at the time of PCI or 60 IU/kg in those receiving GP IIb/IIIa inhibitors.	I	B
UFH should be used in patients weighing > 150 kg.	IIa	B
Enoxaparin is preferable to UFH in patients with a clearance ≥ of 15 mL/min/1.73 m²), unless myocardial revascularization surgery is planned within 24 h.	IIa	B
UFH is preferred in emergency patients who require hemodynamic study.	IIa	C
Heparin cross-over (UFH and enoxaparin).	III	
Enoxaparin can be used in patients with creatinine clearance < 15 mL/min and weight > 150 kg.	III	
Fondaparinux can be used in patients with creatinine clearance < 20 mL/min.	III	
Fondaparinux can be used alone in PCI.	III	

## Part 2 – Treatment During Hospitalization

### 1. Hospitalization and Coronary Care Unit Discharge

Patients with intermediate- and high-risk NSTE-ACS should remain hospitalized in a coronary care unit whenever possible until the definitive treatment is implemented. After the PCI is completed, the patient must return to the coronary care unit and remain for 12 to 24 hours if there are no complications. Post-PCI complications are serious outcomes, such as occlusion of the target vessel, the need for emergency revascularization surgery or a new unscheduled PCI, recurrent angina, and death. When a CABG procedure is indicated, patients should remain hospitalized (ICU or Coronary Unit) until surgery is performed. When intravenous drug treatment is indicated, patients should remain in the coronary care unit until they are stable and can be discharged and prescribed oral medications.

### 2. Nitrates

No controlled clinical trials have tested the effects of nitrates on clinical outcomes and mortality in UA, although their use is universally accepted; only small and observational studies have investigated them.^128^^-^^130^^,^^198^ Borzak et al. evaluated the effects of pre and in-hospital antianginal medication (calcium antagonists, beta-blockers, and nitrates) on clinical outcomes, mortality, and non-fatal myocardial infarction in 410 patients. No benefits were found. Multivariate analysis also showed no correlation with the incidence of death, nonfatal infarction, or recurrent angina.^199^

**Table:** 

Hospital nitrates - Summary of recommendations and evidence		
Nitrates should be used in intermediate- and high-risk patients who remain symptomatic or to control blood pressure.	I	C
Use associated with arterial hypotension, right ventricular infarction. Use of the phosphodiesterase inhibitors sildenafil and vardenafil within 24 h and tadalafil within 48 h (20 mg dose).	III	A

### 3. Beta-blockers

Clinical experience with beta-blockers is limited to small controlled clinical studies whose results are extrapolated to patients with UA and STE-ACS. Despite the lack of large-scale randomized studies, beta-blockers, such as nitrates, are widely used. Further details about this drug class are provided in Part 1.

### 4. Calcium Channel Antagonists

Calcium channel antagonists are a heterogeneous class of drugs, some of which act as negative regulators of chronotropism and coronary vasodilators. Their anti-ischemic action occurs by reducing calcium influx through the cell membrane, which reduces myocardial contractility, vascular tone, atrioventricular (AV) conduction, and sinus node activity.

These agents differ in terms of their ability to produce arterial vasodilation, reduce myocardial contractility, and delay AV conduction. Their beneficial effects in NSTE-ACS are due to a combination of decreased myocardial oxygen consumption, afterload, myocardial contractility, and heart rate, in addition to increased oxygen supply to the myocardium due to coronary vasodilation. Coronary vasodilation among different agents is similar. Dihydropyridines cause greater peripheral arterial vasodilation and tend to produce reflex tachycardia (more evident with short-acting nifedipine); verapamil and diltiazem tend to cause bradycardia by reducing chronotropism and dromotropism, which can lead to atrioventricular block (most evident with verapamil). These drugs should generally be avoided in patients with impaired LV function and/or changes in atrioventricular conduction. Water retention is another side effect that usually appears only after prolonged use.

Calcium channel antagonists are as effective as beta-blockers for controlling symptoms.^200^^,^^201^ However, they do not reduce the incidence of refractory angina, infarction, or death; on the contrary, a meta-analysis suggests that they increase the incidence of these complications.^202^ So far, only first-generation representatives have been assessed in UA. Although these deleterious results were observed in all tested classes of calcium antagonists ^135^^,^^136^^,^^203^, conclusive data on dihydropyridines do not exist. On the other hand, in patients with NSTEMI, there is evidence that diltiazem and verapamil (which are not associated with induced tachycardia) may have a protective effect.^204^^,^^205^ These agents can be used to control refractory ischemic symptoms in patients already using nitrates and beta-blockers in appropriate doses or in patients who cannot tolerate these drugs (especially in cases of contraindication), or even in cases of variant angina of Prinzmetal. However, the routine use of calcium channel antagonists is not recommended, and fast-acting nifedipine is particularly contraindicated.

**Table:** 

Calcium channel antagonists - Summary of recommendations and evidence		
Use in intermediate- and high-risk patients. Non-dihydropyridine derivatives can be used in patients contraindicated to beta-blockers and without LV dysfunction.	I	B
Use in patients with variant angina of Prinzmetal.	I	B
Long-acting dihydropyridines can be used in patients with refractory ischemia and without ventricular dysfunctionwho are taking appropriate doses of nitrates and beta-blockers.	IIa	B
Dihydropyridine derivatives (rapid onset) can be used in high-risk patients who are taking appropriate doses of beta-blockers.	IIb	B
Dihydropyridine derivatives (rapid onset) can be used in patients who are not taking appropriate doses of beta-blockers.	III	B

### 5. Antiplatelet Drugs

#### 5.1. Acetylsalicylic acid

ASA is the antiplatelet drug of choice and should always be prescribed, except in cases of intolerance or adverse events.^206^^-^^209^ Its importance in NSTE-ACS treatment is based on studies published in the 1980s,^210^^,^^211^ which, for the most part, had relatively small samples and a low outcome incidence. However, in general, they found that ASA clearly reduced non-fatal AMI and mortality in the short and medium terms.

The recommended dosage for ASA is 150 to 300 mg for the loading dose, followed by 75 to 100 mg daily for the maintenance dose. In one of its arms, the CURRENT OASIS-7 trial tested a high maintenance dose of ASA in patients with ACS (about 70% of patients without ST elevation). There was no difference between the normal (75 to 100 mg per day) and high maintenance doses (300 to 325 mg/day) for serious cardiovascular events (mortality, non-fatal AMI, or stroke, p = 0.61). There was also no difference regarding the occurrence of severe bleeding (p = 0.90).^212^

#### 5.2. P2Y_12_ Inhibitors

Thienopyridine derivatives (clopidogrel and prasugrel) are platelet-activating antagonistic drugs mediated by adenosine diphosphate (ADP). They irreversibly inhibit P2Y_12_ platelet receptors, reduce the level of circulating fibrinogen, and partially block GP IIb/IIIa receptors, impeding binding to fibrinogen and von Willebrand factor. In addition to ticagrelor (derived from cyclopentyltriazolopyrimidine, a non-thienopyridine agent), a reversible inhibitor of ADP-induced platelet aggregation, these are the three platelet receptor P2Y_12_ inhibitors currently available in Brazil and should preferably be used in association with ASA.

##### 5.2.1. Clopidogrel

CURE, the first large clinical trial to test the role of clopidogrel in addition to ASA in NSTE-ACS, followed more than 12,000 patients for 3 to 12 months (average of 9 months). At the end of follow-up, there was a 20% reduction (RR 0.80; 95% CI 0.72-0.89; p = 0.00005) in the incidence of events (cardiovascular death, AMI or stroke) in the clopidogrel group + ASA vs. the placebo + ASA group, at the expense of increased bleeding incidence in the clopidogrel + ASA group (RR 1.38, p = 0.001).^213^ The beneficial effects of DAPT occurred in patients of all risk levels. Other CURE analyses demonstrated that DAPT would benefit both surgical myocardial revascularization patients and patients receiving isolated drug treatment.^214^

Based on ACS studies and a study that focused primarily on PCI^215^, DAPT becomes imperative when NSTE-ACS patients are treated with PCI. In the context of ACS, the CURE sub-study (PCI-CURE),^151^ , and the CREDO study have been developed.^156^

Due to clopidogrel's limitations regarding metabolism and drug interactions, a double dose was also tested in the CURRENT OASIS-7 trial.^216^ In this study, 25,086 invasive strategy ACS patients (71% without ST elevation) were randomized to receive a double dose of clopidogrel (600 mg loading dose followed by 150 mg daily for 6 days and 75 mg daily after the first week) or the usual dosage (300 mg loading dose followed by 75 mg daily). At the end of 30 days, there was no significant difference in the primary outcome (cardiovascular death, non-fatal AMI, or stroke) between groups (HR 0.94; 4.2% vs. 4.4%, p = 0.30). However, the double dose was associated with a significant increase in major bleeding (HR 1.24; 2.5% vs. 2%, p = 0.01). In a pre-specified analysis,^212^ considering only patients treated with PCI (n = 17,263), there was a 14% reduction in the primary composite outcome at 30 days (HR 0.86; 3.9% vs. 4.5 %, p = 0.039, number needed to treat = 167), in addition to a significant reduction in definite stent thrombosis (HR 0.54; 0.7% vs. 1.3%, p = 0.0001), although there was a higher incidence of severe bleeding (HR 1.41; 1.6% vs. 1.1%, p = 0.009, number needed to harm = 200). Considering the high number needed to treat, a cautious assessment of the patient's prior bleeding risk is recommended when determining the dose.

Patients who do not reach the expected level of platelet inhibition with clopidogrel are referred to as having a “poor response” (or “resistance”). This is identified by*in vitro* laboratory tests that quantify the intensity of platelet aggregation mediated by ADP. Poor clopidogrel response has been consistently associated with a higher incidence of thrombotic events, especially in patients undergoing PCI with stenting.^217^^-^^219^ Currently, three main factors are related to poor clopidogrel response: 1) genetic variability, characterized by polymorphisms associated with cytochrome P450 enzymes involved in the hepatic metabolization process, notably CYP2C19;^220^ 2) changes in intestinal absorption related to P-GP expression in intestinal epithelial cells;^221^ 3) concomitant use of other drugs that could interfere with liver metabolism mediated by cytochrome P450 enzymes, such as ketoconazole (which inhibits cytochrome P450 and reduces the action of clopidogrel) and rifampicin (which stimulates cytochrome P450 and enhances the action of clopidogrel).

Several *in vitro* studies have indicated that platelet inhibition is reduced when proton pump inhibitors are associated with clopidogrel, which could be especially frequent with omeprazole.^222^^-^^225^ However, studies analyzing ischemic events showed conflicting results,^226^^-^^228^, as well as an association between increased ischemic events and the use of clopidogrel + proton pump inhibitors. A subanalysis of the TRITON trial found no association,^229^, and a similar subanalysis of the PLATO trial^230^ found a greater incidence of ischemic events in both the clopidogrel and ticagrelor groups when taken with proton pump inhibitors. More recently, the TRILOGY trial found that among ACS patients who did not undergo myocardial revascularization, proton pump inhibitors did not affect the antiplatelet response in the prasugrel or clopidogrel groups although they were associated with a lower incidence of infarction in the prasugrel group.^228^

COGENT, the only randomized clinical trial that has directly tested such a hypothesis,^231^ evaluated 3761 patients indicated for DAPT for at least 12 months (clopidogrel + omeprazole group vs. a clopidogrel + placebo group). However, this study was stopped early due to funding issues and an insufficient number of patients for follow-up (i.e., a very small number of events were obtained), which significantly compromised its statistical power. In any case, until the study was interrupted, there was no significant difference in the incidence of ischemic events (4.9% in the clopidogrel + omeprazole group vs. 5.7% in the clopidogrel + placebo group, p = 0.96). As expected, a higher incidence of digestive bleeding was observed in the placebo group (2.9% vs. 1.1%, p < 0.001). Thus, considerable research suggests that, in principle, using PPIs (mainly omeprazole) in conjunction with clopidogrel should be avoided. Patients at increased risk of gastrointestinal bleeding (history of gastrointestinal bleeding, diagnosed peptic ulcer, *Helicobacter pylori* infection, age ≥ 65 years, and concomitant use of anticoagulants or steroids) may receive H2-receptor blockers (e.g., ranitidine). If a PPI is necessary, pantoprazole is suggested, whose metabolism via cytochrome P450 is less pronounced.

Therefore, clopidogrel is indicated in NSTE-ACS when there is a moderate to high risk of new ischemic events. Administration consists of a loading dose of 300 mg and a maintenance dose of 75 mg daily. In patients undergoing PCI and with a low risk of bleeding, a loading dose of 600 mg, with a maintenance dose of 150 mg in the first 7 days and 75 mg/day afterward, can be considered. The drug must be used for 12 months, regardless of the treatment received (clinical, percutaneous, or surgical). When elective surgical revascularization is indicated, clopidogrel should be discontinued at least 5 days before the procedure due to the risk of bleeding. Since there is no evidence that adjusting antiplatelet therapy by assessing platelet aggregability is superior to standard antiplatelet therapy, this strategy should only be used in special situations.^232^^-^^233^ Due to clopidogrel's limitations, new drugs have been developed to obtain faster, more effective, andd more consistent platelet aggregation inhibition.

##### 5.2.2. Prasugrel

Prasugrel is a newer generation thienopyridine that fulfills these objectives, due basically to the fact that its metabolism is simpler than clopidogrel, involving only one phase of hepatic metabolism. As a consequence, its active metabolite reaches the plasma peak in just 30 minutes, in addition to having less interaction with medications metabolized by cytochrome P450.^234^

The TRITON trial included 13,608 ACS patients who had not recently used thienopyridine and had defined coronary anatomy (in patients with NSTE-ACS) and scheduled PCI. Of these patients, 74% presented with NSTE-ACS and a moderate or high risk of ischemic/thrombotic complications. Patients who were thrombocytopenic, anemic, or at high risk of bleeding were excluded. The patients were randomized to clopidogrel (300 mg loading and 75 mg daily maintenance) or prasugrel (60 mg loading and 10 mg daily maintenance) after coronary angiography and PCI indication, with a mean follow-up of 14.5 months. The primary efficacy endpoint, cardiovascular death, (re)infarction, and stroke, was 19% lower in the prasugrel group (RR 0.81; 12.1% vs. 9.9%, p < 0.001) than the clopidogrel group. Regarding secondary efficacy outcomes, the prasugrel group had a 24% reduction in AMI (RR 0.76; 9.5% vs. 7.3%, p < 0.001), a 34% reduction in urgent revascularization (RR 0.76; 3.7% vs. 2.5%, p < 0.001) and a 52% reduction in stent thrombosis(RR 0.48; 2.4% vs. 1.1%, p < 0.001).

In the prasugrel group, the safety outcome analysis showed a 32% increase in the incidence of major bleeding (according to the TIMI criterion) unrelated to revascularization surgery (RR 1.32; 1.8% vs. 2.4%, p = 0.03) and a 52% increase in life-threatening bleeding (RR 1.52; 0.9% vs. 1.4%, p = 0.01), in addition to a significant increase in fatal bleeding (RR 4.1; 0.1% vs. 0.4%, p < 0.002).

Overall, prasugrel had lower net benefits than clopidogrel in individuals with previous stroke or TIA (RR 1.54, p = 0.04), was neutral in individuals > 75 years of age or weighing < 60, kg and had higher net benefits in individuals < 75 years of age and weighing > 60 kg without previous stroke or TIA, (RR 0.8; p < 0.001).^154^

Applying the new AMI classification to the TRITON-TIMI results, Morrow et al. demonstrated that prasugrel was more effective than clopidogrel for reducing various types of infarction.^235^ Study sub-analyses showed favorable results to prasugrel in diabetic patients^236^ and in those who underwent myocardial revascularization surgery.^237^

Prasugrel was also evaluated in the TRILOGY trial, which included 9326 patients with NSTE-ACS and an additional risk factor (minimum age of 60, DM, history of AMI, PCI or myocardial revascularization), who did or did not undergo coronary angiography but were specifically indicated for clinical treatment (without myocardial revascularization after the index event for study entry). The patients were randomized to clopidogrel (300 mg loading dose with a 75 mg daily maintenance dose) or prasugrel (30 mg loading dose with a 10 mg daily maintenance dose if aged < 75 years, or 5 mg daily if aged ≥ 75 years or weighing < 60 kg). The patients were followed up for an average of 17 months, and the medication was used for an average of 15 months.^238^

Regarding the primary efficacy endpoint (cardiovascular death, AMI, or stroke), there was no significant difference between the prasugrel and clopidogrel groups (p = 0.21). There were also no significant differences between groups regarding the main safety outcomes (severe or life-threatening bleeding by the GUSTO criterion or severe bleeding by the TIMI criterion).

Finally, in the TRILOGY trial, approximately one-third of the patients underwent platelet aggregation analysis and, despite superior platelet aggregation inhibition in the prasugrel group, a significant association between platelet reactivity and ischemic/thrombotic events could not be determined.^238^

As with clopidogrel, if indicated, prasugrel should, in principle, be taken for 12 months. Prasugrel should be suspended at least 7 days before elective myocardial revascularization.

##### 5.2.3. Ticagrelor

Ticagrelor also blocks ADP-induced platelet aggregation through P2Y_12_ inhibition, although it does not belong to the thienopyridine class. Ticagrelor is a cyclopentyltriazolopyrimidine with a half-life of approximately 12 hours and, unlike thienopyridines, its inhibition of P2Y_12_ receptors is reversible and does not depend on hepatic metabolism to initiate its action. With such characteristics, ticagrelor has a more intense, rapid, and consistent antiplatelet effect than clopidogrel.^239^

In the PLATO trial,^152^ approximately 18,000 patients admitted for intermediate- and high-risk ACS within 24 hours of symptom onset were randomized to receive ticagrelor (180 mg loading dose followed by a maintenance dose of 90 mg every 12 hours) or clopidogrel (300 mg loading dose followed by a maintenance dose of 75 mg daily). Patients who underwent PCI received an additional dose of 90 mg of ticagrelor and could, at the discretion of the investigator, receive an additional dose of 300 mg of clopidogrel. All patients, except when contraindicated, received ASA, and the study medication was continued for 12 months, regardless of the treatment strategy (PCI, surgical revascularization, or clinical treatment alone). In patients indicated for CABG, clopidogrel was discontinued 5 days before the procedure, while ticagrelor was discontinued 24 to 72 hours prior to surgery. The primary efficacy endpoint was the composite of death from vascular causes, non-fatal AMI or stroke in 12 months, while the primary safety endpoint was severe bleeding according to the study's own criterion. In the total sample, the prevalence of NSTE-ACS was approximately 60%, of whom approximately 43% had AMI without ST elevation, and 17% had intermediate- or high-risk UA. The median patient age was 62 years, with 15% older than 75 years. Approximately 64% underwent PCI, and 10% underwent CABG, with the remainder receiving clinical treatment alone. At the end of the study, the ticagrelor group had a significant reduction (16%) in the primary efficacy outcome (RR 0.84; 9.8% vs. 11.7%, p < 0.001). In separate analyses of composite outcome components (main secondary goals), there were significant reductions in the incidence of AMI (RR 0.84; 5.8% vs. 6.9 %, p = 0.005) and death from vascular causes (RR 0.79; 4.0% vs. 5.1%, p < 0.001), with no significant differences in stroke (p = 0.22). Regarding the primary safety outcome, there was no significant difference in the incidence of severe bleeding according to the study's criterion (HR = 1.04; p = 0.43) and the TIMI criterion (HR = 1.03, p = 0.57). Although there was no difference in the incidence of fatal bleeding (HR = 0.87; p = 0.66) or in the need for transfusions (HR = 1; p = 0.96), ticagrelor led to a slight but significant overall increase in fatal intracranial bleeding (0.1 vs. 0.01; p = 0.02) and major bleeding unrelated to CABG (4.5% vs. 3.8%, p = 0.03). There was a higher incidence of other adverse effects in the ticagrelor group. There was a significant increase in the occurrence of dyspnea (RR 1.84; 13.8% vs. 7.8%, p < 0.001), which, in general, was transient and led to drug cessation in less than 1% of patients. It is debated whether this side effect could be explained by increased plasma adenosine levels.^240^^-^^242^ There was also an increase in the incidence of transient bradycardia, with a significant increase in the ventricular pauses > 3 seconds in the first 7 days of medication use (5.8% vs. 3.6%, p = 0.01). However, this effect lost significance after 30 days of medication use (2.1% vs. 1.7%, p = 0.52).^243^ There was no overall difference between the groups regarding the need for pacemaker implantation or the occurrence of syncope or heart block.^243^ There was an increase in creatinine (10% vs. 8%) and uric acid levels (14% vs. 7%), which reverted 1 month after the end of treatment.^244^

The PLATO database provides data on several pre-specified subgroups, including diabetics^245^ chronic kidney disease,^246^ previous stroke,^247^ PPI use,^230^ initial invasive or conservative strategy,^248^ surgical myocardial revascularizations,^249^ the recurrence of events,^250^ cost-effectivenesses,^251^^,^ etc. In summary, ticagrelor appears to be cost-effective, and the sub-analysis results were very close to those of the original publication, which included the entire population. The drug must be suspended at least 3 days prior to elective myocardial revascularization surgery.

More recently, ISAR-REACT 5, a multicenter, non-blinded randomized trial, compared the use of ticagrelor (180 mg at hospital arrival followed by 90 mg twice a day) vs. prasugrel (60 mg in the hemodynamics unit followed by 10 mg or 5 mg for patients ≥ 75 years of age or < 60 kg, respectively) in 4018 patients with ACS indicated for early invasive treatment (41.1% of AMI with supra and 58.9% of NSTE-ACS). The primary outcome (death, AMI, or stroke within 1 year of follow-up) occurred in 184 patients (9.3%) in the ticagrelor group and in 137 patients (6.9%) in the prasugrel group (HR 1.36; 95% CI, 1.09 to 1.70; p = 0.006). Major bleeding, the safety outcome, was similar in both groups: 5.4% in the ticagrelor group vs. 4.8% in the prasugrel group (HR 1.12; 95% CI, 0.83 to 1.51; p = 0.46). However, some study limitations should be pointed out: the study was open (not blinded); 90% of the patient contacts were made by telephone (83%) or letter (7%); neither medication was dispensed by the study (patients acquired them on their own); invasive stratification in NSTE-ACS was determined very quickly, which is not our practice; in the prasugrel group, the primary outcome occurred less frequently (6.9%) than in the TRITON TIMI 38 trial (9.9%) and well below what was estimated for this group (12.9%) in the sample calculation.^252^

#### 5.3. Adjusting Antiplatelet Therapy According to Platelet Aggregation Tests or Genetic Tests

Three randomized clinical studies have sought to demonstrate the clinical impact of antiplatelet therapies in patients with poor response to clopidogrel. The GRAVITAS trial^232^ tested whether a high dose of clopidogrel (600 mg loading dose and 150 mg/day maintenance dose) would prevent cardiovascular events after PCI and drug-eluting stenting better than standard therapy (75 mg/day without a loading dose). Patients were selected according to high platelet reactivity to clopidogrel, which was assessed with the VerifyNow® P2Y_12_ assay. Of the 5429 patients initially assessed, 2214 (40.8%) had platelet hyperreactivity (via ADP) and were included in the study (40% diagnosed with NSTE-ACS). Despite a 22% overall reduction in the number of patients with continued platelet hyperreactivity (P2Y_12_ reaction unit value ≥ 230), treatment with a high dose of clopidogrel did not reduce the risk of death from cardiovascular causes, non-fatal AMI, or stent thrombosis in 6 months after PCI (HR 1.01, 95% CI 0.58 to 1.76, p = 0.97). There was also no increase in severe bleeding between groups. In the ARCTIC study,^233^ 2440 patients with scheduled PCI and drug-eluting stent implantation (27% with NSTE-ACS) were randomized to two different platelet anti-aggregation strategies: a conventional strategy with ASA and P2Y_12_ inhibitors used at normal dosages and a monitored strategy in which the doses were adjusted according to the results of platelet aggregability determined through the VerifyNow® aspirin system and P2Y_12_ assay. Randomization, assessment of platelet reactivity, and intervention, when indicated in the monitored therapy group, were performed prior to the scheduled stent implantation procedure. The incidence of platelet hyperreactivity in clopidogrel patients was 34.5%, and resistance to ASA was observed in 7.6%. In the monitored therapy group, a new platelet function assessment was performed 2 and 4 weeks after stent implantation, and new therapeutic adjustments were made when necessary. In this new assessment, there was a significant reduction (15.6% vs. 34.5% [i.e., approximately 50%], p <0.001) in the number of patients with platelet hyperreactivity at the time of PCI, defined as a P2Y_12_ reaction unit value ≥ 235 or ≤ 15% platelet inhibition compared to control values. However, the primary outcome (all-cause mortality, AMI, stroke, TIA, emergency coronary revascularization, or stent thrombosis within 1 year) was 34.6% in patients who received monitored therapy and 31.1% in those treated conventionally (HR 1.13; 95% CI 0.98 to 1.29; p = 0.10). The rate of serious bleeding events also did not differ significantly between groups. In the ANTARTIC trial, older adults (age ≥ 75 years) undergoing PCI after ACS were randomized to a strategy guided by platelet aggregation assays vs. a conventional strategy. All patients were initially treated with prasugrel 5 mg and, in the guided therapy group, the dose could be increased to 10 mg in case of inadequate platelet inhibition (defined as P2Y_12_ reaction unit ≥ 208) or to 75 mg clopidogrel in case of excessive platelet inhibition (defined as P2Y_12_ reaction unit ≤ 85). The outcome consisted of cardiovascular death, stroke, infarction, urgent revascularization, stent thrombosis, or clinically relevant bleeding in 12 months (HR 1.00, 95% CI 0.78 to 1.29).^253^ Thus, antiplatelet therapy guided by aggregability tests should only be used in selected cases.

Regarding genetic tests, the POPular Genetics trial tested therapy guided by cytochrome 2C19 polymorphism testing. In this study, 2488 patients with ACS were randomized to a standard strategy with ticagrelor or prasugrel vs. a personalized strategy in which patients without polymorphisms (which leads to a loss of 2C19 function and, thus, have normal clopidogrel metabolism) received clopidogrel instead of the other two antiplatelet agents. The personalized strategy was not inferior to the conventional one for the composite outcome (death, infarction, stroke, stent thrombosis, or major bleeding), and it reduced major bleeding (HR 0.78; 95% CI 0.61 to 0.98; p = 0.04).^254^ The TAILOR PCI study included patients undergoing PCI (approximately 80% with ACS and 60% with NSTE-ACS) and assessed a therapy strategy guided by genotyping. This strategy was not superior to conventional treatment for reducing the composite outcome of cardiovascular death, infarction, stroke, stent thrombosis, or severe recurrent ischemia (HR 0.66; 95% CI 0.43-1.02; p = 0.06) in patients carrying the allele that leads to loss of 2C19 function (approximately 35% of included patients).^255^ Further studies, including cost-effectiveness analyses, are needed to confirm these results before a final decision is made about the routine use of genotyping.

**Table:** 

Antiplatelet therapy - Summary of recommendations and evidence		
ASA (162-300 mg loading dose, 81-100 mg/day maintenance dose) should be used in all patients, except when contraindicated, regardless of the treatment strategy, continuing indefinitely.	I	A
Thienopyridines can be used in patients with ASA intolerance.	I	B
Dual antiplatelet therapy can be used for 12 months after an acute event regardless of the initial strategy (clinical, percutaneous, or surgical) unless contraindicated.	I	A
Clopidogrel (300 mg loading dose, 75 mg/day maintenance dose) should be used in addition to ASA for 12 months in intermediate- or high-risk NSTE-ACS patients, in patients with a very high risk of bleeding, with concomitant use of oral anticoagulants, or when prasugrel or ticagrelor is not available.	I	B
Ticagrelor (180 mg loading dose, followed by 90 mg twice/day maintenance dose) should be used in intermediate- or high-risk NSTE-ACS patients, regardless of the subsequent treatment strategy (clinical, surgical, or percutaneous), rather than clopidogrel, for 12 months.	I	B
Prasugrel (loading dose 60 mg, followed by 10 mg daily) should be used rather than clopidogrel in intermediate- or high-risk NSTE-ACS patients with known coronary anatomy treated with PCI, and without risk factors for bleeding (age ≥75 years; with <60 kg; previous stroke or TIA) for 12 months.	I	B
In addition to ASA, clopidogrel (600 mg loading dose, followed by 150 mg daily for 7 days, and a subsequent dose of 75 mg daily) should be used in patients undergoing PCI who have a high risk of ischemic events and a low risk of bleeding.	IIa	B
Ticagrelor, prasugrel, or clopidogrel use should be resumed after coronary bypass surgery, as soon as it is safe.	IIa	B
Platelet aggregation tests or genetic tests (genotyping) should be used in selected cases.	IIb	B
ASA can be combined with other NSAIDs.	III	C
Prasugrel can be used in patients with a history of TIA or previous stroke or in NSTE-ACS patients with unknown cardiac anatomy.	III	B

### 6. Antithrombotic Therapy

#### 6.1. Unfractionated Heparin

UFH was the first injectable anticoagulant to be synthesized. Heparin is a sulfated polysaccharide with a molecular weight range of 3000 to 30,000 Da (mean 15,000 Da). Its action is due to the inactivation of thrombin and activated factor X (factor Xa) through an antithrombin-dependent mechanism. Heparin binds to antithrombin through a high-affinity pentasaccharide and, to inhibit thrombin, it binds to both antithrombin and the coagulation enzyme. By inactivating thrombin, prevents fibrin formation and inhibits thrombin-induced activation of platelets and factors V and VIII. Its variable pharmacological response occurs through independent binding of heparin antithrombin to plasma proteins and through the proteins released by platelets and endothelial cells, causing heparin resistance. For this reason, it is important to monitor thromboplastin activation time. Due to its short half-life, UFH should be administered in a continuous intravenous infusion to ensure stable levels of anticoagulation.^256^^,^^257^

The evidence for using UFH in NSTEMI treatment comes from randomized studies and meta-analyses. In a meta-analysis of six randomized studies, Eikelboom et al. found that UFH or LMWH reduces major cardiovascular outcomes in ACS.^182^ UFH is a widely used anticoagulant in NSTEMI when cardiac catheterization is performed within the first 2 hours of arrival at the hospital, despite consistent evidence of a higher risk of bleeding compared to other antithrombotics.^191^

The proposed therapeutic regimen for UFH should be adjusted to patient body weight: an initial dose of 60 units/kg should be administered intravenously, followed by an infusion of 12 units/kg/hour. Anticoagulation intensity is adjusted by monitoring thromboplastin activation time. The first blood sample must be collected in the third hour of infusion and, subsequently, every 6 hours until the target range is reached, after which it can be collected every 24 hours. The therapeutic range is narrow, so thromboplastin activation time values should be maintained between 1.5 and 2.0 times the control values in seconds.^198^

During PCI, the UFH dose should keep the activated clotting time between 250 and 350 seconds. Dose titration should be guided by body weight. The administration of a bolus of 70-100 IU/kg of UFH is sufficient for adequate anticoagulation. With the concomitant use of GP IIb/IIIa inhibitors, the initial dose should be 60-70 IU/kg.^258^

The most frequent side effects of UFH are major or minor bleeding, which occurs mainly when the clotting time is above the therapeutic range. UFH can trigger an intense immune reaction, heparin-induced thrombocytopenia, a potentially fatal clinical condition that concomitantly induces bleeding and thrombosis.^258^

During life-threatening bleeding or when emergency surgery is required, an antidote must be used to neutralize the anticoagulant effect. Protamine sulfate is used to inactivate the effects of UFH. Since the half-life of UFH is approximately 1 to 1.5 hours, the protamine dose should be based on the total UFH dose administered in the previous 2 to 3 hours. Approximately 1 mg neutralizes 100 IU of UFH. A slow intravenous infusion is recommended to avoid hypotension and bradycardia.^258^

#### 6.2. Low-Molecular-Weight Heparins

During investigations into the structure of conventional heparin (UFH), it was found that its polysaccharide chains can undergo depolymerization through various physical and chemical processes to obtain heterogeneous compounds with lower molecular weight, which are called generic fractionated or LMWH.^256^^,^^257^

LMWH consist of short polysaccharide chains, which result in a more predictable anticoagulant effect than UFH. LMWH has several potential advantages over UFH:^257^

- Greater anti-factor Xa activity than factor IIa activity. More effective inhibition of thrombin production.
- Greater inhibition than UFH in the tissue factor pathway.
- Less frequently induce thrombocytopenia.
- Subcutaneous administration due to their great bioavailability.
- Predictable anticoagulation due to less binding to plasma proteins.
- Plasma level monitoring is unnecessary.

Fraxiparin, dalteparin, and enoxaparin are examples of LMWH that have been tested in ACS. Enoxaparin remains the drug of choice in this context due to the larger body of experimental data. Previous studies have shown that fraxiparin and nadroparin are similar to UFH.

Two studies, TIMI IIB, and ESSENCE have compared enoxaparin with UFH for clinical efficacy and safety in patients with UA and NSTEMI. In summary, they showed for the first time that LMWH (in this case, enoxaparin) was superior to UFH and that enoxaparin provides no additional benefits after the hospitalization phase (TIMI IIB). During the outpatient phase, major bleeding occurred in 1.5% of the group treated with placebo and 2.9% in the group treated with enoxaparin (p = 0.021).^259^^,^^260^ Perhaps, more importantly, a pooled analysis of the two studies showed that the enoxaparin group had significantly fewer “hard” events, i.e., death or (re)infarction, than UFH, with the advantages still evident 1 year after the initial treatment.^261^

The SINERGY study compared enoxaparin to UFH in patients with high-risk NSTE-ACS. A total of 10,027 patients were randomized, and the primary outcome was a composite of cardiovascular death, AMI, stroke, and urgent revascularization. In the enoxaparin arm, the event rate was 14.0% (696/4993), while in the UFH arm, it was 14.5% (722/4985) (RR, 0.96; 95% CI, 0.86-1.06). Regarding safety, there was a statistically significant increase in major bleeding with enoxaparin (9.1% vs. 7.6%, p = 0.008), (TIMI criteria) and a non-significant increase in severe bleeding (GUSTO criteria) (2.7% vs. 2.2%, p = 0.08) and blood transfusions (17.0% vs. 16.0%, p = 0.16). The authors' conclusion was that enoxaparin was neither superior to UFH nor inferior to high-risk treatment for NSTE-ACS).^262^ Enoxaparin is a safe and effective alternative to UFH. The advantage of subcutaneous use and the fact that it does not require routine anticoagulation monitoring should be considered in light of the modest excess of major bleeding. Another consideration from an unspecified analysis was that switching classes of anticoagulants during the hospital phase of patients with ACS should be avoided.^262^

A 1 mg/kg dose of enoxaparin should be administered subcutaneously twice a day. Beginning at 75 years of age, the dose should be reduced to 0.75 mg/kg twice a day. In patients with an estimated glomerular filtration rate ≤ 30 mL/min/1.73 m^²^, the dose should be reduced by half, and then 1 mg/kg should be administered subcutaneously once a day. When the estimated glomerular filtration rate reaches 15 mL/min/1.73 m^²^, enoxaparin should be avoided. UFH is an alternative for this condition since its metabolism is exclusively hepatic. In patients pretreated with enoxaparin, using additional enoxaparin during PCI is not recommended if the last subcutaneous enoxaparin injection was administered 8 hours before the procedure. An additional 0.3 mg/kg intravenous bolus is recommended if the last subcutaneous dose of enoxaparin was administered ≥ 8 hours before angioplasty.^263^

#### 6.3. Fondaparinux

Fondaparinux is a synthetic pentasaccharide that consists of a sequence of sugars (A B C D F) - the smallest sequence capable of directly binding to thrombin. This discovery was a milestone in the pharmacological development of heparins. It provided the tools to understand the biology of this class of drugs and advance its pharmacology. The development of fondaparinux showed that selective and direct inhibition of factor Xa promotes an important antithrombotic effect, which opened the way for new direct oral anticoagulants.^264^ Synthetic pentasaccharide, which selectively binds to thrombin, leads to factor Xa inhibition. Due to its discreet interaction with plasma components, it has predictable anticoagulant action and little individual variability. It has good bioavailability, which favors subcutaneous administration. It reaches its plasma peak quickly (2 hours) and has a long half-life (17 hours), allowing the use of a single daily dose. Fondaparinux is eliminated exclusively by the kidneys, which requires renal monitoring. When the estimated glomerular filtration rate is < 30/mL/1.73 m^²^, its use should be suspended.^265^

The OASIS-5 trial tested the efficacy and safety of fondaparinux vs. enoxaparin in patients with NSTE-ACS. It was designed to assess whether fondaparinux would continue the anti-ischemic benefits of enoxaparin while reducing bleeding in patients with ACS. A total of 20,078 patients with NSTE-ACS who received fondaparinux (2.5 mg/day) or enoxaparin (1 mg/kg twice a day) were randomized for an average exposure of 6 days. The primary composite efficacy outcome was cardiovascular death, AMI, or refractory ischemia on a ninth day, while major bleeding was the primary safety outcome. A combination of these outcomes was considered the net clinical benefit. The patients were followed for up to 6 months, and the composite efficacy outcome was similar in both groups (579 events with fondaparinux [5.8%] vs. 573 with enoxaparin [5.7%]; RR: 1.01 in the fondaparinux group; 95 % CI 0.90-1.13; p for non-inferiority = 0.007).^194^ Further sub-analyses of the study were published, the most important of which analyzed the 12,715 patients who underwent angiography during hospitalization (6238 treated with PCI). During the course of OASIS 5 (after reports of catheter thrombosis from some centers), the protocol was amended, requiring the hemodynamic catheter to be washed with 200 IU of UFH before the procedure. Among the patients who underwent PCI, the efficacy of fondaparinux was similar to enoxaparin for the primary outcome in 9 days (6.2% vs. 6.3%; RR 1.09; p = 0.79), with a lower incidence of severe bleeding (10.4% vs. 8.2%; RR = 0.78; p = 0.004). However, there was a higher incidence of catheter thrombosis (0.4% vs. 0.9%), and this complication was associated with higher rates of stroke and AMI.^266^^,^^267^

**Table:** 

Antithrombotic therapy - Summary of recommendations and evidence		
UFH can be used routinely.	I	A
LMWH can be used routinely.	I	A
Subcutaneous fondaparinux 2.5 mg can be used once/day for 8 days or until hospital discharge.	I	B
For patients using fondaparinux, administer UFH 85 lU/kg intravenously at the time of PCI, or 60 lU/kg for those receiving GP llb/llla inhibitors.	I	B
Enoxaparin is preferential to UFH unless myocardial revascularization surgery is planned within 24 h.	IIa	A
Consider discontinuing anticoagulation after PCI unless there is an indication to maintain it.	IIa	C
Heparin cross-over (UFH and enoxaparin).	III	B

### 7. Associating Oral Antiplatelets and Anticoagulants in Special Circumstances

In recent years, four DOACs have been studied regarding atrial fibrillation in patients undergoing angioplasty and have become viable alternatives to warfarin.

Clinical studies that tested DOACs in patients with CAD (chronic and acute) associated with atrial fibrillation found the following results in comparison to triple therapy (ASA + clopidogrel + warfarin):

In the PIONEER AF-PCI trial, 15 mg rivaroxaban (reduced to 10 mg in patients with creatinine clearance between 30-50 mL/min) + P2Y_12_ inhibitors (clopidogrel/ticagrelor) for 12 months was associated with significantly lower bleeding rates (primary goal), and the efficacy component (cardiovascular death, AMI, and stroke) was similar (secondary outcome).^268^

In the REDUAL PCI trial, dabigatran 110 mg or 150 mg twice a day + P2Y_12_ inhibitors (clopidogrel/ticagrelor) for 12 months was associated with significantly lower bleeding rates (primary outcome), with a significant p-value for non-inferiority in both dabigatran groups but a significant p-value for superiority only in the 110 mg dabigatran group; dual therapy (both dabigatran groups) was similar to triple therapy for the composite outcome of thromboembolic events.^269^

• In the AUGUSTUS trial, apixaban 5 mg twice a day (or 2.5 mg twice a day was used if two of the following three conditions were present: age > 80 years, weight < 60 kg or serum creatinine > 1.5 mg/dl) + P2Y_12_ inhibitors (clopidogrel) was associated with a lower bleeding rate (primary outcome) and a lower rate of death or hospitalization (secondary outcome), with no significant difference in the incidence of ischemic events. It should be pointed out that ACS patients without PCI were also included in the study.^270^

• In the ENTRUST AF-PCI trial, edoxaban 60 mg in association with P2Y_12_ inhibitors (n = 751) was used in one group, while warfarin was combined with DAPT (ASA + P2Y_12_ inhibitors) was used in the other group, totaling 1,506 patients. The primary outcome was major bleeding, (HR = 0.83; 95% CI 0.65-1.05; p = 0.0010 for non-inferiority and p = 0.1154 for superiority). There was no significant difference for the secondary outcome (cardiovascular death, stroke, systemic embolism, AMI, or stent thrombosis).^271^

In these studies, about half of the patients had ACS, and clopidogrel was much more frequently used than ticagrelor as a P2Y_12_ inhibitor. The safety outcome (bleeding) was always the primary objective. Although they had different designs, these studies showed that DOACs were safe compared to warfarin when associated with antiplatelet agents. However, there were no significant differences in ischemic (i.e., secondary) outcomes.^272^ The total time for triple therapy, either with DOACs or warfarin, and for suspending ASA, should be determined according to the risk of ischemic and hemorrhagic events ^273^

### 8. Hypolipidemic Drugs

Several studies have demonstrated the benefits of cholesterol-lowering therapies in secondary prevention.^274^^,^^275^ In high-risk patients, the benefits are independent of baseline LDL-C levels.^276^ A study by Dondo et al. with more than 389,000 NSTE-ACS patients and a mean follow-up of 2.2 years found that non-adherence to statin treatment was one of the most important factors in reduced survival. If the guidelines were applied and followed correctly,i.e., adherence to statin use, coronary angiography when indicated, cardiovascular rehabilitation, and smoking cessation, 28.9% of the associated deaths could be prevented.^277^

Although lipid targets vary in different guidelines, a more rigorous strategy to reduce LDL levels has recently been recommended. The 2017 Brazilian Guideline on Dyslipidemia and the Prevention of Atherosclerosis recommended LDL levels < 50 mg/dL and non-HDL levels < 80 mg/dL in very high-risk patients.^278^ When large doses of statins cannot be tolerated, association with ezetimibe is an alternative.^279^

It is assumed that lipid-lowering therapy should ideally begin during hospitalization, provided there is no contraindication. The SECURE-PCI trial evaluated an 80 mg loading dose of atorvastatin before coronary angiography in 4,191 ACS patients indicated for invasive strategy. The loading dose did not reduce major cardiovascular outcomes in 30 days compared to the placebo (HR 0.88; 95% CI 0.69 to 1.11; p = 0.27), although the subgroup of patients who underwent PCI may have benefitted (HR 0.72; 95% CI 0.54 to 0.96; p = 0.02).^280^

**Table:** 

Hypolipidemic drugs - Summary of recommendations and evidence		
Begin early treatment with a high dose of statin in all patients, regardless of LDL levels, as long as there are no contraindications.	1	A
Associate ezetimibe in patients using the maximum tolerated statin dose if LDL levels do not reach the established goals.	1	A

### 9. Renin-Angiotensin-Aldosterone System Inhibitors

This group of drugs is very important for treating high blood pressure, HF, and some groups of patients with CAD. There is no conclusive evidence that the early use of these drugs benefits patients with NSTE-ACS, although some studies have suggested that they may be useful in the chronic phase after an acute episode. The HOPE trial^281^ included patients at high risk of cardiovascular events, often with major atherosclerotic arterial disease (usually reaching the coronary region). Regardless of the stage, ramipril (target dose of 10 mg/day) had long-term benefits: in 5 years of follow-up, there was a 26% reduction in the relative risk of death (p < 0.001), a 20% reduction in infarction, (p < 0.001), and a 32% reduction in stroke (p < 0.001). Similar results have also been demonstrated for perindopril in chronic coronary disease patients.^282^ The benefits are greater in patients with LV dysfunction and previous AMI, including improved cardiac remodeling and LV function and decreased progression to HF.^283^

The aim of treatment is to reach the highest tolerable dose of medications: captopril 50 mg every 8 hours (which can later be replaced by ramipril 10 mg/day or enalapril 20 mg/day every 12 hours), losartan 50 mg every 12 hours, or valsartan 320 mg/day.

Regarding aldosterone antagonists, the EPHESUS trial found lower mortality with eplerenone (not available in Brazil) in patients with AMI (with or without ST elevation) and LV dysfunction who showed symptoms of HF or DM (RR 0.85; 95% CI 0.75-0.96; p = 0.008). The benefits were already evident in the first 30 days of treatment.^284^^,^^285^ The RALES trial had previously found lower mortality in patients with chronic HF and LV ejection fraction (LVEF) ≤ 35%.^286^ Thus, a target dose of 25 mg of spironolactone (an aldosterone antagonist available in Brazil) once a day (which can be increased to 50 mg after 8 weeks of treatment if there are signs of HF progression without hyperkalemia). Aldosterone blockers are contraindicated in patients with significant renal dysfunction (Cr > 2.5 mg/dL) or potassium levels > 5.5 mEq/L. Potassium levels and renal function must be carefully monitored, especially when aldosterone blockers are used concomitantly with angiotensin-converting enzyme (ACE) inhibitors or angiotensin receptor blockers.

**Table:** 

Renin-angiotensin-aldosterone system inhibitors - Summary of recommendations and evidence		
Administer angiotensin-converting enzyme (ACE) inhibitors to intermediate- and high-risk patients with LV dysfunction, high blood pressure, or DM.	I	A
Administer angiotensin II receptor blockers to intermediate- and high-risk patients with contraindications to ACE inhibitors.	I	C
Use spironolactone in patients with NSTE-ACS, LVEF ≤ 35%, HF symptoms, or a history of DM.	I	C
Administer ACE inhibitors to all intermediate- and high-risk patients.	IIb	B

### 10. Complementary Methods of Risk Stratification

In patients with ACS, risk stratification must be a continuous process, from the initial clinical evaluation to the previously discussed subsidiary exams to the complementary methods described below.

### 11. Non-Invasive Tests for Ischemia Diagnosis and Prognostic Assessment

Functional tests are indicated for assessing myocardial ischemia and, in some cases, are essential for decision-making, especially for the diagnosis and prognosis of intermediate- and high-risk patients. Non-invasive prognostic methods are more common in the conservative therapeutic strategy.

#### 11.1. Exercise Testing

During the hospitalization phase, ET is used to determine the residual degree of ischemia and assess cardiac performance. Residual ischemia is estimated by ST-segment changes during exercise or the recovery phase and by anginal pain. Cardiac performance is assessed through blood pressure and double product. Cardiac performance and autonomic response related to heart rate reduction during the recovery phase have shown a better correlation with mortality than other parameters and have a very high value (98% to 100%), although their positive predictive value is modest (approximately 50%), with abnormal ET results being uncommon in patients indicated for this stratified procedure in chest pain units.^287^^-^^289^

In multivariate regression analysis, the main independent predictors of event-free survival (death and AMI) in 1 year were the number of leads with depressed ST-segment and the maximum load achieved. Regarding safety, there is a 0.5% incidence of complications (death or AMI) in stabilized patients within 24 hours of ET.^290^^-^^292^

ET is recommended for estimating prognosis and assisting clinical decision-making in intermediate-risk patients 24 to 48 hours after complete clinical stabilization (hemodynamic stability, no active clinical or electrocardiographic ischemia, no new Q waves, no clinical signs of HF, and normal myocardial injury markers), provided the patient is able to exercise.^51^^,^^198^

Thus, ET as an early strategy (< 48 hours) is formally contraindicated in high-risk patients. However, 48 hours after full stabilization of the clinical condition, even during hospitalization, ET may be indicated in patients undergoing coronary angiography, when a functional assessment of a known lesion is required or the risk level must be determined before hospital discharge, as well as to properly guide cardiac rehabilitation programs.^293^^,^^294^

**Table:** 

Exercise testing - Summary of recommendations and evidence		
ET can be performed in intermediate-risk patients 24 to 48 h after complete clinical stabilization, provided that there are no active ischemic symptoms, no signs of HF or hemodynamic impairment, resting ECG without ischemia, and normal myocardial necrosis markers.	I	B
ET can be performed in high-risk patients 48 h after clinical stabilization.	IIb	C
ET can be performed in high-risk patients 48 h before clinical stabilization.	III	C

#### 11.2. Echocardiographic Methods (Ischemia, Viability, Stress, Contrast Microbubbles, Strain, etc.)

##### 11.2.1. Transthoracic Echocardiogram

A new analysis of LV contractile changes may, in fact, help determine the diagnosis and prognosis of ACS.^295^^-^^299^ The wall motion score index is the reference parameter for expressing LV segmental function, and its normal value is 1 (the LV is divided into 16 or 17 segments that are classified based on systolic thickening); values between 1 and 1.6 show a mild contractile change, while wall motion score index values > 1.6 indicate greater involvement and worse prognosis. Obviously, a lack of segmental contractility changes in resting transthoracic echocardiogram results does not exclude CAD.^300^

It is worth remembering that ventricular function assessment by transthoracic echocardiogram during the acute phase of ischemia could be compromised by myocardial stunning. After 2 weeks, marked improvement in ventricular function may occur.^301^

Through new technologies in echocardiography, such as 2D speckle-tracking, it has become possible, by analyzing the global longitudinal strain (GLS) of the LV walls, to obtain an early diagnosis of ischemic changes in patients with troponin changes but no ECG or resting echocardiogram changes (25% to 75% of patients with ACS have normal echocardiogram results).^302^

GLS (when < 16.5%) can complement existing diagnostic algorithms and act as an early marker of ischemia.^303^ A recent publication proposes that GLS can help determine candidates for invasive strategy.^304^ Small centers have routinely used GLS in the emergency department, and changes in GLS patterns (< 18%) have been reported even before changes in troponin.^305^

In special circumstances with a suboptimal acoustic window, a transthoracic echocardiogram should be complemented with microbubble contrast echocardiography to better determine the endocardial borders or evaluate any myocardial perfusion defects.^306^

**Table:** 

Transthoracic echocardiogram - Summary of recommendations and evidence		
Use to assess global and segmental ventricular function.	I	C
Use in a differential diagnosis of alternative causes of chest pain: severe aortic stenosis, hypertrophic cardiomyopathy, pulmonary embolism, aortic dissection, pericarditis, and cardiac tumors.	I	C
Use in chest pain with hemodynamic instability and suspected cardiac origin.	I	C
Use for suspected mechanical complications in myocardial infarction: LV aneurysm, free wall or papillary muscle rupture, interventricular communication, and pericardial effusion.	1	C
Calculate GLS using speckle tracking as an adjunct to existing diagnostic and risk classification algorithms in patients with suspected coronary disease.	IIa	B

##### 11.2.2. Stress Echocardiography

Stress echocardiography can detect transient regional contraction abnormalities, which are indicative of exercise-induced and pharmacologically-induced ischemia.^307^ The stress echocardiogram is an independent predictor of cardiovascular death; adding value to other methods, it can prevent cardiac catheterization. It is recommended for risk stratification in chest pain units, especially when the diagnosis cannot be determined through ECG and the ET is submaximal, cannot be performed, or is inconclusive, given that the pain is relieved for at least 24 hours in low- to moderate-risk patients and no ischemic changes or necrosis markers are evident in the ECG.^307^^,^^308^

An improved segmental contraction in dyssynergic areas after initial dobutamine doses (5 to 10 g/kg/min) identifies myocardial viability in regions "stunned" by prior ischemia.^307^^-^^309^

**Table:** 

Assessment by stress echocardiogram - Summary of recommendations and evidence		
Use in patients with unstable angina or clinically controlled low-risk NSTE-ACS* before deciding on an invasive strategy.	IIa	A
Use to assess the functional significance of moderate coronary obstruction on angiography, as long as a result interferes with management.	IIa	C
Use for risk stratification after uncomplicated myocardial infarction.	IIa	A
Use to investigate suspected microvascular disease to determine whether segmental changes occur in conjunction with angina and ECG changes.	IIa	C
Use strain and strain rate parameters derived from speckle tracking as an adjunct tool to the wall motion score index for diagnosis and/or prognosis of acute coronary disease.	IIa	B
Use in high-risk unstable angina or acute myocardial infarction.	III	C

* Without recurrent angina, signs of HF, changes in initial/serial ECG and with normal troponin; typical anginal pain with abnormal ECG or functional test with normal cardiac catheterization.

##### 11.2.3. Echocardiographic Assessment of Myocardial Perfusion

In patients with acute chest pain and non-diagnostic ECG, contrast echocardiography increases sensitivity for ACS diagnosis.^310^ Patients with normal perfusion and myocardial function at rest have a good prognosis, while perfusion defects at rest indicate a high-risk ACS subgroup.^306^^,^^311^

Echocardiographic contrast agents are solutions that contain gas microbubbles with size of red blood cells, whose interface with the liquid medium is highly refractive, which improves the echocardiographic signal of the medium. Myocardial microcirculation can be assessed at the bedside with this method since microbubbles are microvascular markers that behave like red blood cells, i.e.,i.e.hey do not reach areas of microvascular obstruction. Thus, infarcted areas do not show this contrast and are easily detected by echocardiography.^306^ Through this type of echocardiography, changes in LV segmental contraction and myocardial perfusion are obtained simultaneously and instantly.^312^

Echocardiographic contrast allows a better definition of the endocardial borders, allowing an adequate assessment of myocardial thickening and global and segmental contractile functions of the LV at rest and under stress. In addition, contrast agents allow more accurate measurement of ventricular volumes and LVEF, especially in cases of suboptimal images, and have proven useful in determining anatomical changes.^306^^,^^313^

Extensive transient regional perfusion defects during stress are indicative of severe CAD. An improved segmental contraction in dyssynergic areas with perfusion during the initial dobutamine infusion (5 to 10 *µ*g/kg/min) identifies myocardial viability in regions "stunned" by prior ischemia.

In summary, in patients with chest pain treated at the emergency department, contrast echocardiography at rest adds diagnostic and prognostic data. Complete filling of the myocardium in contrast (perfusion) within 4 seconds identifies patients with normal motility and perfusion and, therefore, of low risk. Normal perfusion but a segmental change in wall movement indicates a stunned myocardium, while a fixed perfusion defect shows that the entire area is at risk during acute ischemic injury.^314^^,^^315^ In patients with AMI, stress echocardiography with myocardial contrast not only identifies the areas of infarction or stunning at rest but can identify ischemic areas at a distance by analyzing the transient changes in myocardial motility and perfusion.

###### 11.2.3.1. Echocardiographic Assessment of Myocardial Perfusion - Summary of Recommendations and Evidence

**Table:** 

Assessment of Myocardial perfusion by Echocardiogram - Summary of Recommendations and Evidence		
Use contrast transthoracic echocardiography to improve the Doppler signal in patients with suboptimal imaging or to determine the endocardial borders during stress echocardiography in patients with suboptimal images at rest.	IIa	B
Use stress echocardiogram with microbubbles in intermediate-risk patients with unclear ET results.	IIb	B
Use stress echocardiogram with microbubbles in high-risk patients.	III	C

#### 11.3. Nuclear Cardiology Methods

MPS is fundamental when an ET cannot be performed and when there are difficulties interpreting the exercise ECG. One advantage of MPS is that it is among the most widely used non-invasive techniques for low-risk UA patients. It allows assessment of patients who cannot undergo physical exertion through pharmacological stress and has a better correlation with anatomical data than ET.

MPS and radionuclide ventriculography have great diagnostic and prognostic value in acute and chronic coronary diseases.^316^^-^^318^ One of the main advantages of MPS is that it can be performed early in ACS and has a wide safety margin, using vasodilating agents such as dipyridamole and adenosine. For this application, MPS was found to be superior to other tests. It should also be pointed out that tomographic scintigraphy can be synchronized with ECG (gated SPECT) to assess regional systolic function and measure ventricular ejection fraction with a single exam.^319^ Studies have consistently demonstrated that patients diagnosed with UA who have normal findings belong to a subgroup with a markedly reduced risk of serious events (about 1% in 1 year), while reversible defects indicate an unfavorable prognosis, with an event rate of approximately 20% in 1 year.^320^^-^^323^

Positron emission tomography, a non-invasive technique for assessing obstructive coronary disease, has a very high accuracy for detecting myocardial ischemia and myocardial viability. It has been found useful for diagnostic and prognostic assessment of CAD. It also provides a very accurate assessment of coronary flow reserve by absolute quantification. This information can be useful for assessing infarction in patients without significant coronary obstructions (MINOCA).^324^^,^^325^ It is difficult to obtain radiotracers (rubidium and ammonia) in Brazil, although new gamma chamber detectors of cadmium-zinc-tellurium can also quantify flow reserve using technetium tracers.^326^

**Table:** 

Myocardial perfusion scintigraphy - Summary of recommendations and evidence		
Use MPS in intermediate-risk patients with unclear ET results or who cannot undergo ET.	1	B
Use MPS to identify the presence/extent of ischemia in patients who cannot undergo catheterization or when its results are insufficient to determine treatment.	1	B
Use MPS after catheterization to identify which artery is related to the event (region to be revascularized) and/or for complementary risk stratification.	1	A
Use MPS in patients with ventricular dyssynergic areas when it is necessary to determine the viability of the myocardium to guide treatment.	1	A
Use MPS as the first option for ischemia assessment, even in patients for whom ET is feasible and interpretable.	IIb	B
Use nuclear angiocardiography in intermediate- and high-risk patients to identify right ventricular involvement.	IIa	C
Use MPS in high-risk patients before the first 48 h of stabilization.	III	C

#### 11.4. Cardiovascular Magnetic Resonance

Cardiovascular magnetic resonance (CMR) is a very useful method of morphological and functional cardiac assessment, providing important diagnostic and prognostic information about the ischemic disease and nonischemic cardiomyopathies. The test can provide accurate information about morphological aspects of the heart, quantify volumes, mass, and global and regional ventricular function, assess myocardial ischemia (by analyzing the segmental contractility under dobutamine stress without using contrast medium or by stress myocardial perfusion with vasodilators, such as dipyridamole and adenosine, and gadolinium-based contrast), as well as assess myocardial fibrosis/necrosis with the myocardial enhancement technique.^327^ All of this information is provided in an integrated manner through a single exam. The method also allows, by combining these and other forms of image acquisition, visualization of the pericardium and the great vessels, as well as flow and valve function analysis.

Several clinical studies and meta-analyses have shown that CMR is a highly accurate method for detecting myocardial ischemia, even compared to stress echocardiography,^328^ myocardial scintigraphy,^329^^,^^330^ and FFR.^331^

The use of delayed enhancement imaging to assess myocardial viability has also been studied and validated in ACS. This method provides important diagnostic and prognostic information and is considered the gold standard for assessing myocardial viability and detecting infarctions.^332^^-^^336^ Uni- and multicenter studies have shown that CMR is an extremely sensitive technique for detecting and localizing infarction, even small subendocardial infarctions,^337^^-^^339^ which have prognostic importance, perhaps due to their frequent association with critical coronary stenosis and the fact that they may go unnoticed both in clinical evaluation and other diagnostic tests.^340^^-^^342^ In addition, the infarcted area, which is measured by CMR, has a great prognostic impact independent of other clinical factors; some studies have found it more important than LVEF itself.^343^^,^^344^ When techniques for identifying areas of edema are associated with the measurement of areas of delayed enhancement, the rate of myocardial salvage can also be obtained, which has prognostic value, especially in patients undergoing thrombolytic treatment.^345^

Delayed enhancement imaging also allows detection of hypoenhanced (dark) areas in the midst of the hyperenhanced area (infarction), which are related to microvascular obstruction (the no-reflow phenomenon), adding prognostic information about this population.^343^^-^^347^

In addition to these applications, CMR is very useful for differentiating ischemic from nonischemic cardiomyopathies;^327^^,^^348^ it is used to diagnose myocarditis^349^^,^^350^ and Takotsubo syndrome.^351^^,^^352^ Furthermore, when there are elevated myocardial necrosis biomarkers and “normal” coronariography, CMR can confirm the presence of infarction, which could be related to spasm, thrombophilic syndromes, etc. Other differential diagnoses in which CMR can help include LV hypertrophy (primary of hypertrophic cardiomyopathy or secondary) and isolated acute pericarditis without associated myocarditis.

Due to its sensitivity for finding areas of infarction and diagnosing other cardiac abnormalities, CMR has been recommended to assess patients with infarction and those without significant coronary obstructions (MINOCA). The examination allows the correct differentiation between inflammatory processes and actual ischemic necrosis, even without significant atheroma plaque, and can provide fundamental data for managing these cases. Such an approach may be particularly relevant for women, who have a higher incidence of MINOCA.^353^

In cases of confirmed CAD, CMR can also help define the infarction-related coronary anatomy when it is not clear in catheterization, such as in patients with severe multivessel diseaseand non-specific ECG. In these patients, CMR can also differentiate between old and recent infarctions that are related to the current condition. Finally, CMR can provide high-resolution images for assessing post-infarction mechanical complications, such as LV rupture, mitral regurgitation, an extension of the infarction to the right ventricle, and interventricular communication.^340^^,^^354^

**Table:** 

Cardiovascular Magnetic Resonance - Summary of recommendations and evidence		
CMR can be used to assess ventricular function, the presence/extent of necros, is, and myocardial viability.	I	A
CMR can be used to search for possible mechanical changes.	I	A
CMR can be used to assess patients with a clinical presentation of ACS but without significant coronary obstructions.	I	A
CMR can be used in the differential diagnosis of patients whose clinical picture is compatible with acute CAD but whose ECG shows nonspecific changes and negative biochemical markers of necrosis.	IIa	B
Use CMR as an adjunct in ACS diagnosis, especially in patients with an intermediate or high probability of ACS.	IIb	B

### 12. Cardiac Catheterization and Intravascular Coronary Imaging (fractional flow reserve, intravascular ultrasound, optical coherence tomography)

Coronary angiography, which is commonly used to diagnose and determine treatment for patients with NSTEMI, may not show the culprit lesion in UA and NSTEMI. This is due to the fact that the test is not 100% sensitive to plaque ruptures.^355^ The triggering factors for ACS can range from obstructive atherosclerotic disease (which may be incipient or with or without ruptured plaque) to spasm, embolism, intramural coronary hematoma, spontaneous coronary dissection, and aortic dissection.

In a recent meta-analysis, Barcarawi et al. demonstrated that for patients with NSTE-ACS, an early invasive strategy resulted in a lower incidence of major cardiovascular events (defined by each included study) than late invasive strategy (RR 0, 65, 95% CI 0.49-0.87; p = 0.003), although the benefits persisted only in patients with GRACE scores > 140.^356^ However, there were no significant differences in all-cause mortality, cardiovascular mortality, myocardial infarction or hemorrhagic events between the groups. Early invasive treatment was also associated with a lower risk of ischemic outcomes in the SWEDEHEART study.^357^

The therapeutic strategy, especially with respect to invasive evaluation, depends not only on the anatomical and functional diagnosis but on the comorbidities, clinical presentation, frailty, cognitive status, life expectancy, and, especially, risk stratification, because “very high risk” patients must be evaluated in less than 2 hours and “intermediate risk” patients must be evaluated within 72 hours, as is presented in Table 2.1.^358^

**Table 2.1:** NSTE-ACS treatment strategy according to initial risk stratification

Invasive evaluation in NSTE-ACS		
Very high risk	High risk	Intermediate risk
Hemodynamic instability or cardiogenic shock	Positive troponin	DM or kidney failure
Recurrent or persistent angina refractory to clinical treatment	Dynamic ST-T change	HF or LVEF < 40%
Malignant ventricular arrhythmia or CRA	GRACE > 140	Post-AMI angina
Mechanical complications		Previous PCI or MRS
Acute HF		GRACE 109-140 or recurrent symptoms or positive functional test
Recurrent dynamic ST/T changes		
		
Immediate invasiveHF	Early invasive(less than 24 h)AI	Invasive(less than 72 h)AI

Source: adapted from Neumann et al.^358^

AI: aortic insufficiency; AMI: acute myocardial infarction; CRA: cardiorespiratory arrest; DM: diabetes mellitus; GRACE: Global Registry of Acute Coronary Events; HF: heart failure; LVEF: left ventricular ejection fraction; MRS: myocardial revascularization surgery; PCI: percutaneous coronary intervention

The invasive strategy allows diagnosis of underlying CAD, identifies the lesion, guides treatment for antithrombotic drugs, and determines the coronary anatomy for PCI or CABG surgery, since up to 40% of NSTE-ACS patients have plaques of complex morphology and up to 25% have acute coronary occlusion.

The European Society of Cardiology and the European Association of Cardiothoracic Surgery consider FFR to be the diagnostic standard for functional assessment of injury severity in patients with intermediate-grade stenosis (typically around 40% to 50%) without evidence of ischemia in non-invasive tests or in patients with multivessel disease. This guideline includes the instantaneous wave-free ratio (iFR), a new measure that does not require adenosine-induced hyperemia and has a class IA recommendation like FFR.

For left main coronary disease, functional assessment by FFR or iFR can be technically complex, and evidence supporting their use in this condition is scarce. Consequently, intravascular ultrasound is a class IIaB recommendation, and revascularization should be excluded when the minimum luminal area is > 6 mm^2^. For all lesions outside this area, functional assessment is preferable to intracoronary imaging. Intravascular ultrasound and optical coherence tomography (OCT) are recommended to optimize stenting (class IIaB).

FFR assessment requires the administration of adenosine to obtain maximum and stable hyperemia, unlike iFR, which assesses rest indexes derived only from resting gradients, i.e., the ratio of distal coronary pressure to aortic pressure (Pd/Pa). In the iFR-SWEDEHEART trial, which evaluated iFR-guided revascularization, 17.5% of the patients had ACS at the time of the assessment and, this strategy was not inferior to FFR-guided revascularization regarding the rate of major cardiac events and events up to 12 months after the procedure.^359^

The DEFINE–FLAIR trial,^360^^,^ which evaluated 2,492 patients with CAD, found that iFR-guided coronary revascularization was no less efficacious than FFR-guided revascularization regarding the risk of major adverse cardiac events in 1 year, with the added benefit that iFR was more economical than FFR. The cut-off points for defining a lesion as hemodynamically significant were: iFR ≤ 0.89 and FFR ≤ 0.8.

OCT uses infrared light to provide high-resolution in vivo cross-sectional images of the coronary artery. This imaging technique allows a detailed assessment of plaque morphology in patients with ACS and can clarify the underlying mechanisms, including plaque rupture or erosion and calcification. Therefore, OCT is useful for optimizing PCI and evaluating vascular response to coronary intervention and pharmacological therapy.^361^

By identifying the thrombus and plaque rupture or erosion, OCT can help clarify the nature of lesions and determine the underlying mechanisms of ACS, especially when the lesions are ambiguous on angiography.^362^

The DOCTORS trial assessed ACS patients referred for angioplasty and stenting in whom a single localized lesion in the culprit artery was considered responsible for ACS.^363^ This trial was designed to evaluate the effectiveness of OCT in optimizing percutaneous coronary intervention among patients with NSTE-ACS, demonstrating that stent underexpansion was common (approximately 42%) and an independent predictor of adverse outcomes. The authors concluded that an intervention with OCT results in greater functional benefits according to post-intervention FFR assessment than an intervention guided by routine angiography.

Although long-term data demonstrate that physiological assessment has better results in CAD treatment, FFR and iFR are underused in current practice.^364^ Nevertheless, advances in alternative approaches to FFR, including non-invasive coronary computed tomography, invasive angiography, and OCT, are already being guided by artificial intelligence algorithms and robust tools that allow detailed pre-procedure intervention.

**Table:** 

Hemodynamic and coronary angiographic assessment - Summary of recommendations and evidence		
Immediate cardiac catheterization should be performed in very high-risk patients	I	A
In high-risk patients, early cardiac catheterization (within 24 h) should be performed.	I	A
In intermediate-risk patients, those with recurrent symptoms, or those with a positive noninvasive test for ischemia, perform cardiac catheterization within 72 h.	I	C

### 13. Myocardial Revascularization (Myocardial Revascularization Surgery and Percutaneous Coronary Intervention)

The difference between UA and NSTEMI treatment in chronic CAD is a quick decision to indicate revascularization, thus avoiding cardiovascular complications.^365^^-^^367^ Risk stratification is essential for the proper choice of conservative or invasive treatment. The most common risk scores are TIMI and GRACE.^28^^,^^368^

Coronary angiography determines the treatment strategy; revascularization should be immediate in cases of refractory angina and electrical or hemodynamic instability. Earlier approaches also have favorable outcomes and are indicated in high-risk patients.^28^^,^^368^^-^^370^ In a cohort of 363,500 diabetic patients with NSTEMI, an early invasive assessment strategy was used in 164,740 patients (45.3%). In an analysis adjusted for propensity score, 21,681 diabetics were paired in each arm. Lower in-hospital mortality was associated with the invasive strategy in NSTEMI but not in UA (2.2% vs. 3.8%; RR 0.57, 95% CI; 0.50-0.63, p < 0.01).^371^

Invasive strategies include angioplasty or myocardial revascularization surgery.^372^ The SYNTAX Score is a fundamental tool for making this decision based on coronary anatomy.^373^ Patients with SYNTAX scores > 22 (intermediate or high) have the greatest long-term benefits from surgical revascularization.^374^^,^^375^

Approximately 5% to 10% of patients with NSTE-ACS require CABG.^376^ This is a challenging subgroup due to its high-risk characteristics compared to patients undergoing elective myocardial revascularization.^377^

In patients with ongoing ischemia or hemodynamic instability who are indicated for CABG, surgery should be performed as soon as possible and should not be delayed as a consequence of antiplatelet treatment.

Since data from randomized trials are lacking, the ideal time for myocardial revascularization should be determined individually. The risk of ischemic events related to suboptimal antiplatelet therapy while awaiting surgery is < 0.1%, while that of perioperative bleeding complications associated with platelet inhibitors is > 10%.^378^

If the patient's clinical condition allows for delayed surgery, antiplatelet therapy should be stopped in a timely manner. Depending on the type and dosage of the agent, the effects should be reduced, e.g., 5, 5, and 7 days before the procedure for clopidogrel, ticagrelor, and prasugrel, respectively. The waiting time for surgery could be shortened by daily platelet aggregation testing to determine whether the patient has recovered from aggregation inhibition. An ongoing Brazilian study (NCT 02516267) is testing this hypothesis.

Current surgical management of NSTE-ACS is similar to that of stable disease. Therefore, after discussion with the heart team and appropriate surgical indication, the most important issue is determining the ideal time to perform the procedure. The best results are achieved by considering the systemic repercussions of the acute event while optimizing the patient's clinical condition. This means that patients could undergo surgery in the same hospital immediately, wait for a reduction of antiplatelet action, or be discharged with an upcoming surgery scheduled.

In an emergency, venous grafts are preferential to arterial grafts. Surgery may be performed with or without cardiopulmonary bypass, according to the technical conditions relevant to each case. In patients with cardiogenic shock, complete revascularization through an angioplasty procedure is an initial option; however, in view of the procedure's limitations, surgery may be indicated according to multi-professional assessment.^358^

Patients who undergo surgery 1-2 days and 3-7 days after AMI have similar mortality rates, which suggests that, for some patients, it would be possible to reduce the AMI-CABG interval without compromising the results. A higher mortality rate was observed in patients who underwent surgery on the first day after AMI.^379^

The After Eighty study, conducted in 16 Norwegian centers with NSTEMI and UA patients > 80 years of age, randomized patients to an invasive strategy (ATC or CABG + optimized clinical treatment) vs. conservative strategy with optimized clinical treatment alone. The best outcomes occurred in 93 (40.6%) out of 229 patients in the invasive strategy group and in 140 (61.4%) out of 228 patients in the non-invasive strategy group (RR 0.53 [95% CI 0.41-0.69], p = 0.0001). There was no difference in bleeding complications between the two groups.^380^

A multicenter study of 1810 AMI patients without ST elevation or UA within 48 hours of pain onset also compared invasive and conservative strategies. Although there was no difference in infarction or death rates in the first year of follow up, these rates were significantly lower in the invasive strategy group in 5 years.^381^

A systematic review that included 8915 patients, 4545 of whom were assigned to invasive strategy and 4370 to conservative strategy, also observed lower mortality between 6 and 12 months with the invasive strategy.^382^ More advanced techniques, such as FFR and intravascular ultrasound, can help determine risk stratification and the therapeutic strategy.^383^^-^^385^

**Table:** 

Myocardial revascularization (myocardial revascularization surgery and PCI) - Summary of recommendations and evidence		
According to the clinical picture and SYNTAX score, perform PCI or CABG in multiple vessel lesions.	I	B
Perform routine revascularization (PCI) for injuries unrelated to AMI in cardiogenic shock.	III	B

## Part 3 – Discharge Recommendations and Post-discharge Care

### 1. Lifestyle Change

Secondary prevention is a fundamental part of post-ACS patient care. This stage includes lifestyle changes, cardiovascular rehabilitation, education about risk factors, and promoting better treatment adherence. Despite its proven effectiveness in preventing negative outcomes, secondary prevention is still suboptimal.^386^^,^^387^ Patients and family members must be guided in a clear and understandable way regarding evidence-based benefits.^388^^-^^390^ Increasing the scope of secondary prevention is an important goal in this patient population.

Adequate guidance should be given about returning to work, sexual activities, and driving vehicles, working to gradually reintegrate patients into their routine. In addition, special attention should be paid to psychosocial and socioeconomic issues, including attention to the risk of depression and social isolation.^391^^-^^398^

#### 1.1. Smoking Cessation

Smoking cessation, a particularly important factor in lifestyle change, is a very effective measure for reducing mortality in post-NSTE-ACS patients.^399^ Cigarettes contain approximately 7,000 chemical components, many of which directly interfere with cardiovascular disease, 69 of which are carcinogenic.^400^ Cigarettes interfere with the cardiovascular system in various ways, increasing the heart rate and myocardial oxygen demand while decreasing supply.^401^ Smoking increases the incidence of sudden death,^402^ the risk of thrombosis, inflammation, vasoconstriction, and increased oxidation of LDL cholesterol.^403^ It is estimated that the risk of atrial fibrillation increases 1.5 to 2 times among smokers.^404^

In patients after NSTE-ACS, referral to a smoking cessation program and pharmacological agents, such as nicotine patches or gum, have proven useful.^405^ In an observational study of 12,656 patients, Parasuraman et al. found that, compared to ex-smokers and never smokers, smoking patients underwent coronary intervention at an earlier age, and the intervention was more associated with ACS. Former smokers had similar results to never smokers.^406^

The smoking cessation strategy should play a central role in the patient's overall rehabilitation and begin as soon as possible, even during the hospital phase. Family members who live in the patient's house should also be encouraged to participate in smoking cessation programs, thus reinforcing the patient's commitment, in addition to reducing the risk of secondhand smoke.^407^

Four steps are involved in treating nicotine addiction. The Fagerström test (Table 3.1) is commonly used for the first step, determining the addiction level. The next steps are counseling, medication/behavioral treatment, and clinical follow-up.

**Table 3.1:** Fagerström tolerance questionnaire

How many cigarettes do you smoke per day?
(0) less than 11
(1) between 11 and 20
(2) between 21 and 30
(3) more than 30
**2. How long after waking do you wait before having your first cigarette?**
(0) more than 60 min
(1) between 31 and 60 min
(2) between 6 and 30 min
(3) less than 6 min
**3. Do you find it difficult to refrain from smoking in places where it is forbidden?**
(0) no
(1) yes
**4. Does the first cigarette in the morning bring you the most satisfaction?**
(0) no
(1) yes
**5. Do you smoke more in the morning than during the rest of the day?**
(0) no
(1) yes
**6. Do you smoke even when you are bedridden due to illness?**
(0) no
(1) yes
Interpretation of the results (sum of the points for each question): 0 to 2 points = very low physical dependence 3 to 4 points = low physical dependence 5 points = medium physical dependence 6 to 7 points = high physical dependence 8 to 10 = very high physical dependence

Source: adapted from Ranney et al.^407^

To prevent relapse, it is important to identify high-risk situations and act to address them. The strategies basically consist of avoiding, escaping, distracting, and postponing. Parties, alcohol or caffeine intake, and meeting with smokers should be avoided, at least during the first weeks of cessation.

Medications such as bupropion, varenicline, and nicotine replacement therapy may increase the risk of cessation, especially in association with other types of therapy, such as support and counseling groups, psychotherapy, and alcohol withdrawal.

Bupropion is an antidepressant with anxiolytic action that several studies have found to decrease withdrawal symptoms. One study that compared smoking cessation among patients treated with bupropion 300 mg/day vs. placebo found abstinence rates of 44.2% and 9.2% in the bupropion and placebo groups, respectively.^408^ A literature survey revealed that bupropion appears to be more effective in patients who have abandoned and/or failed nicotine replacement therapy.^409^ On the other hand, two studies on smokers hospitalized for acute coronary events found no difference in smoking cessation between bupropion and placebo groups.^410^^,^^411^

Nicotine replacement therapy is effective and increases smoking cessation rates. In one study, any form of nicotine replacement resulted in up to a 60% increase in abstinence (RR: 1.60; 95% CI: 1.53 to 1.68) compared to placebo. The increase in efficacy only occurred when comparing an association of nicotine replacement modes to a single mode(RR: 1.34; 95% CI: 1.18 to 1.51).^412^ The most common long-term nicotine replacement mode is transdermal patches, while the most common short-term modes are chewing gum, nasal sprays, and inhalers, some of which are still not available in Brazil.

Varenicline is a nicotine receptor partial agonist that, by replacing the action of nicotine, reduces the intensity of withdrawal symptoms. Its bond with the receptor also reduces the reward and pleasure effects of smoking, which can reduce some of the effects of deprivation. Its effectiveness has been demonstrated in six clinical trials with a total of 3,659 chronic smokers.^413^^-^^416^ Varenicline doubled abstinence rates compared to placebo (RR: 2.24; 95% CI: 2.06 to 2.43).^417^ A study involving more than 8,000 smokers compared the use of varenicline, bupropion, nicotine patches,and placebo, randomized in a 1:1:1:1 ratio, and found a higher abstinence rate in the varenicline group at 6 months than the other groups.^418^ In a randomized study in patients with chronic coronary disease, the probability of continuous abstinence after 1 year was higher in the varenicline group than the placebo group (OR: 3.14; 95% CI: 1.94 to 5.11).^419^ Two randomized studies in smokers with ACS found higher rates of continuous abstinence at 24 weeks and a higher rate of abstinence at 52 weeks in the varenicline group than the placebo group.^420^^,^^421^ One of varenicline's main limitations is its high cost and the occurrence of nausea as a limiting side effect in some patients.

An association of methods seems to be more effective than treatment with a single method. In a recent meta-analysis of four studies, an association of varenicline + bupropion resulted in higher abstinence rates at 6 months than varenicline alone. The effects were more pronounced in more nicotine-dependent and heavier smokers. The benefits, however, did not last for 12 months, and the association significantly increased anxiety symptoms.^422^ Therefore, an association between methods appears to be safer and more effective when varenicline or bupropion is combined with nicotine replacement therapy.

Bupropion and varenicline also appear to be safe for patients with cardiovascular disease. Some studies have found an increased occurrence of neuropsychiatric events with these medications, which led the U.S. Food and Drug Administration to require a label warning. After further study, this risk was reassessed, and the warning was withdrawn in 2016.^423^

Replacing traditional cigarettes with low-nicotine cigarettes or vaporizers (electronic cigarettes) is a controversial issue.^424^ Both strategies are only classified as potential damage control.

Studies on low-nicotine cigarettes indicate that they have little effect, since smokers generally compensate by smoking more cigarettes or adding other forms of nicotine.^425^ Vaporizers apparently expose the smoker to fewer carcinogens than traditional cigarettes, although there is great variability in the toxic substances they contain. In addition, there has been an explosive increase in their use among young people, especially in the USA.^426^ Sale of these devices is prohibited in Brazil. They have been associated with several recent deaths in the USA due to acute lung problems from inhaled substances. A total of 2,807 vaping-related hospitalizations and 68 deaths have been confirmed by the U.S. Centers for Disease Control. To date, there is no evidence that these strategies facilitate smoking cessation, and electronic cigarettes, in particular, are potentially dangerous.

**Table:** 

Smoking cessation - Summary of recommendations and evidence		
Patients should stop smoking and avoid exposure to environments with smokers, both at work and at home.	I	A
Long-term follow-up and referral to specific programs or pharmacotherapy (including nicotine replacement) are useful when associated with traditional non-pharmacological strategies.	I	A
Low-nicotine cigarettes can be used for damage control.	III	C
Vaporizers can be used as a damage control and smoking cessation strategy in resistant patients	III	B

#### 1.2. Dietary Recommendations

Since patients with NSTE-ACS are classified as very high cardiovascular risk, a considerable reduction in cholesterol values is absolutely indispensable. In addition to statin therapy, which is supported by the results of several meta-analyses^427^^,^^428^ and randomized trials,^429^^-^^432^ dietary therapy is of central importance and is supported by significant evidence.^433^^,^^434^

A standard diet rich in fruits, vegetables, whole grains, healthy protein sources (dairy products, low-fat [skinless] poultry, fish/seafood, and nuts, non-tropical vegetable oils is recommended. Sweets, sugary drinks, and sugar itself shuld be avoided, as well as excess red meat. The PREDIMED trial tested a Mediterranean-type diet, finding a reduction in cardiovascular events, especially stroke.^435^

This diet must be adjusted for appropriate caloric requirements, cultural patterns, food preferences,,a nd nutritional therapy for other conditions, including DM. Caloric intake should be adjusted to avoid weight gain and, for overweight/obese patients, it must promote weight loss. Vitamins or antioxidant food supplements should not be routinely prescribed for secondary prevention.^436^

### 2. Cardiovascular Rehabilitation

Physical exercise reduces atherogenesis, promotes anti-inflammatory action, improves endothelial function, decreases sympathetic tone, increases HDL, and reduces blood pressure and insulin resistance, among other beneficial effects, and should not be denied to patients after ACS.^437^ Exercise is included in the therapeutic context of cardiovascular rehabilitation programs.. Sedentary lifestyle, a worldwide problem, is the main target of primary prevention and is even more important for secondary prevention of coronary events.

The cardiovascular rehabilitation process of ACS patients should begin during hospitalization withphysical therapy, including early walkingand passive and active movement of the main muscle groups. Even while hospitalized, the patient should be familiarized with scales of perceived effort (e.g., the BORG scale), which will be useful after discharge.

At discharge, every patient should receive adequate guidance about physical activity, which should initially be mild intensity, a continuation of the exercises performed during the hospital phase (phase 1), including self-monitoring techniques. Early assessment in a formal rehabilitation program should be indicated, or, when unavailable, the patient should be advised about an exercise program that can be administered by a physical therapist or physical educator according to doctor-imposed limits (phase 2).

Studies have shown that patients referred to exercise programs after an acute coronary event have a better quality of life, less symptom recurrence, and fewer new cardiovascular events.^438^^-^^440^

Despite the positive effects of rehabilitation programs, systematic referral and compliance are still major challenges.^441^ A lack of continuing medical education in cardiac rehabilitation, a lack of knowledge about program safety (event rate of 1 for every 112,000 patients/hour), and difficulty making these programs cost-effective are important parts of the problem.

#### 2.1. Instructions about Physical Activity at Discharge

The first step in assessing patients for a cardiovascular rehabilitation program after clinical evaluation and complementary exams is to determine whether there is any absolute contraindication to physical exertion.^442^^,^^443^ Table 3.2 lists contraindications to regular physical activity.

**Table 3.2:** Risk classification for exercise training in cardiac patients

Low risk	Moderate risk	High risk
LVEF > 50% No complex arrhythmias No symptoms of congestive HF No angina upon exertion or in the recovery period No severe or moderate valve injury Functional capacity ≥ 7 METS	LVEF: 40% to 49% Signs/symptoms, including moderate angina during exercise (5 to 6.9 METS) or in the recovery period Moderate valve injury	LVEF < 40% cardiac arrest or sudden death survivors Complex ventricular arrhythmias at rest or during exercise Severe valve disease Uncorrected congenital heart disease Functional capacity < 5 METSIschemic ST-segment depression during exercise > 2 mm

LVEF: left ventricular ejection fraction; METS: metabolic equivalents.

The risk classification for physical activity in cardiac patients is based on clinical data regarding angina, functional classification, ventricular function, residual coronary lesions, and arrhythmias. This classification is useful for determining the level of support required during the program and the need for monitoring during exercise sessions.^442^^,^^443^

Low-risk patients may begin exercising with a physical therapist or physical educator, without necessarily being supervised by a physician. Moderate risk patients, however, should preferably exercise in an environment with medical supervision and advanced life support resources; they can progress to a semi-supervised external program if no complications occur after 12 weeks. When a medically supervised cardiovascular rehabilitation program is not available, these patients can exercise under the supervision of a physical educator or physical therapist, conforming to safety limits predetermined by the cardiologist. The exercise team must have training in at least basic life support and an automatic external defibrillator must be available on site. High-risk patients should exercise under medical supervision, with a physical therapy and/or physical education team trained in basic life support and with local resources for advanced life support.

In all situations, heart rate monitoring should guide exertion intensity. The need for electrocardiographic monitoring during the rehabilitation program can be defined on a case-by-case basis by the medical team.

Safe exercise zones can be ideally defined through a functional test (cardiopulmonary test or ET) or, in specific cases, they can be guided by perceived effort. When a cardiopulmonary test is available, aerobic exercise should generally involve a heart rate between what is indicated by the anaerobic threshold and the respiratory compensation point, ie, a zone at which, despite the production of lactic acid, there is no decompensated metabolic acidosis, which is associated with a lower risk of arrhythmic events or cardiovascular overload. When a cardiopulmonary test is not available, exercise at between 70% and 85% of the maximum heart rate achieved in an ET or 50% to 80% of the heart rate reserve added to the resting heart rate, where: heart rate reserve = peak heart rate - resting heart rate. In patients with ECG changes or symptoms suggestive of ischemia on exertion, the limit should, in general, be 10 beats less than symptom onset or ST change. In specific cases and under medical supervision, the patient can approach the point of ischemia onset.

The doctor should ideally determine the following exercise parameters:

- Weekly frequency.
- Intensity (using a Borg scale and the heart rate or load determined in a cardiopulmonary or ET).
- Mode (eg, walking, cycling, rowing).
- Session duration.
- Resistance exercises should also be encouraged. The recommended intensity is 40% to 60% of the maximum voluntary contraction (low to moderate intensity), 8 to 15 repetitions, 1 to 3 sets.^443^^,^^444^

**Table:** 

Cardiopulmonary rehabilitation - Summary of recommendations and evidence		
Physical activity guidance should be provided at discharge; cardiac rehabilitation programs should be encouraged in all patients after ACS.	I	B
Physical activity guidance should be provided at discharge; cardiac rehabilitation programs should be encouraged in all patients after ACS.	I	B
Physical activity guidance should be provided at discharge; cardiac rehabilitation programs should be encouraged in all patients after ACS.	III	A

### 3. Medications to be Prescribed at Discharge

#### 3.1. Antithrombotic drugs

##### A. Switching Antiplatelet Treatment

Although a second antiplatelet may have been previously selected for use with ASA, at the time of hospital discharge it may be necessary to switch to a different antiplatelet for some reason, such as:

- Cost.
- Limiting adverse effects (eg, dyspnea with ticagrelor, bleeding while using the most potent drugs, such as ticagrelor and prasugrel).
- Patient preference for a single daily dose (eg, prasugrel, if treated with PCI and without prior stroke/TIA) rather than twice a day (ticagrelor).

The transition from clopidogrel to ticagrelor has been the only such change studied with sufficient power to assess clinical outcomes, despite the fact that the study was not developed for this purpose. In the PLATO trial, 46% of the patients randomized to receive ticagrelor had been pretreated with clopidogrel (300 to 600 mg dose).^152^ The safety and efficacy of ticagrelor were not affected by previous use of clopidogrel.^248^ In the TRITON-TIMI-38 trial, which evaluated the use of prasugrel, previous use of another P2Y_12_ inhibitor was considered an exclusion criterion.^154^ Other changes in P2Y_12_ inhibitors (e.g., between ticagrelor and prasugrel or from ticagrelor/prasugrel to clopidogrel) have been evaluated by pharmacodynamic and observational studies, which serve more as hypothesis generating, since they were not designed to assess clinical outcomes.^445^^-^^449^ Thus, there is less evidence to support such transitions. Figure 3.1 shows the recommendations for switching P2Y_12_ agents in patients with ACS for less than 30 days.^450^^,^^451^

**Figure 3.1:** Recommendations for switching P2Y_12_ inhibitors in the first 30 days after acute coronary syndrome. A loading dose of the new agent is routinely recommended. *If prasugrel or ticagrelor therapy is being de-escalated due to bleeding or an increased risk of bleeding, only a maintenance dose of clopidogrel can be considered. Adapted from Valgimigli et al.^451^

Thus, as a general rule, when switching P2Y_12_ antiplatelet therapy during the acute phase of coronary disease (< 30 days), a loading dose of the new medication should be used, followed by a maintenance dose. One exception to this rule is when prasugrel or ticagrelor is replaced with clopidogrel due to bleeding or increased risk of bleeding. The new drug should be administered 24 hours after the final administration of the first drug, with the exception of when clopidogrel is being replaced.

When the patient has had ACS for more than 30 days, and it becomes necessary to switch agents, see Figure 3.2.

**Figure 3.2:** Recommendations for changing P2Y_12_ inhibitors in patients after 30 days of acute coronary syndrome. * If ticagrelor to clopidogrel therapy is being de-escalated due to bleeding or an increased risk of bleeding, only a maintenance dose of clopidogrel can be considered. Adapted from Valgimigli et al.^451^

Some differences should be considered:

- In all situations, a 24-hour wait is recommended between the final dose of the previous medication and the first dose of the new medication.
- A loading dose of the new agent is recommended only when ticagrelor is being replaced. Clinical studies have shown greater platelet reactivity in the first 48 hours when changing from ticagrelor to clopidogrel^448^^,^^449^ or prasugrel.^447^ This difference is due to the fact that ticagrelor is a reversible P2Y_12_ inhibitor, while thienopyridines (clopidogrel and prasugrel) are non-reversible.

**Table:** 

Switching P2Y_12_ inhibitors - Summary of recommendations and evidence		
In admitted ACS patients who received clopidogrel and wish to change to ticagrelor in the first 30 days, a loading dose of 180 mg should be administered, regardless of the timing and dosage of clopidogrel.	I	B
Other changes in oral P2Y_12_ inhibitors should be considered in light of adverse effects or other factors and should follow the regimens recommended in this Guideline.	IIb	C

##### B. Duration of Dual Antithrombotic Therapy

###### B.1. More than 12 Months

####### B.1.1. Antiplatelets

Studies have assessed the use of clopidogrel, prasugrel, and ticagrelor in ACS patients on DAPT for an average of 12 months. This is the recommended time, regardless of therapeutic strategy (clinical treatment, angioplasty, or myocardial revascularization surgery). For patients who receive clinical treatment, the only options for antiplatelet drugs are ticagrelor and clopidogre, since it has been demonstrated that prasugrel is not superior to clopidogrel in such cases.^452^

DAPT is a very effective way to prevent stent thrombosis. The risk of late (between 1 month and one year after stent implantation) and very late stent thrombosis (> 1 year after angioplasty) has been decreasing considerably with the use of more modern drug-eluting stents. Thus, continuing DAPT more than one year after an ACS event and angioplasty seems to carry an increased risk of bleeding that is more harmful than the potential benefits of preventing very late stent thrombosis. However, the PEGASUS trial demonstrated that in patients with a history of infarction (> 1 year) and additional high risk criteria (DM, age > 65 years, chronic kidney disease, multivessel coronary diseas or two or more previous infarctions), prolonged DAPT with ASA + ticagrelor reduced the incidence of ischemic outcomes, including new cases of infarction unrelated to stent thrombosis or stroke, compared to ASA monotherapy (HR 0.84; 95% CI 0.74 to 0.95; p = 0.004 and HR 0.85; 95% CI 0.75 to 0.96; p = 0.008, for doses of 60 mg and 90 mg, respectively).^453^ Continuing DAPT beyond 12 months would thus be more associated with systemic protection against thrombotic events than with preventing late stent thrombosis. This finding was reinforced by a subanalysis of the same trial, which found a similar reduction of ischemic cardiovascular events in patients with no history of stent angioplasty and patients with previous angioplasty.^454^ However, the decrease in cardiovascular events was accompanied by an increase in major bleeding episodes (HR 2.32; 95% CI 1.68 to 3.21; p < 0.001 and HR 2.69, 95% CI 1.96 to 3, 70; p < 0.001, for doses of 60 and 90 mg, respectively). There was no increase in fatal bleeding or intracranial hemorrhage with ticagrelor. The highest dose of ticagrelor (90 mg, twice a day) did not reduce ischemic risk and led to higher bleeding rates. Thus, the 60 mg dose is approved for selected patients 1 year after NSTEMI who tolerated DAPT during this period.

The DAPT trial also assessed the use of DAPT for > 12 months with clopidogrel and prasugrel (65.2% and 34.8% of patients, respectively).^455^ All included patients had undergone angioplasty with a drug-eluting st,ent and angioplasty in the context of NSTE-ACS was performed in approximately one third of the patients. In this study, patients received DAPT for a standard 12-month period and, if they adhered to treatment and did not experience relevant bleeding, they were then randomized to continue DAPT or ASA + placebo for another 18 months. Like the PEGASUS trial, a reduction in ischemic events occurred, but this benefit was offset by the increased risk of bleeding. Although stent thrombosis was reduced by one case in every 100 patients treated with prolonged DAPT, major or moderate bleeding occurred in one out of every 111 patients.

A meta-analysis of > 32,000 patients before the publication of the PEGASUS trial found a significant increase in infarction incidence in the group that received DAPT for 12 months (compared with > 12 months; OR = 1.57, 95% CI 1.30 to 1.90), as well as a lower incidence of bleeding (OR = 0.65, 95% CI 0.52 to 0.81).^456^ Subsequent meta-analysis, including the PEGASUS trial, found a significant decrease in all-cause mortality in the DAPT > 12 months group (HR = 0.89, 95% CI 0.79 to 0.99).^457^ It was concluded that any of the three P2Y_12_ inhibitors available in Brazil (clopidogrel, ticagrelor, and prasugrel) can reduce ischemic events when used in association with ASA for more than 12 months, although at an increased risk of bleeding. Thus, patient risk for ischemic events and bleeding must be individually assessed. Clinical scores allow ischemic and hemorrhagic risk to be estimated with more objective parameters according to the patient's clinical characteristics, assisting with decision making regarding the duration of DAPT.

The PRECISE-DAPT trial assessed 14,963 patients who had undergone angioplasty, using a score that consisted of five variables (age, creatinine clearance, hemoglobin, leukocyte count, and a history of spontaneous bleeding) to predict the risk of bleeding.^458^ The score was also validated in a group of more than 14,000 patients. Patients with low scores (< 25) benefitted from prolonged DAPT, including a reduction in the composite ischemic outcome (AMI, definitive stent thrombosis, stroke, and need for revascularization); the number needed to treat was 65, and there was no significant increasing in risk of bleeding. On the other hand, in patients with high scores (≥ 25), prolonged DAPT was associated with an increased risk of bleeding (number needed to harm = 38) and provided no benefits regarding ischemic outcome prevention. It should be pointed out, however, that this score was derived from randomized studies with PCI (with or without ACS) and not exclusively from a population with ACS, whose risk of ischemic events tends to be higher than that of patients with stable CAD.

The DAPT score (Table 3.3) was derived from a cohort in the DAPT trial.^459^ In this analysis, patients with a high risk score (≥ 2) who received DAPT for 30 months had a significant reduction in ischemic outcomes (number needed to treat = 34) and a small increase in the risk of bleeding (number needed to harm = 272). On the other hand, in patients with a low score (< 2), prolonged DAPT led to no reduction in ischemic risk and a considerable increase in the risk of bleeding (number needed to harm = 64).

**Table 3.3:** Dual Antiplatelet Therapy trial score

Variables	Points
Age ≥ 75 years	–2
Age between 65 and 74 years	–1
Age < 65 years	0
Current smoker	1
Diabetes mellitus	1
AMI at initial presentation	1
Previous PCI or AMI	1
Stent diameter < 3 mm	1
Stent eluted with paclitaxel	1
HF or LVEF < 30%	2
PCI in saphenous vein graft	2

AMI: acute myocardial infarction; PCI: percutaneous coronary intervention; HF: heart failure; LVEF: left ventricular ejection fraction.

####### B.1.2. Anticoagulants

The COMPASS study randomized > 27,000 patients with chronic coronary disease to rivaroxaban 2.5 mg twice a day + ASA 100 mg once a day (n = 9152) or rivaroxaban 5 mg twice a day (n = 9117) or ASA 100 mg (n = 9126). In an average follow-up of 23 months, rivaroxaban + ASA was superior to ASA alone (HR = 0.76, 95% CI 0.66 to 0.86 for the primary outcome (cardiovascular death, AMI or stroke), although it increased the incidence of bleeding (HR = 1.70, 95% CI 1.40 to 2.05). Importantly, there was a significant decrease in all-cause mortality (HR = 0.82, 95% CI 0.71 to 0.96) but no significant difference in the incidence of fatal or intracerebral bleeding.^460^ Considering only the rivaroxaban + ASA and ASA groups, 2423 patients had a history of AMI < 2 years, 3279 between 2 and 5 years, and 5673 > 5 years. The comparison between groups showed hazard ratios of 0.70 for patients with AMI < 2 years, 0.81 for those with AMI between 2 and 5 years, and 0.72 for those with AMI > 5 years (P-interaction = 0.93).^461^

###### B.2. Less than 12 Months

Ticagrelor monotherapy was evaluated in the GLOBAL LEADERS trial ^462^ in 15,968 patients undergoing PCI (34% with NSTE-ACS). The groups received ASA + ticagrelor for 1 month, followed by ticagrelor monotherapy for 23 months or by standard DAPT with ASA + clopidogrel (stable CAD) or ASA + ticagrelor (acute CAD) for 12 months, followed by ASA monotherapy for 12 more months. At the end of 2 years of follow-up, the primary outcome (all-cause mortality or infarction) was similar in both groups (RR 0.87, 95% CI 0.75 to 1.01). The secondary outcome, bleeding (BARC 3 or 5), was also similar in both groups (RR = 0.97, 95% CI 0.78 to 1.20). Secondary analysis in a later publication found a significant decrease in the primary outcome in patients with acute CAD and PCI in more than one coronary artery.^463^

The TWILIGHT trial evaluated whether early discontinuation of ASA would be safe in patients treated with PCI who had a high risk of thrombosis or bleeding according to clinical and angiographic characteristics.^464^ A total of 7119 patients (4614 with NSTE-ACS) were included, all treated for 3 months after PCI with ASA + ticagrelor. After this period, they were randomized to continue with DAPT or ticagrelor monotherapy for another 9 months. The main outcome was bleeding (BARC 2, 3 or 5). At the end of follow-up, the ticagrelor monotherapy group there was a 44% reduction (HR 0.56; 95% CI: 0.45-0.68; p < 0.001) in the main outcome, with no increase in ischemic risk (secondary outcome, insufficient sampling power for this conclusion).

In a recently published meta-analysis, O'Donoghue et al. assessed monotherapy with P2Y_12_ inhibitors vs. DAPT in approximately 17,000 patients with acute coronary disease, finding a hazard ratio of 0.50 (95% CI 0.41 to 0.61) for bleeding, and a hazard ratio of 0.85 (95% CI 0.70 to 1.03) for major adverse cardiac events.^465^

**Table:** 

Duration of dual antithrombotic therapy in patients with NSTE-ACS in sinus rhythm - Summary of recommendations and evidence		
After NSTE-ACS, DAPT should be continued for 12 months, regardless of the clinical strategy (angioplasty, myocardial revascularization surgery, or clinical treatment).	I	A
In patients with NSTE-ACS and increased risk of bleeding, DAPT should be continued for only 6 months, with the P2Y_12_ inhibitor suspended after this period, regardless of the clinical strategy (angioplasty, coronary artery bypass surgery, or clinical treatment)	IIa	B
In NSTE-ACS patients undergoing PCI, DAPT may be considered for 3 months, followed by P2Y_12_ inhibitor monotherapy (preferably ticagrelor).	lla	A
Associate a second antithrombotic medication (see table below) with ASA after 12 months of DAPT in patients with a high ischemic risk and a low risk of bleeding.	IIa	A

**Table:** 

Extended antithrombotic/antiplatelet therapy options			
Medication	Trial	Dose	Indication
Clopidogrel	DAPT	75 mg per day	Post-angioplasty tolerant to DAPT for 1 year
Prasugrel	DAPT	10 mg per day (5 mg if weight < 60 kg or age > 75 years)	Post-angioplasty tolerant to DAPT for 1 year
Ticagrelor	PEGASUS-Tl MI-54	60 mg twice/day	Post-infarction with increased ischemic risk
Rivaroxabana	COMPASS	2,5mg 2 vezes/ dia	Pacientes coronariopatas ou com doenga arterial periferica com alto risco trombotico

Care should be taken when prescribing antiplatelet drugs to patients with ACS who, during hospitalization, undergo CABG. Subanalyses of the CURE,^466^ PLATO,^249^ and TRITON^237^ trials showed that patients undergoing surgical revascularization seemed to benefit like the other patients included in these trials. Thus, thienopyridine should be reintroduced as soon as it is considered safe after surgical revascularization.

##### C. Antiplatelet Management in Patients Who Need Chronic Anticoagulation

Evidence about antiplatelet management in patients who need chronic anticoagulation is, for the most part, restricted to atrial fibrillation (AF) patients. The incidence of AF in patients with NSTE-ACS ranges from 5% to 23%.^467^ The standard therapy for ACS is DAPT for at least 12 months to prevent ischemic events. In patients with AF and a CHA_2_DS_2_VAS_C_ score ≥ 2, chronic anticoagulation is recommended to prevent thromboembolic events. When both of these conditions are present, it becomes important to determine the best combination of antithrombotic agents, including the appropriate dose and the ideal duration of each treatment. These questions were evaluated in a series of phase 3 clinical trials.

The first of these studies was the WOEST trial,^468^ which recruited 573 patients with AF who were on anticoagulants and underwent angioplasty. The patients were then randomized into two groups: one that used clopidogrel associated with anticoagulant and another that used triple therapy (anticoagulant + clopidogrel + ASA). Dual therapy was associated with a lower risk of bleeding at 1 year (19.4% vs. 44.4%, HR 0.36, 95% CI 0.26-0.50, p <0.0001) without a greater risk of ischemic events.

The first randomized trial to assess DOAC in the context of double and triple therapy in patients with AF who underwent angioplasty was PIONEER AF-PCI.^268^ This trial compared three antithrombotic strategies: rivaroxaban 15 mg/day associated with a P2Y_12_ inhibitor; triple therapy with DAPT and low-dose rivaroxaban (2.5 mg every 12 h); triple therapy with DAPT and a vitamin K antagonist. Strategies involving rivaroxaban were associated with a lower risk of bleeding than triple therapy with vitamin K antagonists. There was no difference in ischemic outcomes, but the sample size (2124 patients) had insufficient power for this outcome. One criticism of this study was the fact that the low doses of rivaroxaban had not been approved for antithrombotic treatment in AF.

The following year, the RE-DUAL PCI trial was published, which randomized 2725 patients with AF who underwent angioplasty.^269^ Dual therapy with dabigatran at different doses (150 mg twice a day and 110 mg twice a day) associated with a P2Y_12_ inhibitor was compared to triple therapy with warfarin. In the triple therapy group, ASA was continued for 1 month in patients who received a non-drug-eluting stent, and for 3 months in patients who received a drug-eluting stent. The two dabigatran regimens were associated with a lower risk of bleeding than the triple therapy warfarin regimen. No interaction was found between proton pump inhibitors and the effect of the drug, since this class of drugs did not influence the main results of the study.^469^ The design of RE-DUAL PCI and PIONEER AF-PCI did not allow assessment of whether the reduced risk of bleeding in dual therapy was secondary to replacing vitamin K antagonists with DOAC or to excluding ASA from the treatment.

The AUGUSTUS trial, published in 2019,^270^ set out to answer this question by randomizing 4,614 patients with AF who had undergone PCI or had ACS in the last 14 days. The study used a 2x2 factorial design; patients were randomized into groups that compared apixaban 5 mg twice a day to warfarin, and groups that compared ASA to placebo. All patients received a P2Y_12_ inhibitor, which was clopidogrel in more than 90% of cases. Thus, four groups were studied: (1) P2Y_12_ inhibitor + warfarin + ASA; (2) P2Y_12_ inhibitor + warfarin + placebo; (3) P2Y_12_ inhibitor + apixaban + ASA; (4) P2Y_12_ inhibitor + apixaban + placebo. In the comparison between apixaban and warfarin, there was a lower risk of bleeding with apixaban. One major or clinically relevant bleeding episode was avoided during the study period for every 24 patients treated with apixaban when compared with warfarin. In the comparison of ASA vs. placebo, it was demonstrated that ASA increased the risk of bleeding. There was one additional significant bleeding episode in 6 months for every 14 patients treatedwith ASA when compared with placebo. Compared to triple therapy, there was no increase in ischemic outcomes with double therapy; however, the study was not powered to assess efficacy outcomes. It is important to point out that in the AUGUSTUS trial, patients were randomized an average of 6 days after PCI or ACS. Thus, patients received at least a short course of ASA and triple therapy prior to randomization. Another relevant fact about this study is that it also included patients with AF and medically managed ACS (approximately 25% of the sample).

The ENTRUST trial randomized 1506 patients with AF who underwent coronary angioplasty, following them for 12 months.^271^ The study design compared double therapy with edoxaban 60 mg/day associated with clopidogrel 75 mg/day to traditional triple therapy (warfarin + double antiplatelet therapy with ASA + clopidogrel). Dual therapy with edoxaban was non-inferior to triple therapy with warfarin regarding the primary outcome of bleeding. However,this was the only randomized clinical trial that did not demonstrate a superiority for bleeding outcomes of a dual therapy with a DOAC compared to a triple therapy with warfarin.

One possible concern with dual therapy in AF patients who undergo angioplasty would be an increased ischemic risk, especially for stent thrombosis. The five previously mentioned trials (WOEST, PIONEER AF-PCI, RE-DUAL PCI, AUGUSTUS and ENTRUST) were designed to determine whether less aggressive antithrombotic therapy resulted in reduced bleeding. None of them, however, had sufficient power to adequately assess increased ischemic risk. To assess this issue, two meta-analyses were developed, both of which demonstrated that, compared to warfarin + DAPT, dual antithrombotic therapy decreases hemorrhagic events.^272^^,^^470^^,^^471^ However, when comparing double and triple antithrombotic therapy, Gargiulo et al.^472^ found that dual antithrombotic therapy led to a significant decrease in bleeding at the expense of a significantly higher incidence of stent thrombosis (RR = 1.59, 95% CI 1.01 to 2.50) and a trend toward increased AMI, suggesting that some patients with a very high risk of ischemic events and (especially) a low risk of bleeding events may benefit from triple antithrombotic therapy for up to 30 days after the index event (Figure 3.3).

**Table:** 

Antithrombotic therapy in patients after ACS who have AF and are indicated for anticoagulation - Summary of recommendations and evidence		
DOAC is preferable to warfarin	I	A
Clopidogrel is the preferred P2Y_12_ inhibitor, since it has been the most studied drug in this context	I	A
Triple therapy should be continued for the shortest possible time due to the high risk of associated bleeding.	IIa	A
When using warfarin, an INR between 2 and 2.5 is desirable.	IIa	C
ASA should be used in low doses, preferably ≤ 100 mg/day.	IIa	C
During hospitalization, triple therapy with ASA + P2Y_12_ inhibitor + anticoagulant should be used.	IIa	C
After hospital discharge, P2Y_12_ inhibitor and anticoagulant should be routinely continued for up to 12 months after the event. The P2Y_12_ inhibitor should be discontinued after 12 months, continuing only with the anticoagulant.	IIa	A
In patients with increased ischemic risk, triple therapy can be maintained for a longer period.	IIa	B
PPI should be considered the first choice as prophylaxis for stress ulcers due to the high risk of gastrointestinal bleeding.	IIa	C

INR: inInternationalormalized ratio

**Figure 3.3:** Antithrombotic regimen proposed for patients with non-ST-elevation acute coronary syndrome who have AF and are indicated for anticoagulation.

#### 3.2. Renin-Angiotensin-Aldosterone System Inhibitors

ACE inhibitors are recommended for post-ACS patients with systolic LV dysfunction/HF, systemic arterial hypertension or DM. Angiotensin receptor blockers can be used in patients intolerant to ACE inhibitors.

Mineralocorticoid antagonists are indicated in post-ACS patients with LVEF ≤ 40% associated with evidence of HF or DM.^283^

When managing arterial hypertension after hospitalization for ACS and in the long term, special care must be taken to maintain adequate blood pressure control, as recommended by the Brazilian Hypertension Guidelines.^473^ However, the J-curve should also be considered, despite the controversy about its existence and role. The ONTARGET study^474^^,^^475^ demonstrated that excessive reductions in SBP were associated with an increase in ischemic events and that a SBP of 126 mmHg was associated with the lowest occurrence of AMI. The TNT study^476^ assessed the impact of blood pressure levels on more than 10,000 coronary disease patients. More ischemic events occurred when SBP was below 110-120 mmHg, and diastolic blood pressure was below 60-70 mmHg. However, the SPRINT study, which randomized 9,361 patients without DM and high cardiovascular risk to an intensive control goal (SBP < 120 mmHg) or a conventional goal (SBP < 140 mmHg), found that intensive treatment reduced the occurrence of the composite outcome (cardiovascular death, infarction, stroke, ACS, or HF)(HR 0.75; 95% CI 0.64 to 0.89; p < 0.001), as well as all-cause mortality (HR 0.73; 95% CI 0, 60 to 0.90; p = 0.003). There was an increased incidence of adverse events from falls, syncope, hypotension, and acute renal failure in the intensive group. No increase in infarction or ACS was found in the intensive treatment group. However, the study included less than 20% of patients with overt cardiovascular disease.^477^ No other studies have been conducted specifically on patients with a history of ACS. Thus, due to the potential for worsening ischemia, lowering the patient's blood pressure should be viewed with caution. To avoid reducing coronary perfusion pressure, diastolic blood pressure should not fall below 60 mmHg.

**Table:** 

Renin-angiotensin-aldosterone system inhibitors in patients after NSTE-ACS - Summary of recommendations and evidence		
Renin-angiotensin-aldosterone system inhibitors in patients after NSTE-ACS - Summary of recommendations and evidence	I	A
Spironolactone is recommended in patients with LVEF ≤ 40% associated with HF or DM.	I	A

#### 3.3. Beta-blockers

Long-term use of beta-blockers is recommended in patients with HF and an EF ≤ 40%. In patients with preserved LV systolic function, the data are less robust. Evidence favorable to beta-blockers emerged in the early 1980s, when ACS treatment differed substantially from current protocols.^478^^,^^479^ Their use in NSTE-ACS patients without HF has not been evaluated in randomized clinical trials. The data available for this population are derived from observational studies suggesting that long-term beta-blocker use after AMI is useful.^480^^,^^481^ A specifically designed Brazilian study that followed NSTE-ACS patients for approximately 17 years suggested that the long-term use of beta-blockers benefits populations with LV dysfunction, but not those with normal EF.^137^ The ongoing DANBLOCK study (NCT 03778554) is randomizing patients with recent myocardial infarction and EF > 40% to assess whether beta-blockers reduce ischemic outcomes in the context of current ACS therapy. The results are expected in 2023.

**Table:** 

Use of beta-blockers in patients after non-ST-segment elevation acute coronary syndrome - Summary of recommendations and evidence		
The indefinite use of beta-blockers is recommended in patients with LV dysfunction. Agents with proven effectiveness in this context should be used.	I	B

#### 3.4. Antidiabetic drugs

Over 30% of NSTE-ACS patients have DM.^482^ The treatment of this comorbidity has undergone major changes in recent years with the arrival of drugs that not only lower blood glucose levels, but also reduce the incidence of cardiovascular events. There is evidence that more intensive control of glycated hemoglobin (HbA1c) in diabetic patients reduces the risk of microvascular complications.^483^^,^^484^ Their impact on macrovascular events has been more heterogeneous, although there seem to be benefits in long-term follow-up (> 10 years), as long as treatment begins soon after diagnosis.^485^ Based on microvascular event findings, an HbA1c target of less than 7% is recommended in most diabetic patients. It is important to point out, however, that this number is more of a general principle and it should be individualized for each patient. In the ACCORD trial, for example, more aggressive glycemic control, in addition to not reducing major cardiovascular outcomes, was associated with higher overall mortality, which led to early interruption of the study.^486^ The included patients had long-standing DM (10 years on average), in addition to a high prevalence of cardiovascular disease (approximately 35%). Therefore, in patients with low life expectancy, long-standing DM, a history of severe hypoglycemia, advanced microvascular or macrovascular disease, less stringent HbA1c targets (eg, < 8%) can be considered. Conversely, in patients with recent-onset DM, no relevant comorbidities, and a good understanding of the disease, more intensive targets (eg, HbA1c < 7%) can be targeted.

Once the glycemic targets have been determined, the drugs must be chosen. Every patient diagnosed with DM should receive metformin unless it is formally contraindicated.^487^ This low-cost medication is widely available in the public health system and has favorable effects on weight reduction and glycemic control, in addition to potentially reducing the incidence of cardiovascular events.^484^ Recent randomized trials have shown that treatment with new antidiabetics in association with metformin has reduced cardiovascular outcomes in more than 70% to 80% of patients.

The main side effects of metformin are gastrointestinal (feeling of bloating, abdominal pain, diarrhea) and can be avoided with a slower dose progression. Patients with creatinine clearance below 30 mL/min are formally contraindicated to the drug.^488^ Chronic metformin treatment is associated with a higher incidence of low vitamin B12 levels, and monitoring should be considered in this population, especially if there is associated anemia or neuropathy.^489^

In patients with a history of coronary disease, therapy with SGLT2 inhibitors or GLP1 receptor agonists should be considered, regardless of the HbA1c level. Medications that have already shown benefits in reducing cardiovascular events in phase 3 studies should be selected.

Choosing between SGLT2 inhibitors and GLP1 receptor agonists depends on several factors, some of which are shown in Table 3.4. Both drug classes have similar reductions in major cardiovascular outcomes in secondary prevention patients.^490^ Ideally, the decision should be shared with the patient, weighing practical aspects such as route of administration (oral vs. subcutaneous) and price, as well as the clinical benefits found in randomized studies. Due to their benefits beyond glycemic control, these drugs should be prescribed as soon as possible after ACS, since diabetic patients with ACS are a very high risk group and therapeutic inertia should be avoided. In a subanalysis of patients with previous AMI from the DECLARE trial, dapagliflozin reduced the composite outcome of cardiovascular death, infarction and ischemic stroke (mainly driven by a reduction in reinfarction) compared to placebo, and this benefit appeared to be greater closer to the acute phase of a coronary event.^491^

**Table 3.4:** Factors to be considered when deciding on the best new antidiabetic treatment in coronary patients

	SGLT2 inhibitors	GLP1-RA
Method of administration	Daily oral administration	Subcutaneous (oral semaglutide already exists, but is not yet available in Brazil) Daily or weekly administration
Benefits	Reduces MACE Reduces cardiovascular death (empagliflozin) Reduces HF Reduces (slightly) AP Prevents progression of nephropathy	Reduces MACE Reduces cardiovascular death (liraglutide) Weight loss Prevents progression of nephropathy (liraglutide)
Caution	Creatinine clearance < 30 mL/min/1.73 m^²^ History of genital infections History of ketoacidosis Osteoporosis/fractures (canagliflozin) Peripheral arterial disease (canagliflozin)	Creatinine clearance <30 mL/min/1.73 m^²^ Nausea History of pancreatitis Retinopathy (semaglutide)

MACE: major adverse cardiac events.

Importantly, in the DAPA-HF study, dapagliflozin showed cardiovascular benefits in patients with HF and a LVEF <40%, regardless of DM.^492^ In this trial, coronary disease was the cause of HF in 55% of the patients and a there was a 23% reduction in the composite outcome (worsening HF and cardiovascular death), regardless of the patient's HbA1c levels. Thus, in patients with LV dysfunction due to coronary disease and associated DM, there is more evidence for the benefit of SGLT2 inhibitors than GLP1 receptor agonists.

Another situation in which SGLT2 inhibitors showed benefits was in diabetic patients with chronic kidney disease. In the CREDENCE trial, canagliflozin at a dose of 100 mg per day reduced the progression of nephropathy in diabetic patients with creatinine clearance between 30 and 90 mL/min associated with proteinuria (albumin to urinary creatine ratio ≥ 300 mg/g).^493^ In end-stage renal disease, the primary endpoint (a 2-fold increase in creatinine levels or death from renal or cardiovascular causes) was reduced by 30% with the use of SGLT2 inhibitors (HR 0.70; 95% CI 0.59 to 0.82; p = 0.00001).

Most of the other antidiabetics had no effect on cardiovascular events. However, in some cases, the outcomes worsened. In the SAVOR-TIMI 53 trial, the DPP-4 inhibitor saxagliptin was tested against placebo in a population of 16,492 diabetic patients.^494^ In addition to not reducing cardiovascular outcomes, saxagliptin increased the hospitalization rate for HF (from 2.8% to 3.5%, HR 1.27; 95%CI 1.07 to 1, 51; p = 0.007). Thus, this medication should be avoided in patients with HF.

**Table:** 

Outpatient management of diabetic patients with a history of the acute coronary syndrome - Summary of recommendations and evidence		
An HbA1c target < 7% should be used in most diabetic patients to reduce microvascular events.	I	A
The HbA1c target must be individualized, considering characteristics such as comorbidities, life expect,ancy and the duration of DM	I	C
Unless contraindicated, metformin is the drug of choice for initial DM treatment.	I	A
After ACS, consider combined treatment including an SGLT2 inhibitor or a GLP1-RA with proven cardiovascular benefits regardless of HbA1c level.	I	A
In patients with HF or nephropathy (creatinine clearance between 30 and 90 mL/min and/or with proteinuria), SGLT2 inhibitors are the drug of choice.	I	A
Use saxagliptin and pioglitazone in diabetic patients with HF.	III	A

#### 3.5. Lipid lowering drugs

Treatment with statins should begin early, preferably with high-intensity therapy (ie, rosuvastatin 20-40 mg or atorvastatin 40-80 mg). In patients who are already using this medication, treatment should not be interrupted. If the previously used statin is of low or moderateintensity, changing to a high potency statin should be considered.

In outpatient follow-up, consider therapeutic LDL-c and HDL-c goals of < 50 mg/dL and < 80 mg/dL, respectively. If the goals are not achieved, and the patient is not yet using high-potency statins, prescbing a medication from this group is recommended. However, if LDL targets are still not achieved, an association with ezetimibe 10 mg/day is recommended. After these measures, if the LDL remains above the target, PCSK9 inhibitors should be considered.

The FOURIER trial (2017) was the first study to evaluate clinical outcomes associated with PCSK9 inhibitors^495^. It included 27,564 patients with a history of cardiovascular disease with LDL ≥70 mg/dL despite statin use. The patients were randomized to evolocumab (140 mg subcutaneously every 2 weeks or 420 mg subcutaneously once a month, according to the patient's preference) or placebo. A significant reduction in the composite outcome (cardiovascular death, AMI, stroke, coronary revascularization, or hospitalization due to UA) occurred in the evolocumab group. This outcome occurred in 11.3% of the control group and in 9.8% of the evolocumab group over a mean follow-up of 26 months. There was no difference in mortality.

The ODYSSEY-OUTCOMES trial (2018) evaluated alirocumab in patients with ACS in the year prior to inclusion.^496^ The patients, who had LDL ≥ 70 mg/dL despite using high-potency statins, were randomized to receive alirocumab 75 mg subcutaneously every 2 weeks or placebo. In an average follow-up of 2.8 years, there was a 14.4% reduction in the composite outcome (death from coronary heart disease, AMI, stroke, or hospitalization for UA). In this trial there was an apparent reduction in overall mortality (from 4.1% to 3.5%), although there was no reduction in cardiovascular death. The benefits of the new medication were more prominent in patients with baseline LDL above 100 mg/dL. Although the absolute risk of mortality was reduced by 0.6% in the general group, the reduction was 1.7% in the group with LDL > 100 mg/dL.

One relevant fact about the ODYSSEY-OUTCOMES trial is that the PCSK9 inhibitor was initiated an average of 2.6 months after the ACS episode. In the FOURIER study, however, in patients with a history of AMI (about 80% of the total population), evolocumab was initiated an average 3.4 years after the last AMI. In other words, based on the evidence from these studies, PCSK9 inhibitors, as a rule, should be considered in outpatient follow-up for infarcted patients and in the acute in-hospital phase.

Another lipid lowering drug that reduced cardiovascular events in secondary prevention was omega-3 supplementation (eicosapentaenoic acid: icosapent-ethyl 4 g/day). This strategy was evaluated in the REDUCE-IT study, which randomized high-risk patients (secondary prevention or DM with another associated risk factor) who had hypertriglyceridemia despite statin use.^497^ Most patients (70.7%) were onsecondary prevention. There was a reduction in the primary endpoint (cardiovascular death, AMI, stroke, need for revascularization, or UA) from 22% to 17.2%. There was also a reduction in cardiovascular death from 5.2% to 4.3% (CI 0.66 to 0.98; p = 0.03). For every 21 patients treated during the mean 4.9 years of follow-up, one cardiovascular event was prevented. One criticism about the REDUCE-IT trial was that the placebo, mineral oil, led to an LDL increase of 5 mg/dL in the control group compared to the EPA group, which could have increased the risk of events in the placebo group.

It should be pointed out that in the REDUCE-IT trial, a specific formulation of omega 3 based on eicosapentaenoic acid (icosapent-ethyl) was used, which had no relevant impact on LDL during the trial (an increase of 2 mg/dL). There is evidence that omega 3 formulations containing docosahexaenoic acid raise LDL levels.^498^ Thus, it is recommended that, for omega-3 supplementation in post-ACS patients, the REDUCE-IT formulation should be used. It will not be available in Brazil until after this Guideline has been published.

**Table:** 

Use of lipid-lowering drugs in NSTE-ACS patients after discharge - Summary of recommendations and evidence		
In patients with a previous ACS diagnosis, LDL-c should be reduced to < 50 mg/dL, and HDL-c should be reduced to < 80 mg/dL.	I	B
When tolerated, preference should be given to high-intensity statins.	I	A
When the LDL-c target is not achieved with statins at the maximum tolerated dose, ezetimibe should be added.	I	A
PCSK9 inhibitors can be considered in patients who are receiving statin treatment at the highest tolerated dose, associated or not with ezetimibe, and who have not reached recommended LDL-c or HDL-c target levels.	IIa	A

#### 3.6. Other Medications

Post-ACS patients are at increased risk of bleeding due to antithrombotic therapy. The gastrointestinal tract is the most frequent site of clinically relevant bleeding in patients on chronic antiplatelet therapy.^499^ Gastric protection is one way to reduce the risk of these events. PPIs have been shown to be more effective than H2 receptor antagonists in observational studies.^500^ However, some in vitro studies have suggested that associating PPIs with clopidogrel could reduce its antiplatelet activity.^225^^,^^226^ The COGENT trial (see Part 2) showed that combining omeprazole with clopidogrel reduced the risk of gastrointestinal complications without an apparent increase in ischemic events. However, given that the study was discontinued early, it cannot be ruled out that omeprazole would have led to an increase in ischemic events.^231^

Therefore, PPIs are recommended for patients receiving DAPT who are at increased risk of bleeding, i.e., those with a history of gastrointestinal bleeding, a history of peptic ulcer disease, anticoagulant use, chronic NSAID use, chronic corticosteroid use, or those with two or more of the following factors: age ≥ 65 years, dyspepsia, gastroesophageal reflux disease, H. pylori infection, or chronic alcohol use.

After discharge, patients must be instructed that if they have angina at rest, they should use sublingual nitrate (dinitrate, mononitrate, or nitroglycerin), as long as there are no contraindications. If the pain does not improve in 3 to 5 minutes, the emergency department should be called.

Viral and bacterial infections can trigger an acute cardiovascular event in patients with heart disease. There is evidence that vaccinating for influenza and pneumococcus can reduce hospitalizations and even mortality in these patients.^501^ Thus, annual vaccination for influenza is recommended for patients with coronary disease. Ideally, the vaccine should be administered during the normal vaccination campaign (April and May in Brazil). Regarding the pneumococcal vaccine, a dose of the 23-valent polysaccharide (Pneumo 23) vaccine with a booster after 5 years is recommended.^502^ An ongoing Brazilian randomized trial is testing whether a double-dose of anti-influenza vaccine during hospitalization for ACS is superior to conventional vaccination 30 days after the event for reducing cardiovascular outcomes (NCT 04001504).

Therapy aimed at reducing plaque inflammation has begun to yield promising results. In the CANTOS trial, canakinumab, an anti-interleukin 1-β monoclonal antibody, reduced major cardiovascular events (cardiovascular death, reinfarction or stroke) in patients with previous infarction (at least 30 days after the index event) and elevated C-reactive protein (HR 0.88; 95% CI 0.79 to 0.97; p = 0.02).^503^ However, there was an increase in death from infection. Moreover, due to its prohibitive cost, this medication has not been incorporated into normal clinical practice. Colchicine, a widely available and low-cost drug, significantly reduced the composite outcome (cardiovascular death, reinfarction, hospitalization for UA with a need for revascularization, stroke, or resuscitated cardiac arrest (HR 0.77; 95% CI 0.61 to 0.96; p = 0.02) in patients with recent AMI (within 30 days of the index event). However, there was an increase in the incidence of pneumonia.^504^ Figure 3.4 summarizes the recommended drugs for NSTE-ACS patients after discharge.

**Table:** 

Other pharmacological interventions in ACS patients after discharge - Summary of recommendations and evidence		
If there are no contraindications, patients should use sublingual nitrate if they have angina at rest.	I	C
Patients should be vaccinated against influenza and pneumococcus to reduce morbidity and mortality.	I	B
PPI should be used in patients receiving DAPT who have an increased risk of bleeding (history of gastrointestinal bleeding or peptic ulcer disease, anticoagulant use, chronic NSAID use, chronic corticosteroid use) or have two or more of the following characteristics: age ≥ 65 years, dyspepsia, gastroesophageal reflux disease, H. pylori infection, or chronic alcohol use.	IIa	B

**Figure 3.4:** Summary of discharge prescriptions after non-ST-elevation acute coronary syndrome.

### 4. Post-discharge Clinical Screening

When early reassessment is recommended for low-risk patients and those revascularized after NSTE-ACS, it should be performed between 2 and 6 weeks after discharge. In patients with greater severity, reassessment is recommended within 14 days.^505^ Obviously, these deadlines must consider the particularities of each situation since the procedural complications, health care access, and individual characteristics of each patient can vary considerably.

Since there is a risk of complications even in asymptomatic patients, risk assessment should be applied to both symptomatic and asymptomatic patients. Patients should be closely monitored in the first 12 months after revascularization and/or stabilized ACS because they are at higher risk for complications and are subject to changes in pharmacological treatment.^506^ Therefore, at least two visits are recommended in the first year of follow-up, the first occurring ideally within 3 months.

In patients with LV systolic dysfunction prior to the revascularization procedure or after ACS, LV function should be reassessed 8 to 12 weeks after the intervention.^507^^,^^508^

Cardiac function may improve due to mechanisms such as recovery from myocardial dizziness or hibernating myocardium, which can be reversed through revascularization or worsened due to other comorbidities, such as heart valve disease, arrhythmia, inflammation, etc. These conditions should be identified and treated.^509^

Similarly, non-invasive ischemia testing can be performed after revascularization to identify residual ischemia and serve as a reference for subsequent comparisons.^509^ This approach must be individualized since each revascularization involves anatomical peculiarities and varies greatly from patient to patient. These recommendations are mostly based on expert opinion.

In patients who have been asymptomatic for more than 1 year, at least one annual reassessment is recommended.^509^ The assessment should include the patient's general clinical status, treatment adherence, and risk profile based on risk scores. Laboratory tests, including a lipid profile, kidney function, a complete blood count, and biomarkers, should be performed at least every 2 years.^507^^,^^508^

The lipid profile and glycemic status should be evaluated periodically and, although there is no evidence to support a specific periodicity, an annual evaluation is generally recommended.^509^

Certain inflammatory biomarkers appear to be event markers. The highsensitive C-reactive protein has been tested in multiple studies, even in patients in primary prevention. NT-proBNP, von Willebrand factor, and interleukin-6 also seem to predict events.^510^ Scores based on an association of biomarkers (LDL, NT-proBNP, troponin T, fibrin degradation products) have been found to have better reclassification power and higher C-statistics than scores based on clinical models, and they are promising tools for predicting events in patients with coronary disease.^511^^,^^512^ However, the lack of more robust evidence limits the routine application of these biomarkers, which should be reserved for selected cases.

An ECG should be requested at each visit to determine heart rate and rhythm. Echocardiograms can help assess LV function (diastolic and systolic), valve status, and cardiac dimensions in apparently asymptomatic patients every 3 to 5 years. Likewise, it may be beneficial to non-invasively assess the presence of silent ischemia in apparently asymptomatic patients every 3 to 5 years, preferably with stress imaging tests.^513^

Due to the lack of functional information, coronary CCTA should not be used for evaluation. In specific cases, it can help assess the patency of coronaries and grafts.^512^

For patients with a history of NSTE-ACS and/or revascularization and unequivocal symptoms of angina, the vast majority of evidence indicates that invasive stratification is the best option. For patients with unclear symptoms, stress imaging is recommended.^513^

**Table:** 

Post-discharge clinical screening - Summary of recommendations and evidence		
Asymptomatic patients		
Patients should visit a cardiologist (preferably within 3 months) to reassess any potential changes in their risk situation, including clinical assessment of lifestyle modification measures, adherence to the target risk factors, and new comorbidities that could affect treatment and results.	I	C
In patients with mild or no symptoms in whom non-invasive risk stratification indicates high risk and for whom revascularization could improve prognosis, invasive coronary angiography is recommended (FFR, when necessary and available).	I	C
Risk stratification by stress imaging can be performed in high-risk patients approximately 6 months after revascularization.	IIb	C
A new angiography may be considered in high-risk revascularization patients (e.g., unprotected left main coronary artery disease), regardless of the symptoms.	IIb	C
Routine stress imaging should be performed in patients who were revascularized percutaneously > 1 year or surgically > 5 years.	IIb	C
CCTA alone can be used for routine stratification.	III	C
Coronary angiography alone can be used for risk stratification in an asymptomatic patient.	III	C
**Asymptomatic patients**		
Evaluate coronary disease status in patients who have impaired LV systolic function with no identifiable cause.	I	C
Perform stress imaging (preferably) in patients with new symptoms and/or worsening symptoms.	I	C
Perform angiography (with FFR or iFR, if necessary) in patients with unmistakable symptoms of coronary disease, especially if it is refractory to drug treatment or if they fit the high-risk profile.	I	C
Perform angiography in patients with high-risk stress imaging findings.	I	C
In a previously revascularized patient, stress imaging should be performed rather than ET.	IIa	C
